# Outbred genome sequencing and CRISPR/Cas9 gene editing in butterflies

**DOI:** 10.1038/ncomms9212

**Published:** 2015-09-10

**Authors:** Xueyan Li, Dingding Fan, Wei Zhang, Guichun Liu, Lu Zhang, Li Zhao, Xiaodong Fang, Lei Chen, Yang Dong, Yuan Chen, Yun Ding, Ruoping Zhao, Mingji Feng, Yabing Zhu, Yue Feng, Xuanting Jiang, Deying Zhu, Hui Xiang, Xikan Feng, Shuaicheng Li, Jun Wang, Guojie Zhang, Marcus R. Kronforst, Wen Wang

**Affiliations:** 1 State Key Laboratory of Genetic Resources and Evolution, Kunming Institute of Zoology, Chinese Academy of Sciences, Kunming 650223, China; 2 BGI-Shenzhen, Shenzhen 518083, China; 3 Department of Ecology and Evolution, University of Chicago, Chicago, Illinois 60637, USA; 4 City University of Hongkong, Hongkong, China; 5 Department of Evolution and Ecology, University of California Davis, Davis, California 95616, USA; 6 University of Chinese Academy of Sciences, Beijing 100049, China; 7 School of Bioscience and Biotechnology, South China University of Technology, Guangzhou 510641, China; 8 Centre for Social Evolution, Department of Biology, Universitetsparken 15, University of Copenhagen, Copenhagen DK-2100, Denmark

## Abstract

Butterflies are exceptionally diverse but their potential as an experimental system has been limited by the difficulty of deciphering heterozygous genomes and a lack of genetic manipulation technology. Here we use a hybrid assembly approach to construct high-quality reference genomes for *Papilio xuthus* (contig and scaffold N50: 492 kb, 3.4 Mb) and *Papilio machaon* (contig and scaffold N50: 81 kb, 1.15 Mb), highly heterozygous species that differ in host plant affiliations, and adult and larval colour patterns. Integrating comparative genomics and analyses of gene expression yields multiple insights into butterfly evolution, including potential roles of specific genes in recent diversification. To functionally test gene function, we develop an efficient (up to 92.5%) CRISPR/Cas9 gene editing method that yields obvious phenotypes with three genes, *Abdominal-B*, *ebony* and *frizzled*. Our results provide valuable genomic and technological resources for butterflies and unlock their potential as a genetic model system.

## Abstract (web summary)

Butterflies are a promising system to study the genetics and evolution of morphological diversification, yet genomic and technological resources are limited. Here, the authors sequence genomes of two *Papilio* butterflies and develop a CRISPR/Cas9 gene editing method for these species.

## Graphical abstract

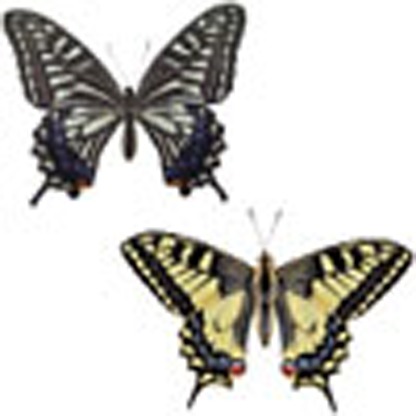

Butterflies are famous for their extraordinarily diverse wing patterns, which differ not only among species but also among populations, sexes and even seasonal forms^1^,^2^. Wing patterns are highly variable, because they are multifunctional, involved in roles from crypsis to warning colouration, mimicry, thermoregulation and mate selection^2^,^3^. Beyond wing pattern, butterflies are also diverse in virtually all aspects of their biology, ranging from behaviour and biogeography to cellular biology and biochemistry, with decades of study having placed much of this variation in a well-resolved ecological context^4^. These features make butterflies a promising system to explore the genetics, evolution and development of morphological diversification and speciation^5^,^6^.

Although butterflies have notable strengths in terms of natural variation and ecology, they also suffer critical shortcomings in the quest to characterize the genetic basis of organismal phenotypes, in particular difficulty in deciphering usually heterozygous genomes and a lack of functional genetics methodology. For instance, although there are estimated 18,000 butterfly species, there are currently only 6 butterfly genome sequences^7^,^8^,^9^,^10^,^11^. If additional, high-quality genomes from closely related species were available, it would be feasible to comprehensively trace genetic changes responsible for phenotypic change over evolutionary time. However, the nature of high heterozygosity in most wild insects, including butterflies, has hampered efforts to obtain high-quality reference genomes^7^,^12^,^13^. In addition, efficient and precise genetic manipulation technologies are indispensable for a model organism. However, general and efficient genetic manipulation of butterflies has not been reported, which, together with limited genomic resources, has greatly restricted their application as model organisms. The only case of genome editing in a butterfly is the use of zinc-finger nucleases in the monarch butterfly^14^. Recently, clustered regulatory interspaced short palindromic repeat (CRISPR)-associated (Cas)-based RNA-guided DNA endonucleases such as the *Streptococcus pyogenes* Cas9 nuclease (CRISPR/Cas9) has emerged as an efficient tool for gene editing across a wide spectrum of organisms^15^,^16^, including insects such as fruitfly *Drosophila melanogaster*^17^,^18^ and silkworm *Bombyx mori*^19^,^20^.

*P. xuthus* and *P. machaon* are two closely related swallowtail butterfly species from the most basal lineage of butterflies, the family Papilionidae. Despite being closely related, *P. xuthus* and *P. machaon* differ in many aspects of their biology, including adult and larval colour pattern, larval host plants and geographic distribution, with *P. xuthus* mainly distributed in East Asia and *P. machaon* widely distributed across Asia, Europe and North America. Papilionidae is one of the most historically significant groups of butterflies; *P. machaon* was named as the type species for all butterflies by Linnaeus^21^ and since then the group has been a long-term focus in the study of mimicry, vision and learning, and pigmentation^3^.

Here we present high-quality reference genomes for the two highly heterozygous and closely related butterflies *P. xuthus* and *P. machaon*. Integrating comparative genomics and analyses of gene expression yields multiple insights into butterfly evolution. We develop an efficient and widely applicable CRISPR/Cas9 gene editing method that results in obvious phenotypes with three genes, *Abdominal-B* (*Abd-B*), *ebony* and *frizzled (fz)*. Our results provide valuable genomic and technological resources for butterflies and unlock their potential as a genetic model system.

## Results

### Genome sequencing and assembly

**Figure 1:**
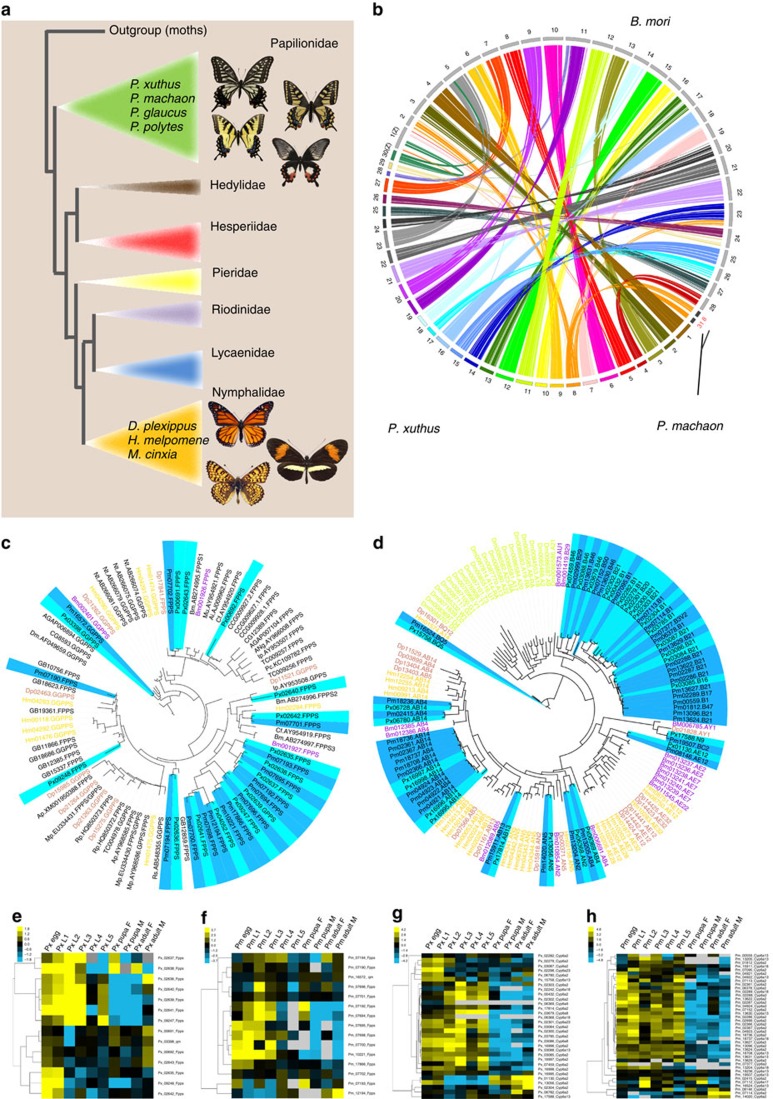
Butterfly comparative genomics. (**a**) Phylogeny of butterfly families^58^ showing relationship of *P. xuthus* and *P. machaon* to *D. plexippus*, *H. melpomene*, *M. cinxia*, *P. glaucus* and *P. polytes*. (**b**) Chromosome mapping of *P. xuthus* (*n*=30) to *B. mori* (*n*=28). For *P. machaon* (*n*=31), we only plot chromosome 8 (chr8) and chr31, which were fused in *P. xuthus*. (**c**) Maximum-likelihood tree showing strong expansion of the *scIPPS* genes in the genomes of swallowtail butterflies. Included are all *scIPPS* genes identified in the genomes of ten holometabolous insects (*P. xuthus*, *P. machaon*, *D. plexippus*, *H. melpomene*, *B. mori*, *P. xylostella*, *A. gambiae*, *D. melanogaster*, *T. castaneum* and *A. mellifera*). The clades of *P. xuthus* and *P. machaon* are highlighted by light blue and deep blue, respectively. (**d**) Maximum-likelihood tree showing expansions of *CYP6B* genes in the genomes of *P. xuthus* and *P. machaon*, and of the *CYP6AB* genes in the genome of *P. machaon*, as compared with *CYP6* genes of *D. plexippus*, *H. melpomene*, *B. mori* and *D. melanogaster*. In **c**,**d**, the clades of *P. xuthus* and *P. machaon* are highlighted by light blue and deep blue, respectively. (**e**,**f**) Expression profiles of *FPPS* and *GGPPS* genes at all development stage of *P. xuthus* (**e**) and *P. machaon* (**f**). (**g**,**h**) Expression profiles of *CYP6* genes at all development stage of *P. xuthus* (**g**) and *P*. *machaon* (**h**). Expression measured in reads per kilobase of transcript per million reads mapped (RPKM).

We collected *P. xuthu*s and *P. machaon* samples from the suburb of Ya’an (Sichuan, China) (Supplementary Fig. 1). The heterozygosity of the two species was high, 1.008% for *P. xuthu*s and 1.229% for *P. machaon*, and initial assembly using standard Illumina next-generation sequencing data resulted in poor assemblies (Supplementary Note 1, Supplementary Tables 1–3 and Supplementary Fig. 2). Therefore, we used 454FLXPlus long reads (≥700 base pair (bp)) in combination with a large quantity of Illumina short reads (100–150 bp, >87 × ), to assemble contigs, and we then used Illumina long-insert mate-pair sequencing data to generate scaffolds (Supplementary Note 1, Supplementary Tables 1–11 and Supplementary Figs 2–6). This hybrid assembly approach eliminated issues due to high heterozygosity, yielding a final assembly comprising 244 Mb with contig N50 of 492 kb and scaffold N50 of 3.4 Mb for *P. xuthus* and a 281-Mb assembly with contig N50 of 81 kb and scaffold N50 of 1.15 Mb for *P. machaon* (Table 1 and Supplementary Tables 6 and 7). Three evaluation methods show that our assemblies are quite complete and reliable (Supplementary Note 1 and Supplementary Tables 8–11). The assembled genome sizes are consistent with estimates by both *k-mer* analysis (Supplementary Table 3) and by flow cytometry (Supplementary Table 5). Notably, the contig N50 sizes for both genomes are large compared with those of all published Lepidoptera genomes (Fig. 1a), and the contig N50 size of *P. xuthus* genome is the largest among all published animal genomes excluding such classical models as fruitfly *D. melanogaster*, human *Homo sapiens*, mouse *Mus musculus* and rat *Rattus norvegicus* (Supplementary Table 12).

We generated a linkage map using 74 offspring from a *P. xuthus* cross and used it to assemble 87% of the *P. xuthus* genome into 30 chromosomes (Supplementary Note 2, Supplementary Table 13 and Supplementary Fig. 7). Based on the *P. xuthus* chromosomal assembly, we compared syntenic relationships among *P. xuthus*, *P. machaon* and *B. mori* (Fig. 1b), and found that chromosome 8 (chr8) of *P. xuthus* resulted from a fusion of ancestral lepidopteran chr8 and chr31. The Z chromosome (that is, chr30) of *P. xuthus* is highly homologous to that of *B. mori*, although a large fragment of *P. xuthus* chrZ is homologous to a region of *B. mori* chr5.

The assembled genomes of *P. xuthus* and *P. machaon* have similar composition of repetitive element (Supplementary Note 3, Supplementary Tables 14–16 and Supplementary Fig. 8) and are predicted to contain 15,322 and 15,499 protein-coding genes, respectively (Table 1, Supplementary Note 4, Supplementary Tables 17 and 18, and Supplementary Figs 9–11). More than 80% of gene models are supported by evidence from at least two prediction methods (*ab initio*, homology and RNA sequencing (RNA-seq); Supplementary Fig. 9) and both genomes show similar gene features to those of other Lepidoptera (Supplementary Table 17 and Supplementary Figs 10 and 11). About 50% of gene families are conserved in butterflies (Supplementary Table 18 and Supplementary Fig. 12). We inferred a phylogenetic tree among representative taxa spanning holometabolous insects including seven lepidopteran species (*P. xuthus*, *P. machaon*, *Heliconius melpomene*, *Danaus plexippus*, *Melitaea cinxia*, *B. mori* and *Plutella xylostella*) using 1,071 single-copy orthologues (Supplementary Note 5 and Supplementary Fig. 13). We analysed lineage-specific genes and gene families, and identified positively selected genes (Supplementary Note 5, Supplementary Tables 19–26 and Supplementary Figs 13 and 14). We also obtained genome-wide gene expression profiles for *P. xuthus* and *P. machaon* at each of ten development stages: egg, each of the five larval instars, male pupa, female pupa, male adult and female adult (Supplementary Note 6, Supplementary Tables 27–30 and Supplementary Figs 15–19). By integrating these multiple layers of information, we inferred important genes and gene pathways in butterfly evolutionary history and then we developed an efficient and widely applicable genome-editing method for butterfly functional genetics.

Assembly, gene annotations, gene family and developmental transcriptome can be obtained from ftp://ftp.genomics.org.cn/pub/papilio.

### Comparative genomics

Comparison with other insect genomes revealed interesting evolution patterns, in particular evolution in the juvenile hormone (JH) pathway in tandem with butterfly morphological diversification. JHs are key hormones in insect development. There are six different JH forms in insects and, interestingly, Lepidoptera have five of the six JH forms, while other insects generally have only one JH form^22^. Short-chain isoprenyl diphosphate synthases (scIPPSs) are key enzymes in the JH synthesis pathway^23^ (Supplementary Fig. 20). Based on comparisons across holometabolous insects, all butterflies experienced *scIPPS* gene expansion (Fig. 1c, Supplementary Note 7 and Supplementary Tables 31 and 32). Furthermore, scIPPSs can be classified into farnesyl pyrophosphate synthases (FPPSs) and geranylgeranyl pyrophosphate synthases (GGPPSs). In butterflies, *FPPS* genes expanded from 1 copy to 14 copies in *Papilio*, while *GGPPS* genes expanded from 1 copy to 7–8 copies in nymphalid butterflies (Fig. 1c and Supplementary Table 32). It has been suggested that in moths it is alternative splicing of the *scIPPS* genes that contributes to diverse JH forms^24^. In addition, notable expansions of genes encoding JH epoxide hydrolase and JH diol kinase in *Papilio* (Supplementary Table 31 and Supplementary Fig. 21), combined with the prominent expansion of *scIPPS* genes in butterfly JH biosynthesis, suggest significant diversification of JH metabolism in butterflies, which may be related to the evolution of their numerous morphological forms. Interestingly, the *scIPPS* genes may have also played roles in the differentiation among *Papilio* species, because we observed that although *P. machaon* and *P. xuthus* share the same 14 *FPPS* genes, these orthologues were differentially expressed between *P. machaon* and *P. xuthus* (Fig. 1e,f and Supplementary Table 33). Collectively, the above observations suggest that scIPPSs might have played roles in both the deep evolutionary history of butterflies as well as very recent divergence between closely related species.

Comparative genomics also revealed evidence for genes critical in host plant adaptation. Although *P. machaon* and *P. xuthus* are congeneric species, their host plants are different. As larvae, *P. xuthus* feeds on plants in the family Rutaceae, such as citrus, whereas *P. machaon* feeds on plants in the family Apiaceae. Both groups of plants contain toxic furanocoumarins, but Apiaceae have higher levels of furanocoumarins than Rutaceae^25^. By comparing *P. xuthus* and *P. machaon* genomes with those of *D. plexippus*, *H. melpomene*, *B. mori* and *D. melanogaster*, we found *CYP6*, a cytochrome P450 monooxygenase (*P450*) gene family, expanded in swallowtail butterflies (Supplementary Note 8, Supplementary Table 34 and Supplementary Fig. 22). CYP6, together with CYP9 and α-esterase families, are thought to contribute to xenobiotic detoxification in insects^26^. We observed that 50% of *CYP6* members in the two *Papilio* genomes belong to the *CYP6B* subfamily, while there is no *CYP6B* in the monarch or passion-vine butterfly genomes, species whose host plants have no toxic furanocoumarins (Fig. 1d, Supplementary Table 35 and Supplementary Fig. 22). Higher expression of *CYP6* genes in larvae of *P. machaon* and *P. xuthus*, compared with other developmental stages (Fig. 1g,h), suggests that *CYP6* genes may play an important role in larval feeding. *CYP6AB* is another *P450* subfamily found only in Lepidoptera. We found additional expansions of *CYP6B* and *CYP6AB* in *P. machaon* only (Fig. 1d and Supplementary Fig. 22), which may account for this species’ ability to feed on Apiaceae plants with a higher content of furanocoumarins.

### Genomic divergence among swallowtail species

There are striking morphological differences between *P. xuthus* and *P. machaon*, especially at the fifth-instar larval ontogenetic stage (Supplementary Fig. 1). Similar to caterpillars of hundreds of other tropical butterfly species, the fifth instar larvae of *P. xuthus* display false eyespots on their metathorax, which permit them to escape predation by mimicking venomous snakes^27^,^28^. On the other hand, fifth instar larvae of *P. machaon* exhibit disruptive colouration characterized by highly contrasting patterns of black and green stripes with yellow spots, providing camouflage that conceals body shape at a distance, while serving as warning colouration at close range^29^,^30^. We found a number of genes related to morphological traits that were expanded, positively selected, or differentially expressed in *P. xuthus* and *P. machaon* (Supplementary Notes 9–11, Supplementary Tables 36–40 and Supplementary Figs 22–36). Butterfly larvae, swallowtails’ in particular, frequently have marked green pigmentation, which consists of blue bile binding to bilin-binding protein and a yellow component binding to proteins encoded by yellow-related gene^31^,^32^. Interestingly, we observed an expansion of the bilin-binding protein gene family in butterflies, especially in swallowtails (Supplementary Table 36 and Supplementary Fig. 32), which may be related to the exceptionally green caterpillars in swallowtails. We also identified a *Papilio*-specific single-copy yellow-related gene and found it was expressed at a much higher level in the fifth instar larvae of *P. machaon* compared with that of *P. xuthus* (Supplementary Table 36 and Supplementary Figs 33 and 34), corresponding to the stronger yellow marking in *P. machaon* (Supplementary Fig. 1).

**Figure 2:**
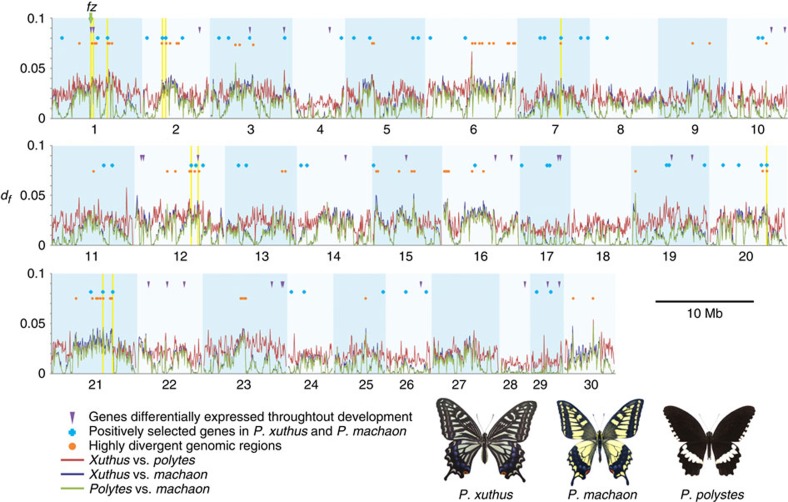
Genome-wide divergence among *Papilio* species. Pairwise genetic divergence among *P. xuthus*, *P. machaon* and *P. polytes* was calculated in 50-kb windows. This yielded 70 highly divergent genomic regions, windows in the upper 95th percentile in all three comparisons, encompassing 915 genes. When overlaid with signatures of positive selection and differential expression throughout development, 11 genes (highlighted in yellow) emerged as strong candidates for a role in recent diversification, including *frizzled*. Degree of freedom (df) is the density of different single-nucleotide polymorphisms (SNPs) per bp of each 50-kb window.

To better characterize patterns of genome evolution among closely related swallowtail species, we performed a genome-wide sliding window analysis of DNA sequence divergence among *P. xuthus*, *P. machaon* and a third *Papilio* species, *Papilio polytes* (Fig. 2, Supplementary Note 12, Supplementary Tables 41–43 and Supplementary Fig. 37) with its resequencing data available^33^. A total of 915 genes showed strong divergence in all pairwise comparisons. By integrating this with results from our analyses of recent positive selection and differential gene expression, and looking for overlap among data sets, we found a small subset of genes that might have played an important role in recent diversification among these three closely related *Papilio* species (Supplementary Table 43). One particular gene was *frizzled (fz)*. We found that the genome window containing the 5′-untranslated region of *fz* emerged as a highly divergent segment in all pairwise comparisons among the three *Papilio* species, but the coding region itself did not. This gene also showed strong differential expression between *P. xuthus* and *P. machaon* across all developmental stages (Supplementary Note 11, Supplementary Table 39 and Supplementary Fig. 36), 1 of only 32 genes to do so (and 1 of only 4 highly divergent genes to do so) (Supplementary Fig. 17). *Frizzled* is well-known for its role in polarizing epithelia during development (planar cell polarity, PCP), but it also plays a distinct role patterning the larval cuticle during embryogenesis^34^. In *Drosophila*, *fz* interacts with *wingless*, *armadillo* and other genes, to determine the distribution of naked cuticle versus denticle on larvae^35^. Given the highly divergent larval morphology among swallowtails, we hypothesized that *fz* may play a similar role in influencing larval cuticle development, in which case DNA sequence and expression divergence at this gene could be a result of natural selection on larval anatomy. Furthermore, another PCP pathway (*Fat/Dachsous*) that also influences larval denticle patterning in *Drosophila*^36^ showed evidence of recent, positive selection in *P. machaon* (Supplementary Tables 38–40). Experimentally, testing genes’ potential role in morphological diversification among *Papilio* species required functional genetics methodology not available in butterflies.

### CRISPR/Cas9 system

**Figure 3:**
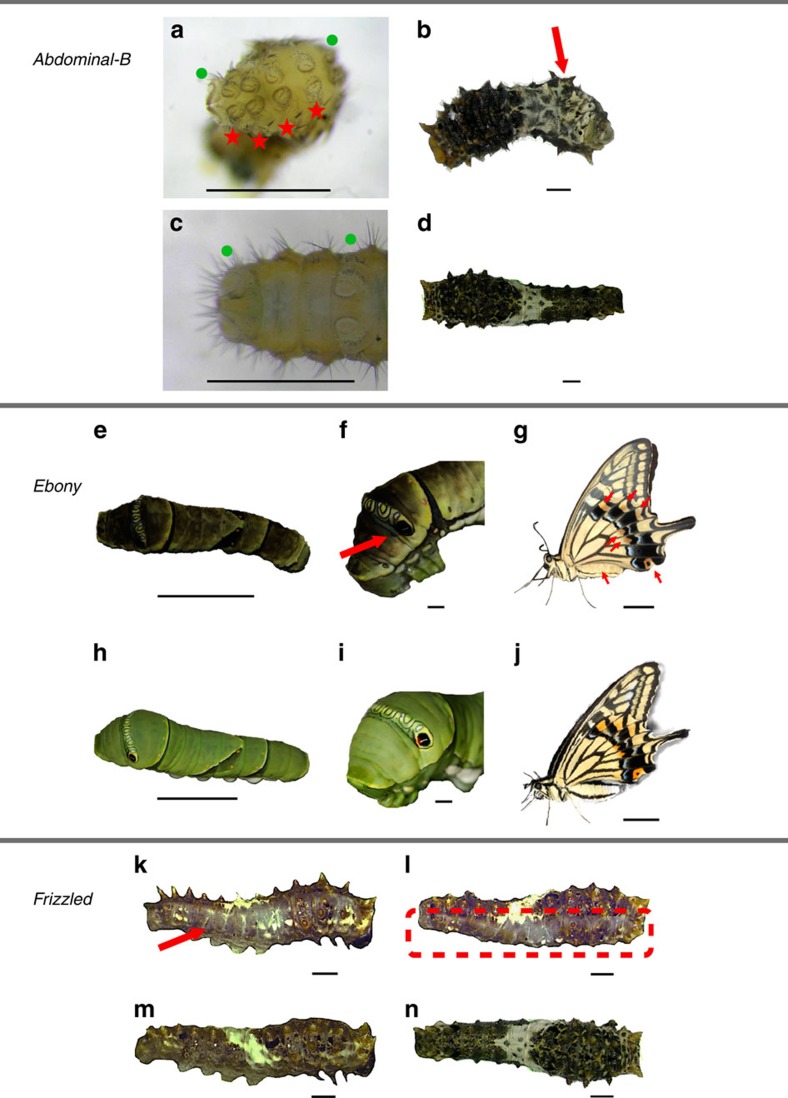
CRISPR/Cas9-induced morphological mutants of *Abdominal-B*, *ebony* and *frizzled* in *P. xuthus*. (**a**–**d**) Mutations induced by the injection of Cas9 mRNA and *Abdominal-B* sgRNA with prolegs on segment A7–A10 (**a**), segments that do not normally have prolegs (**c**), or with a curled abdomen resulting from abnormal terga on segments A3 and after (**b**), compared with wild type (**d**). Green dots in **a** and **c** show normal prolegs of A6 and A10, while red stars in **a** denote the redundant prolegs on A7–A10. (**e**–**j**) Mutations induced by the injection of Cas9 mRNA and *ebony* sgRNA with enhanced melanic pigmentation (**e**) and an absence of orange colour in the false eyespot (**f**) in fifth instar larvae, compared with wild type (**h**,**i**), and with brown pigmentation (**g**) across the body in regions that were normally yellow and in wing patches that were normally orange in adult (**j**). (**k**–**n**) A mutant induced by the injection of Cas9 mRNA and *frizzled* sgRNA with smooth and colourless dorsal cuticle in right side (**k**), while normal in its left side (**m**), and its dorsal view (**l**) compared with wild type (**n**). Scale bars, 1 mm (**a**–**d**,**f**,**i**,**k**–**n**) and 10 mm (**e**,**g**,**h**,**j**).

Given the recent, widespread utility of CRISPR/Cas9 (refs 15, 16), we decided to implement a CRISPR/Cas9 genome-editing approach in *P. xuthus* (Table 2, Supplementary Note 13, Supplementary Tables 44–50 and Supplementary Figs 38–55). Before targeting candidate diversification genes, such as *fz*, we developed our pipeline (Supplementary Fig. 38) by focusing on the gene *Abd-B* and we then verified it with *ebony* (Supplementary Table 44 and Supplementary Fig. 39), because these two genes are expected to have predictable and visible morphological phenotypes. *Abd-B* is a HOX gene that plays a critical role in determining cell fate in the tail end of the organism. For instance, mutation or decreased expression of *Abd-B* causes development of extra prolegs on all segments posterior to the sixth abdominal segment (A6) in silkworm larvae^37^. We began our *Abd-B* gene-editing experiment with low concentrations of single guide RNAs (sgRNAs) (150 ng μl^−1^) and Cas9 messenger RNA (300 ng μl^−1^) but failed to observe morphologically mutated individuals, although sequencing yielded evidence of low-frequency disruptions (Supplementary Table 45 and Supplementary Fig. 40). Increasing injection concentrations (sgRNAs:Cas9 (ng μl^−1^), 200:300, 600:600) or the ratio (sgRNAs/Cas9 mRNA (ng μl^−1^): 100/150) gave rise to a low frequency (7.4%) of morphologically mutated larvae with varying abnormalities (Supplementary Tables 45 and 46, and Supplementary Figs 41–44). Remarkably, further increasing injection concentration of sgRNAs (982 ng μl^−1^) and Cas9 mRNA (1,200 ng μl^−1^), together with shortening the time period from egg laying to injection from 4 to 2 h, resulted in both a high frequency (92.5%) of morphologically mutated individuals and a majority (90%) of mutated individuals with severe expected phenotypic abnormalities (Fig. 3a–d, Supplementary Tables 45 and 46, and Supplementary Fig. 43a). For example, all mutated individuals had a curled abdomen resulting from abnormal terga on segments A3 and after, and most individuals had prolegs on all or part of segments A7–A10, segments that do not normally have prolegs (Supplementary Tables 45 and 46, and Supplementary Fig. 43). Sequencing of the targeted region showed a high frequency of gene disruption (Supplementary Table 46 and Supplementary Fig. 42). In addition, we also tested whether expression of the target gene (*Abd-B*) at RNA or protein level disappeared or was extremely reduced in mutants using both the quantitative reverse transcription–PCR (qRT–PCR) and western blotting. Both qRT–PCR and western blotting results suggested that the level of Abd-B protein in mutants was extremely low compared with that of the wild-type individuals (Supplementary Note 13 and Supplementary Fig. 45a,b), further providing evidence of *Abd-B* gene disruption. Based on our experience with *Abd-B*, we conclude that the key factors for successful gene editing in butterflies include high concentrations of sgRNA and Cas9 mRNA, an appropriate ratio, mixed injection of two or more sgRNAs with close targeting sites and timing egg injection to target early embryogenesis (Supplementary Note 13). It is noteworthy that all the mutant test experiments used individuals that developed from injected eggs (G_0_), and DNA, RNA, as well as protein were extracted from G_0_ whole body samples.

Having established the methodology, we further tested it on a second proof-of-principle gene, *ebony*. *Ebony* encodes the enzyme *N*-β-alanyl dopamine synthetase, a central component of melanin biosynthesis (Supplementary Fig. 25), and expresses not only in pupa and adult but also larvae of *P. xuthus* and *P. machaon*, including the green region of both larvae (Supplementary Figs 26, 28c and 30). Swallowtail butterflies have a unique class of wing pigments, papiliochromes, that derive in part from melanin precursors such as tyrosine and β-alanine via the action of *ebony*^38^. *Ebony* is also known to influence behaviour^39^. We obtained many *ebony* mutants of *P. xuthus* with the CRISPR/Cas9 system (Fig. 3e–j, Supplementary Note 13, Supplementary Tables 47 and 48 and Supplementary Figs 46–51). Mutated fifth instar larvae displayed enhanced melanic pigmentation (Fig. 3e and Supplementary Fig. 46), consistent with *ebony* mutants in other insects^40^,^41^,^42^, and they also showed an absence of orange colour in the false eyespot (Fig. 3f and Supplementary Fig. 46). Interestingly, two mutated fifth instar larvae also showed abnormal curticular structure in the location of eversible osmeterium, a swallowtail’s specific defensive organ, and one or both of tubular arms of their osmeterium, unlike those of wild-type individuals, could not evert when irritated by abrupt contact (Supplementary Fig. 47). Adult mutants showed brown pigmentation across the body and the regions of wings that were normally yellow (Supplementary Fig. 48). We also verified the decreased expression of *ebony* in the fifth instar larvae of mutants compared with that of wild type by qRT–PCR (*t*-test, *P*=0.001) (Supplementary Note 13 and Supplementary Fig. 51), suggesting that *ebony* gene was largely disrupted in mutants.

Finally, we turned to the candidate diversification gene *fz*. Consistent with our hypothesis that *fz* plays a role in generating divergent larval morphology, we obtained mutants showing a variety of larval anatomy phenotypes, including asymmetric appendages and cuticular structures (Fig. 3k–n, Supplementary Tables 49 and 50, and Supplementary Figs 52–55). For example, mutated larvae had smaller prolegs on one side of the body, smooth and colourless dorsal cuticle, or vestigial tubercles on the prothorax or metathorax. These results suggest that divergent evolution at the *fz* gene, as well as differential expression, may be related to the distinct larval anatomy between species.

To exclude the possibility that these morphological mutants were induced by off-target cleavage events during the genome-editing process, we further identified all possible off-target sites in the genome using three methods (CasOT^43^, Cas-OFFinder^44^ and COSMID^45^) with the mismatches up to 5 bp. For the two target sites (Px_10703_e-T454 and -T6) of *ebony*, the three methods identified 197 possible off-target sites, but none of them is completely identical to the target sites (Supplementary Note 13 and Supplementary Table 51). Next, we performed whole-genome resequencing for an *ebony* fifth instar larva mutant (Supplementary Note 13). Our resequencing data confirmed disruption of the two target sites of the *ebony* gene (Supplementary Fig. 56), but there was no disruption of any other functional gene in the mutant (Supplementary Table 52). Among the 197 possible off-target sites of *ebony* T454 (143 sites) and T6 (54 sites), we observed that 33 sites have variation different from the reference genome. Among these 33 sites, 29 are due to single nucleotide variations (SNVs) and 4 are due to indels. Three SNVs are found at the possible off-target sites of three genes, respectively, but these three SNVs are all synonymous. Other 30 sites are all in non-coding regions. Therefore, the observed phenotypes are most possibly caused by the designed target genes’ disruption rather than off targeting. The 33 variation sites that did not disrupt genes could result from polymorphisms given the high heterozygosity of *P. xuthus*, although the possibility of off targeting could not be completely excluded at this point. High specificity of Cas9 editing and low incidence of off-target mutations have recently been reported by whole-genome sequencing of human stem cell^46^,^47^; in butterflies, future detailed analysis on offsprings from the same parents will allow to test whether some of the variations are from off targeting or not. Nevertheless, our results show that Cas9 is an efficient and reliable tool in butterfly gene editing.

It is worth noting that we have only focused on *P. xuthus* at this point, because we found difficult to raise *P. machaon* under laboratory conditions. However, our experience suggests that this Cas9 method will be widely applicable to any butterfly species as long as one can collect a sufficient number of eggs and then rear them to an appropriate developmental stage to observe mutants’ phenotypes.

## Discussion

The amazing diversity of butterflies provides a rich, natural experiment with which to explore the molecular mechanisms of morphological and species diversification, but doing so with butterflies has been hindered by complications associated with assembling genome sequences from highly heterozygous, outbred insects and an absence of widely applicable gene-editing technology. By focusing on a biologically significant group of butterflies, we have developed widely applicable methods to overcome these challenges, thereby enabling the scientific community to fully harness the research potential of this remarkable insect radiation. Furthermore, our results lend unique insight into the evolutionary history of butterfly diversification. Much attention is often paid to adult butterflies, and their wing patterns in particular. However, similar to all holometabolous insects, the butterfly life cycle is a two-stage process consisting of larva and adult. Perhaps surprisingly, our genomic analyses point repeatedly to the caterpillar stage as a major driver of natural selection with many of the genes that emerge from our analyses connecting to the larval stage through effects on larval morphology or host plant use. This enriches our view of insect evolution, and with these new genetic and genomic tools in hand we can now test the generality of these findings.

## Methods

### Butterfly husbandry and sample collection

Pupae of *P. xuthus* Linnaeus (*Px*) and *P. machaon* Linnaeus (*Pm*) were mainly purchased from butterfly insectariums in the suburbs of Ya’an, Sichuan, China. A proportion of pupae and adults were also acquired by raising the larvae collected by Xueyan Li and Guichun Liu in the suburb of Kunming, Yunnan, China. Pupae were reared under the conditions of 26 °C, 75% relative humidity and 18 h/6 h light/darkness. Emerged adults were crossed via hand pairing^48^,^49^. After mating, females were placed in net rooms with host plants for oviposition and then the mated adults were frozen at −80 °C for DNA extraction or kept as dried specimens. Eggs were collected to be used for experiments such as microinjection or allowed to develop on host plant at room temperature. *Px* larvae were fed with a horticulturally rutaceous plant (*Zanthxylum piperitum*) found by Xueyan Li, which appears to be a new host plant due to the species expansion and local adaptation. *Pm* larvae were fed with Umbelliferae plants fennel (*Foeniculum vulgare*) or carrot (*Daucus carota*).

To get high quality and high volume of DNA for library construction, head and thorax tissues (excluding wings) of six homogametric male (ZZ) adults of *P. xuthus* and *P. machaon* both from Sichuan, China, were dissected and used for DNA isolation using a Gentra Puregene Blood kit (Qiagen, Germany) following manual instructions. A single individual was used to construct short-insert libraries (150, 250 and 500 bp); each of other five individuals was used to construct long-insert libraries (2, 5, 10 and 20 kb) and 454-library, respectively. RNA samples from different development stages of both species including egg, larvae from first instar (L1) to fifth instar (L5), pupa (female and male) and adult (female and male) were collected for transcriptome sequencing. Owing to small body of egg and early development larvae, ten eggs, ten L1 and five L2 were used for constructing single RNA library; for other development stages, single individual was used to construct RNA library. For linkage mapping, *P. xuthus* pupae were obtained from a butterfly breeder, and one 3-day old male and one newly emerged female were used to set up a cross. The two parents (adults) and their 94 F1 offspring (second to fourth instar larvae) of *P. xuthus* were collected for DNA isolation and restriction site associated (RAD) sequencing. The eggs of *P. xuthus* for microinjection of Cas9 were collected in our greenhouse. The related protocols involved with butterfly in this study have been reviewed and approved by the internal review board of Kunming Institute of Zoology, Chinese Academy of Sciences.

### Genome sequencing and assembly

All short reads from short- and long-insert libraries for genome assembly were produced with the Illumina Hiseq2000 platform at BGI (Shenzhen, China) (Supplementary Note 1). Long reads were produced with the Roche 454FLXPlus Titanium platform at Duke University (USA) using the 454 Life Science/Roche protocol on the GS-FLX+ System. We carried out several rounds of assembly using high-quality data (Supplementary Note 1) from different sequencing platforms, that is, Illumina short reads, 454 long reads or both combined (Supplementary Table 1). Considering that there exist a larger number of short contigs in the assemblies only by Illumina short reads (Supplementary Table 1), we took a hybrid assembly strategy (Supplementary Fig. 5) of combining Illumina short reads with 454 long reads for the final assembly. First, about 30 × Illumina reads from short insert libraries were used to correct amplicon pyrosequencing errors in 454 reads. To do the correction, Coral 1.4 ( http://www.cs.helsinki.fi/u/lmsalmel/coral/)^50^ was used to correct sequencing errors by forming multiple alignments. We employed 454 model with options ‘−f −k 21 −e 0.07 −p 20’ during correction. For *P. xuthus* and *P. machaon*, 1.60% and 1.10% errors in 454 read bases were corrected, respectively. Second, corrected 454 reads were then fed into Newbler 2.6 (ref. 51) in DataAnalysis_2.6 toolkit with options ‘−mi 90 −ml 40 −nrm −het −m −cpu 20 −l 500’ to build contigs. Third, paired-end sequencing Illumina short reads were employed to combine contigs by SSPACE-1.2 basic^52^ ( http://www.baseclear.com/landingpages/sspacev12/). The reads from short insert size libraries (<2 kb) were first used to construct scaffolds (with parameters −a 0.6 −x 0 −g 1 −k 5). Next, reads from long insert size libraries (≧2 kb) were added in with parameters ‘−a 0.6 −x 0 −g 1 −k 3’. Finally, we used short paired-end (150, 250 and 500 bp libraries) information to retrieve gap-crossed read pairs with one end mapped to a unique contig and the other located in a gap, and performed a local assembly for these collected reads to fill the gaps by GapCloser v1.12 (ref. 53) ( http://soap.genomics.org.cn/soapdenovo.html). We further estimated genome size using flow cytometry (Supplementary Note 1) and then made haplotype separation and excluded microsporidia genome reads (Supplementary Note 1). Assembly quality was evaluated by three methods, that is, aligning short reads to assemblies, aligning transcripts to evaluate completeness of assemblies and evaluating completeness by CEGMA (Core Eukaryotic Genes Mapping Approach) (Supplementary Note 1).

### RAD-based linkage mapping of scaffolds

RAD library preparation was performed mainly according to Etter *et al.*^54^ with some modifications and sequencing was performed with an Illumina Hiseq 2000, and EcoRI site was used for RAD tag extraction (Supplementary Note 2). Linkage maps were constructed using both regression (Kosambi mapping function) and maximum likelihood algorithms (Supplementary Note 2). Syntenic relationship of chromosomal linkage between *P. xuthus* and *P. machaon* was analysed based on their orthologues (Supplementary Note 2).

### Genome annotation

Repetitive sequences and transposable elements in *P. xuthus*, *P.machaon* and two nymphalid butterflies (*D. plexippus* and *H. melpomene*) were annotated using a combination of homology to Repbase sequences, *de novo* prediction approaches and Tandem Repeats Finder (Supplementary Note 4). Protein-coding genes in *Papilio* genomes were predicted using homology-based methods, *ab initio* gene prediction and RNA-seq data (Supplementary Note 5). Gene function information, motifs and domains of their proteins were assigned by comparing with public databases including SwissProt, TrEMBL, KEGG, InterPro and Gene Ontology (Supplementary Note 5). To avoid biased comparisons of gene sets among butterfly species used in this study and two former studies (*D. plexippus* and *H. melpomene*), we conducted all these analyses for the four butterfly genomes.

### Gene evolution

To explore gene evolution patterns among butterflies, gene cluster was analysed including the genomes of five butterflies (*P. xuthus*, *P. machaon*, *D. plexippus*, *H. melpomene* and *M. cinxia*), moths (*B. mori* and *P. xylostella*), mosquito (*Anopheles gambiae*), fruit fly (*D. melanogaster*), beetle (*Tribolium castaneum*), bee (*Apis mellifera*), which cover all orders of holometabolous insects (Supplementary Note 5). One thousand and seventy-one single-copy genes were used for constructing phylogenetic tree of 11 species (Supplementary Note 5). Gene orthologous relationship among Lepidopteran insects (*P. xuthus*, *P. machaon*, *D. plexippus*, *H. melpomene* and *B. mori*) was determined (Supplementary Note 5) and positively selected genes were identified in *P. xuthus*, *P. machaon* and both of them (Supplementary Note 5).

### Transcriptome sequencing and analysis

Transcriptome sequencing for different developmental stages was performed with Illumina RNA-seq protocols and two methods, that is, *de novo* assembly of clean RNA reads and mapping them back to the assembled genomes, were carried out for transcriptome assembly (Supplementary Note 6). RPKM (reads per kb per million reads) was used to measure gene expression abundance. We analysed gene expression dynamics in development (Supplementary Note 6), differentially expressed genes (Supplementary Note 6), expression patterns of positively selected genes and lineage-specific genes (Supplementary Note 6).

### Specific gene families and pathways

To explore possible significance of expansion of farnesyl pyrophosphate synthase (*FPPS*) gene family in *Papilio* butterflies, we investigated all genes encoding scIPPS and other enzymes in the pathways of JH biosynthesis and degradation, as well as protein prenyltransferases in ten insect species (*P. xuthus*, *P. machaon*, *D. plexippus*, *H. melpomene*, *B. mori*, *P. xylostella*, *A. gambiae*, *D. melanogaster*, *T. castaneum* and *A. mellifera*), constructed their gene trees and analysed their expression patterns (Supplementary Note 7). We also identified gene families involved in detoxification of various xenobiotics, including cytochrome P450s, glutathione *S*-transferase and carboxylesterases (Supplementary Notes 8 and 9). In addition, we also analysed the genes related to body colour (Supplementary Note 10) and PCP genes (Supplementary Note 11) playing a fundamental role in morphogenesis of vertebrates and invertebrates. All phylogenetic trees were constructed using Maximum likelihood in PAML^55^.

### Analysis of genomic divergence

The divergence regions were analysed based on the assembled genomes of *P. xuthus*, *P. machaon* and Illumina genome resequencing data of one individual of a third *Papilio* species (*P. polytes*) with its data available. We conducted pairwise local alignments using the *P. xuthus* genome as a reference and aligned paired-end reads of one male *P. polytes* individual and simulated paired-end reads of the *P. machaon* genome. Next, we calculated fixed different single-nucleotide polymorphisms per bp for each 50-kb window across the reference genome for three comparisons (*P. xuthus*/*P. machaon*, *P. xuthus*/*P. polytes* and *P. machaon*/*P. polytes*) and the highly divergent regions were characterized using 95% smoothed empirical likelihood quantiles among all three comparisons as a cutoff (Supplementary Note 12).

### Genome editing in butterfly using CRISPR/Cas9 system

We designed sgRNA target sites by seeking sequences corresponding to N_20_NGG on exon regions of the sense or antisense strand of the DNA by ZiFit Targeter programme^56^. Next, we BLAST (using Basic Local Alignment Search Tool) these candidate target sequence against the *P. xuthus* genome to eliminate those with off-target sites using strict criteria, where the candidate-editable site is defined only when the seed region (12 nucleotides (nt) to protospacer adjacent motif NGG) is unique^15^. From candidate-editable sites, we selected those with the first two bases of GG, GA or AG for sgRNA synthesis. sgRNA can be synthesized by plasmid-based or PCR-based strategies and we used a PCR-based method to synthesize sgRNAs. Briefly, a unique oligonucleotide encoding T7 polymerase-binding site and the sgRNA target sequence N_20_ was designed as forward primer (CRISPRF 5′-GAAATTAATACGACTCACTATAN_20_GTTTTAGAGCTAGAAATAGC-3′) and a common oligonucleotide encoding the remaining sgRNA sequence (sgRNAR 5′-AAAAGCACCGACTCGGTGCCACTTTTTCAAGTTGATAACGGACTAGCCT TATTTTAACTTGCTATTTCTAGCTCTAAAAC-3′) was designed as a reverse primer^18^. All the oligonucleotides (CRISPRF and sgRNAR) were synthesized by GENEray Company (Shanghai, China). The information of target sites and the primers for analysing target sites of three genes (*Px_03961_Abd-B*, *Px_01073_e* and *Px_15230_fz*) are shown in Supplementary Table 44 and Supplementary Fig. 39. PCR of primer-self amplification was performed with Q5 high-quality DNA polymerase (BioLabs) in 100 μl reaction volumes. Three hundred nanograms of purified PCR product was used as DNA template to perform *in-vitro* transcription with the MAXIscript T7 kit (Life Technology, USA) for 4 h at 37 °C. The Cas9 mRNA was transcribed using NotI-digested Cas9 expression vector pTD1-T7-Cas9 (ref. 19) and the mMESSAGE mMACHINE T7 ULTRA kit (Life Technologies). After the transcription reaction, the poly (A) tailing reaction and *DNase I* treatment were performed according to the manufacturer’s instructions. Both the sgRNA and the Cas9-encoding mRNA were then purified by LiCl precipitation and redissolved in RNase-free water.

Fresh eggs were collected from the host plant leaves, dipped into clear water and then aligned on the microscope slide with a soft paintbrush and fixed with glue. Two-nanolitre mix of sgRNA(s) and Cas9-encoding mRNA, at varying concentrations (Supplementary Tables 45, 47 and 49), were injected through the chorion into each egg under a dissecting microscope (Nikon SMZ800), using a TransferMan NK2 and FemtoJet microinjection system (Eppendorf, Germany). After injection, eggs were put in a petri dish and then placed in an incubator at 25 °C and 70% relative humidity. When embryos hatched, host plant leaves were placed into the dishes for newly hatched larva feeding. The leaves with larvae were carefully transferred into large dishes. The phenotype of G_0_ generation larvae was carefully checked using a dissecting microscope and photographed using a digital camera. Pupae were transferred into plastic baskets to eclose. We observed morphologic changes in different developmental stages, using a microscope and by the eye. For *Abd-B*, we mainly observed variation of the larval abdomen; for *ebony*, we primarily noted colour variation of larvae and adults. For *fz*, we mainly observed cuticular structures.

For *Abd-B* mutants of *P. xuthus*, genomic DNA extraction for the whole body of unhatched G_0_ larvae dissected from developed eggs and subsequent PCR were carried out using an Animal Tissue Direct PCR kit (Foregene, China), following manual instruction, and genomic DNA of whole body of G_0_-hatched larvae were extracted using Gentra Puregene Blood Kit (Qiagen), and exTaq polymerase was used in PCR amplification. The primer pairs included F3/R6, F9/R11, and F10/R11 (Supplementary Fig. 39a). For *ebony* mutants, whole body of larvae, prepupae and adult were used to extract genomic DNA, using Gentra Puregene Blood Kit (Qiagen), and exTaq polymerase was used in PCR amplification. The primer pairs were F2/R2 for amplifying target sites of T2-T303 (*Px_01073_e*-I and *Px_01073_e*-II) or F7/R4 for amplifying T454-T6 (*Px_01073_e*-III) (Supplementary Fig. 39b). For *fz* mutants, whole body of larvae were used to extract genomic DNA, using Gentra Puregene Blood Kit (Qiagen) and exTaq polymerase was used in PCR amplification. The primer pair for amplifying target sites is F2/R2 (Supplementary Fig. 39c). PCR products of target sites were detected with T7 endonuclease I (T7EI) as previously described^57^. T7EI-positive and morphologically mutated individuals were further confirmed by Sanger sequencing 12 TA clones. We also performed qRT–PCR and western blot analysis to check the effect of disruption of *Abd-B* gene on its expression at RNA or protein levels (Supplementary Note 13). In addition, we also performed qRT–PCR to check the effect of *ebony* gene on its expression at RNA level (Supplementary Note 13).

For those targets resulting in morphological mutants, to exclude the possibility that these morphological mutants were induced by off-target cleavage events during the genome-editing process, we further analysed possible off-target sites in the genome using three methods CasOT^43^, Cas-OFFinder^44^ and COSMID^45^ with the least stringent parameters and carried out whole-genome next-generation sequencing validation for the *ebony* gene mutant (Supplementary Note 13). The resequencing reads of mutant have been uploaded to the NCBI Sequence Read Archive and are available via the accession number SRA272356.

## Additional information

**How to cite this article:** Li, X. *et al.* Outbred genome sequencing and CRISPR/Cas9 gene editing in butterflies. *Nat. Commun.* 6:8212 doi: 10.1038/ncomms9212 (2015).

## Supplementary Material

Supplementary Information Supplementary Figures 1-56, Supplementary Tables 1-52, Supplementary Notes 1-13 and Supplementary References

## Figures and Tables

**Table 1:** Comparison of reference genomes among Lepidopterans.

	***Pm***	***Px***	***Hm***	***Dp***	***Bm***	***PLX***
Chromosome number (*n*)	31*	30*	21†	29–30‡	28§	31||
Genome size (Mb) based on the bases in contigs and in scaffolds	265/281	231/244	274/269†	242‡/249¶	432/481§	385/394||
Genome size (Mb) measured by *C*-value	234∼256	218∼238	300#	290#	520#	NA
N50 of contig (kb)/scaffold (Mb)	81/1.15	492/3.4	51/0.2†	51‡/0.7¶	13/3.7§	49/0.72||
TE (% genome)	21.10	20.26	28.47†	12.21‡	21.1§	33.97||
Number of protein-coding genes	15,499	15,322	15,984**	16,866‡	14,623§	18,071||

*Bm*, *B. mori*; *Dp*, *D. plexippus*; *Hm*, *H. melpomene*; *Plx*, *P. xylostella; Pm*, *P. machaon*; *Px*, *P. xuthus*.

^*^From Maeki^59^.

^†^From Dasmahapatra *et al.*^8^

^‡^From Zhan *et al.*^7^

^§^From Xia *et al.*^60^

^||^From You *et al.*^13^

^¶^From Zhan *et al.*^61^

^#^From Gregory *et al.*^62^

^**^Predicted in this study using the same pipeline as in *Pm* and *Px*.

**Table 2:** Mutagenesis efficiency of knocking out genes *Abdominal-B*, *ebony* and *frizzled* in *P. xuthus.*

**Gene**	**Target sites** *	**RNA con. (ng** **μl**^−1^)†	**Phynotype rate (%)** ‡	**Mutation rate (%)**	**Mutation type**
*Px_03961_Abd-B*	T42	200; 300	7.95 (7/88)	35.38	D: 3–51; I: 4–18
		600; 600	8.26 (10/121)	25	D: 3–66; I: 4–5; M: 40
	T42/T95	50/50; 100	6 (6/100)	18.33	D: 5–59; I: 3–19
		566/416; 1,200	91.79 (123/134)	90.85	D: 3–73; I: 3–64; M: 3–29
*Px_01073_e*	T2/T303	158/159; 1,200	31.25 (5/16)	66.67	D: 5–33; I: 3–10; M: 3
	T454/T6	200/150; 1200	88 (22/25)	29.92	D: 6–62; I: 3–15; M: 3–8
*Px_15230_fz*	T268/T283	500/500; 1,,000	4.12 (4/96)	45.16	D: 3–64; I: 3–15; M: 4–5

D, delete fragment (bp); I, insert fragment (bp); M, mutated fragment (bp); sgRNA, single guide RNA.

^*^‘/’ denotes injection of mixed targets.

^†^The number before and after semicolon denotes injected concentration of sgRNA and Cas9 mRNA, respectively.

^‡^The number in the bracket denotes mutant/observed injected individuals.
