# Supplementary material for: Outbred genome sequencing and CRISPR/Cas9 gene editing in butterflies
Source: Nat Commun. 2015 Sep 10;6:8212. doi: 10.1038/ncomms9212 (PMC4568561; doi:10.1038/ncomms9212)
Supplement: Supplementary Information — Supplementary Figures 1-56, Supplementary Tables 1-52, Supplementary Notes 1-13 and Supplementary References [file ncomms9212-s1.pdf]

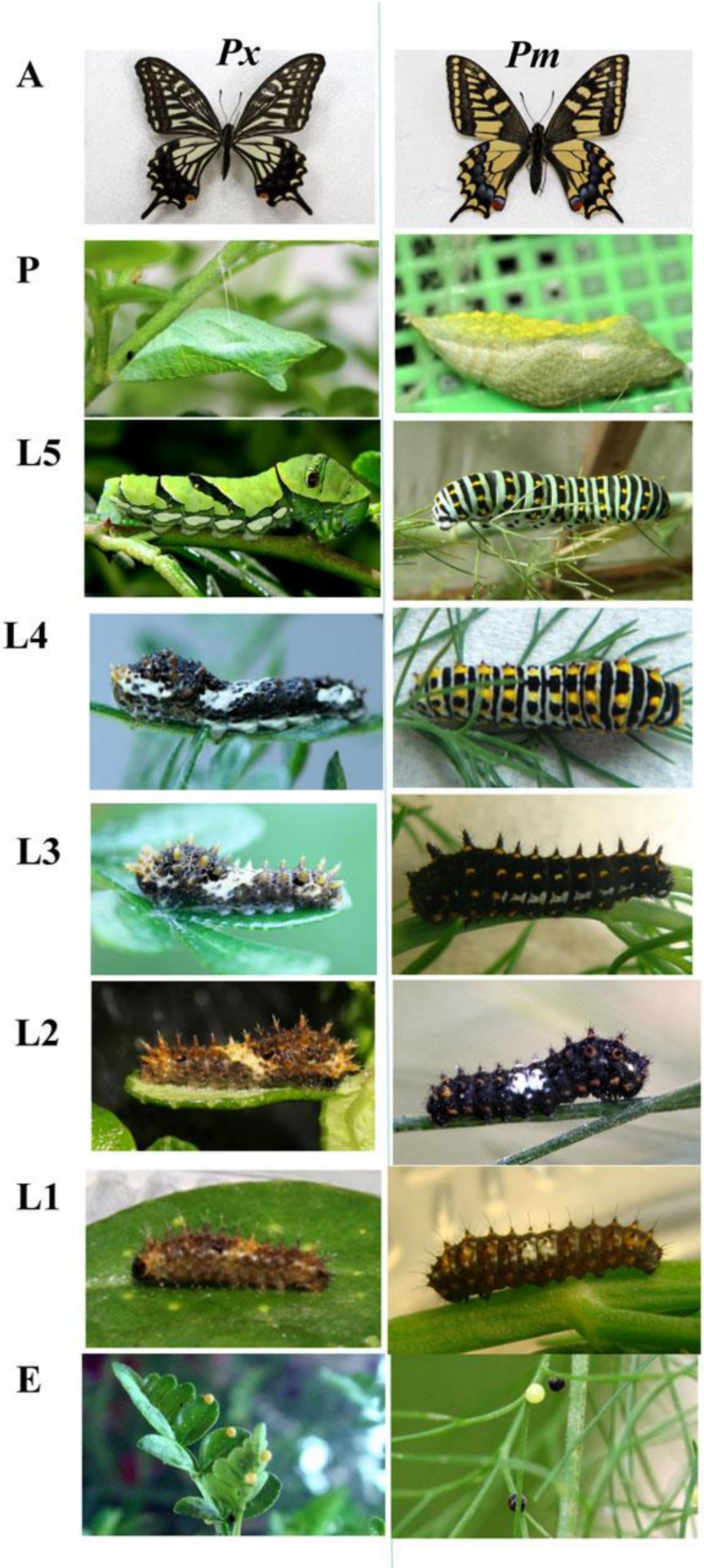

Supplementary Figure 1. Comparisons of different developmental stages between *Papilio xuthus* (*Px*) and *P. machaon* (*Pm*). A: adult; P: pupa; L1, 2, 3, 4, 5: Larva of the 1st, 2nd, 3rd, 4th and 5th instar; E: egg. Photos by the authors Guichun Liu and Xueyan Li.

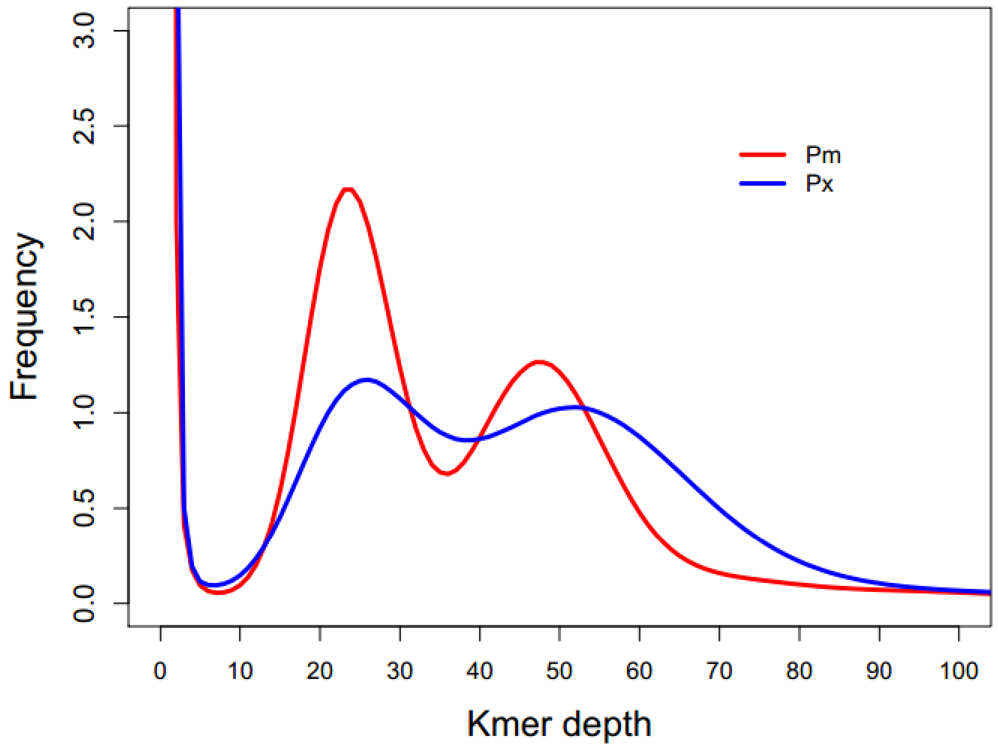

Supplementary Figure 2. *K*-mer frequency distribution curves based on Illumina short reads from 250 bp and 500 bp libraries for *P. xuthus* (*Px*) and *P. machaon* (*Pm*). The bimodality of the curves and the height of the first peak that is higher than the second peak's indicate that the genomes have obvious signature of heterozygosity.

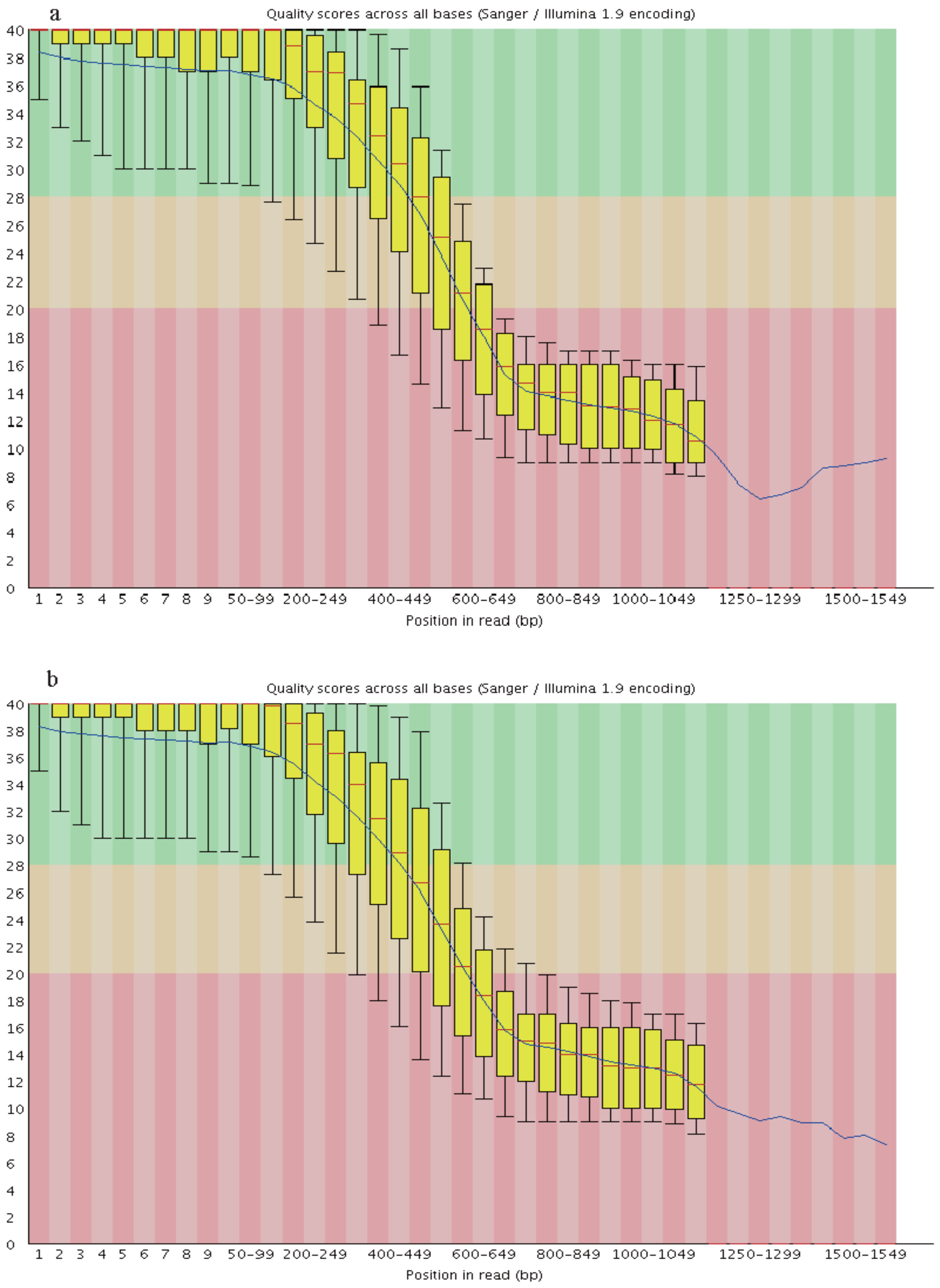

Supplementary Figure 3. The quality distribution of 454 long reads by position for *P. xuthus* (*Px*) and *P. machaon* (*Pm*) evaluated by FastQC. a, *Px*; **b**, *Pm*. **x**-axis: position of reads (bp); y-axis: Phred quality score (Q score). The box plot is used to display the distribution of base phred quality score based on the five number summary: minimum, first quartile, median, third quartile, and maximum.

a

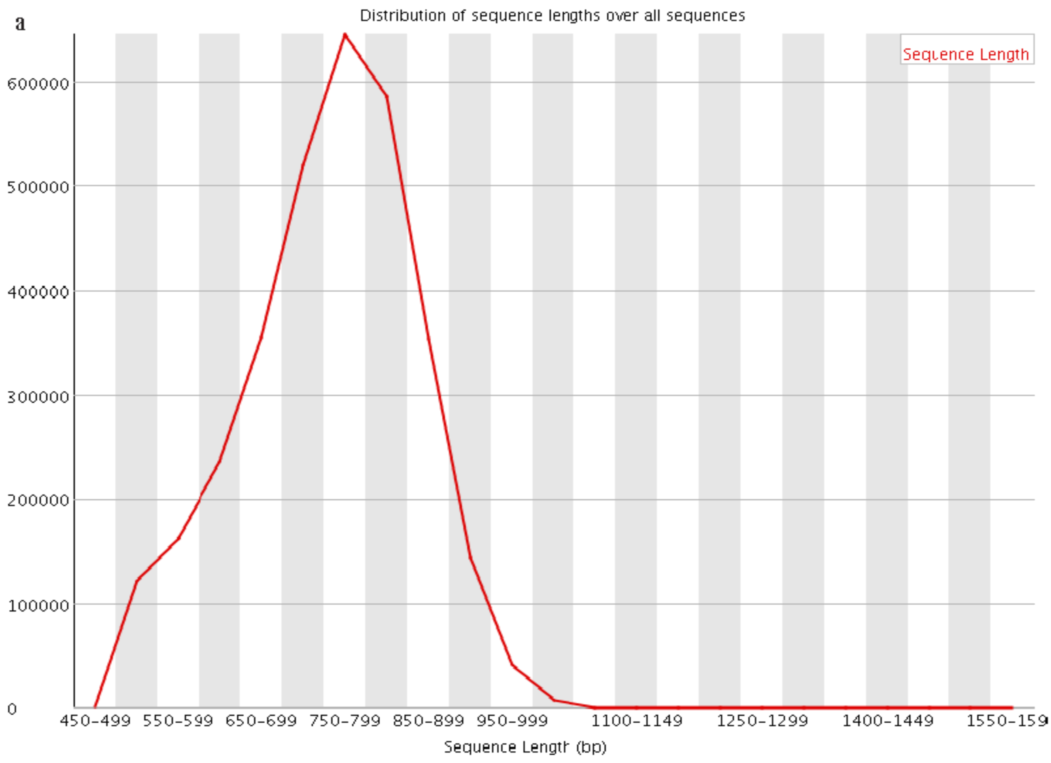

b

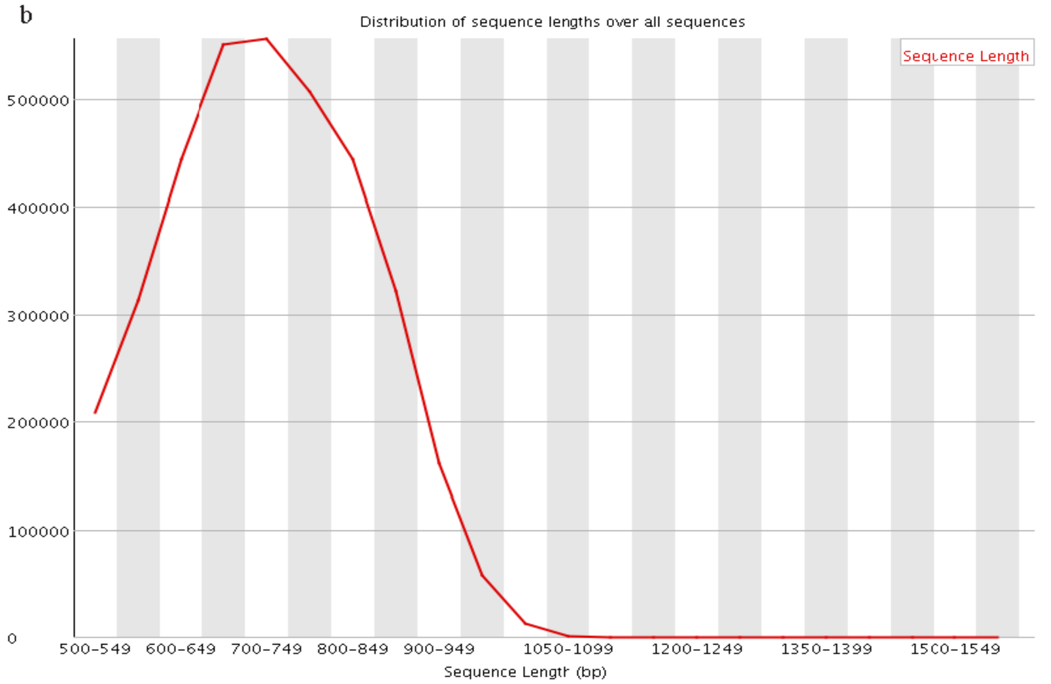

Supplementary Figure 4. The length distribution of 454 long reads for *P. xuthus* (*Px*) and *P. machaon* (*Pm*) evaluated with FASTOC. a, *Px*; **b**, *Pm***.** x-axis: sequence length (bp); y-axis: reads number.

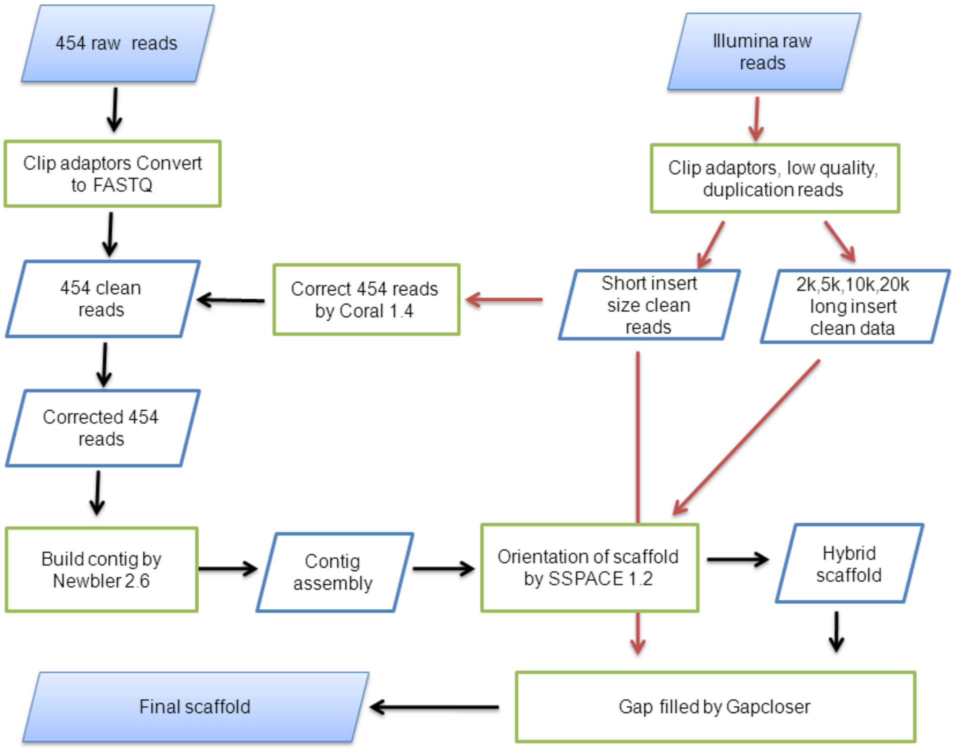

Supplementary Figure 5. Flowchart of hybrid assembly strategy using 454 long reads and Illumina short reads. Parallelograms denote input/output data; rectangles denote methods; red arrows indicate steps of Illumina data analysis and black arrows mainly relied on 454 long data.

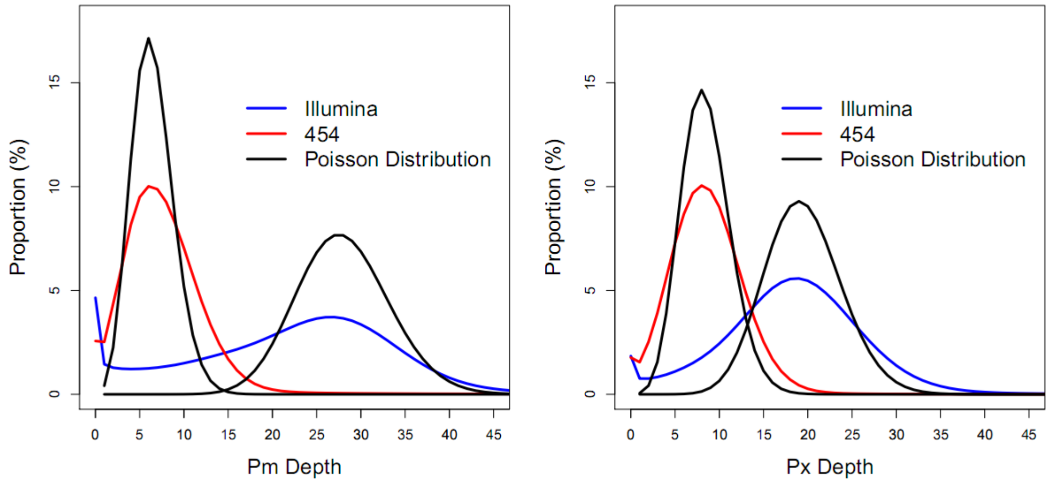

Supplementary Figure 6. The distribution of single base sequencing depth according to reads alignment in *P. xuthus* (*Px*) and *P. machaon* (*Pm*). Distribution of single base depth in *Pm* and *Px* calculated based on Ilumina and 454 reads alignment. The theoretical Possion distribution is also plotted for comparison.

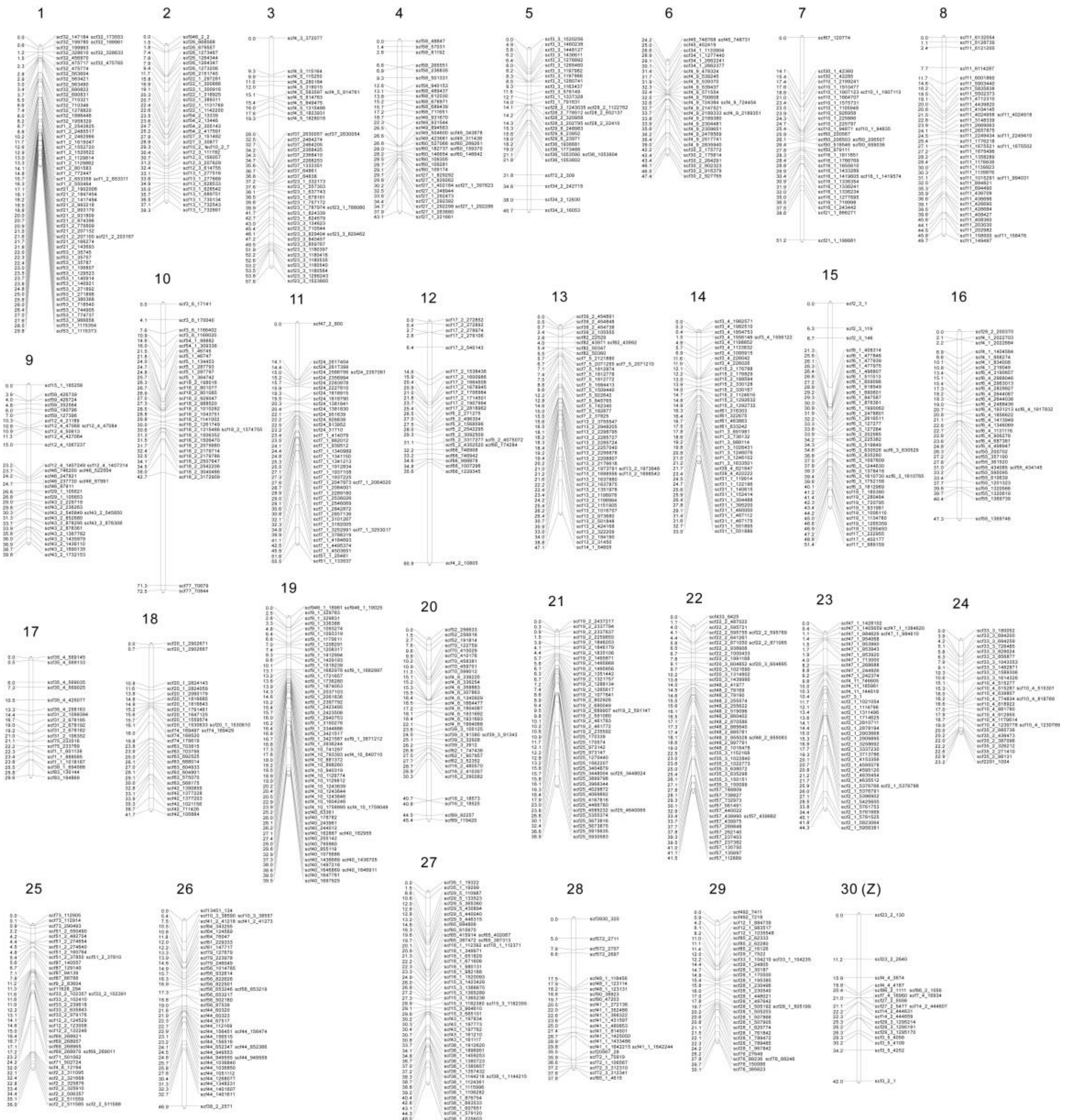

Supplementary Figure 7. RAD linkage map of *P. xuthus* (*Px*). Positions in cM are shown on the left of the linkage group. The linkage mapping markers and their targeted scaffolds are shown on the right.

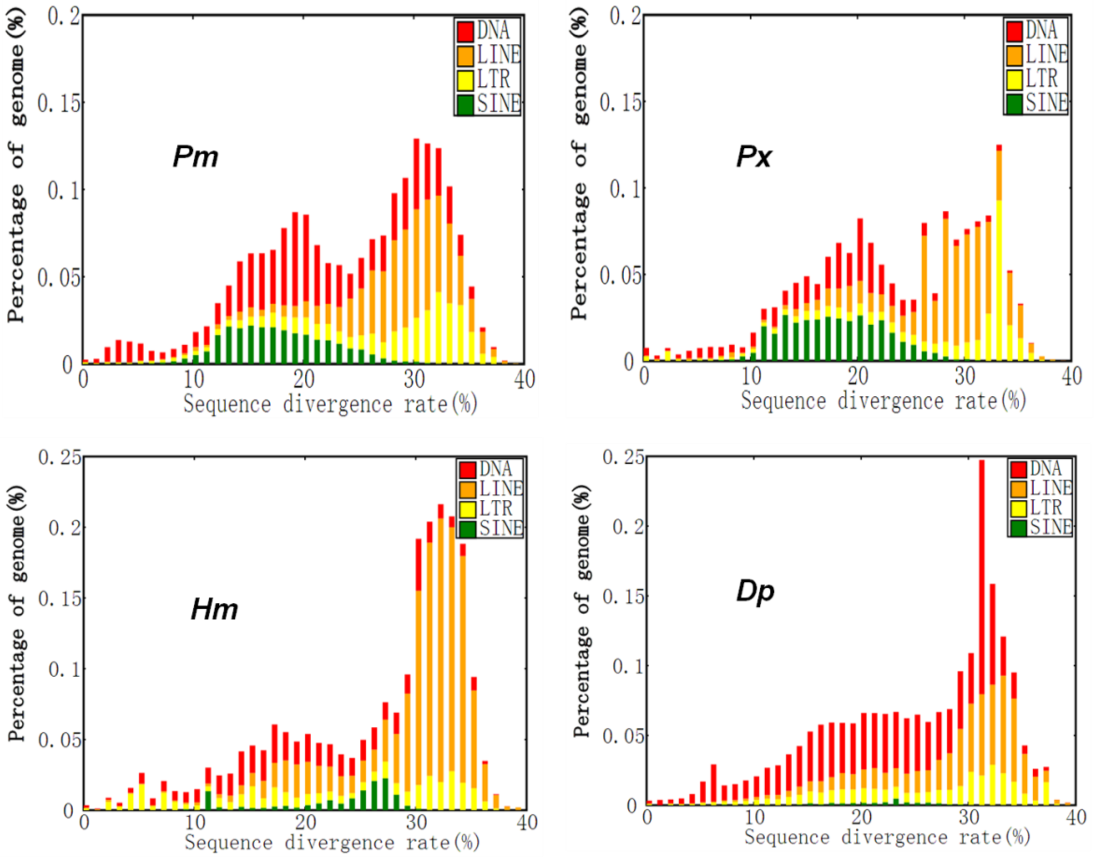

Supplementary Figure 8. The distribution of sequence divergence of classified transposable element (TE) families in four butterflies. *Px* (*P. xuthus*), *Pm* (*P. machaon*), *Hm* (*Heliconius melpomene*), *Dp* (*Danaus plexippus*).

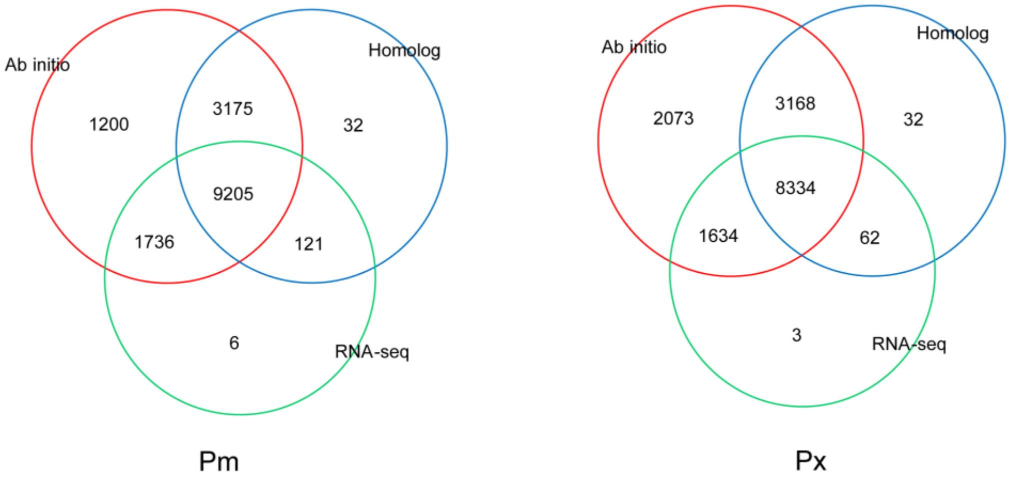

Supplementary Figure 9. GLEANgene sets predicted via different methods. *Ab initio*: red circle; homology-based: blue circle; RNA-seq: green circle. The overlapping gene number among different methods with the overlapping gene region is greater than 50% for *P. xuthus* (*Px*) and *P. machaon* (*Pm*).

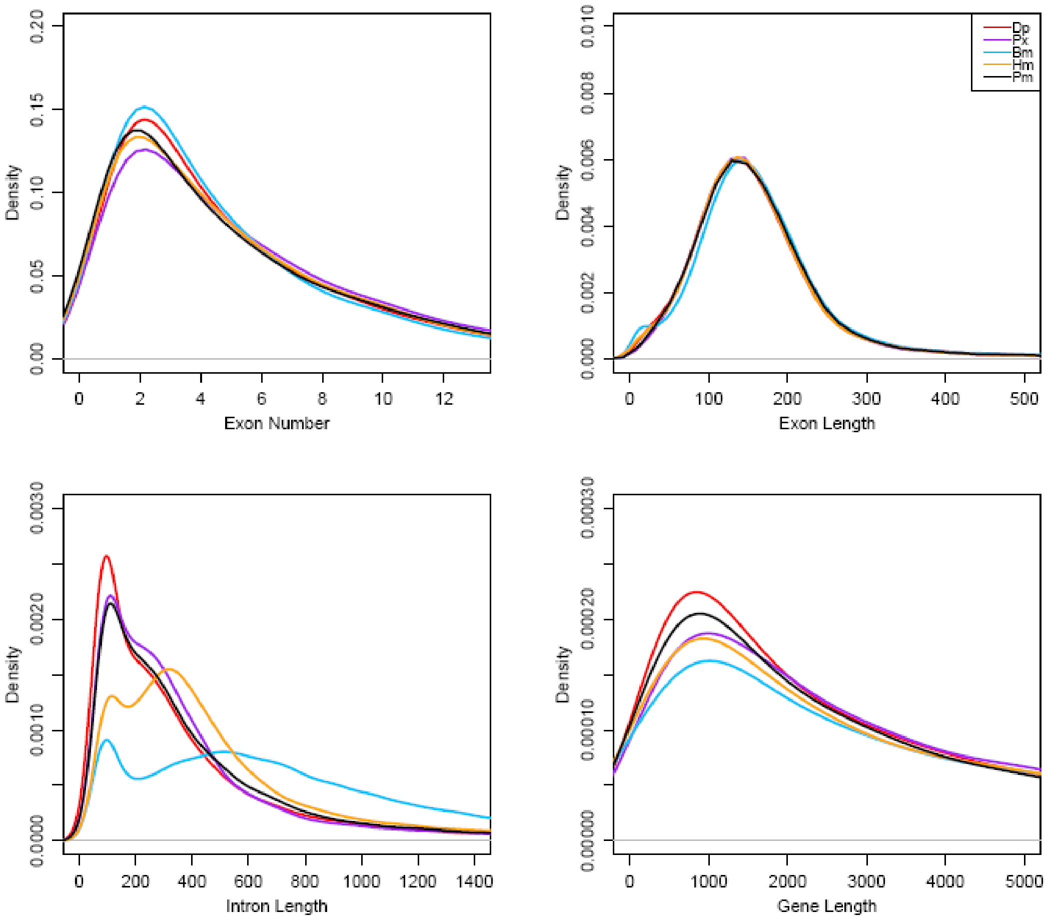

Supplementary Figure 10. Gene features (exon number, exon length, intron length, and gene length) in four butterflies and silkworm. *Px* (*P. xuthus*), *Pm* (*P. machaon*), *Hm* (*H. melpomene*), *Dp* (*D. plexippus*), *Bm* (*Bombyx mori*).

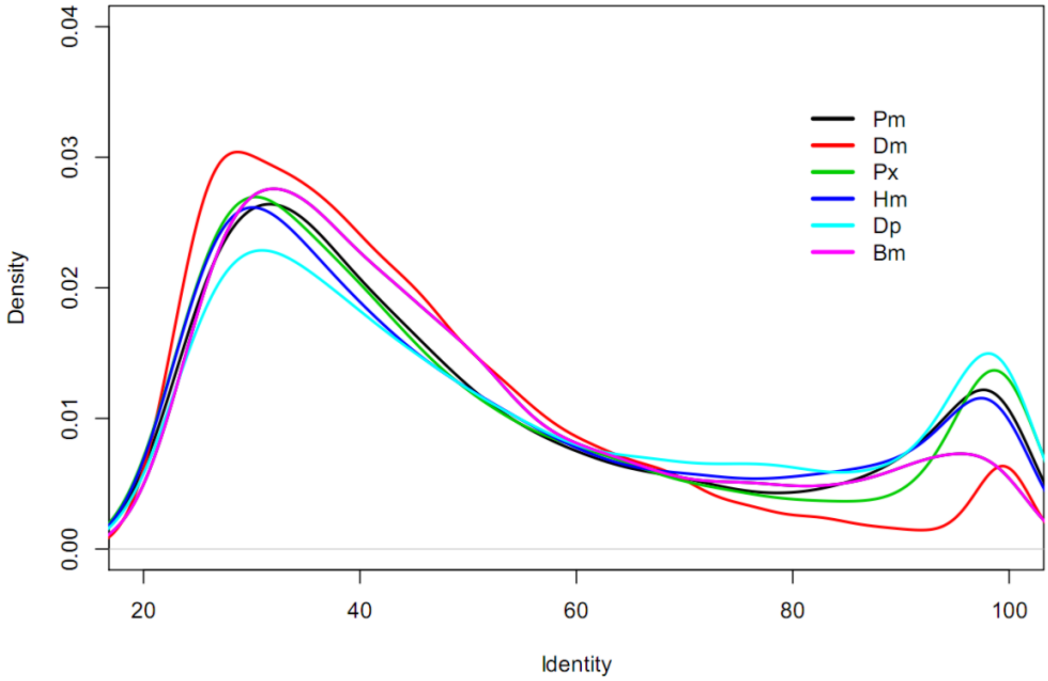

Supplementary Figure 11. Properties of reciprocal besthit gene pairs by intraspecies all-vs-all gene alignment in the four butterflies, silkworm, and fruitfly. Proteins of each species were aligned to itself and the best hit result was defined as selfalign-besthit pair genes. *Px* (*P. xuthus*), *Pm* (*P. machaon*), *Hm* (*H. melpomene*), *Dp* (*D. plexippus*), *Bm* (*B. mori*), *Dm* (*Drosophila melanogaster*).

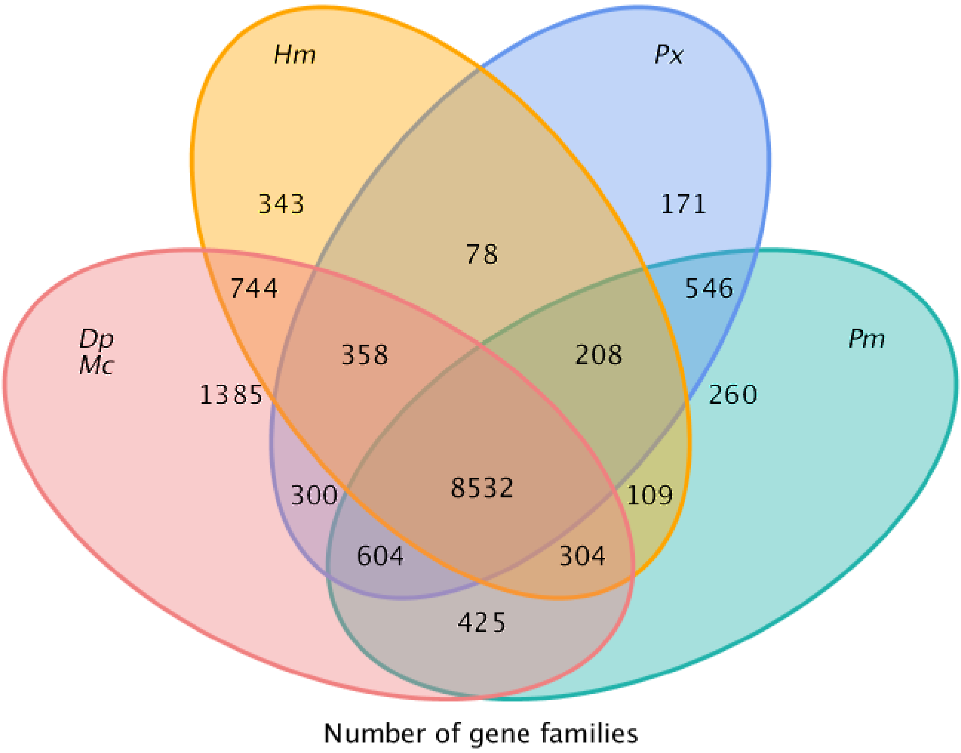

Supplementary Figure 12. Venn diagrams showing unique and overlapping protein family numbers among the four butterflies. *Px* (*P. xuthus*), *Pm* (*P. machaon*), *Hm* (*H. melpomene*), *Dp* (*D. plexippus*), *Mc* (*Melitaea cinxia*).

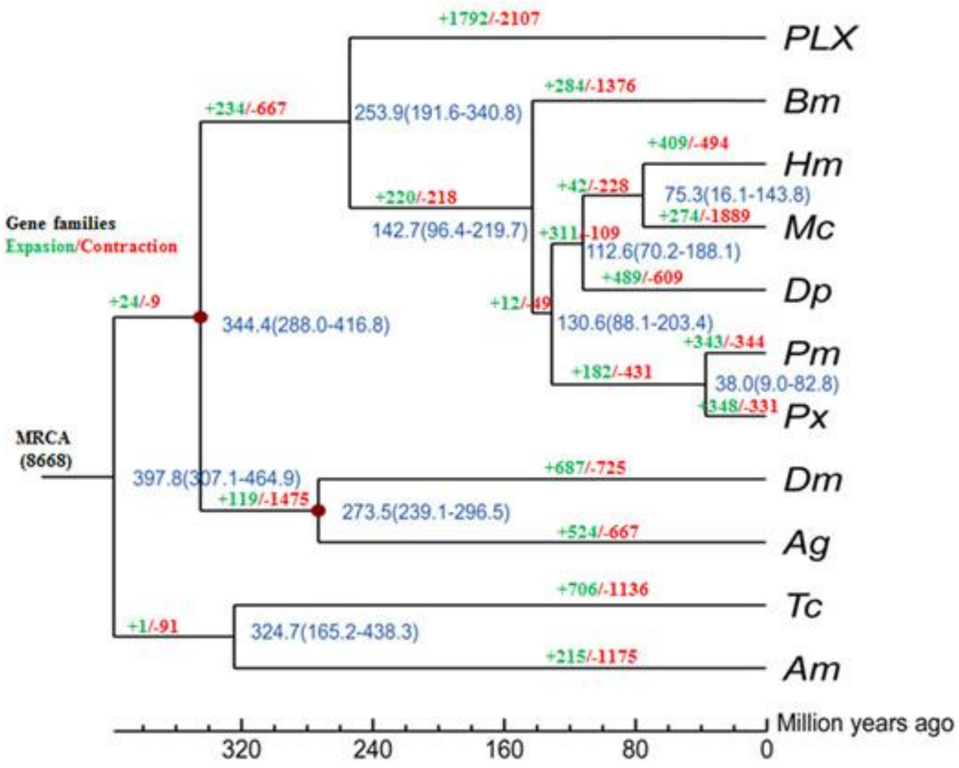

Supplementary Figure 13. Phylogenetic relationship, divergence time as well as expansion and contraction of gene families among 11 insects. Including five butterflies: *Px* (*P. xuthus*), *Pm* (*P. machaon*), *Hm* (*H. melpomene*), *Dp* (*D. plexippus*), *Mc* (*M. cinxia*); 2 moths: *Bm* (*B. mori*), *PLX* (*Plutella xylostella*); 1 fruitfly: *Dm* (*D. melanogaster*): 1 mosquito: *Ag* (*Anopheles gambiae*); 1 beetle: *Tc* (*Tribolium castaneum*); 1 bee: *Am* (*Apis mellifera*). The numbers (blue) marked in each node represent divergence times (million years ago) estimated with two fossil records as calibration times and with the 95% highest posterior density (HPD) credibility intervals in brackets. The numbers on each branch represent the family number gained (green) or lost (red) in this branch.

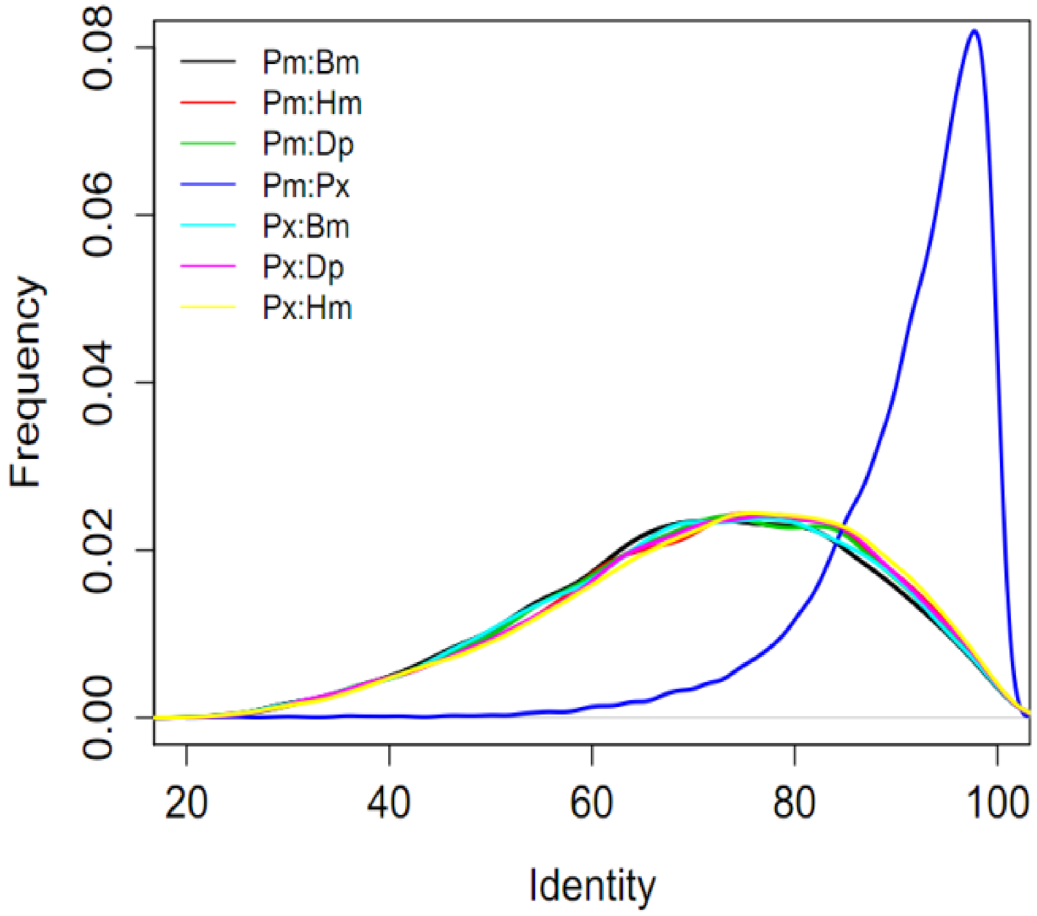

Supplementary Figure 14. Properties of orthologous genes of *Pm*/*Px* proteins in comparison with other butterflies and silkworm. The otholog identity between *Pm* and *Px* is significantly higher than those of other species pairs. And the identity between *Papilionidae* and *Nymphalidae* is also slightly higher than that between *Papilionidae* and *Bm*. *Px* (*P. xuthus*), *Pm* (*P. machaon*), *Hm* (*H. melpomene*), *Dp* (*D. plexippus*), *Bm* (*B. mori*).

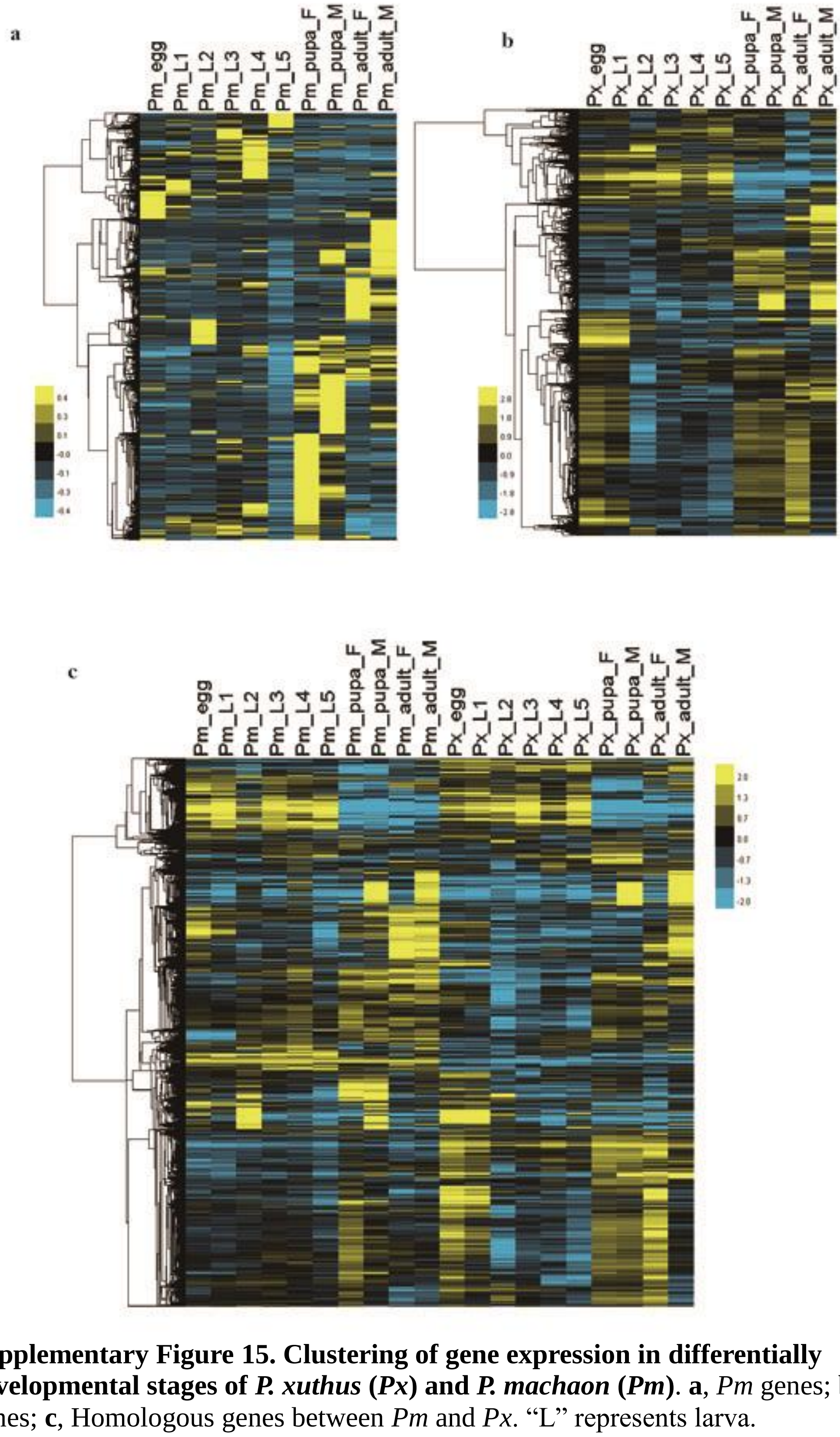

Supplementary Figure 15. Clustering of gene expression in differentially developmental stages of *P. xuthus* (*Px*) and *P. machaon* (*Pm*). **a**, *Pm* genes; **b**, *Px* genes; **c**, Homologous genes between *Pm* and *Px*. “L” represents larva.

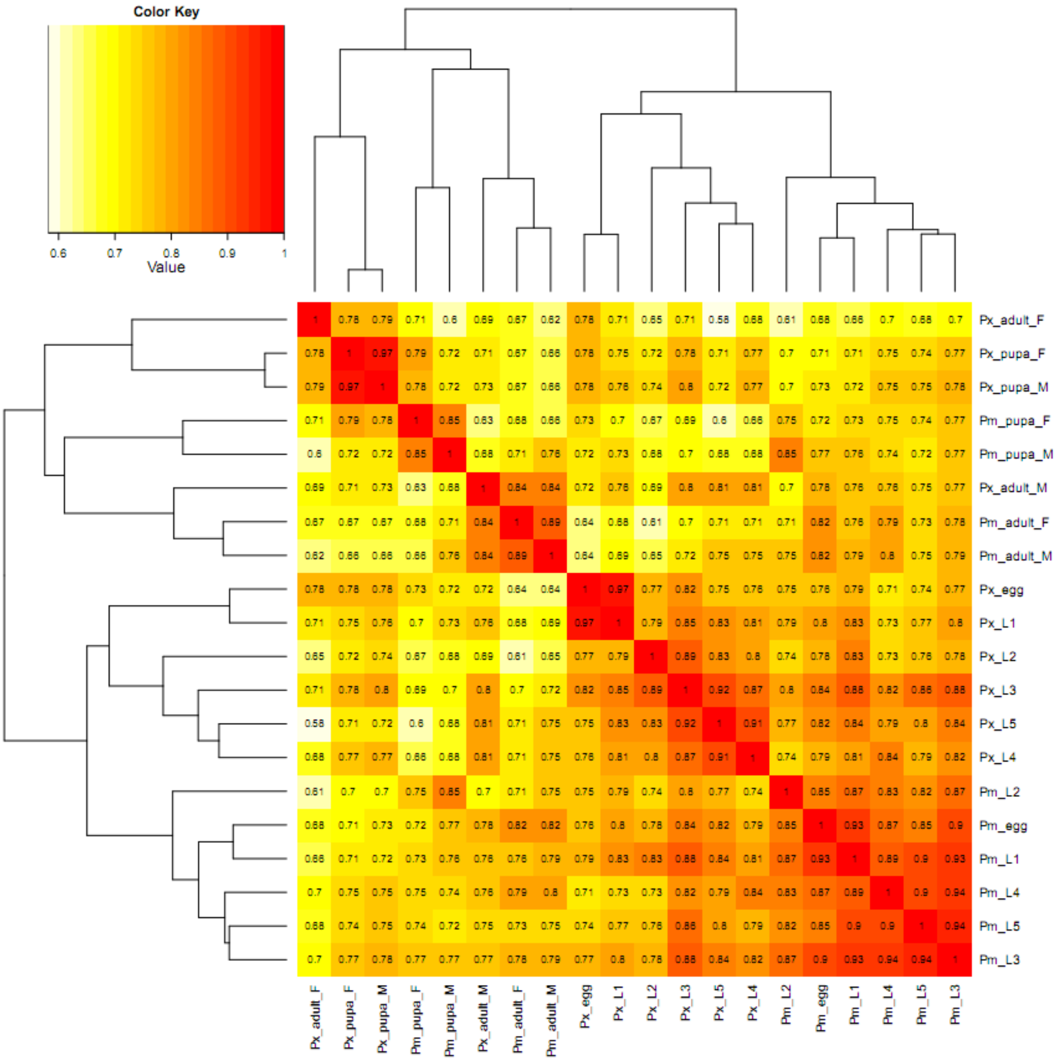

Supplementary Figure 16. Gene expression correlations of developmental stages between *P. xuthus* (*Px*) and *P. machaon* (*Pm*). The numbers in the cells present Pearson correlation in each of the two stages.

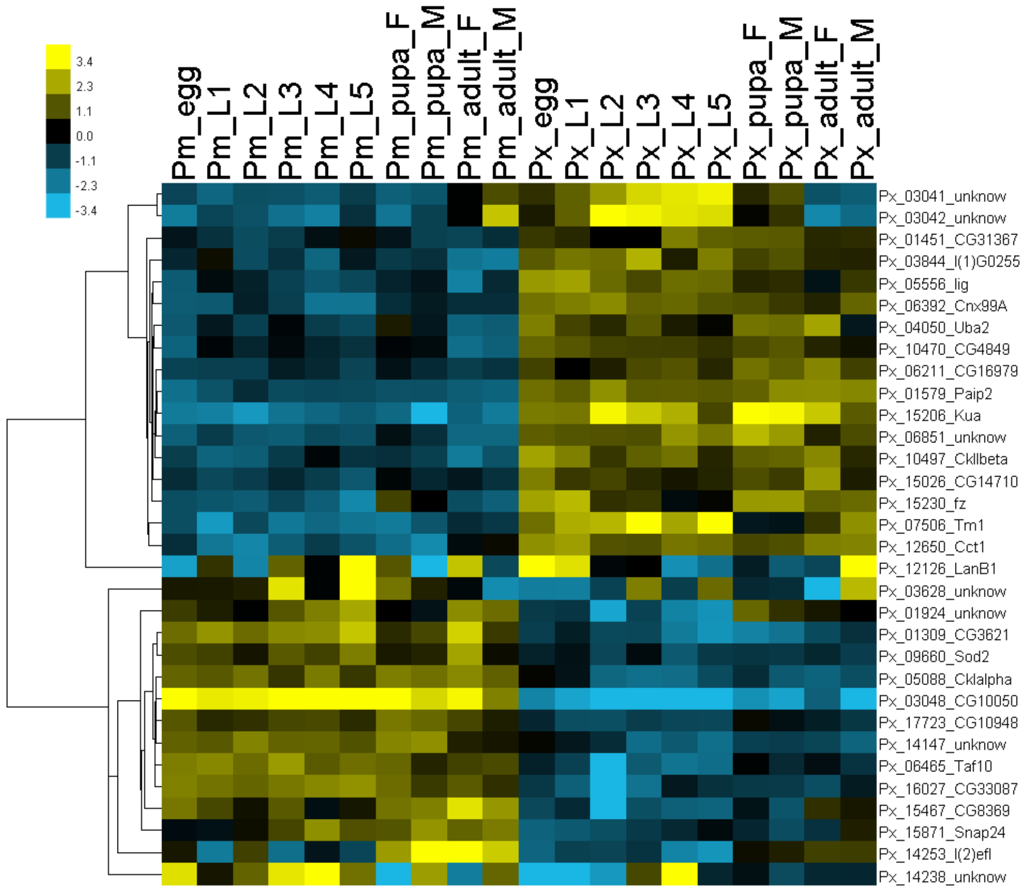

Supplementary Figure 17. Heatmap of the 32 orthologous pairs with differential expression in all the stages between *P. xuthus* (*Px*) and *P. machaon* (*Pm*).

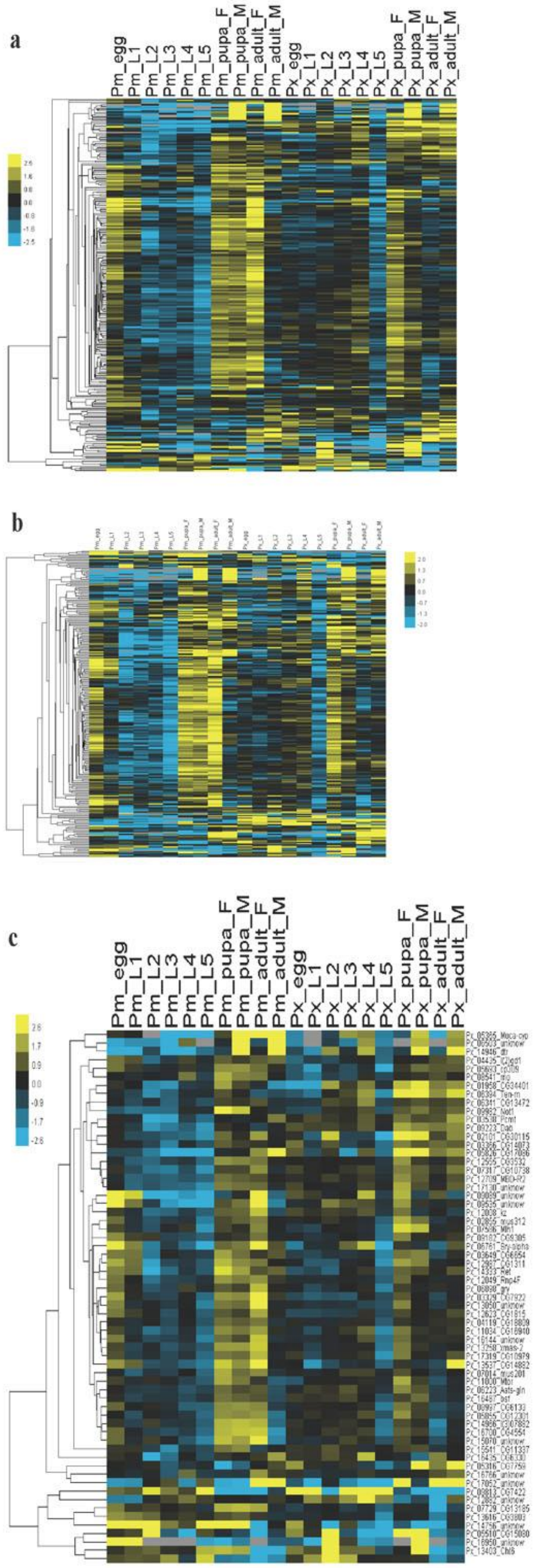

Supplementary Figure 18. Expression heat maps of positively selected genes in *P. xuthus* (*Px*) and *P. machaon* (*Pm*). **a**, *Px* selected gene and their orthologs; **b**, *Pm* selected genes and orthologs; **c**, positively selected genes in both *Pm* and *Px*.

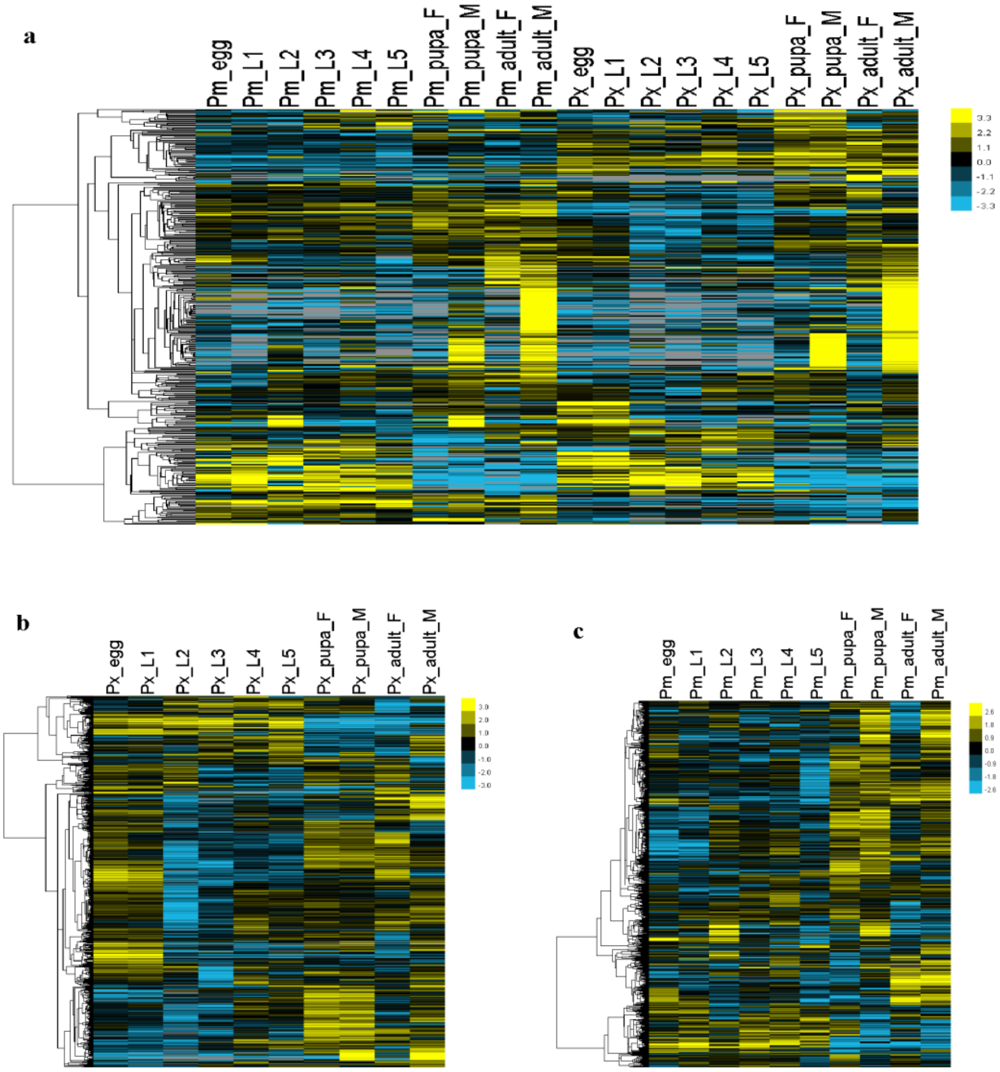

Supplementary Figure 19. Developmental expression pattern of lineage specific genes in *P. xuthus* (*Px*) and *P. machaon* (*Pm*). **a**, *Papilio* specific genes; **b**, *Px* orphan genes**;** **c**, *Pm* orphan genes**.**

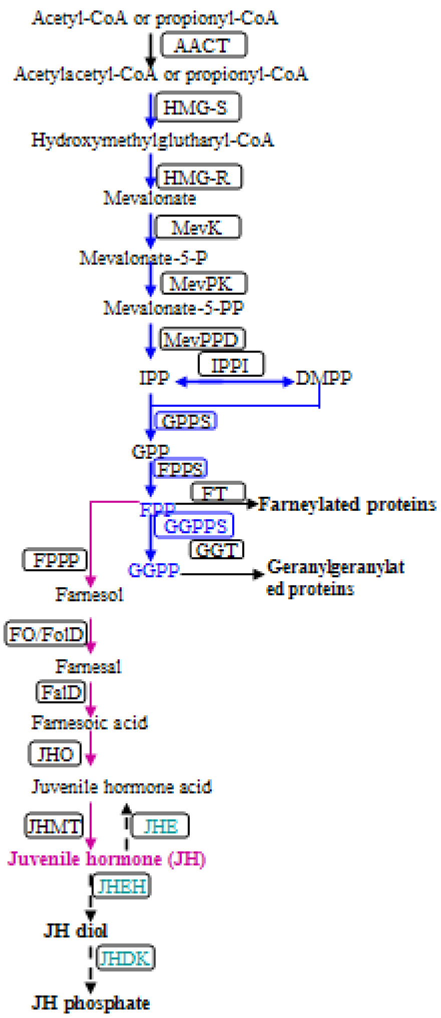

Supplementary Figure 20. The pathway of short-chain isoprenyl diphosphate (scIPP) biosynthesis (blue arrows**), juvenile hormone (JH) biosynthesis (*purple arrows*) and degradation.** After Belles et al.[1], Kinjoh et al.[2], Vandermoten et al.[3]. The abbreviations in square column represent enzymes of each step. Abbreviations: Acetoacetyl-CoA thiolase (AACT), 3-Hydroxy-3-methylglutaryl-CoA synthase (HMGS), 3-Hydroxy-3-methylglutaryl-CoA reductase (HMGR), Mevalonate kinase (MevK), Phosphomevalonate kinase (MevPK), Diphosphomevalonate decarboxylase (MevPPD), Isopentenyl diphosphate isomerase (IPPI), Geranyl diphosphate (GPP) synthase (GPPS), Farnesyl diphosphate (FPP) synthase (FPPS), Geranylgeranyl diphosphate (GGPP) synthase (GGPPS), Farnesyl diphosphate pyrophosphatase (FPPP), Farnesol oxidase (FO)/Farmesol dehydrogenase (FolD), Farnesal dehyrogenase (FalD), JH epoxidase (JHO), JH methyl transferase (JHMT), JH esterase (JHE), JH epoxide hydrolase (JHEH), JH diol kinase (JHDK), farnesyl transferase (FT), geranylgeranyl transferase (GGT). Solid arrows represent biosynthesis, and dotted arrows represent degradation.

a

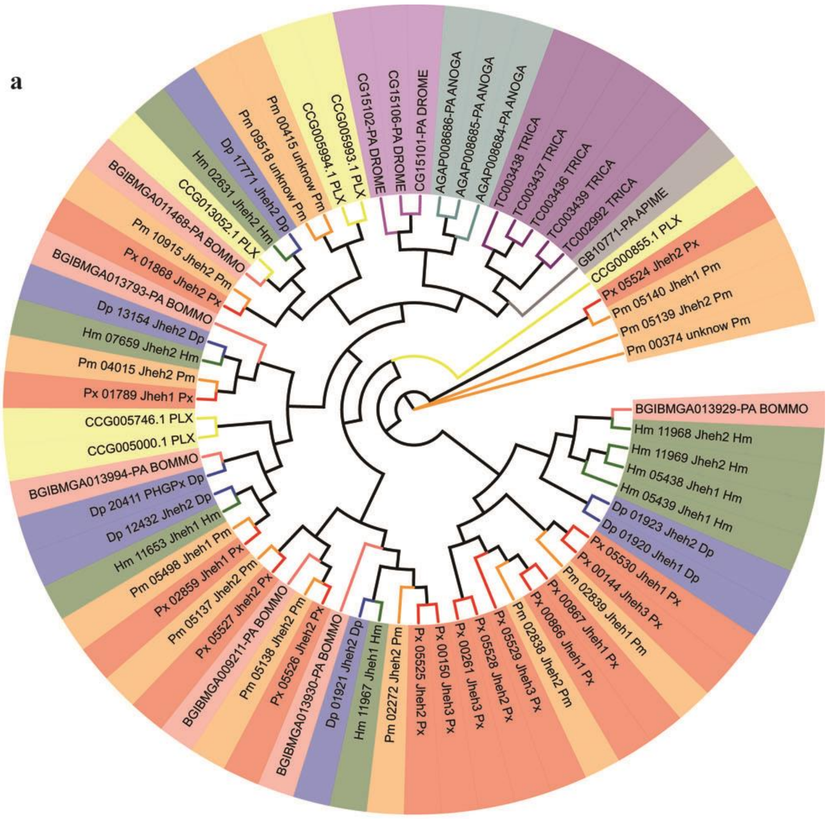

b

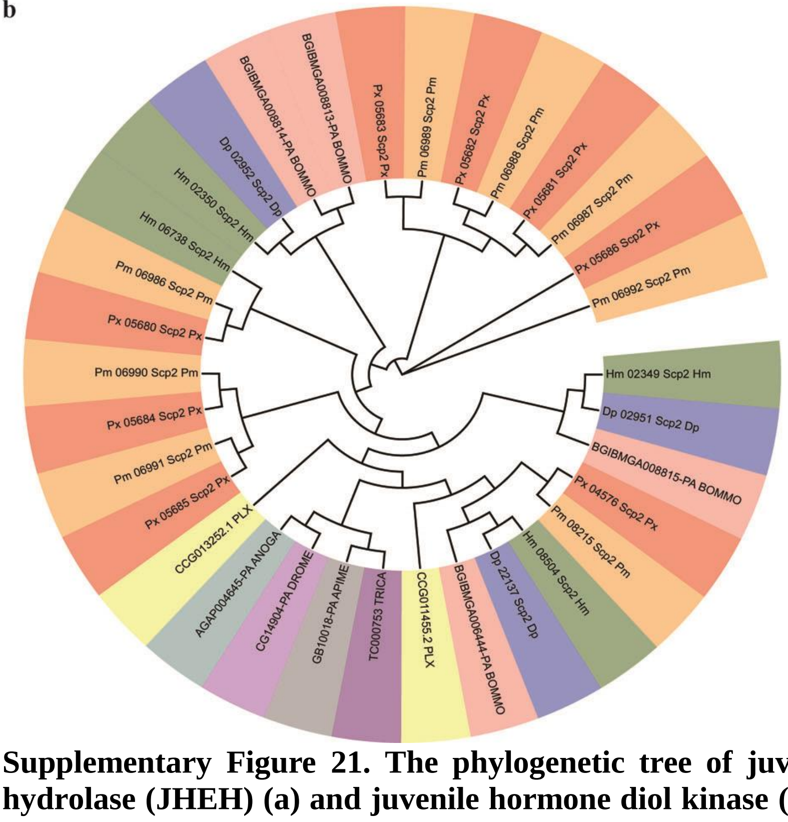

Supplementary Figure 21. The phylogenetic tree of juvenile hormone epoxide hydrolase (JHEH) (a) and juvenile hormone diol kinase (JHDK) (b) identified in 10 holometabolous insects. Including 4 butterflies: *P. xuthus* (*Px*), *P. machaon* (*Pm*), *H. melpomene* (*Hm*), *D. plexippus* (*Dp*); 2 moths: *B. mori* (BGIBMGA), *P. xylostella* (CCG); 1 fruitfly: *D. melanogaster* (CG); 1 mosquito: *A. gambiae* (AgAP); 1 bee: *A. mellifera* (GB); 1 beetle: *T. castaneum* (TC).

a

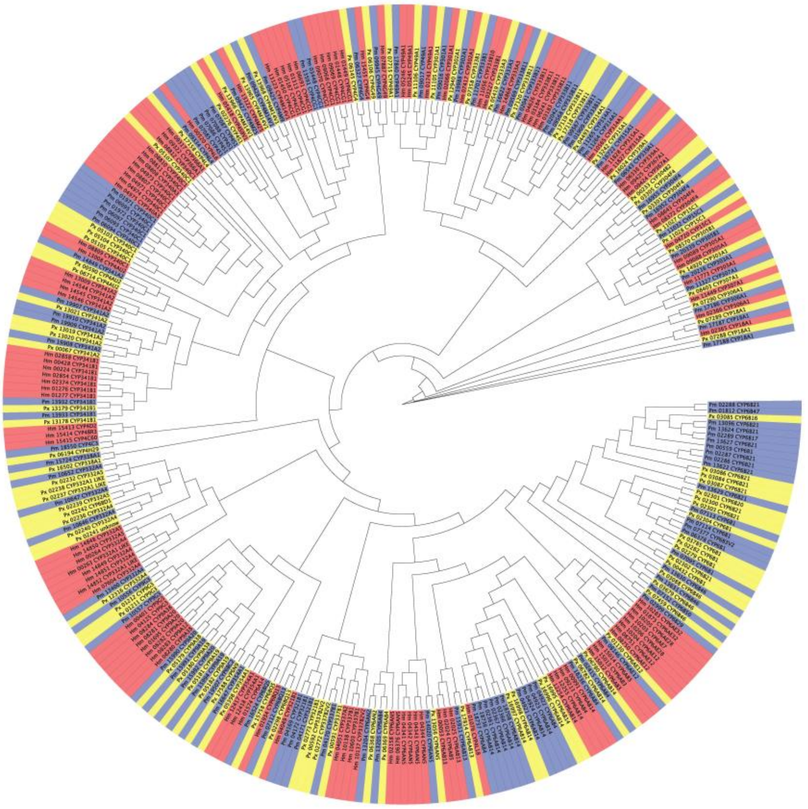

b

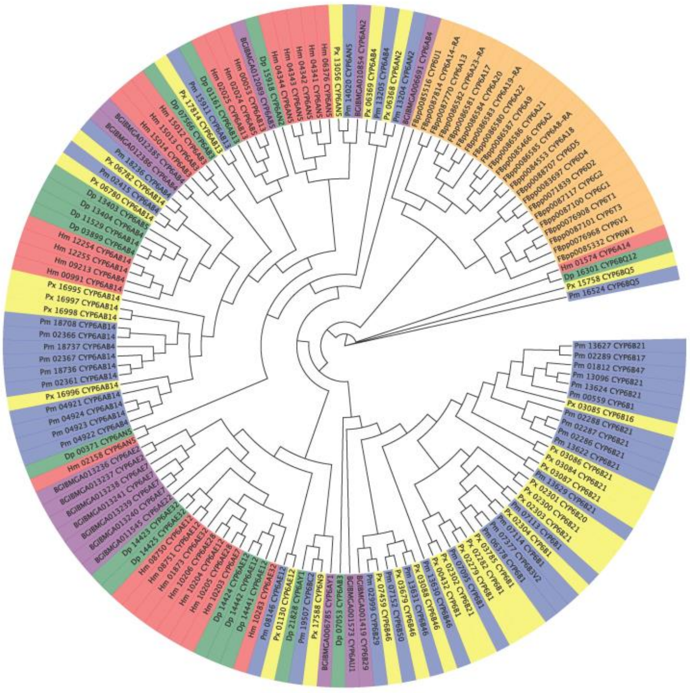

Supplementary Figure 22. The phylogenetic tree of Cytochrome P450 (P450). a, all P450 genes in *Pm* (blue), *Px* (yellow) and *Hm* (red). **b**, CYP6 members in 4 butterflies, silkworm and fruitfly. 4 butterflies: *P. xuthus* (*Px*), *P. machaon* (*Pm*), *H. melpomene* (*Hm*), *D. plexippus* (*Dp*); silkworm: *B. mori* (BGIBMGA); 1 fruitfly: *D. melanogaster* (CG).

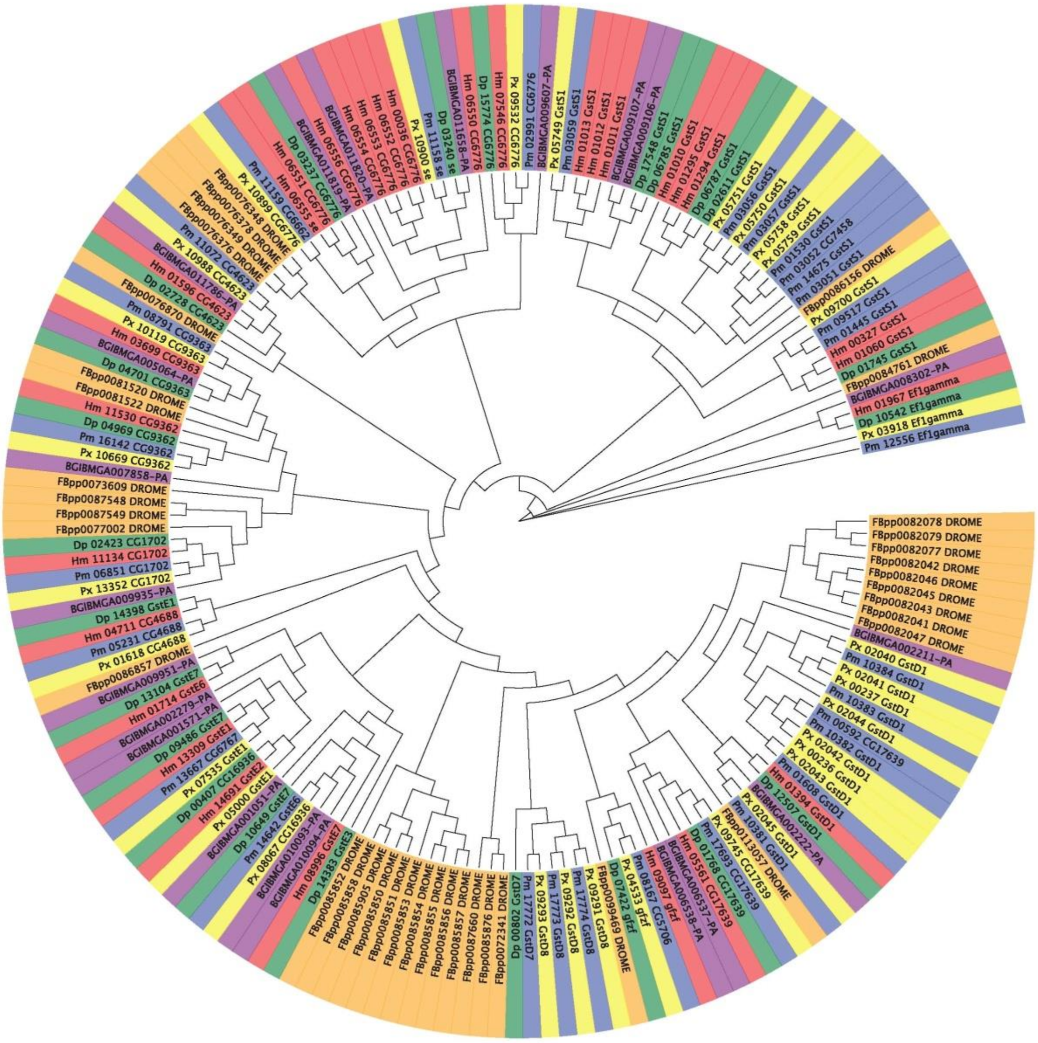

Supplementary Figure 23. The phylogenetic tree of Glutathione S-transferase (GST) in 4 butterflies, silkworm and fruitfly. 4 butterflies: *P. xuthus* (*Px*), *P. machaon* (*Pm*), *H. melpomene* (*Hm*), *D. plexippus* (*Dp*); 2 moths: *B. mori* (BGIBMC), *P*. *xylostella* (CCG); 1 fruitfly: *D. melanogaster* (CG); 1 mosquito: *A. gambiae* (AgAP); 1 bee: *A. mellifera* (GB); 1 beetle: *T. castaneum* (TC).

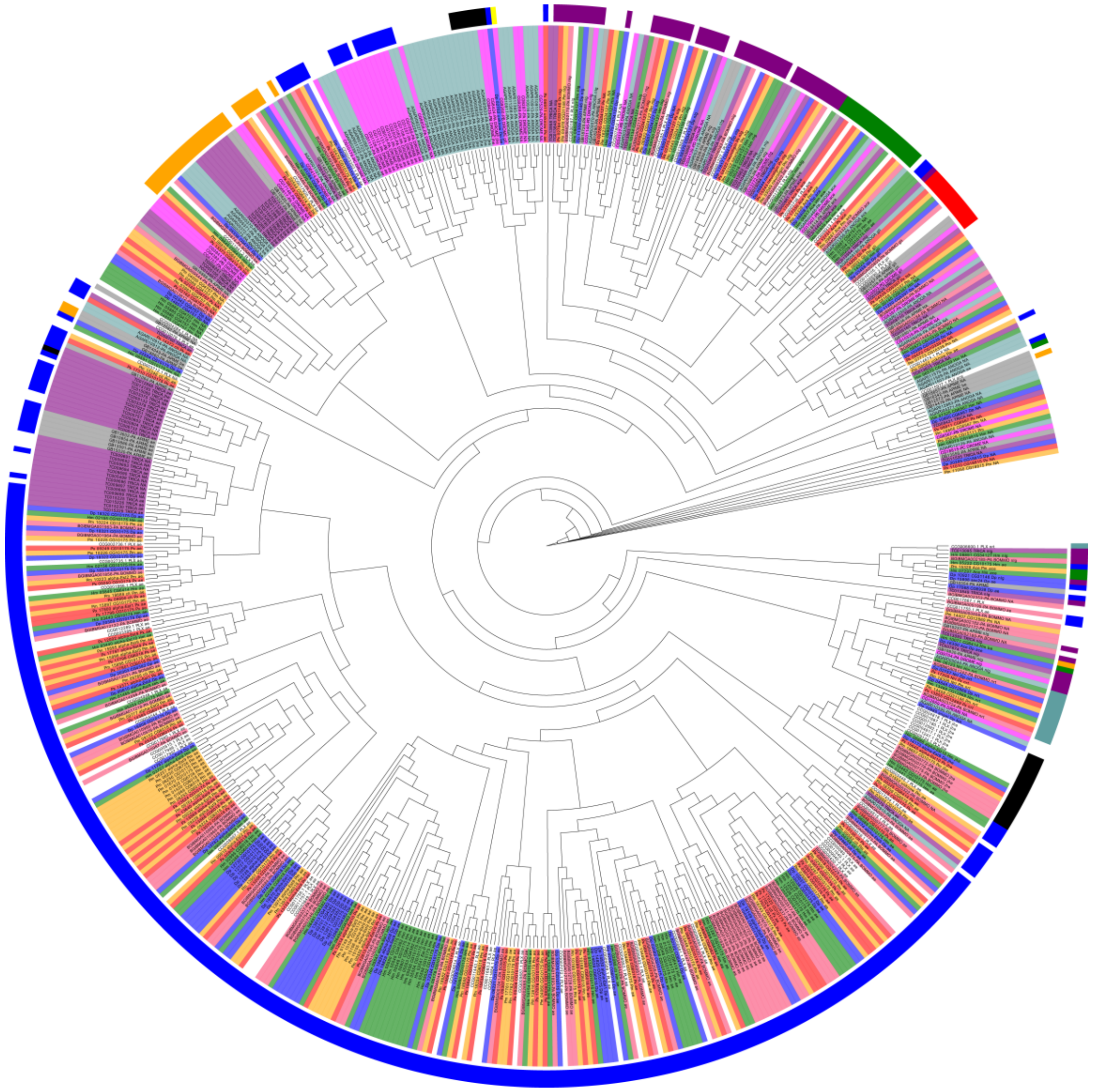

Supplementary Figure 24. The phylogenetic tree of carboxylesterases (COE) in 10 holometabolous species. 10 holometabolous insects include 4 butterflies: *P. xuthus* (*Px*), *P. machaon* (*Pm*), *H. melpomen*e (*Hm*), *D. plexippus* (*Dp*); 2 moths: *B. mori* (BGIBMGA), *P. xylostella* (CCG); 1 fruitfly: *D. melanogaster* (CG); 1 mosquito: *A. gambiae* (AgAP); 1 bee: *A. mellifera* (GB); 1 beetle: *T. castaneum* (TC)). Green; ace (acetylcholinesterase); Blue: ae (alpha-esterase); Orange: be (betaesterase); Red: gli (gliotactin); Yellow: glt (glutactin); Grey: hydr (hydrolase); Black: jhe (juvenile hormone esterase); Purple: nlg (neneuroligin); Cadet Blue: nrt (neurotactin)
.

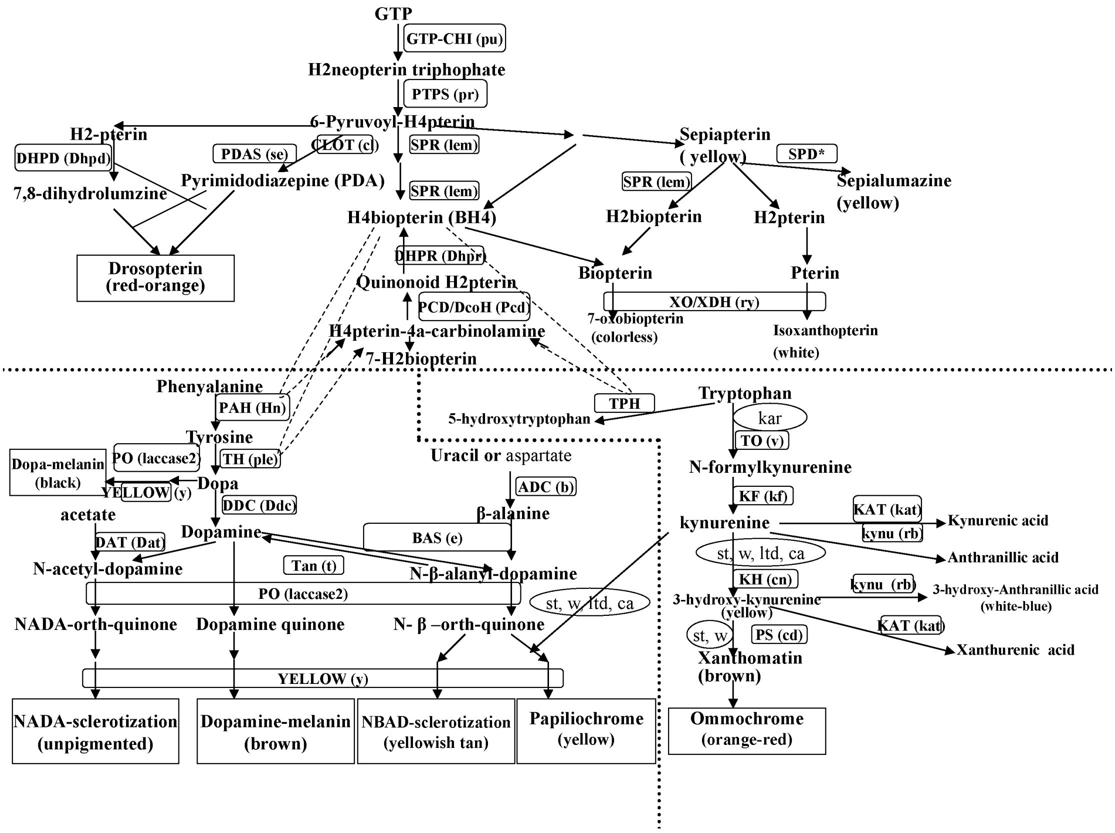

Supplementary Figure 25. The pathways for the synthesis of pteridine, melanin, ommochromes, papiliochrome, curticle sclerotization, and the interrelationship among these pathways. After Ferre et al., 1986[4]; Ficner et al., 1995[5]; Borycz et al., 2002[6]; Ziegler, 2003[7]; Wittkopp et al., 2003[8]; Kato et al, 2006[9]; Meng et al., 2009[10]; Ferguson et al, 2009[11]; Kim et al., 2009[12]; Nijhout, 2010[13]; Andersen, 2010[14]. Abbreviation for enzymes and related proteins, and the genes encoding these enzymes and proteins: GTP-CHI, GTP cyclohydrolase I encoding by *punch* (pu); PTPS, 6-pyruvoyl-tetrahydropterin synthase encoding by *purple* (pr); SPR, sepiapterin reductase encoding by *lemon* (lem); SPD*, sepiapterin deaminase, gene encodling it unknown; PDAS, PDAS synthase encoding by *sepia* (se); CLOT, the protein coding by *clot* (cl); DHPD, dihydropterin deaminase encoding by *DhpD*; XOD, xanthine oxidase encoding by *rosy* (ry); XDH, xanthine dehydrogenase encoding by *rosy* (sy); PCD, pterin-4a-carbinolamine dehydratase encoding by*Pcd*; DHPR, dihydrobiopterin reductase encoding by *Dhpr*; PAH, phenyalanine hydroxylase encoding by *Henna* (Hn); TH, tyrosine hydroxylase encoding by *pale* (ple); TPH, Tryptophan hydroxylase encoding by *Tph*; DDC, dopa-decarboxylase encoding by *Ddc*; DAT, N-β-acetyl-dopamine transferase encoding by *Dat*; ADC, Aspartate-1-decarboxylase encoding by *black* (b); BAS, N-β-alayldopamine synthase encoding by *ebony* (e); TAN, N-β-alayldopamine hydrolase encoding by *tan* (t); PO, phenoloxidase encoding by *laccase 2*; YELLOW, the proteins of *yellow* (y) gene family; TO, Tryptophan oxidase encoding by *vermillion* (v); KF, Kynurenine formamidase encoding by *kf*; KH, Kynurenine-3-hydroxylase encoding by *cn*; PS, Phenoxazinone synthetase encoding by *cd*; KAT: Kynurenine aminotransferase encoding by kaf; Kynu: Kynureninase encoding by *ruby* (rb); kar, karmoisin; st, scarlet; w, white; ltd, lightoid; ca, claret.

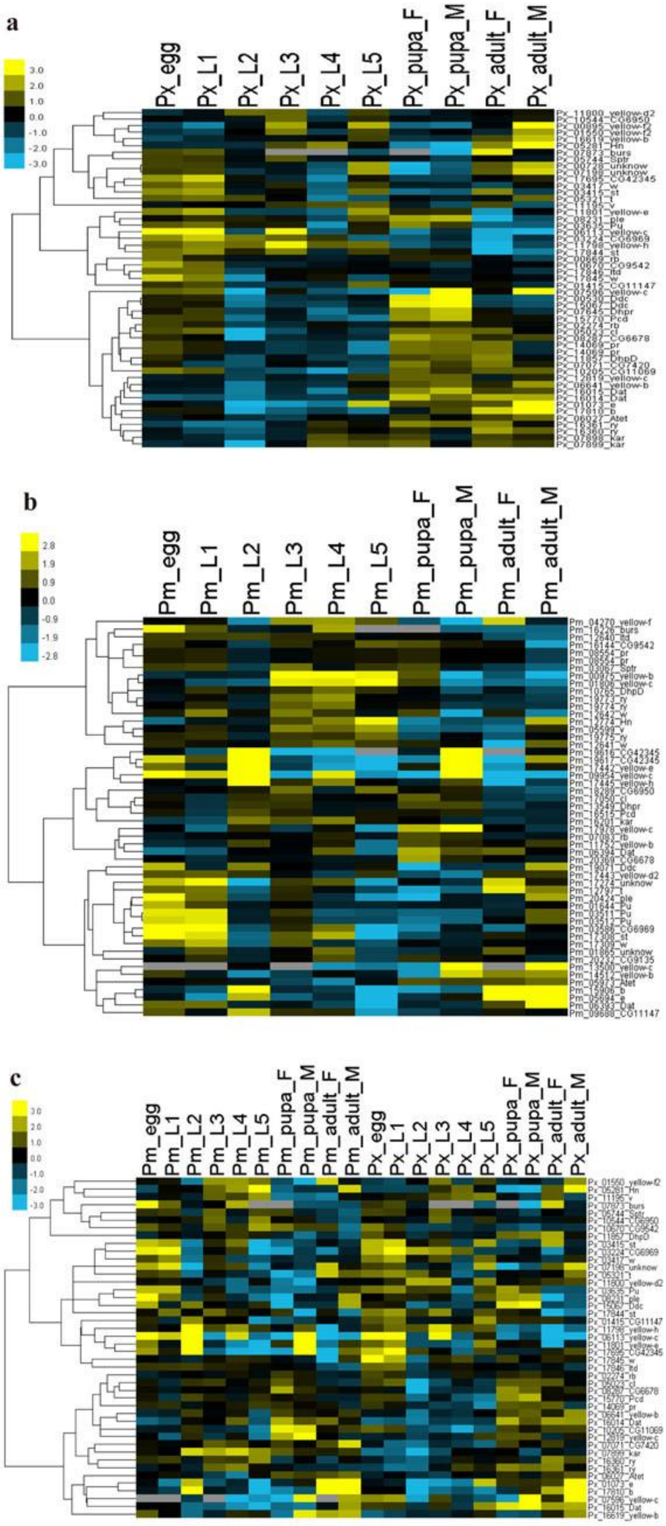

Supplementary Figure 26. Expression pattern of pigmentation genes in the pathways of synthesis of pteridine, melanin, ommochromes, papiliochrome and curticle sclerotization of *P. xuthus* (*Px*) and *P. machaon* (*Pm*). a, *Px*; **b,** *Pm*; **c,** Orthogous pigmentation genes of *Px* and *Pm***.**

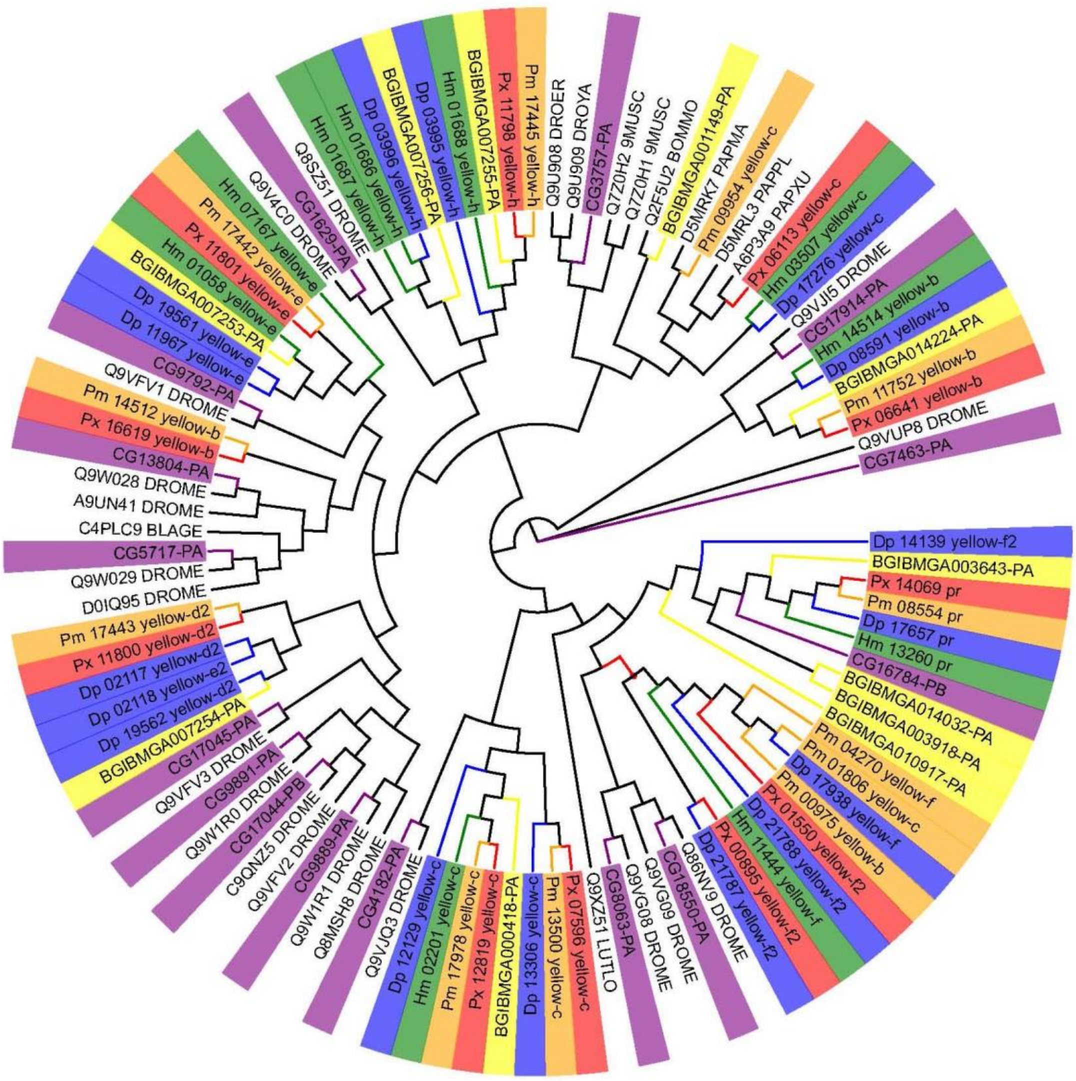

Supplementary Figure 27. The phylogenetic tree of all members of yellow family identified in four butterflies, silkworm and fruitfly, as well as those downloaded in insects from uniprot**.** 4 butterflies: *P. xuthus* (*Px*), *P. machaon* (*Pm*), *H. melpomene* (*Hm*), *D. plexippus* (*Dp*); 2 moths: *B. mori* (BGIBM), 1 fruitfly: *D. melanogaster* (CG).

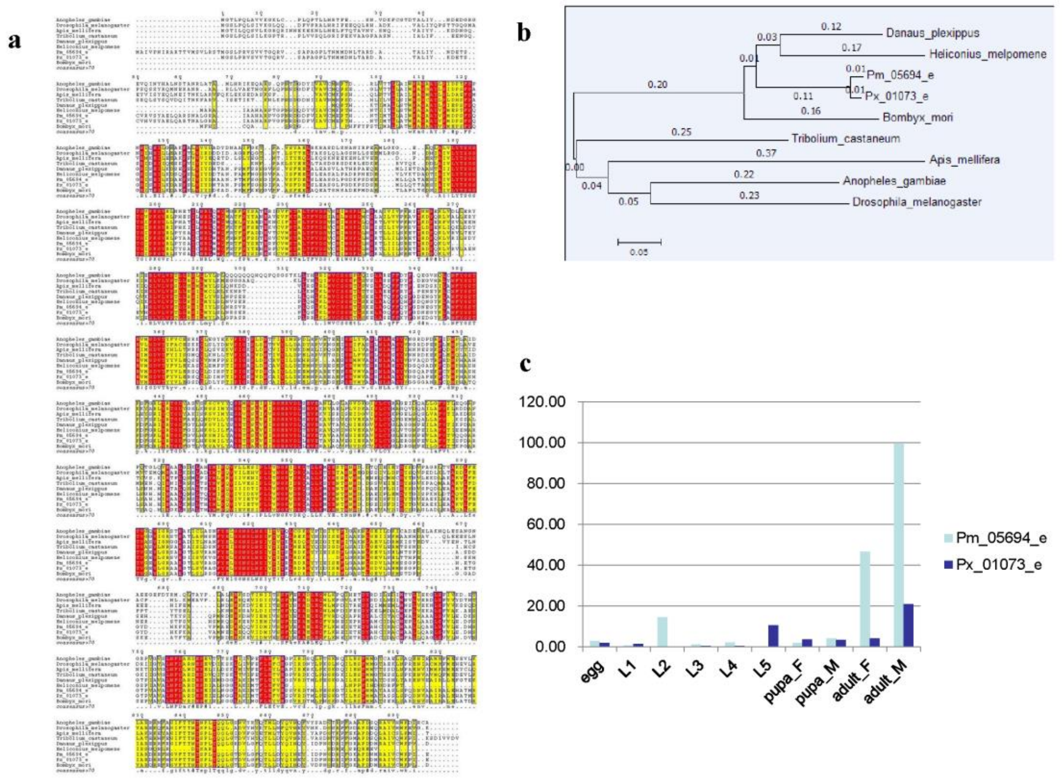

Supplementary Figure 28. Comparisons of *ebony* gene between *P. xuthus* (*Px*) and *P. machaon* (*Pm*). a, Alignment of amino acid. **b**, Gene tree of *ebony* constructed based on amino acid and drawn to scale at the bottom, with branch length (next to the branches) which isin the unit of the number of nucleotide substitutions per site. **c**, Expression of *ebony* along the developmental stages inferred by RNA-seq.

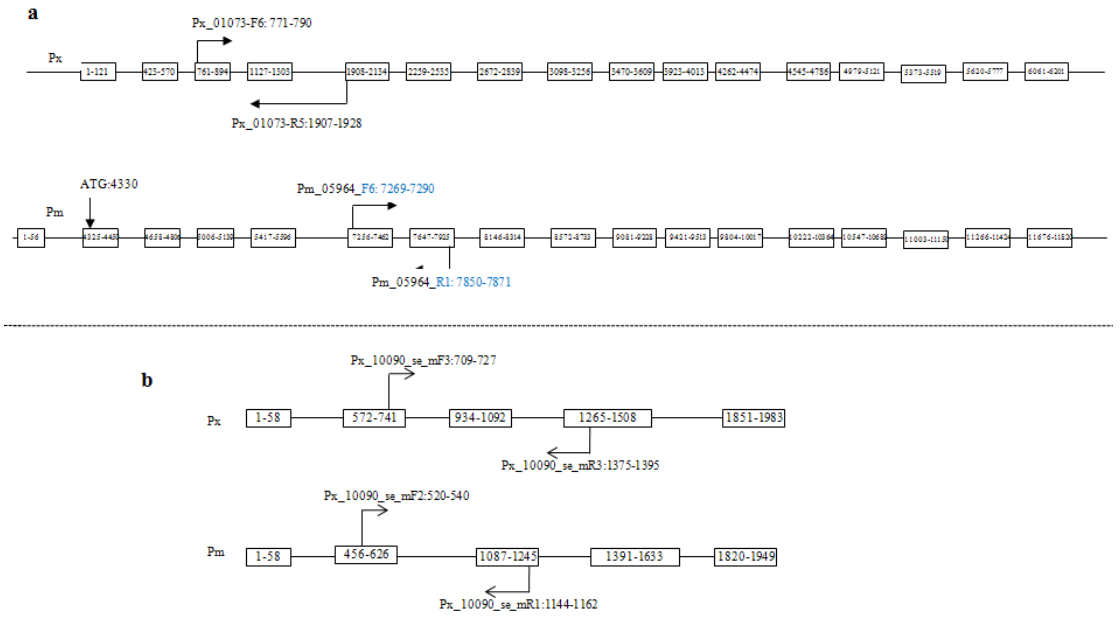

Supplementary Figure 29. The gene structure of *ebony* and *sepia*, and the primer locations of RT-PCR in *P. xuthus* (*Px*) and *P. machaon* (*Pm*). **a**, *ebony*. **b**, *sepia*.

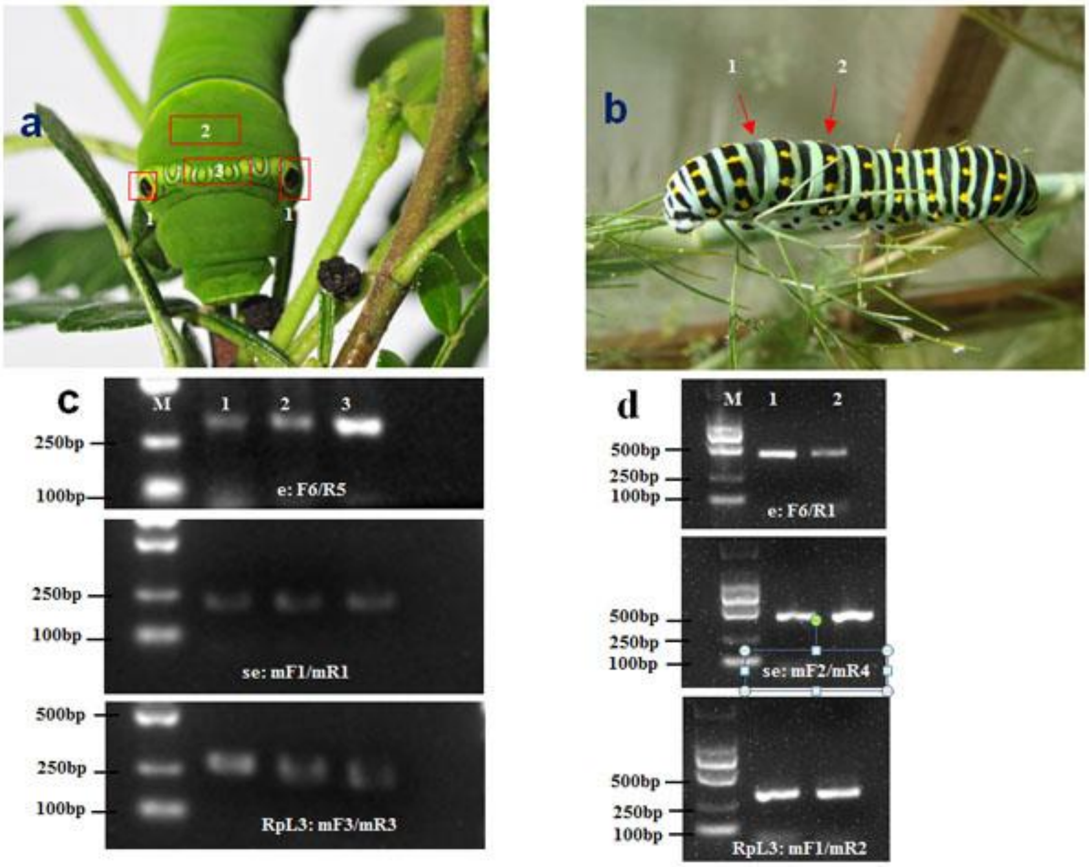

Supplementary Figure 30. RT-PCR of *ebony* and *sepia* gene in *P. xuthus* (*Px*) and *P. machaon* (*Pm*). **a**, Sampling region of *Px*. **b**, Sampling region of *Pm*. **c**, Electrophoresis pictures of RT-PCR for tissue sampling in *Px* (**a**) for gene *ebony* (top), *sepia* (middle) and internal control *RpL3* (bottom). **d**, Electrophoresis pictures of RT-PCR for tissue sampling in *Pm* (**b**) for gene *ebony* (top), *sepia* (middle) and internal control *RpL3* (bottom). M: DL2000 DNA Marker.

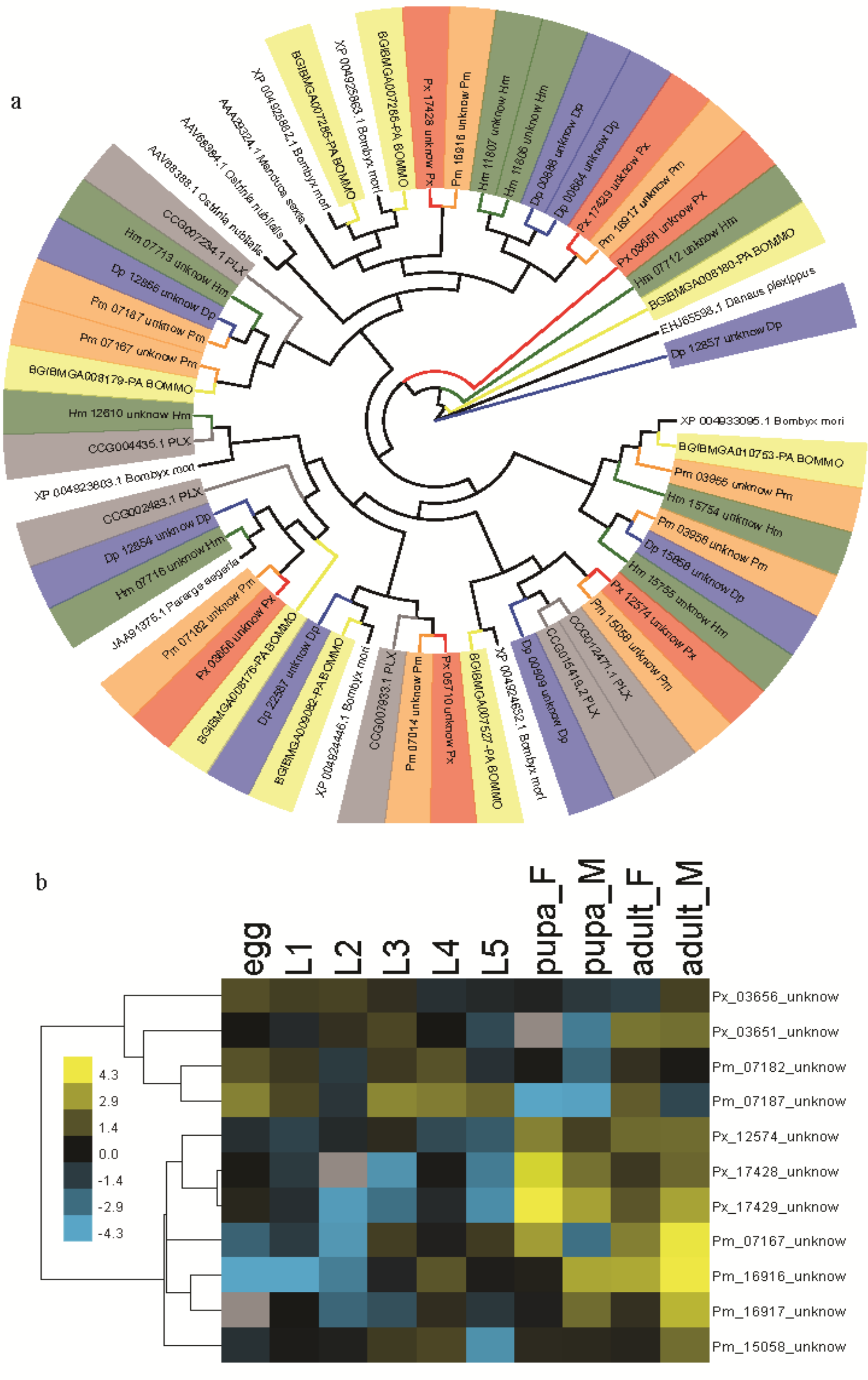

Supplementary Figure 31. Analyis of Lepidoptera-specific ommochrome-binding proteins (Ombps). a, **T**he phylogenetic tree of all members of Ombps identified in four butterflies, silkworm and moth, as well as those downloaded from GenBank. 4 butterflies: *P. xuthus* (*Px*), *P. machaon* (*Pm*), *H. melpomene* (*Hm*), *D. plexippus* (*Dp*); 2 moths: *B. mori* (BGIBM), *P. xylostella* (CCG); 1 fruitfly: *D. melanogaster* (CG). **b**, Expression pattern of Ombps in differentially developmental stages of *Pm* and *Px*.

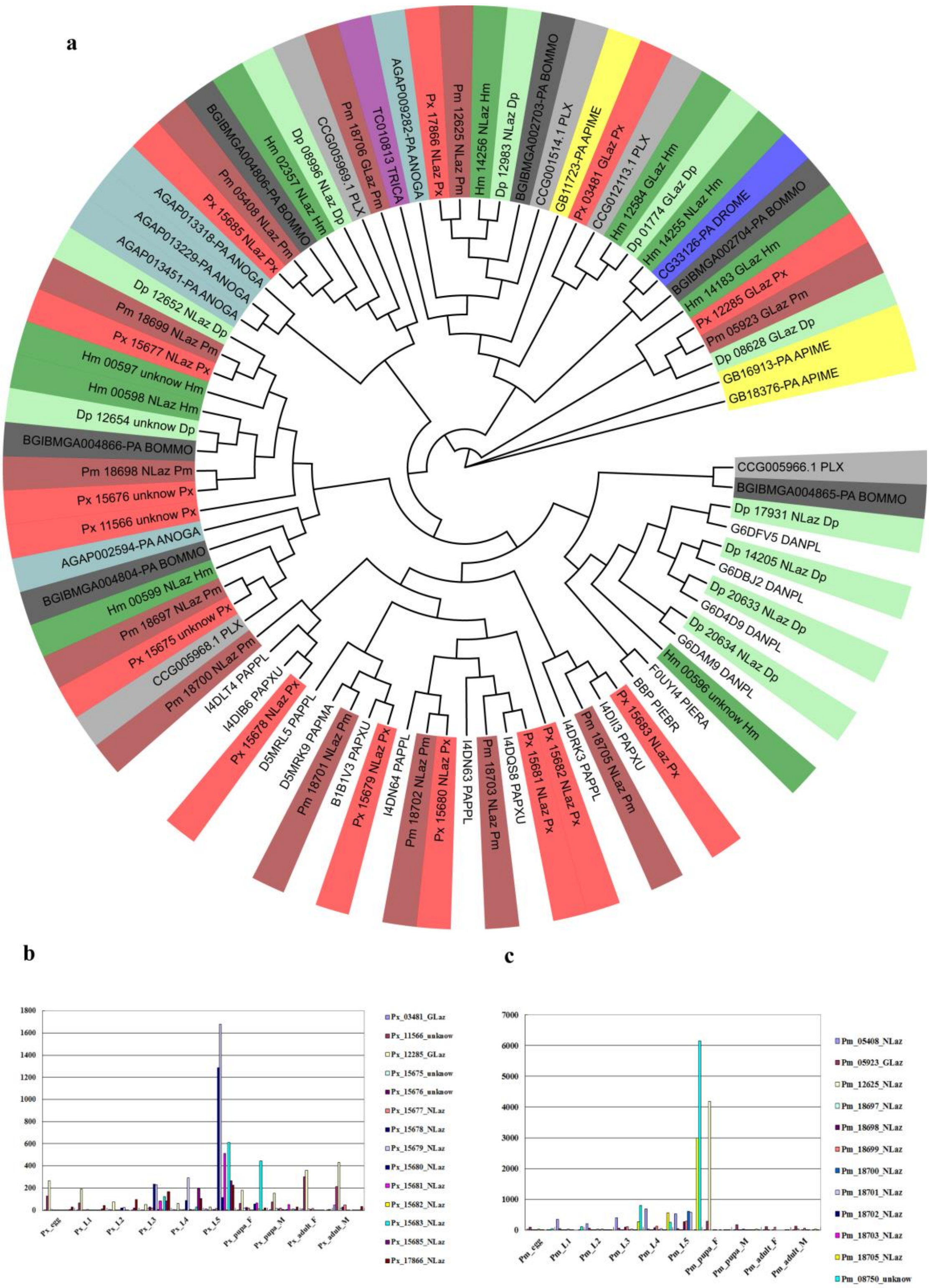

Supplementary Figure 32. Analayis of bilin-binding protein (BBP). a, The phylogenetic tree of BBP identified in the genomes and cloned. 4 butterflies: *P. xuthus* (*Px*), *P. machaon* (*Pm*), *H. melpomene* (*Hm*), *D. plexippus* (*Dp*); 2 moths: *B. mori* (BGIBM), *P. xylostella* (CCG); 1 fruitfly: *D. melanogaster* (CG); 1 mosquito: *A. gambiae* (AgAP); 1 bee: *A. mellifera* (GB); 1 beetle: *T. castaneum* (TC). **b**, Expression pattern of BBP genes in *Px*. **c**, Expression pattern of BBP genes in *Pm*.

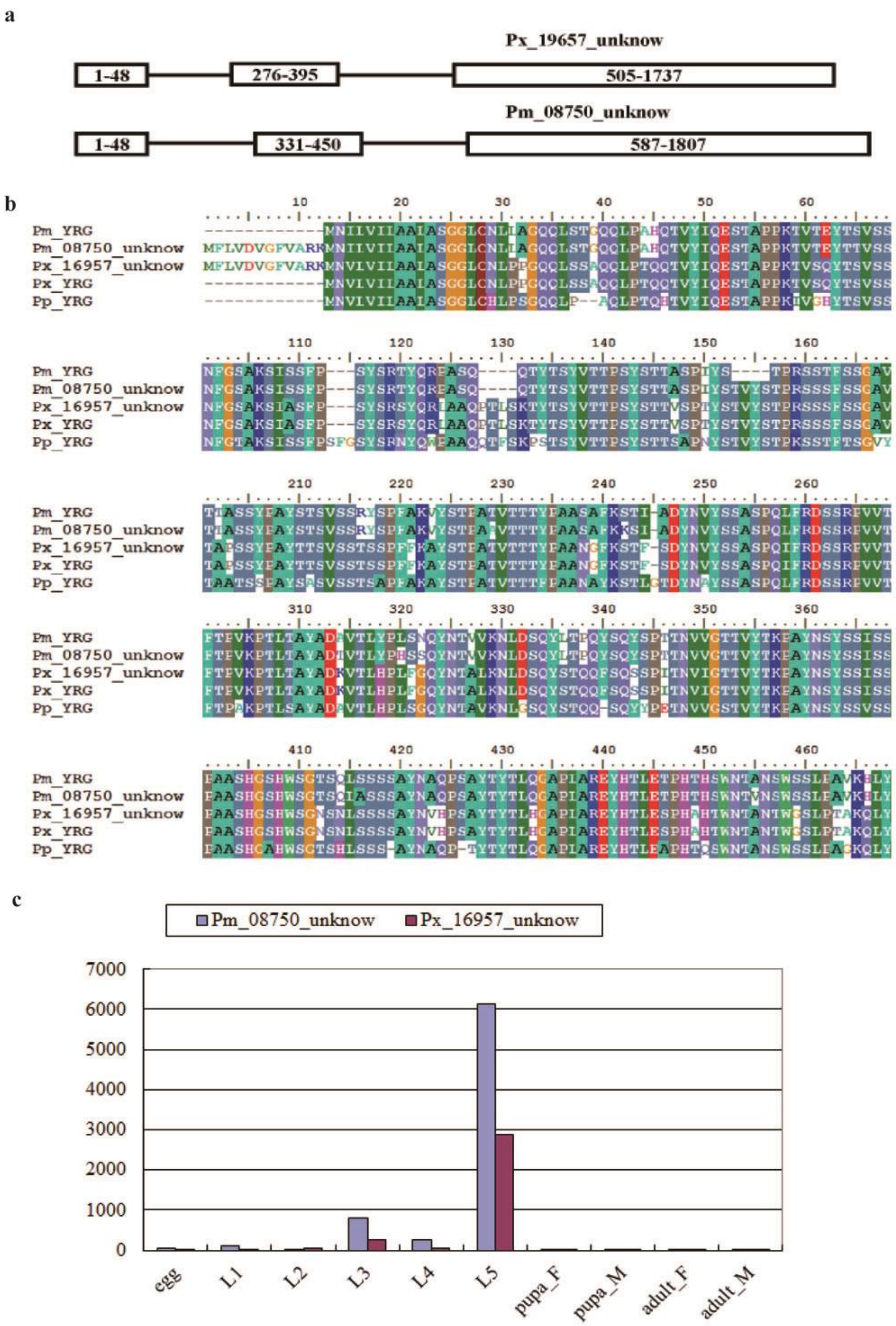

Supplementary Figure 33. Characteristics of yellow related genes (YRG). a**,** Genomic structure of YRG in *P. xuthus* (*Px*) and *P. machaon* (*Pm*). **b**, Alignment of YRG of *Pm*, *Px*, *P. poletyes*. **c**, Expression pattern of YRG in *Pm* and *Px*.

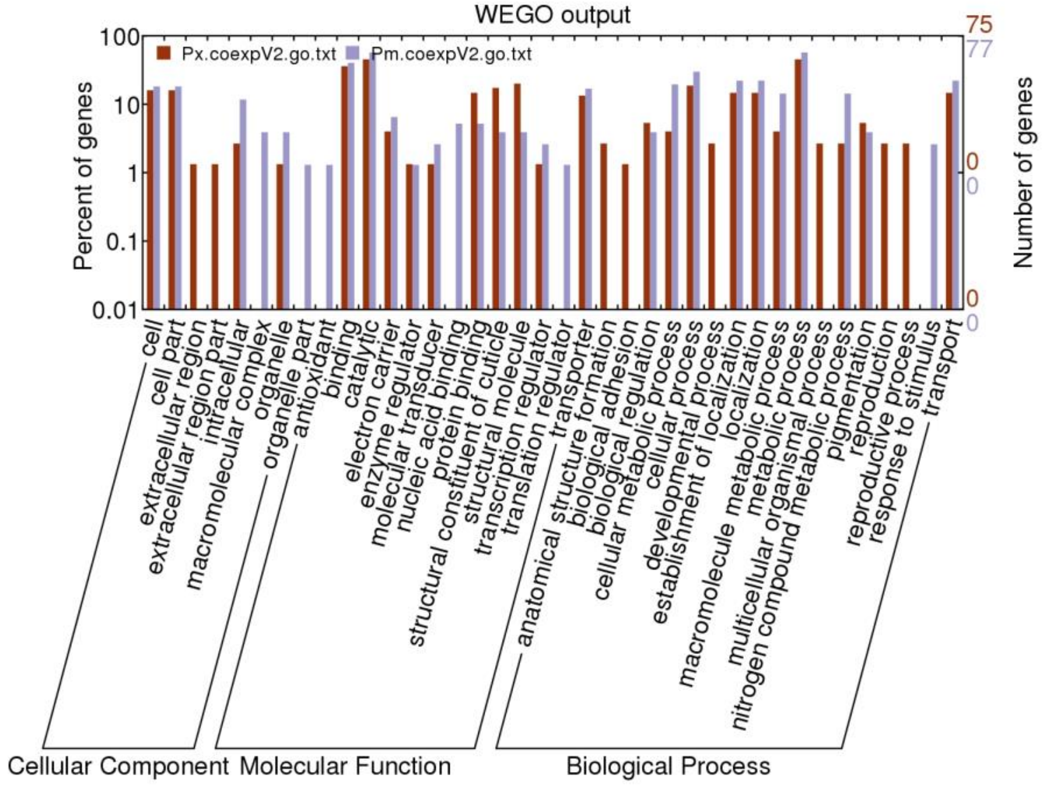

Supplementary Figure 34. Wego analysis of co-expression genes with yellow related genes (YRG) in *P. xuthus* (*Px*) and *P. machaon* (*Pm*). Wego analysis was carried out with BGI WEGO (http://wego.genomics.org.cn/cgi-bin/wego/index.pl).

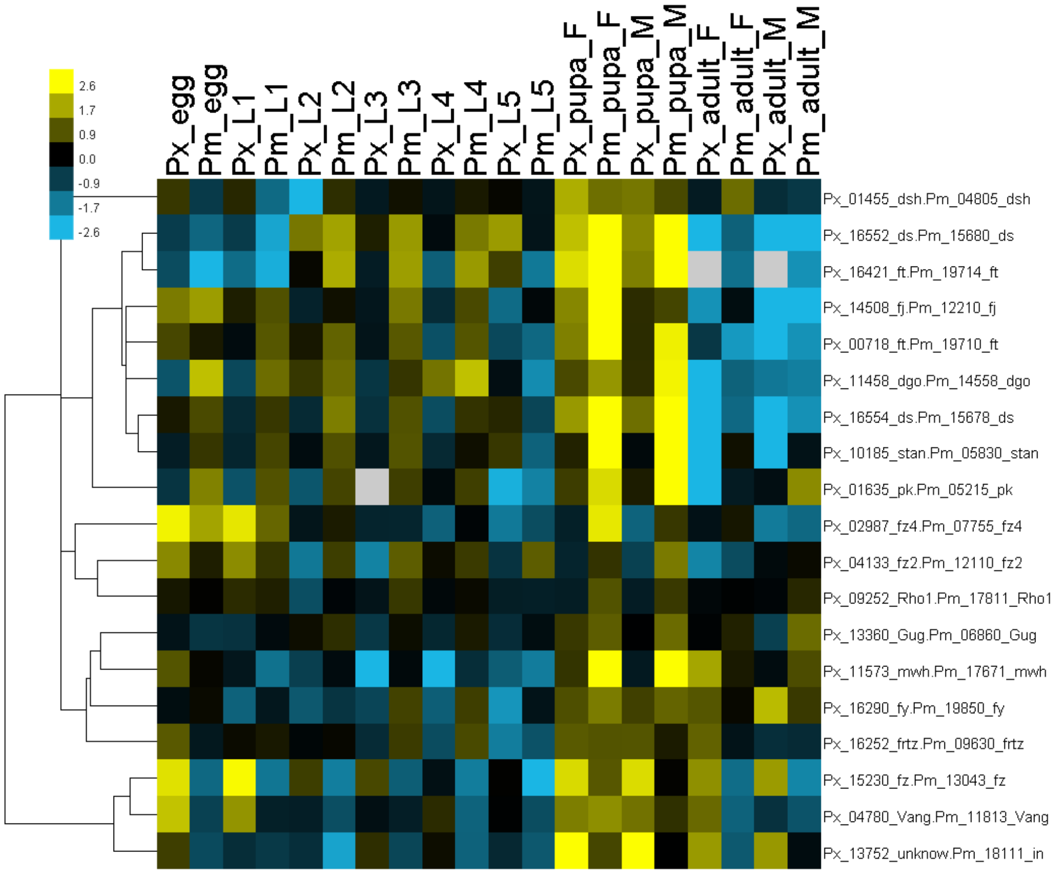

Supplementary Figure 35. Expression of planar cell polarity (PCP) orthologous genes in *P. xuthus* (*Px*) and *P. machaon* (*Pm*).

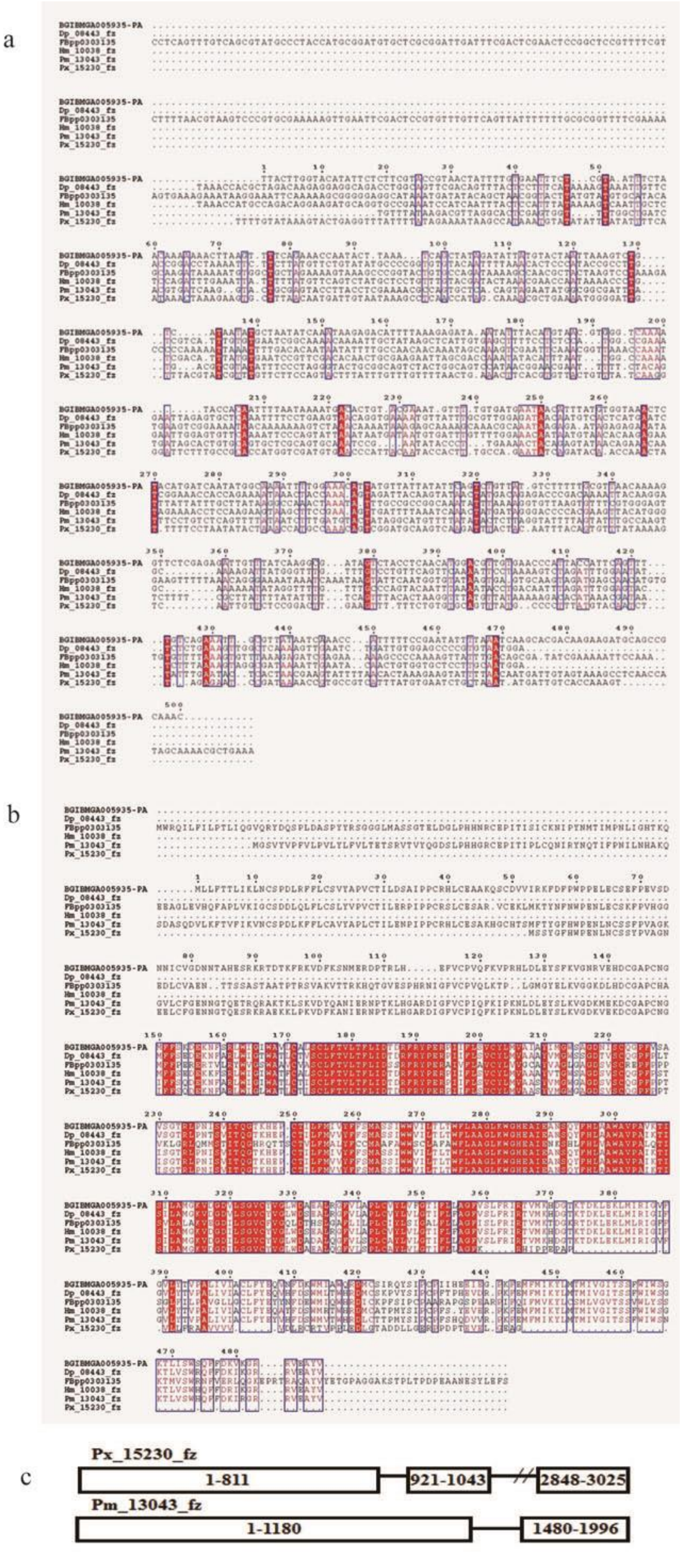

Supplementary Figure 36. Comparison of *frizzled* gene between *P. xuthus* (*Px*) and *P. machaon* (*Pm*). a, 500 bp of 5’ UTR; **b**, CDS; **c**, Gene structure.

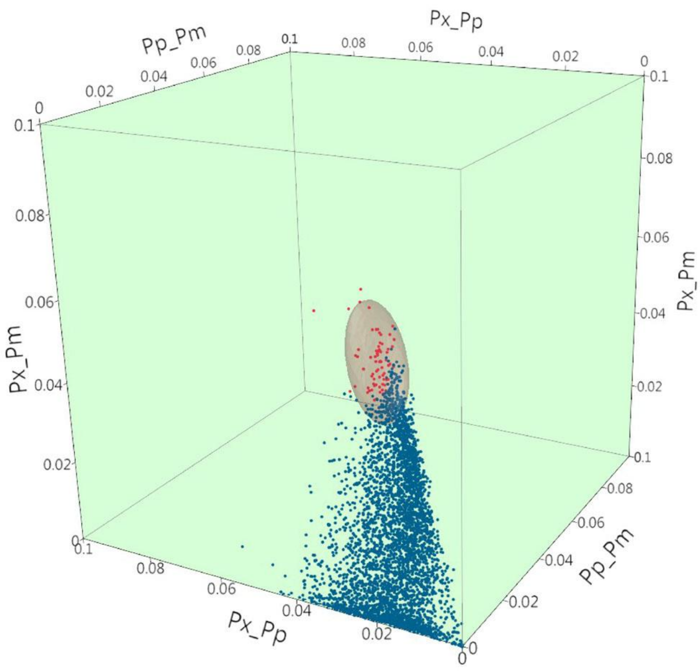

Supplementary Figure 37. The distribution of the highly divergent outlier regions. Red dots indicate the 70 outlier regions with the values of the degree of freedom above 95% smoothed empirical likelihood quantiles in all three comparisons (*Pm*/*Px*, *Px*/*Pp*, *Pm*/*Pp*). *Pm* (*P. machaon*), *Px* (*P. xuthus*), *Pp*（*Papilio polytes*). Blue dots indicate the rest of the estimated regions except the 70 outliers.

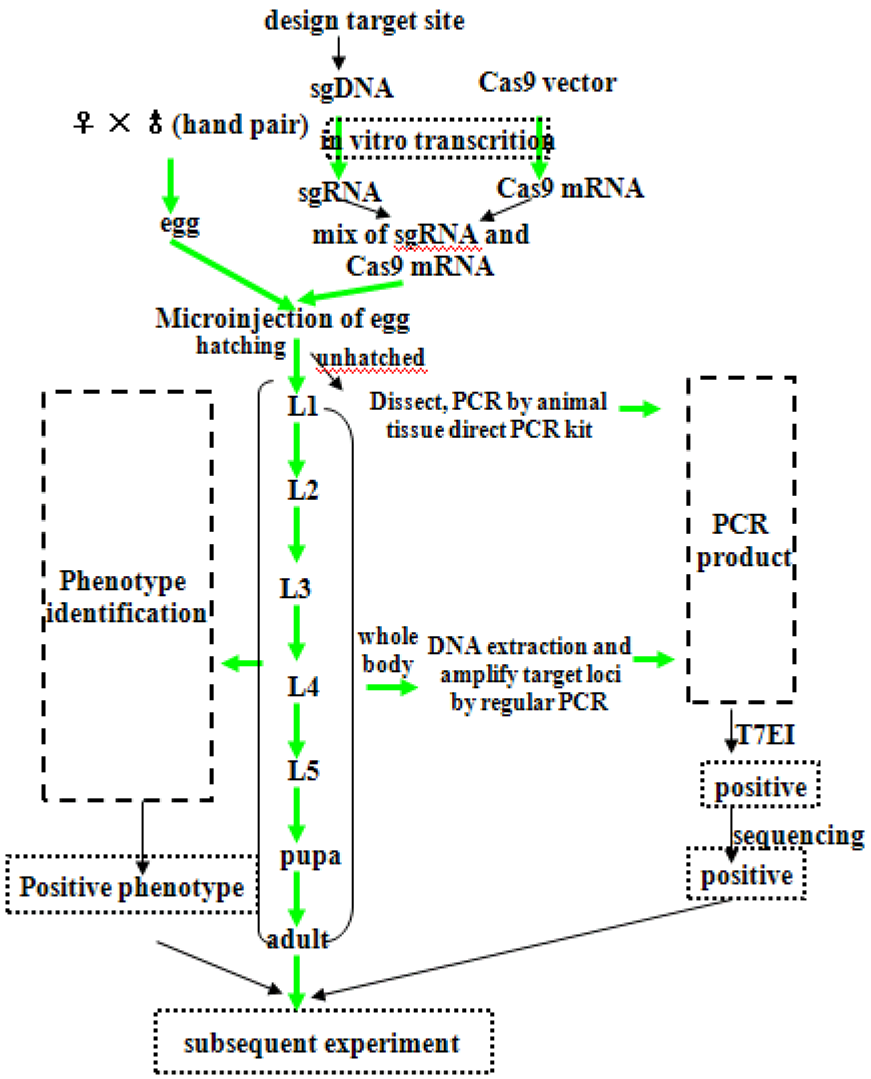

Supplementary Figure 38. Flowchart of CRISPR/Cas9 technology in butterfly. L1 (2, 3, 4, 5): 1st (2nd, 3rd, 4th, 5th) instar larvae; T7EI: T7 endonuclease I mutation detection assays.

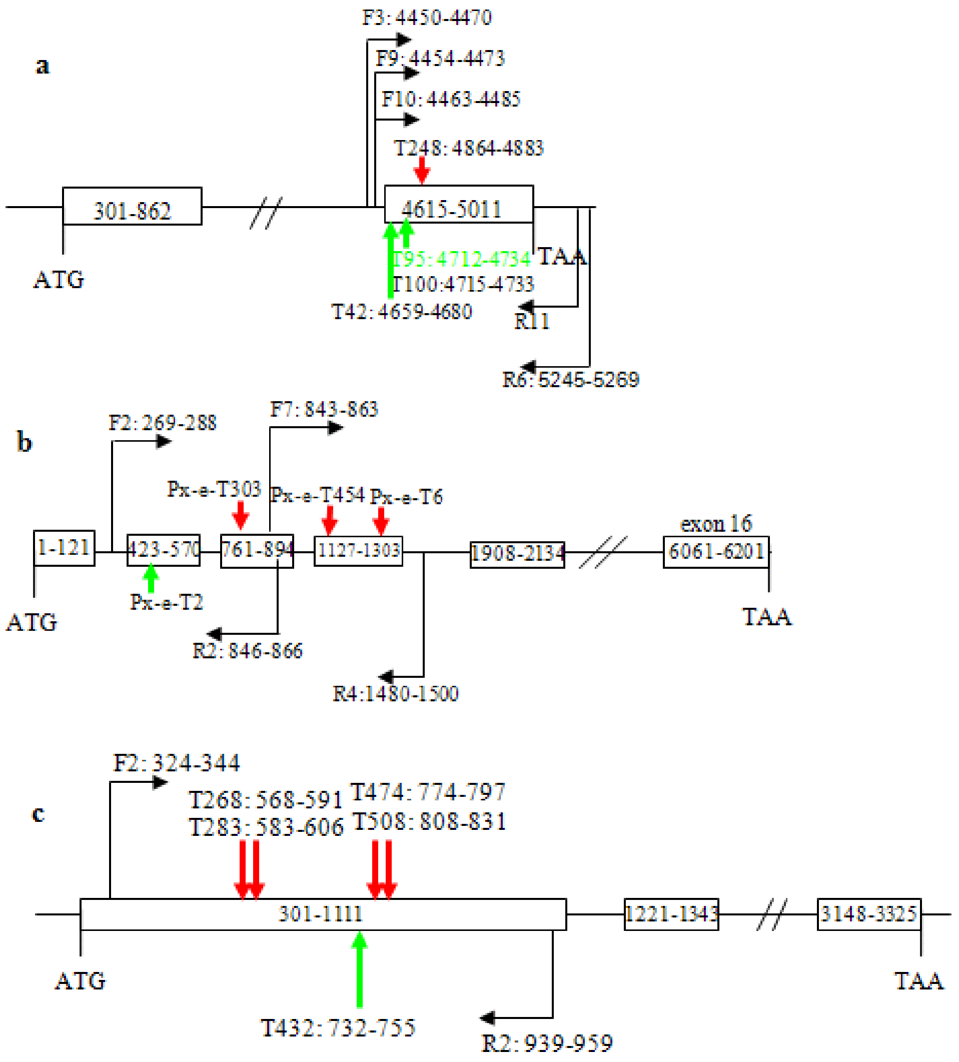

Supplementary Figure 39. Schematic diagram of the sgRNA-targeting sites and primers used for amplifying target sites of different genes in *P. xuthus*. a, *Px_03961_Abd-B*; **b**, *Px_01073_e* (*ebony*); **c**, *Px_15230_fz* (*frizzled*). sgRNA: single guide RNA. Boxes represent exons. Lines represent intron or untranslated regions (UTR) of 5’ or 3’; //: long intron; T: Cas9 targets with the number after colon showing the location; red arrow: targets in plus strand; green arrows: targets in minus strand. F: forward primer with the number after colon showing the location; R: reverse primer with the number after semicolon showing the location. In **(a)** (*Px_03961_ Abd-B*) and **(c)** (*Px_15230_fz*), the fragments including 300 bp of 5’ UTR, gene region and 300 bp of 3’ UTR. In **(b)** (*Px_01073_e*), the fragment includes only gene region from the start codon ATG to the stop codon TAA

.

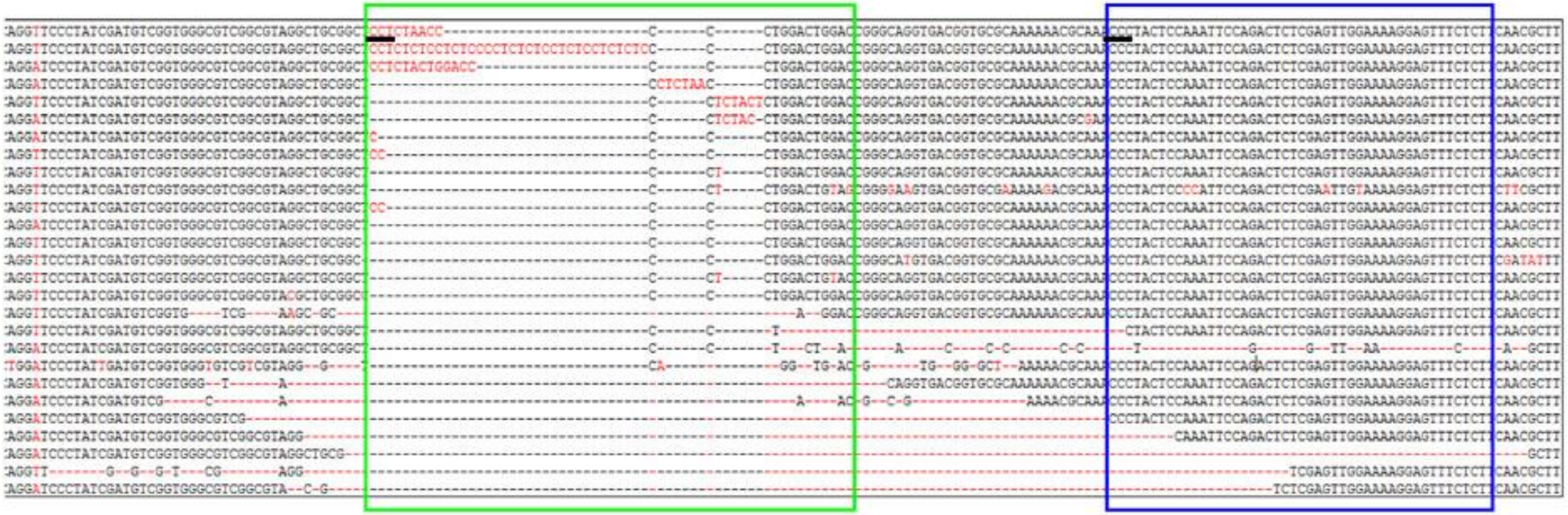

Supplementary Figure 40. Sequencing two disrupted adjacent target sites (T42, T95) of *Abd-B* gene for the injected individuls of treatment I (co-injection of the three sgRNAs (T42, T95, T248) and Cas9 mRNA) in *P. xuthus*. The first line of the alignment represents wild-type sequence, and subsequent lines show individual mutant clones. The T42 target sites are in the green line square, and the T95 target sites are in the blue line square. PAMs are underlined. The third target without disruption is not shown here.

.

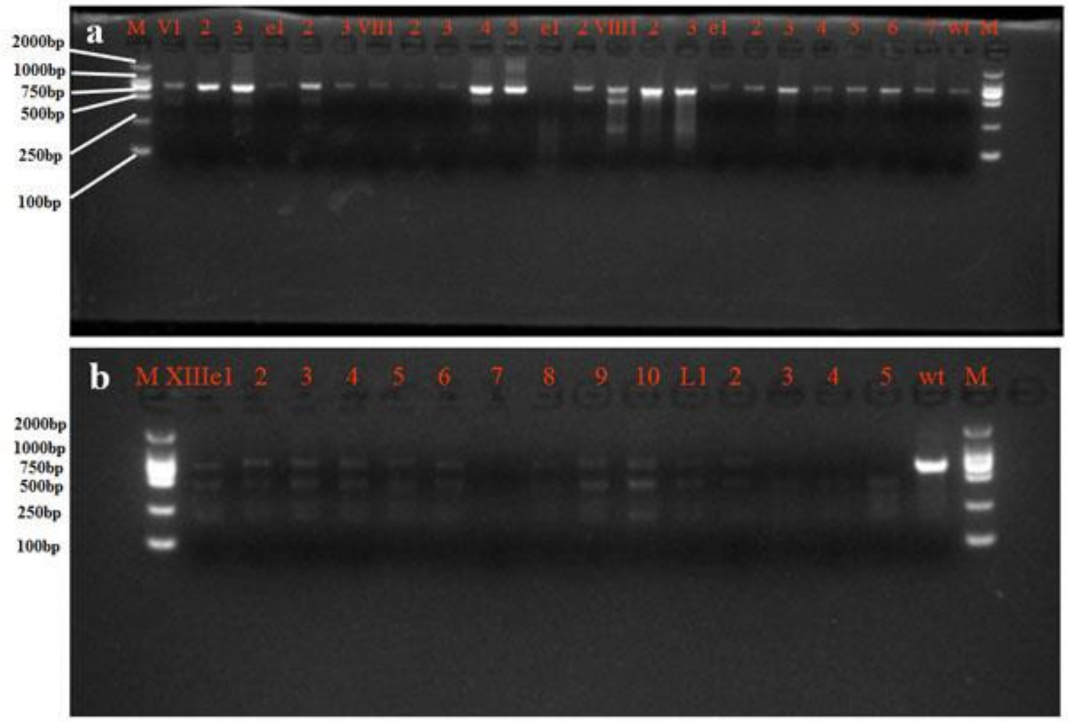

Supplementary Figure 41. Electrophoresis pictures of a T7 endonuclease (T7EI) assay for all morphological mutants of *Abd-B* in *P. xuthus* in the injection treatments V, VII, VIII (a), and some of XIII (b). M: DL2000 DNA Marker. wt: wild type.

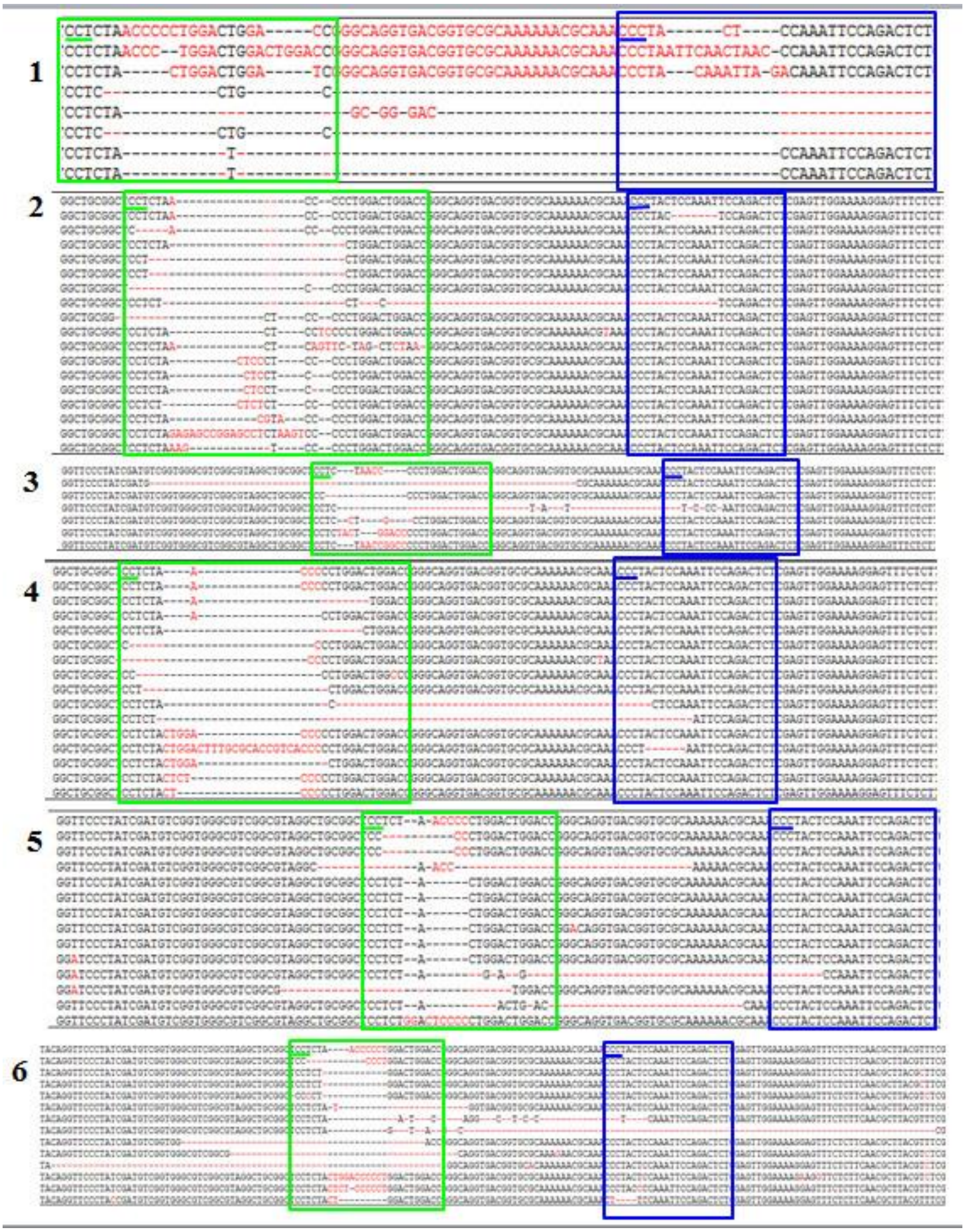

Supplementary Figure 42. Sequencing two adjacent target sites (T42, T95) in different types of morphological mutants induced by co-injection of the two sgRNAs and Cas9 mRNA of the *Abd-B* gene in *P. xuthus*. The first line for each alignment represents wild-type sequence, and subsequent lines show individual mutant clones. The T42 target sites are in the green line square, and the T95 target sites are in the blue line square. PAMs are underlined. The relationship to morphological mutants in **Suplementary Figure 43**: 1: **a**; 2: **b**; 3: **c**; 4: **d**; 5: **e**; 6: without observable phenotype (not shown in **Suplementary Figure 43)**. Target sequences are on minus strand, and here their plus strands are shown.

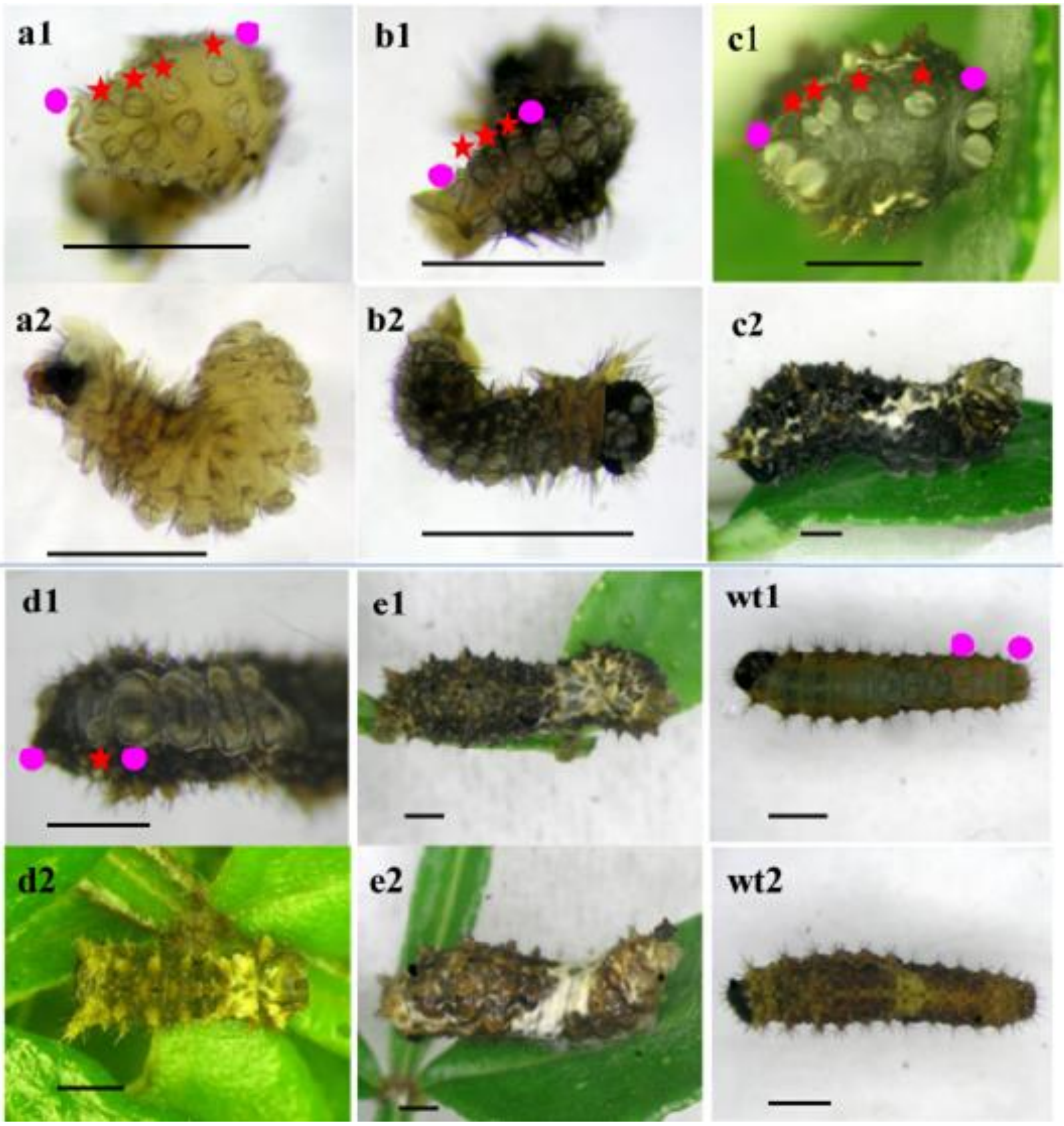

Supplementary Figure 43. Mutants with redundant prolegs and abnormal abdomen induced by Cas9-sgRNA injection of *Abd-B* gene in *P. xuthus*. a, Mutant with pairs of prolegs from abdominal segment 7 (A7) to A10 (**a1**) that do not exist in wild type (**wt1**), and its curling-up abdomen resulting from abnormal terga of A3 and thereafter (**a2**), different from **wt2**. **b**, Mutant with pairs of prolegs fromA) to A9 (**b1**) that do not exist in wild type (**wt1**), and its curling-up abdomen (**b2**). **c**, Mutant with prolegs in the right side of from A7 to A10 (**c1**) that do not exist in wild type (**wt1**), and its curling-up abdomen (**c2**). **d**, Mutant with a proleg in the left side of A7 (**d1**) that do not exist in wild type (**wt1**), and its curling-up abdomen (**d2**). **e**, Mutant with severely abnormal terga of A4 and thereafter (**e1**) even in the case of normal prologs as wild type (**wt1**), and its curling-up abdomen (**e2). wt:** wild type showing its normal prolegs **(wt1)** and normal terga **(wt2).** Red star showing the redundant prolegs; pink circle showing normal legs of A6 and A10. Scale bars: 1 mm.

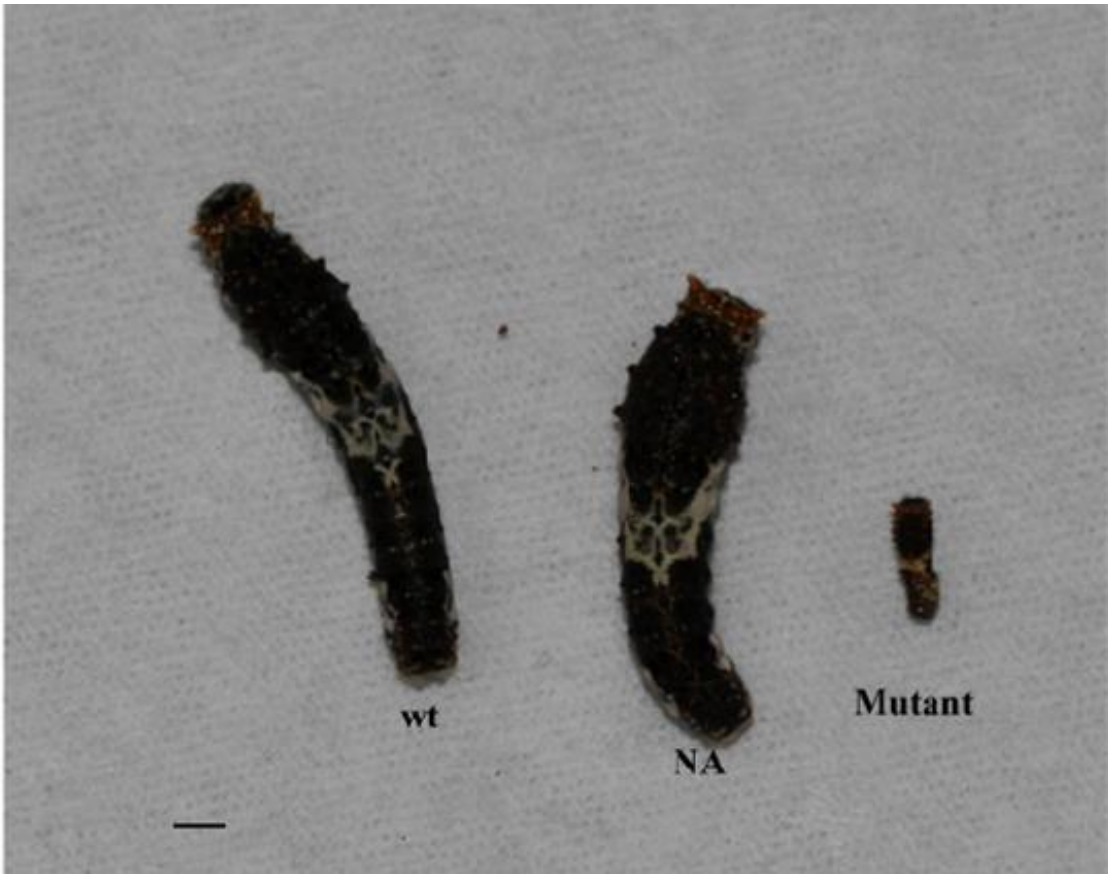

Supplementary Figure 44. Mutant induced by Cas9-sgRNA injection of *Abd-B* gene in *P. xuthus* showing delayed development compared with the larvae of wild type and no morphological mutation, hatched on the same day. wt: wild type; NA: injected larvae with no morphological mutation. Mutant: the type with prolegs in the right side of from A7 to A10 and with curling-up abdomen resulting from abnormal terga of A3 and thereafter. Scale bar: 1 mm.

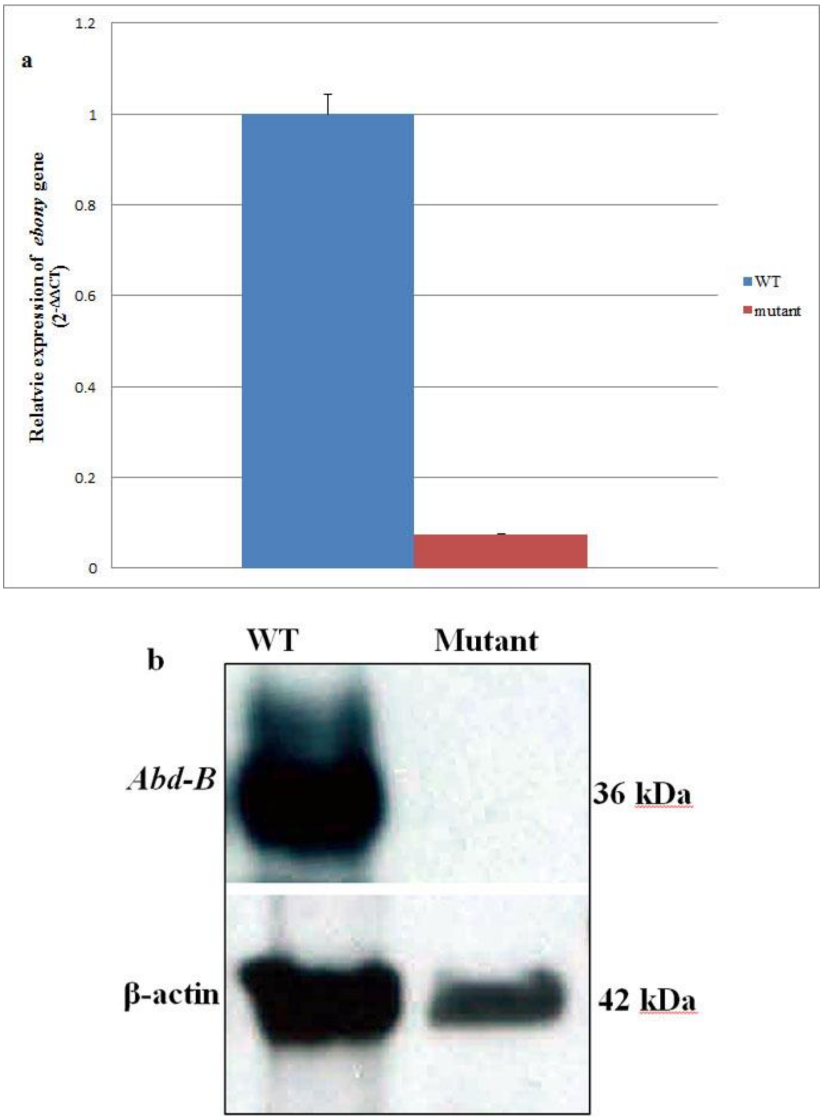

Supplementary Figure 45. Expression of *Abd-B* gene in mutants and in wild-type (WT) detected with quantitative reverse transcription-PCR (RT-PCR) and western blotting. a, Significant reduction of expression of *Abd-B* gene in *P. xuthus* mutants compared to WT individuals examined by RT-PCR (*t*-test, *P*=0.026). The maximum from the wild type was set as 1. Bars, means ± SD (n=3). x-axis: mutants and WT individuals; y-axis; the values of the relative expression ($2^{-\Delta\Delta CT}$) of *Abd-B*. **b**, The relative expression of *Abd-B* gene in 10 mutants and 10 WT individuals of *P. xuthus* examined by western blot using the fruitfly *Abd-B* antibody (1A2E9) (DSHB) and β-actin antibody (66009-1-Ig) (Proteintech) used as loading control.

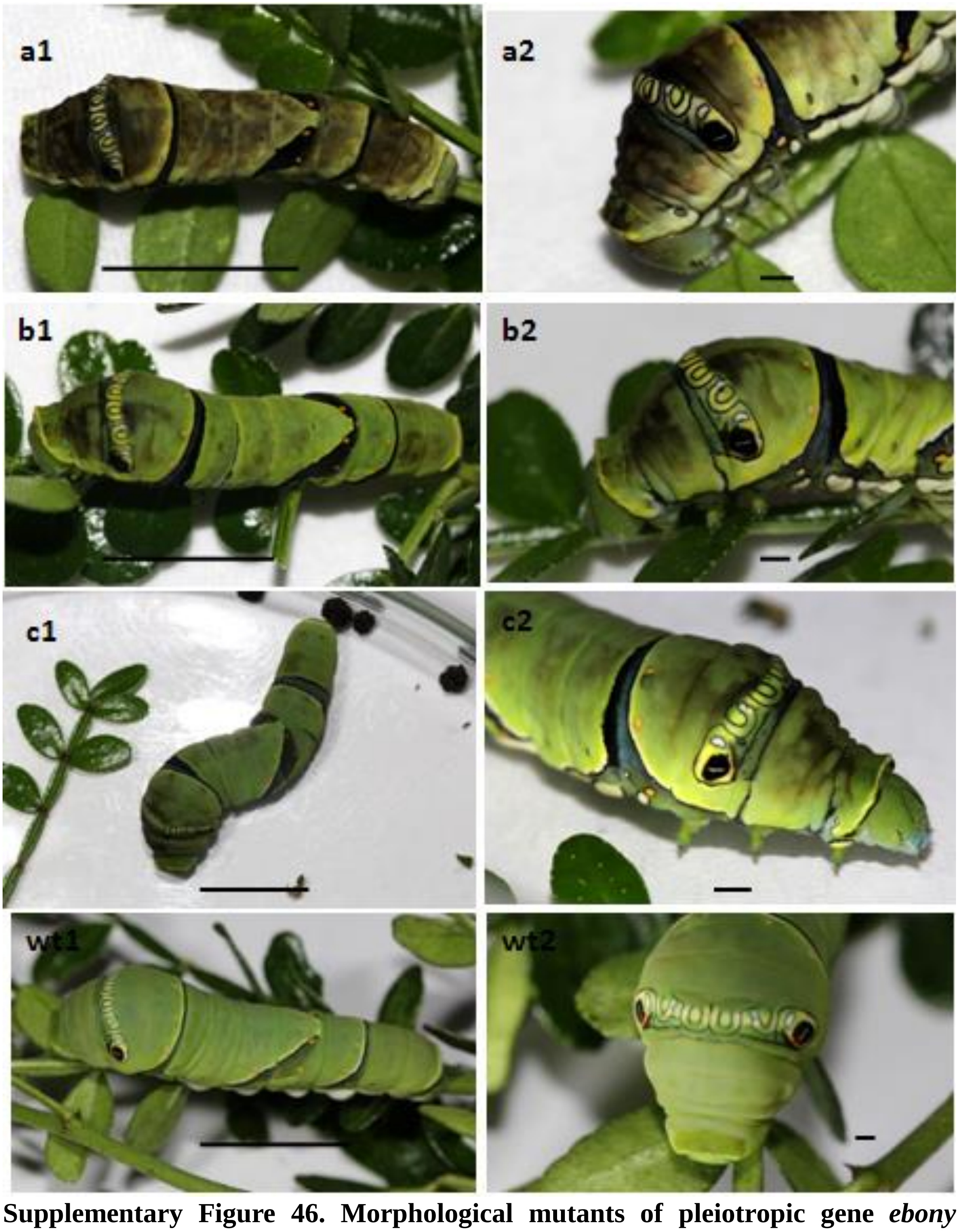

Supplementary Figure 46. Morphological mutants of pleiotropic gene *ebony* disruption induced by CRISPR/Cas9 system in *P. xuthus* larvae. **a**, Enhanced melanic pigmentation in whole dorsal side (**a1**) and an absence of orange color in both false eyes (**a2**). **b**, Enhanced melanic pigmentation in left half dorsal side (**b1**) and an absence of orange color in false eye of same side (**b2**). **c**, Enhanced melanic pigmentation in right half dorsal side (**c1**) and an absence of orange color in the false eyes of same side (**c2**). wt, wild type of whole body (**wt1**) and false eyes (**wt2**). Scale bars: 10 mm in panels **a1**, **b1**, **c1**, and **wt1**;1 mm in panels **a2**, **b2**, **c2** and **wt2**.

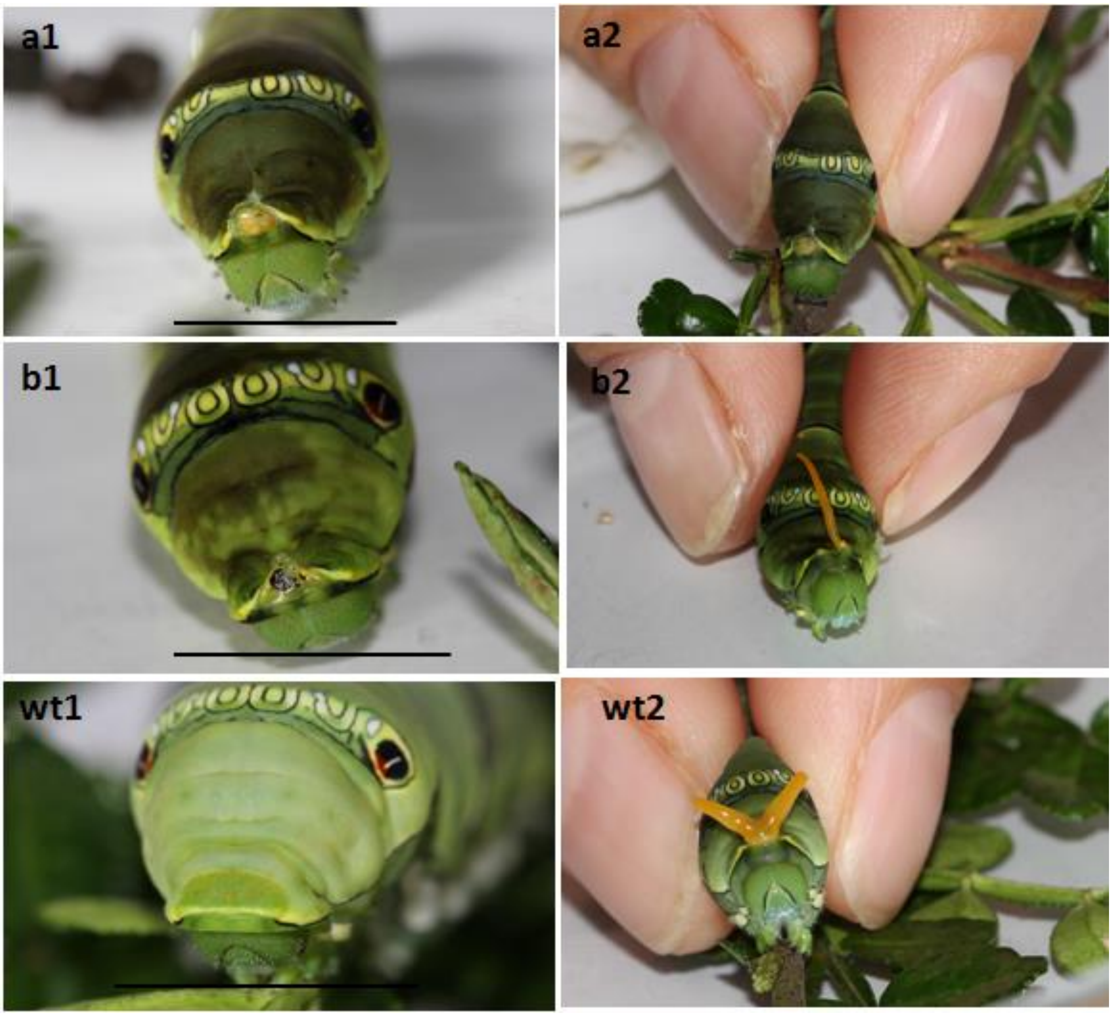

Supplementary Figure 47. Mutants of pleiotropic gene *ebony* disruption induced by CRISP/Cas9 system in *P. xuthus* larvae showing mutated ostemerium morphology (a1, b1) and behavior (a2, b2). wt: wild type. Scale bars: 10 mm

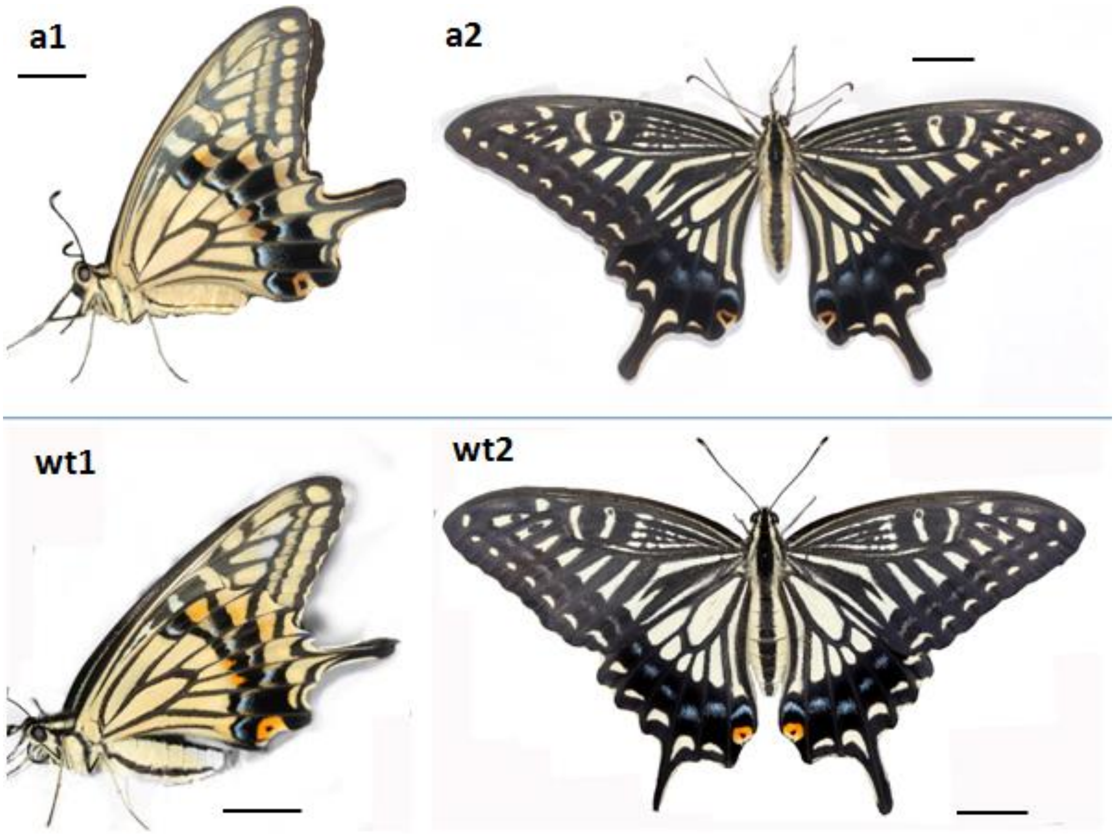

Supplementary Figure 48. ***P. xuthus*** **adult mutants of pleiotropic gene *ebony* induced by CRSISP/Cas9 system. a**, With brown pigmentation across the body (a1, a2) in regions that were normally yellow and in wing patches that were normally orange in adult (wt1, wt2). Scale bars: 1 mm

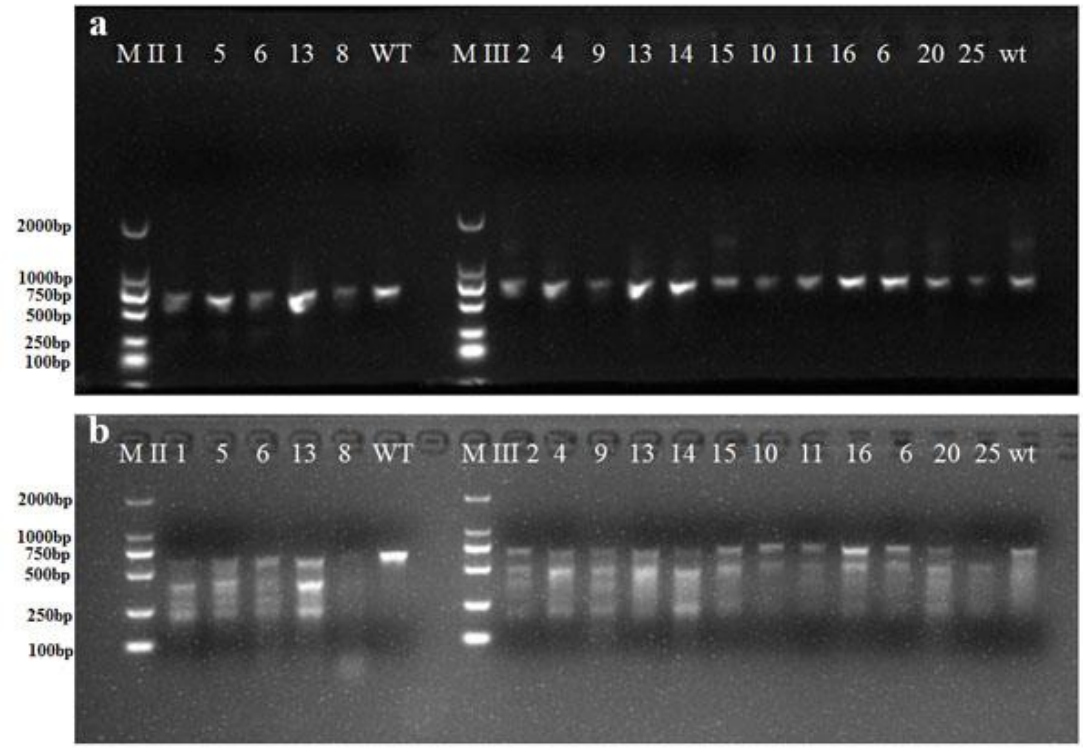

Supplementary Figure 49. Electrophoresis pictures of PCR (a) and T7EI (b) for most mutated individuals induced by the injecton of Cas9 mRNA and *ebony* sgRNA in *P. xuthus*. M: DL2000 DNA Marker. wt: wild type.

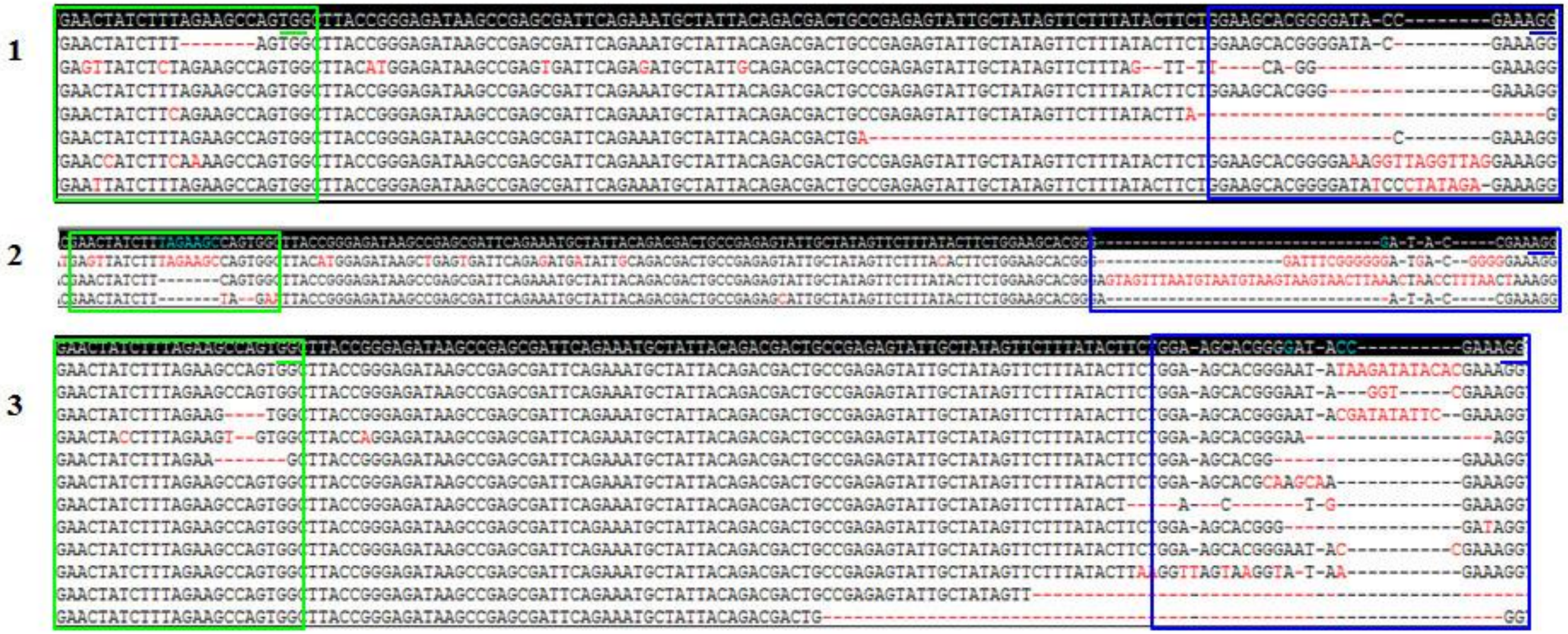

Supplementary Figure 50. Sequencing the target sites (T454, T6) of *ebony* mutants of different types in *P. xuthus* (*Px*). The first line for each alignment represents wild-type sequence, and subsequent lines show individual mutant clones. The T454 target sites are in the green line square, and the T6 target sites are in the blue line square. PAMs are underlined. 1, 2, 3 correspondent to the phenotypes **a**, **b** and **c** of **Supplementary Figure 46**.

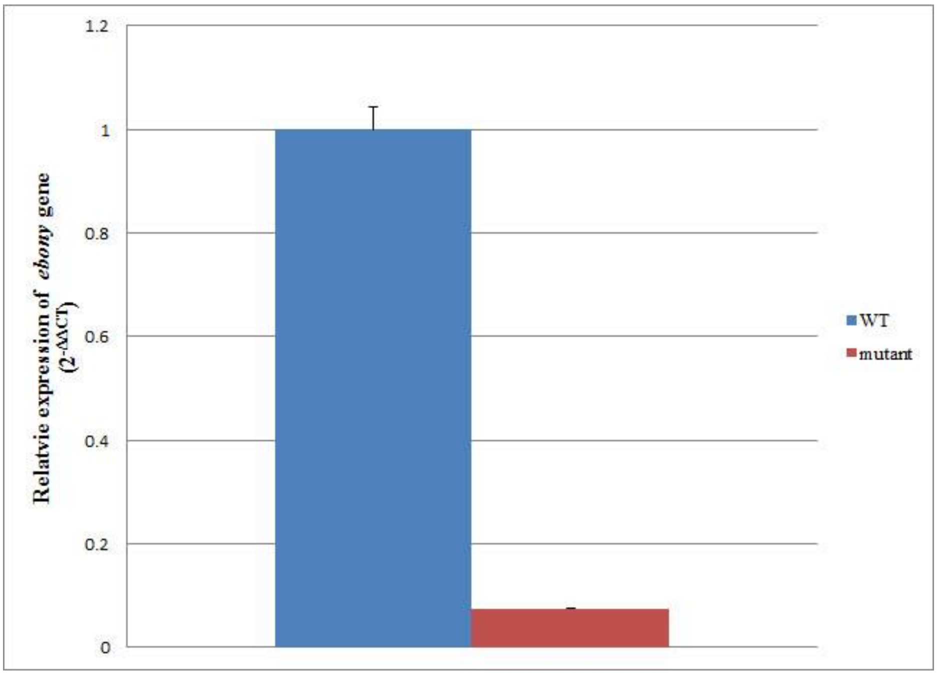

Supplementary Figure 51. Significant reduction of expression of *ebony* gene in *ebony* mutants of injection treatment III compared to the wide type of *P. xuthus* fifth instar larvae examined by quantitative reverse transcription-PCR (RT-PCR) (*t*-test, *P*=0.001)**.** The maximum from wild type was set as 1. Bars, means ± SD (n=3)**.** x-axis: mutants and wild type (WT) individuals; y-axis; the values of the relative expression ($2^{-\Delta\Delta CT}$) of *ebony*.

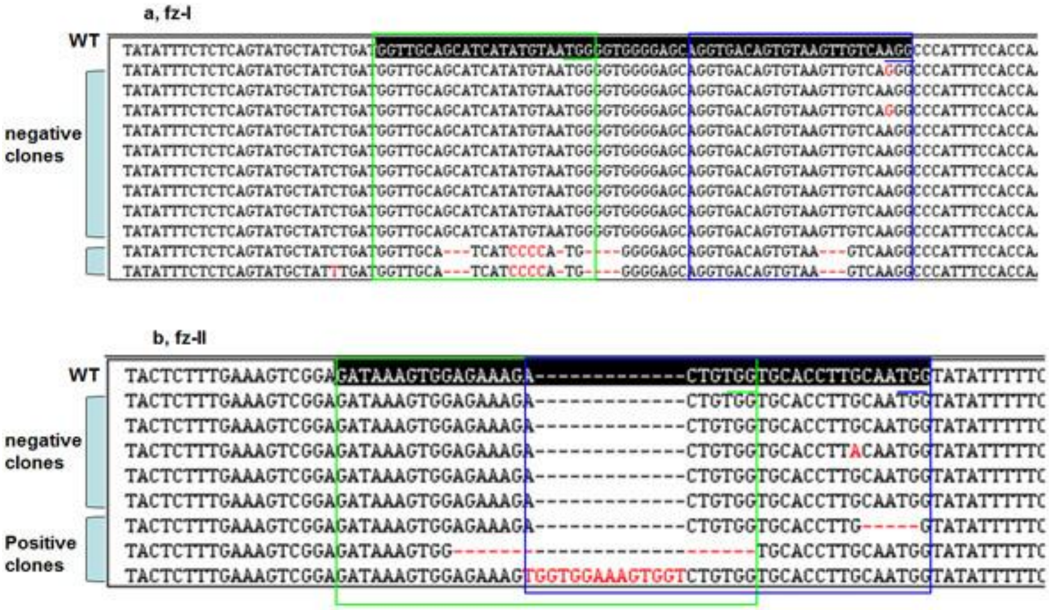

Supplementary Figure 52. Sequencing of induced mutations in I (a) and II (b) of *frizzled* by extracting DNA from 10 in *P. xuthus* mixed unhatched larvae after injection. The first line of the alignment represents wild-type (WT) sequence, and subsequent lines show individual mutant clones. PAMs are underlined. **a**, Injection I (co-injection of sgRNA targeting T432, T474, T508); The T474 target sites are in the green line square, and the T508 target sites are in the blue line square. Target site T432 without disruption not shown here. **b**, Injection II (co-injection of sgRNG targeting T268, T283); The target site T268 is in the green line square, and the target site T283 is in the blue line square.

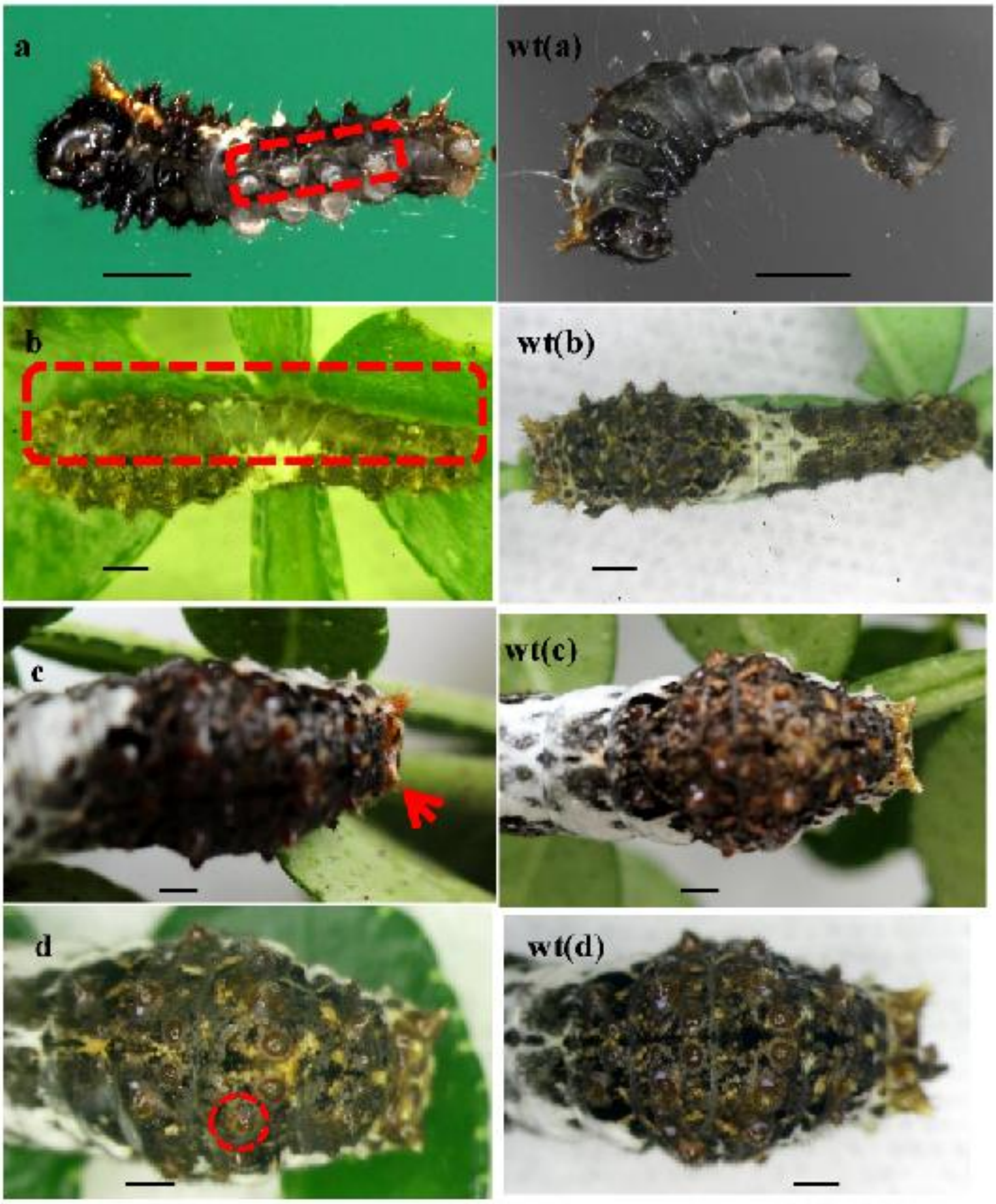

Supplementary Figure 53. Morphological mutants of planar cell polarity (PCP) gene *frizzled* induced by CRISPR/Cas9 system in *P. xuthus*. **a**, Mutated 2nd larva with prolegs of left side smaller than those of right side (**a**, shown in red square), compared with its wild type (**wt(a)**). **b**, Mutated 3rd larva with smoothy and colorless dorsal cuticle in right side (**b,** shown in red square) compared with its wild type (**wt(b)**). **c**, Mutated 4th larva with vestigial tubercle of right prothorax (**c**, shown by red arrow), compared with its wild type (**wt(c)**). **d**, Mutated 4th larva with vestigial tubercle of right metathorax (**d**, shown in red circle), compared with its wild type (wt(d)). Scale bars: 1 mm

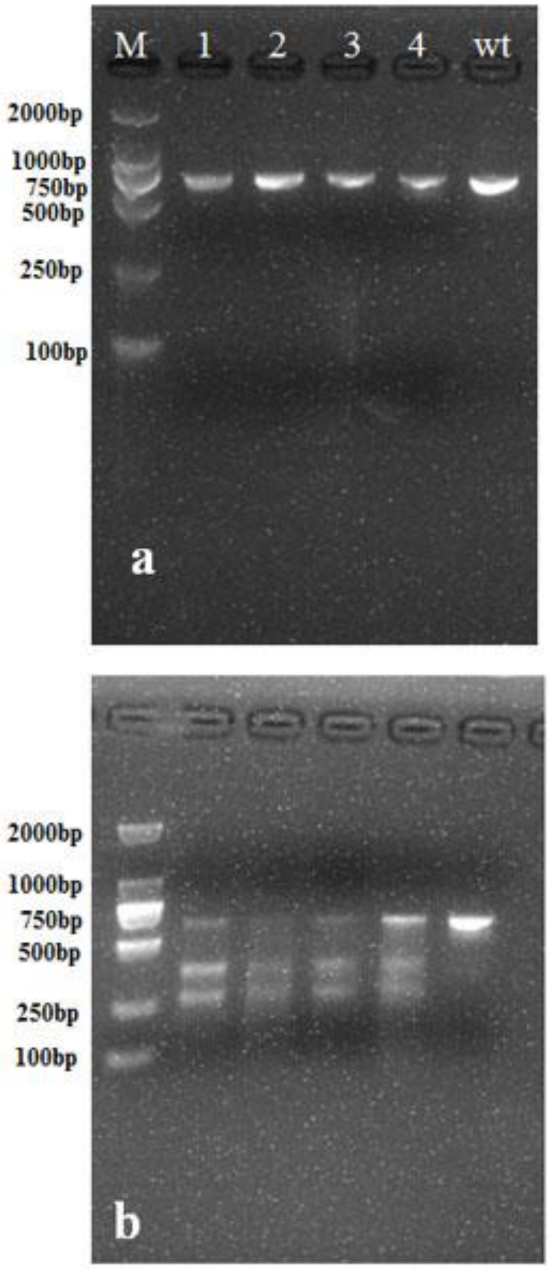

Supplementary Figure 54. Electrophoresis pictures of PCR (a) and T7EI (b) for all mutated individuals after *frizzled* knockout in *P. xuthus*. M: DL2000 DNA Marker. wt: wild type.

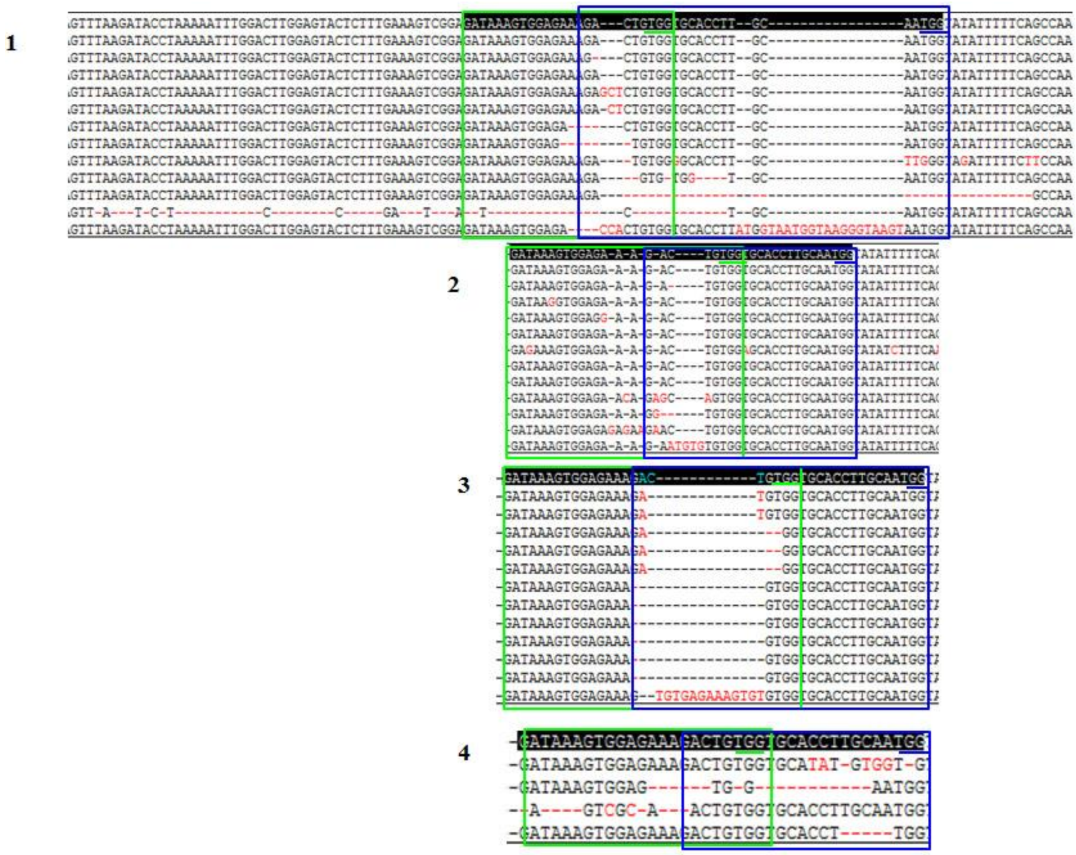

Supplementary Figure 55. Sequencing of induced mutations of different mutated *P. xuthus* (*Px*) phenotypes in the *frizzled* gene by coinjection of two sgRNA targeting two sites (T268, T283). The first line for each alignment represents wild-type sequence, and subsequent lines show individual mutant clones. The target site T268 is in the green line square, and the target site T283 is in the blue line square. PAMs are underlined.1, 2, 3, 4 correspondent to the phenotypes **a**, **b**, **c** and **d** in **Supplementary Figure 53**.

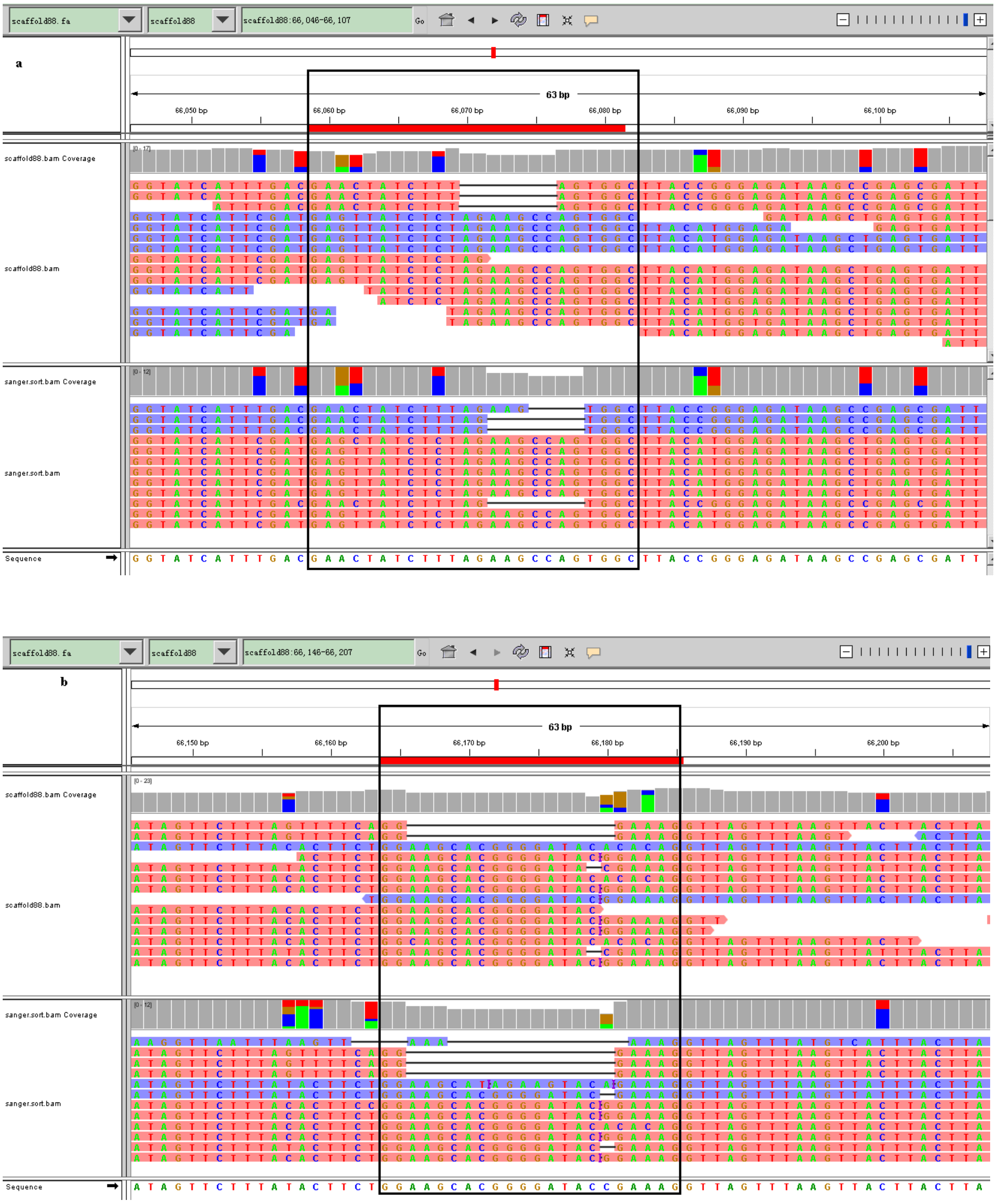

Supplementary Figure 56. The disruption of target sites (T454, T6) verified by both Sanger sequencing and whole genome sequencing in *ebony* mutant of Px_10703_e-III microinjection (co-injection of T454 and T6). a, T454; **b**, T6.

Supplementary Table 1. Comparison of original assemblies by different methods.

		Short reads contig		454 long reads contig		Hybrid contig		Hybrid Scaffold	
		**Length (bp)**	**Number**	**Length (bp)**	**Number**	**Length (bp)**	**Number**	**Length (bp)**	**Number**
Pm	N90	164	326,786	508	145,638	674	38,593	710	29,284
	N80	268	219,859	787	102,700	1,693	9,142	3,298	2,029
	N70	410	153,010	1,101	73,525	17,192	2,588	172,893	215
	N60	576	107,425	1,471	52,146	39,792	1,492	497,473	117
	**N50**	**777**	**74,226**	**1,939**	**36,008**	**66,647**	**926**	**915,272**	**71**
	N40	1,028	49,388	2,543	23,718	102,294	572	1,434,404	44
	N30	1,349	30,482	3,381	14,392	146,245	333	2,131,715	27
	N20	1,822	16,225	4,660	7,504	205,746	162	2,794,849	14
	N10	2,668	5,988	7,066	2,663	342,485	50	4,728,305	6
	Total length (bp)	221,658,236		271,747,604		293,984,005		308,164,783	
	Number>100 (bp)	500,090		240,247		127,305		119,876	
	Number>2000 (bp)	13,004		34,421		7,719		3,595	
	Max_length (bp)	16,556		47,532		1,593,795		6,904,646	
		**Length (bp)**	**Number**	**Length (bp)**	**Number**	**Length (bp)**	**Number**	**Length (bp)**	**Number**
Px	N90	136	437,823	1,009	44,701	19,402	791	20,457	466
	N80	188	305,500	2,092	29,718	122,555	405	1,062,051	60
	N70	282	214,865	3,210	21,259	230,930	265	1,862,359	42
	N60	386	152,109	4,373	15,365	335,222	180	2,722,284	31
	**N50**	**501**	**104,773**	**5,632**	**10,912**	**475,951**	**121**	**3,388,587**	**23**
	N40	656	68,292	7,122	7,417	672,284	79	4,148,284	16
	N30	884	40,828	9,117	4,669	861,767	49	5,008,671	11
	N20	1,242	20,788	11,907	2,536	1,108,347	25	6,848,192	6
	N10	1,971	7,139	16,971	950	2,190,346	10	8,040,173	3
	Total length (bp)	207,906,560		220,915,176		235,745,252		248,952,159	
	Number>100 (bp)	616,620		92,878		34,105		31,823	
	Number>2000 (bp)	6,934		30,658		2,708		1,569	
	Max_length (bp)	17,949		84,759		3,559,743		13,804,598	

Papilio xuthus (*Px*), *Papilio machaon* (*Pm*).

Supplementary Table 2. Summary of sequencing data from 454 and Illumina platforms.

Sequence platform	Species	Insert Size	Total base (Gb)	Read length			Coverage (×)
				Mean	Max	Min	
454（>500 bp）	*Px*	NA	2.40	754	1,596	500	9.6
	Pm	NA	2.61	727	1,596	500	8.4
Illumina	*Px*	150 bp	6.73	100_100			26.92
		250 bp	13.61	150_150			54.42
		500 bp	8.38	100_100			33.52
		2 Kb	4.3	50_50			20.66
		5 Kb	6.89	50_50			33.14
		10 Kb	3.88	50_50			15.5
		20 Kb	2.56	50_50			10.2
	**Total**	--	**46.35**	--			**194.36**
	Pm	150 bp	6.86	100_100			23.99
		250 bp	11.44	150_150			39.99
		500 bp	6.86	100_100			23.97
		2 Kb	6.04	50_50			21.13
		5 Kb	4.62	50_50			16.15
		10 Kb	4.37	50_50			15.28
		20 Kb	1.89	50_50			6.61
	**Total**	--	**42.08**	--			**140.51**

P. xuthus (*Px*), *P. machaon* (*Pm*).

Supplementary Table 3. *K-mer* frequency and genome size evaluation based on Illumina short reads from 250 bp and 500 bp libraries.

	Number of Kmer	Peak depth	Genome size	Used bases	*Used reads*	Coverage Genome (×)
Px	11,776,536,684	52	226,471,859	13,203,995,676	*89,216,187*	58.30
Pm	11,894,849,684	49	242,752,034	14,213,797,092	*144,934,213*	58.55

P. machaon (*Pm*): the first 100 bp of each 150 bp reads from the 250 bp library and the reads from the 500 bp library; *P. xuthus* (*Px*): all reads from 250 bp library.

Supplementary Table 4. Summary of simulating assembly by different read length in *P. xuthus* (*Px*).

**Average read length (bp)**	**225**		**377**		**754**	
	Length (bp)	Number	Length (bp)	Number	Length (bp)	Number
max_length:	59,192		66,025		84,759	
N90	303	234,318	867	51,023	1,009	44,701
N80	448	164,752	1,800	34,270	2,092	29,718
N70	618	115,733	2,711	24,611	3,210	21,259
N60	840	79,866	3,660	17,796	4,373	15,365
**N50**	**1,142**	**53,456**	**4,723**	**12,627**	**5,632**	**10,912**
N40	1,565	34,121	5,971	8,572	7,122	7,417
N30	2,173	20,103	7,631	5,380	9,117	4,669
N20	3,131	10,120	10,104	2,912	11,907	2,536
N10	4,967	3,428	14,293	1,091	16,971	950
Total_length	257,346,195		214,801,880		220,915,176	
number>100 (bp)	357,236		112,158		92,878	
**number>2000 (bp)**	23,231		31,803		30,658	

The coverage is 20 × for 225 bp reads, and 10 × for 377 bp and 754 bp long reads.

Supplementary Table 5. Genome size estimation of *Pm* and *Px* by flow-cytometry.

**In-ctrl**	***Pm*(Mb) (ave.± s.d.)**			***Px* (Mb) (ave.± s.d.)**			**Ex-ctrl** (Mb) (ave.± s.d.) (n=10)
	(males, females) (n=10)	(females) (n=5)	(males) (n=5)	(males, females) (n=10)	(females) (n=5)	(males) (n=5)	
Dm	**234.39±3.51**	235.43±4.23	233.34±2.67	**218.39± 3.21**	217.00±2.29	219.78±3.63	Gd: 1119.95±18.56
Gd	**256.58±5.77**	257.58±3.24	255.48±7.86	**238.83±5.07**	241.55±3.91	236.10±4.90	Dm: 194.95±3.14

P. xuthus (*Px*); *P. machaon* (*Pm*); *Drosophila melanogaster* (*Dm*); *Gallus domestica* (*Gd*).

Supplementary Table 6. Comparisons of original and final assemblies.

		Original assembly	Final assembly	Redundant haplotype scaffold	Microsporidium scaffold
Pm	Bases in scaffold (Mb)	308	**281**	15.6	13.5
	Bases in contig (Mb)	294	**265**		
	N50 scaffold size (Mb)	0.915	**1.15**		
	N50 contig size (Kb)	66	**81**		
Px	Bases in scaffold (Mb)	249	**244**	4.9	0
	Bases in contig (Mb)	236	**231**		
	N50 scaffold size (Mb)	3.4	**3.4**		
	N50 contig size (Kb)	475	**492**		

Final assemblies were obtained after haplotype separation and exclusion of mirosporidium genomes from original assemblies. *P. xuthus* (*Px*), *P. machaon* (*Pm*).

Supplementary Table 7. Summary of final assemblies after removing contaminations and redundant segments.

	Pm				***Px***			
	scaffolds		contigs		scaffolds		contigs	
	Length (bp)	Number	Length (bp)	Number	Length (bp)	Number	Length (bp)	Number
N10	5,275,587	5	375,409	43	8,040,115	3	2,190,270	10
N20	3,511,768	12	221,477	138	6,848,045	6	1,160,060	24
N30	2,320,244	23	158,812	282	5,125,173	10	868,950	47
N40	1,756,030	37	117,996	478	4,147,939	16	703,866	76
**N50**	**1,150,171**	**57**	**81,678**	**751**	**3,432,602**	**22**	**491,654**	**116**
N60	703,262	89	54,287	1,157	2,742,722	30	349,979	171
N70	378,332	144	30,505	1,818	2,042,228	40	242,524	251
N80	22,289	409	7,985	3,345	1,209,323	56	137,437	375
N90	1,005	11,681	936	18,511	21,263	252	45,887	644
**Total_length**	**280,641,486**		**268,231,573**		**244,040,124**		**230,833,217**	
**Number≧100 (bp)**	**78,453**	**84,295**			**20,895**	**23,177**		
**Number≧2000 (bp)**	**3,098**	**6,593**			**1,555**	**2,695**		
**max_len**	**6,905,436**		**1,593,921**		**13,804,215**		**3,559,395**	

P. xuthus (*Px*), *P. machaon* (*Pm*).

Supplementary Table 8. Summary of aligning short reads to assemblies.

	Pm		***Px***	
	**BWA**	**BWA+LASTZ**	**BWA**	**BWA+LASTZ**
**Total reads**	145,513,902	145,513,902	90,289,785	90,289,785
**Map ratio**	88.39%	96.87%	95.82%	97.33%
**Coverage of genome**	95.12%	95.35%	98.04%	98.16%

For *P. machaon* (*Pm*), the Illumina reads were split to 50 bp when aligned by BWA; for *P. xuthus* (*Px*), the Illumina reads length is 100 bp.

Supplementary Table 9. Evaluate assembly completeness by transcripts.

	**Transcript length (bp)**	**Total Number**	**Total aligned**		**>50% of Transcripts aligned**		**>90% of Transcripts aligned**	
			**Number**	**%**	**Number**	**%**	**Number**	**%**
Pm	>500	21,787	21,721	99.70	21,622	99.24	21,175	97.19
	>1000	12,205	12,188	99.86	12,158	99.61	12,009	98.39
Px	>500	21,941	21,783	99.28	21,761	99.18	21,659	98.71
	>1000	12,388	12,342	99.63	12,340	99.61	12,311	99.38

P. xuthus (*Px*), *P. machaon* (*Pm*).

Supplementary Table 10. Evaluate assembly completeness by CEG mapping approach.

Species		#Prots	%Completeness	#Total	Average	%Ortho
Pm	Complete	227	91.53	327	1.44	38.77
	Partial	240	96.77	431	1.8	52.92
Px	Complete	236	95.16	262	1.11	9.32
	Partial	245	98.79	316	1.29	22.86
Am	Complete	234	94.35	249	1.06	5.98
	partial	246	99.19	267	1.09	7.72
Ag	Complete	246	99.19	272	1.11	8.54
	Partial	247	99.6	277	1.12	9.72

Statistics of the completeness of the genome based on 248 CEGs (Core Eukaryotic Genes). Prots = number of 248 ultra-conserved CEGs present in genome; %Completeness = percentage of 248 ultra-conserved CEGs present; Total = total number of CEGs present including putative orthologs; Average = average number of orthologs per CEG; %Ortho = percentage of detected CEGS that have more than 1 ortholog; A catalog of completeness on published genomes are listed in http://korflab.ucdavis.edu/Datasets/genome_completeness/. *P. xuthus* (*Px*), *P. machaon* (*Pm*); *Apis mellifera* (*Am*); *Anopheles gambiae* (*Ag*).

Supplementary Table 11. Alignment of GeneWise predicted genes between CEGs.

Species	Genewise predicted protein	Best hit protein in CEGs	Length of predicted protein	Length of CEG	Coverage of predicted protein	Coverage of CEG	Identity
Pm	KOG0361	7299092-KOG0361	543	501	0.83	0.96	63.19
	KOG2017	SPAC2G11.10c-KOG2017	440	401	0.52	0.63	34.12
	KOG0989	Hs4506491-KOG0989	288	363	0.97	0.78	54.39
	KOG1159	Hs7657393-KOG1159	623	597	0.83	0.86	41.03
	KOG2531	Hs18860918-KOG2531	552	536	0.34	0.35	61.26
	KOG2909	Hs4502315-KOG2909	442	382	0.6	0.61	48.75
	KOG0346	7295505-KOG0346	594	560	0.84	0.87	53.75
Px	KOG1159	7295098-KOG1159	597	582	0.74	0.74	42.02
	KOG0346	7295505-KOG0346	594	560	0.85	0.87	56.21

The genes that cannot be annotated by CEGMA in *P. xuthus* (*Px*) and *P. machaon* (*Pm*) assemblies are predicted by Genewise. And the proteins are aligned by BLASTP. Finally, *Pm* and *Px* assemblies contain 247 and 248 (99.60%) CEGs, respectively.

Supplementary Table 12. Comparison of animal reference genome assemblies.

Species	Taxonomic status (phylum, class, order, family)	Genome size	Contig N50	Scaffold N50	Refences
Papilio xuthus	**Arthropod, Insecta, Lepidotera, Papiliodidae**	**244 M**	**492 K**	**3.4 M**	**This study**
Drosophila erecta	Arthropod, Insecta, Diptera, Drosophilidae	153 M	448 K	N A	[15]
Drosophila melanogaster	Arthropod, Insecta, Diptera, Drosophilidae	180 M	NA	NA	[15, 16]
Adineta vaga	Aschelminthes, Rotifera	244 M	218 K	NA	[17]
Tetranychus urticae	Arthropod, Arachnida,	90 M	212.8 K	3.0 M	[18]
Trichoplax adhaerens	Placozoa	104 M	204.2 K	5.79 M	[19]
Drosophila willistoni	Arthropod, Insecta, Diptera, Drosophilidae	236 M	197 K	NA	[15]
Canis familiaris	Chordata, mammal,Canidae	2.5 G	180 K	45 M	[20]
Petromyzon marinus	Chordata, fish	816 M	174 K	NA	[21]
Homo sapien	Chordata, mammal,	3 G	NA	NA	[22]
Drosophila virilis	Arthropod, Insecta, Diptera, Drosophilidae	206 M	136 K	NA	[15]
Drosophila mojavensis	Arthropod, Insecta, Diptera,Drosophilidae	194 M	132 K	NA	[15]
Papilio xuthus	**Arthropod, Insecta, Lepidoptera, Papiliodidae**	244 M	128 K	6.2 M	[23]
Drosophila yakuba	Arthropod, Insecta,Diptera,Drosophilidae	166 M	125 K	NA	[15]
Drosophila grimshawi	Arthropod, Insecta,Diptera,Drosophilidae	201 M	114 K	NA	[15]
Drosophila ananassae	Arthropod, Insecta,Diptera,Drosophilidae	231 M	113 K	NA	[15]
Monodelphis domestica	Chordata, mammal,Didelphidae	3.4 G	108 K	59.8 M	[24]
Belgica antarctica	Arthropod, Insecta,Diptera,Chironomidae	99 M	98.2 K	NA	[25]
Parus humilis	Chordata,Aves, Paridae	1.08 G	88.3 K	1.58 M	[26]
Gasterosteus aculeatus	Chordata, fish	463 M	83.2 K	10.8 M	[27]
Aedes aegypti	Arthropod, Insecta,Diptera,Culicidae	1376 M	82.6 K	1.5 M	[28]
Papilio machaon	**Arthropod, Insecta, Lepidoptera, Papiliodidae**	**281 M**	**82 K**	**1.5 M**	**This study**
Sus scrofa	Chordata, mammal,Suidae	2.6 G	80.7 K	637.3 K	[29]
Anolis carolinensis	Chordata, reptile	1.78 G	79.9 K	4 M	[30]
Trichinella spiralis	Nematoda,	64 M	76.6 K	1,74 M	[31]
Heliconius melpomene	**Arthropod, Insecta, Lepidoptera, Nymphidae**	**269 M**	**51 K**	**212 K**	**[32]**
Danaus pleppus	**Arthropod, Insecta, Lepidoptera, Nymphidae**	**273 M**	**50 K**	**207 K**	**[33]**
Papilio polytes	**Arthropod, Insecta, Lepidoptera, Papiliodidae**	**227 M**	**47 K**	**3.7 M**	**[23]**
Papilio glaucas	**Arthropod, Insecta, Lepidoptera, Papiliodidae**	**376 M**	**NA**	**230 K**	**[34]**
Plutella xylostella	Arthropod, Insecta, Lepidoptera, Nymphidae	394 M	49 K	737 K	[35]
Apis mellifera	Arthropod, Insecta,	236 M	41 K	362 K	[36]
Tribolium castaneum	Arthropod, Insecta,	204 M	41 K	992 K	[37]
Caenorhabditis briggsae	Nematoda,	104 M	41 K	474 K	[38]
Rattus norvegicus	Chordates, Rodentia, Muridae	2.75 G	38 K	5.3 M	[39]
Trypanosoma cruzi	Euglenozoa	67 M	26 K	151 K	[40]
Branchiostoma floridae	Chordates, Leptocardii	520 M	25.6 K	1.6 M	[41]
Mus musculus	Chordates, Rodentia, Muridae	2.5 G	24.8 K	16.9 M	[42]
Ciona intestinalis	Chordata, Ascidiacea	150 M	20 K	190 K	[43]
termite genome	Arthropod, Insecta,Blattaria	493 M	20 K	740 K	[44]
Nematostella vectensis	Cnidaria,Actinozoa	357 M	19.8 K	472 K	[45]
Crassostrea gigas	Mollusca,Ostreidae	559 M	19.4 K	401 K	[46]
Xenopus tropicalis	Chordata, Amphibian	1.7 G	17.0 K	1.6 M	[47]
Melitaea cinxia	**Arthropod, Insecta, Lepidoptera, Nymphidae**	393 M	13 K	119 K	[48]
Bombyx mori	Arthropod, insect, Lepidoptera	429 M	12.5 K	26.9 K	[49]
Amphimedon queenslandica	Porifera	190 M	11.2 K	120 K	[50]
Acyrthosiphon pisum.	Arthropod, Insecta,	517 M	10.8 K	88.5 K	[51]
Locusta migratoria	Arthropod, Insecta,	6.5 G	9.3 K	320.3 K	[52]
Strongylocentrotus purpuratus	Echinodermata,Echinoidea	1 G	9 K	65 K	[53]

Daphnia pulex	Arthropod, Crustacea	200 M	1.17 K	319 K	54

Including all animals with contig N50>82K; for animal genome with their contig N50<82K, we include those of all Lepidoptera (Bold highlightened), the largest taxa in other orders of insecta, the largest taxa in other class of Arthropod, the largest in the class of Chordata, the largest taxa in other phyla.In addition, we also delt with those of a few classic amimals including fruitfly *Drosophila melanogaster*, human *Homo sapiens*, mouse *Mus musculus* and rat *Rattus norvegicus*. NA, the number not applicable in the references. K, Kilobase; M: megabase; G: Gigabase.

Supplementary Table 13. The statistics of RAD sequencing data for *P. xuthus* (*Px*).

Sample ID	Count (100 bp read pairs)	Depth (×)	Sample ID	Count (100 bp read pairs)	Depth (×)
s1	2,968,037	26.98	s51	1,501,675	13.65
s2	1,964,367	17.86	s52	1,649,108	14.99
s3	1,470,643	13.37	s53	1,975,694	17.96
s4	1,124,790	10.23	s54	1,640,999	14.92
s5	1,963,942	17.85	s55	1,135,403	10.32
s6	2,888,905	26.26	s56	608,332	5.53
s7	2,714,996	24.68	s57	1,324,242	12.04
s8	1,348,508	12.26	s58	1,621,192	14.74
s9	2,232,906	20.30	s59	1,709,838	15.54
s10	2,038,899	18.54	s60	1,672,420	15.20
s11	1,449,049	13.17	s61	2,207,411	20.07
s12	1,618,272	14.71	s62	2,853,393	25.94
s13	2,168,289	19.71	s63	2,233,613	20.31
s14	3,230,823	29.37	s64	897,897	8.16
s15	1,174,367	10.68	s65	1,707,447	15.52
s16	989,556	9.00	s66	229,641	2.09
s17	2,828,492	25.71	s67	1,466,505	13.33
s18	2,315,779	21.05	s68	790,412	7.19
s19	1,203,314	10.94	s69	2,493,179	22.67
s20	1,932,050	17.56	s70	1,095,384	9.96
s21	3,043,494	27.67	s71	1,608,824	14.63
s22	2,240,386	20.37	s72	765,121	6.96
s23	2,672,368	24.29	s73	625,237	5.68
s24	1,941,803	17.65	s74	605,475	5.50
s25	1,355,414	12.32	s75	457,480	4.16
s26	1,029,130	9.36	s76	822,170	7.47
s27	1,366,671	12.42	s77	653,476	5.94
s28	1,363,107	12.39	s78	834,114	7.58
s29	1,419,453	12.90	s79	499,898	4.54
s30	2,090,571	19.01	s80	245,474	2.23
s31	1,547,743	14.07	s81	321,158	2.92
s32	1,355,490	12.32	s82	244,326	2.22
s33	3,444,834	31.32	s83	414,598	3.77
s34	2,462,670	22.39	s84	384,578	3.50
s35	2,363,113	21.48	s85	477,598	4.34
s36	3,656,882	33.24	s86	1,601,536	14.56
s37	2,626,749	23.88	s87	279,975	2.55
s38	3,610,554	32.82	s88	118,409	1.08
s39	6,493,910	59.04	s89	1,100,671	10.01
s40	1,285,078	11.68	s90	339,055	3.08
s41	2,436,962	22.15	s91	309,639	2.81
s42	2,539,651	23.09	s92	133,884	1.22
s43	1,325,030	12.05	s93	268,458	2.44
s44	1,508,273	13.71	s94	1,046,653	9.52
s45	2,703,948	24.58	s95Mom	1,929,075	17.54
s46	2,646,418	24.06	s96Dad	876,102	7.96
s47	2,518,655	22.90	Discarded	1,367,693	
s48	582,764	5.30	**Total**	**154,821,324**	
s49	1,282,564	11.66	**EcoRI sites**: 55851		
s50	1,137,193	10.34	**RAD Tags**: ~110,000		

Supplementary Table 14. Comparison of repetitive elements in 4 butterfly genomes identified by different methods.

Software		*Pm*		*Px*		*Hm*		*Dp*	
		Length	%genome	Length	%genome	Length	%genome	Length	%genome
ProteinMask		4,876,342	1.74	2,014,236	0.83	7,769,084	2.84	6,484,623	2.37
RepeatMasker		9,031,340	3.22	4,264,963	1.75	5,967,395	2.18	5,503,014	2.01
TRF		4,992,615	1.78	3,185,468	1.31	3,691,701	1.35	3,341,702	1.22
Denovo	Ltr_finder	3,920,375	1.4	4,121,576	1.69	3,297,759	1.20	747,423	0.27
	Repeatscout	53,077,214	18.91	45,075,972	18.47	72,197,868	26.36	26,160,178	9.55
	Total	62,611,386	22.31	51,458,069	21.09	78,023,975	28.49	33,467,979	12.21

P. xuthus (*Px*), *P. machaon* (*Pm*), *H. melpomene* (*Hm*), *D. plexippus* (*Dp*).

Supplementary Table 15. The comparison of transposable elements (TEs) in 4 butterfly genomes using repeatmask.

	Pm		*Px*		*Dp*		*Hm*	
	Length	%genome	Length	%genome	Length	%genome	Length	%genome
**DNA**	12,637,440	4.50	3,390,512	1.39	5,145,839	1.88	4,392,809	1.60
**SINE**	9,330,930	3.32	4,665,461	1.91	345,229	0.13	3,773,105	1.38
**LINE**	1,994,463	0.71	825,075	0.34	7,242,911	2.64	11,384,723	4.15
**LTR**	3,971,695	1.42	7,495,075	3.07	1,234,598	0.45	2,907,435	1.06
**Satellite**	150,589	0.05	0	0.00	--	--	5,497	0.002
**Simple**	62,354	0.02	94,499	0.04	87,221	0.03	147,018	0.05
**UnKnown**	32,678,728	11.64	35,160,946	14.41	18,794,537	6.85	59,168,444	21.60
**Total**	56,604,249	20.17	46,773,790	19.17	33,467,979	12.21	78,023,975	28.47

P. xuthus (*Px*), *P. machaon* (*Pm*), *H. melpomene* (*Hm*), *D. plexippus* (*Dp*).

Supplementary Table 16. Top 11 TE copy number of *Pm* and comparison in four butterflies using RepeatMasker.

TE_Class	*Pm*			*Px*			*Hm*			*Dp*		
	Copy Number	#Base	%genome	Copy Number	#Base	%genome	Copy Number	#Base	%genome	Copy Number	#Base	%genome
**DNA/Helitron**	31,883	3,720,833	1.21	8,348	867,887	0.35	857	188,765	0.07	2,747	440,621	0.16
**SINE/SINE**	6,076	642,099	0.21	5,021	505,504	0.20	3,296	311,291	0.11	242	25,734	0.01
**DNA/TcMar**	4,711	1,230,256	0.40	891	122,060	0.05	2,792	478,563	0.17	4,502	1479497	0.54
**LINE/RTE**	4,546	849,816	0.28	2,322	476,144	0.19	1,772	751,281	0.27	1,251	428,168	0.16
**DNA/hAT**	3,093	365,868	0.12	3,112	186,928	0.08	2,535	203,584	0.07	4,163	337,915	0.12
**LTR/Gypsy**	2,529	457,641	0.15	1,509	217,199	0.09	3,647	526,976	0.19	3,281	478,868	0.17
**LTR/Pao**	1,792	276,424	0.09	1,111	110,557	0.04	2,639	230,438	0.08	426	38,001	0.01
**DNA/DNA**	1,739	210,567	0.07	2,284	232,503	0.09	1,205	91,997	0.03	1,795	130,584	0.05
**LTR/Copia**	1,120	179,561	0.06	945	77,572	0.03	858	84,163	0.03	1,060	134,828	0.05
**LINE/CR1**	1,058	264,182	0.09	808	192,163	0.08	1,437	479,073	0.17	2,998	366,181	0.13
**LINE/L2**	1,021	146,777	0.05	546	79,067	0.03	4,975	1,218,340	0.44	2,107	240,212	0.09

P. xuthus (*Px*), *P. machaon* (*Pm*), *H. melpomene* (*Hm*), *D. plexippus* (*Dp*).

Supplementary Table 17. Summary of parameters for annotated genes in four butterflies.

Species	Number	Complete ORF (%)	Single exon gene (%)	Average gene length	Average CDS length	Average exon number	Average exon length	Average intron length
Pm	**15,499**	**97.61**	**15.08**	**5,362**	**1,360**	**6.27**	**217**	**760**
Px	**15,322**	**98.41**	**14.93**	**5,322**	**1,379**	**6.45**	**214**	**723**
Hm	15,984	98.29	17.44	5,607	1,290	6.07	213	852
Dp	16,866	97.17	14.39	4,998	1,238	5.89	210	769
Bombyx mori	14,623	98.63	15.46	6,030	1,224	5.44	225	1082

P. xuthus (*Px*), *P. machaon* (*Pm*), *H. melpomene* (*Hm*), *D. plexippus* (*Dp*).

Supplementary Table 18. Resultsofgene function annotation.

Function class	*Dp*		*Hm*		*Pm*		*Px*	
	Number	Percetage (%)	Number	Percetage (%)	Number	Percetage (%)	Number	Percetage (%)
**Go**	8,562	50.76	8,597	53.79	8,617	55.60	7,499	48.94
**Ipr**	10,605	62.88	10,641	66.57	10,588	68.31	9,535	62.23
**Kegg**	5,494	32.57	6,136	38.39	5,127	33.08	6,484	42.32
**Swispport**	10,935	64.83	10,652	66.64	9,930	64.07	9,664	63.07
**Tremble**	13,294	78.82	12,716	79.55	12,558	81.02	11,047	72.10
**Total**	13,515	80.13	12,960	81.08	13,119	84.64	12,598	82.22

P. xuthus (*Px*), *P. machaon* (*Pm*), *H. melpomene* (*Hm*), *D. plexippus* (*Dp*).

Supplementary Table 19. Summary of gene families in 11 insects.

Species	#Total genes (>50aa)	#Unclustered genes	#Families	#Unique families (gene number)	Ave. genes per family
Ag	12,637	2,040	7,654	247	1.38
Am	10,561	1,967	7,837	134	1.1
Bm	14,592	1,818	10,090	101	1.27
Dp	16,590	1,875	10,952	230	1.34
Dm	13,647	2,874	7,514	291	1.43
Hm	15,914	1,775	10,676	137	1.32
PLX	18,073	3,078	8,938	395	1.68
Pm	15,393	1,510	10,988	54	1.26
Px	15,237	1,548	10,797	42	1.27
Tc	16,460	4,348	8,262	371	1.47
Mc	16,537	4,616	9,655	97	1.23

Including five butterflies: *P. xuthus* (*Px*), *P. machaon* (*Pm*), *H. melpomene* (*Hm*), *D. plexippus* (*Dp*), *Melitaea cinxia* (*Mc*); 2 moths: *Bombyx mori* (*Bm*), *Plutella xylostella* (*PLX*); 1 fruitfly: *D. melanogaster* (*Dm*); 1 mosquito: *A. gambiae* (*Ag*); 1 beetle: *Tribolium castaneum* (*Tc*); 1 bee : *A. mellifera* (*Am*).

Supplementary Table 20. Gene Ontology (GO) enrichment of 107 butterfly-specific gene families.

GO_ID	GO_Term	GO_Class	Pvalue (FDR test)	EnrichDirect
GO:0007050	cell cycle arrest	BP	1.82E-02	Over
GO:0045786	negative regulation of cell cycle	BP	1.82E-02	Over
GO:0008150	biological_process	BP	2.96E-02	Under
GO:0051726	regulation of cell cycle	BP	3.31E-02	Over
GO:0009987	cellular process	BP	3.95E-02	Under
GO:0017176	phosphatidylinositol N-acetylglucosaminyltransferase activity	MF	3.06E-03	Over
GO:0016743	carboxyl- or carbamoyltransferase activity	MF	6.11E-03	Over
GO:0042302	structural constituent of cuticle	MF	8.44E-03	Over
GO:0004861	cyclin-dependent protein kinase inhibitor activity	MF	9.15E-03	Over
GO:0008375	acetylglucosaminyltransferase activity	MF	1.22E-02	Over
GO:0004860	protein kinase inhibitor activity	MF	1.22E-02	Over
GO:0019210	kinase inhibitor activity	MF	1.22E-02	Over
GO:0030291	protein serine/threonine kinase inhibitor activity	MF	1.22E-02	Over
GO:0016597	amino acid binding	MF	1.52E-02	Over
GO:0043176	amine binding	MF	1.52E-02	Over
GO:0016538	cyclin-dependent protein kinase regulator activity	MF	1.52E-02	Over
GO:0016741	transferase activity, transferring one-carbon groups	MF	2.57E-02	Over
GO:0008194	UDP-glycosyltransferase activity	MF	2.72E-02	Over
GO:0008173	RNA methyltransferase activity	MF	3.31E-02	Over
GO:0019887	protein kinase regulator activity	MF	4.20E-02	Over
GO:0019207	kinase regulator activity	MF	4.49E-02	Over

False Discovery Rate (FDR).

Supplementary Table 21. Gene Ontology (GO) enrichments of *Papilio*-specific gene families.

GO_ID	GO_Term	GO_Class	Pvalue (FDR test)	EnrichDirect
GO:0018149	peptide cross-linking	BP	7.95E-04	Over
GO:0042811	pheromone biosynthetic process	BP	7.41E-03	Over
GO:0010817	regulation of hormone levels	BP	7.41E-03	Over
GO:0019748	secondary metabolic process	BP	7.41E-03	Over
GO:0042445	hormone metabolic process	BP	7.41E-03	Over
GO:0042446	hormone biosynthetic process	BP	7.41E-03	Over
GO:0042810	pheromone metabolic process	BP	7.41E-03	Over
GO:0008283	cell proliferation	BP	2.21E-02	Over
GO:0000159	protein phosphatase type 2A complex	CC	2.21E-02	Over
GO:0008287	protein serine/threonine phosphatase complex	CC	2.21E-02	Over
GO:0003824	catalytic activity	MF	7.36E-03	Under
GO:0017056	structural constituent of nuclear pore	MF	7.41E-03	Over
GO:0008262	importin-alpha export receptor activity	MF	1.48E-02	Over
GO:0005049	nuclear export signal receptor activity	MF	1.48E-02	Over
GO:0008601	protein phosphatase type 2A regulator activity	MF	2.21E-02	Over
GO:0019888	protein phosphatase regulator activity	MF	2.93E-02	Over
GO:0019208	phosphatase regulator activity	MF	3.65E-02	Over
GO:0008603	cAMP-dependent protein kinase regulator activity	MF	4.36E-02	Over
GO:0030234	enzyme regulator activity	MF	4.78E-02	Over

False Discovery Rate (FDR).

Supplementary Table 22. Gene Ontology (GO) enrichments of those significant (*P*<0.01) expansion gene families.

Clades	GO_ID	GO_Term	GO_Class	Pvalue (FDR test)	EnrichDirect
Pm	GO:0004252	serine-type endopeptidase activity	MF	1.40E-43	Over
	GO:0070011	peptidase activity, acting on L-amino acid peptides	MF	2.22E-35	Over
	GO:0016787	hydrolase activity	MF	6.92E-32	Over
	GO:0003824	catalytic activity	MF	8.97E-15	Over
	GO:0004499	N,N-dimethylaniline monooxygenase activity	MF	7.20E-07	Over
	GO:0050661	NADP binding	MF	1.41E-05	Over
	GO:0004497	monooxygenase activity	MF	0.000107	Over
	GO:0008234	cysteine-type peptidase activity	MF	0.002694	Over
	GO:0004835	tubulin-tyrosine ligase activity	MF	0.003492	Over
	GO:0050660	flavin adenine dinucleotide binding	MF	0.020171	Over
	GO:0006508	proteolysis	BP	3.11E-34	Over
	GO:0019538	protein metabolic process	BP	1.20E-28	Over
	GO:0008152	metabolic process	BP	6.17E-11	Over
	GO:0044238	primary metabolic process	BP	1.66E-09	Over
	GO:0007602	phototransduction	BP	9.30E-08	Over
	GO:0007601	visual perception	BP	7.10E-06	Over
Px	GO:0070011	peptidase activity, acting on L-amino acid peptides	MF	3.11E-98	Over
	GO:0004252	serine-type endopeptidase activity	MF	1.24E-51	Over
	GO:0016787	hydrolase activity	MF	7.29E-35	Over
	GO:0003824	catalytic activity	MF	7.80E-23	Over
	GO:0004499	N,N-dimethylaniline monooxygenase activity	MF	1.18E-13	Over
	GO:0050661	NADP binding	MF	1.37E-11	Over
	GO:0004497	monooxygenase activity	MF	8.55E-10	Over
	GO:0008234	cysteine-type peptidase activity	MF	5.66E-06	Over
	GO:0004835	tubulin-tyrosine ligase activity	MF	1.95E-05	Over
	GO:0050660	flavin adenine dinucleotide binding	MF	6.62E-05	Over
	GO:0009055	electron carrier activity	MF	0.004122	Over
	GO:0020037	heme binding	MF	0.006233	Over
	GO:0016491	oxidoreductase activity	MF	0.00817	Over
	GO:0006508	proteolysis	BP	5.66E-92	Over
	GO:0019538	protein metabolic process	BP	1.54E-35	Over
	GO:0008152	metabolic process	BP	1.35E-14	Over
	GO:0044238	primary metabolic process	BP	6.48E-11	Over
	GO:0007602	phototransduction	BP	2.33E-10	Over
	GO:0007601	visual perception	BP	3.29E-08	Over
	GO:0007186	G-protein coupled receptor signaling pathway	BP	0.018562	Over
Pm_Px	GO:0005506	iron ion binding	MF	1.14E-42	Over
	GO:0016491	oxidoreductase activity	MF	3.12E-42	Over
	GO:0020037	heme binding	MF	6.80E-41	Over

GO:0004497	monooxygenase activity	MF	1.33E-29	Over
GO:0009055	electron carrier activity	MF	1.08E-26	Over
GO:0046872	metal ion binding	MF	1.30E-17	Over
GO:0046914	transition metal ion binding	MF	3.38E-14	Over
GO:0004601	peroxidase activity	MF	4.99E-14	Over
GO:0008146	sulfotransferase activity	MF	6.33E-11	Over
GO:0008745	N-acetylmuramoyl-L-alanine amidase activity	MF	5.71E-10	Over
GO:0003824	catalytic activity	MF	1.85E-09	Over
GO:0004656	procollagen-proline 4-dioxygenase activity	MF	8.11E-08	Over
GO:0003796	lysozyme activity	MF	2.73E-06	Over
GO:0016702	oxidoreductase activity, acting on single donors with incorporation of molecular oxygen, incorporation of two atoms of oxygen	MF	5.38E-06	Over
GO:0031418	L-ascorbic acid binding	MF	5.38E-06	Over
GO:0005509	calcium ion binding	MF	0.000176	Over
GO:0042302	structural constituent of cuticle	MF	0.031357	Over
GO:0005783	endoplasmic reticulum	CC	0.012441	Over
GO:0055114	oxidation-reduction process	BP	3.02E-54	Over
GO:0006979	response to oxidative stress	BP	4.99E-14	Over
GO:0008299	isoprenoid biosynthetic process	BP	5.92E-14	Over
GO:0009253	peptidoglycan catabolic process	BP	5.71E-10	Over
GO:0007602	phototransduction	BP	6.03E-10	Over
GO:0008152	metabolic process	BP	8.80E-09	Over
GO:0007601	visual perception	BP	7.23E-08	Over
GO:0016998	cell wall macromolecule catabolic process	BP	2.73E-06	Over
GO:0007600	sensory perception	BP	9.17E-05	Over
GO:0009056	catabolic process	BP	0.000933	Over
GO:0050896	response to stimulus	BP	0.031964	Over
GO:0007186	G-protein coupled receptor signaling pathway	BP	0.036352	Over

False Discovery Rate (FDR). *P. xuthus* (*Px*), *P. machaon* (*Pm*).

Supplementary Table 23. Numbers of orthologs among 4 butterflies and silkworm.

Species pair	Ortholog number
Pm:*Px*	11,784
Pm:*Dp*	10,605
Pm:*Hm*	10,445
Pm:*Bm*	9,716
Px:*Dp*	10,553
Px:*Hm*	10,430
Px:*Bm*	9,706

P. xuthus (*Px*), *P. machaon* (*Pm*), *H. melpomene* (*Hm*), *D. plexippus* (*Dp*), *B. mori* (*Bm*).

Supplementary Table 24. List of positively selected genes in *P. xuthus* (*Px*).

**Px_01958_CG34401**	**Px_15070_unknow**	Px_05397_CG5220	Px_09899_Su(P)	Px_14013_cdc2rk
**Px_02101_CG30115**	**Px_15170_Cg25C**	Px_05399_CG11109	Px_09910_unknow	Px_14106_unknow
**Px_02855_mus312**	**Px_15541_CG11337**	Px_05830_CG9642	Px_09986_CG16789	Px_14428_CG3709
**Px_03329_CG7922**	**Px_16144_unknow**	Px_05885_CG5808	Px_10136_CG34461	Px_14685_CG8213
**Px_03366_CG14073**	**Px_16435_CG6330**	Px_06006_CG12821	Px_10400_unknow	Px_14874_CG9005
**Px_03538_Pcmt**	**Px_16487_bsf**	Px_06099_unknow	Px_10420_CG10347	Px_14929_m-cup
**x_03649_CG6654**	**Px_16700_CG4554**	Px_06134_CG11263	Px_10441_CG5245	Px_15106_CG9247
**Px_04119_CG18809**	**Px_16766_unknow**	Px_06171_egh	Px_10501_RpS23	Px_15200_p16-ARC
**Px_04435_l(2)gd1**	**Px_16950_unknow**	Px_06650_l(1)G0269	Px_10722_Smg1	Px_15209_GM130
**Px_05316_CG7759**	**Px_17052_unknow**	Px_06690_pgant3	Px_10855_CG5964	Px_15220_Aats-ala
**Px_05365_Moca-cyp**	**Px_17130_unknow**	Px_06757_CG3226	Px_10900_se	Px_15231_CG31759
**Px_05510_CG15080**	**Px_17319_CG10979**	Px_06889_CG10953	Px_10997_oxt	Px_15248_PNUTS
**Px_05693_cp309**	Px_01239_BicC	Px_06927_unknow	Px_11009_unknow	Px_15336_CG9932
**Px_05826_CG17086**	Px_01248_CG2701	Px_07006_Spase25	Px_11031_CG16838	Px_15382_ck
**Px_05855_CG12301**	Px_01294_CG5660	Px_07054_Upf2	Px_11186_CG13397	Px_15409_unknow
**Px_06223_Aats-gln**	Px_01306_CG6280	Px_07086_CG12125	Px_11386_unknow	Px_15412_ninaC
**Px_06341_CG13472**	Px_01449_unknow	Px_07128_unknow	Px_11415_CG10535	Px_15432_Iap2
**Px_06394_Ten-m**	Px_01543_unknow	Px_07297_CG6294	Px_11422_CG14200	Px_15452_sr
**Px_06503_unknow**	Px_01847_Hex-A	Px_07303_CG1553	Px_11432_Ku80	Px_15456_CG13624
**Px_06761_Sry-alpha**	Px_02112_CG30122	Px_07420_unknow	Px_11568_Ggamma30A	Px_15551_CG10221
**Px_06898_gry**	Px_02516_CG7429	Px_07469_unknow	Px_11598_mus309	Px_15599_CG5728
**Px_07014_mus201**	Px_02742_unknow	Px_07652_mud	Px_11614_CG31320	Px_15717_CG3605
**Px_07317_CG10738**	Px_02789_nos	Px_07792_su(Hw)	Px_11622_Iswi	Px_15844_CG15661
**Px_07586_Mlh1**	Px_02997_CG8378	Px_07807_CG31198	Px_11634_SC35	Px_15848_spn-E
**Px_07729_CG13185**	Px_02998_CG30104	Px_07910_CG1575	Px_11783_nbs	Px_15889_unknow
**Px_08541_rno**	Px_03179_CG32708	Px_08027_CG6043	Px_11834_unknow	Px_16021_WDR79
**Px_08997_CG6133**	Px_03312_unknow	Px_08188_CG12084	Px_11971_CG6696	Px_16040_CG14232
**Px_09089_unknow**	Px_03374_CG6000	Px_08212_Pxn	Px_12080_CG32104	Px_16050_CG4213
**Px_09182_CG9305**	Px_03470_CG8078	Px_08213_CG16985	Px_12084_CG4572	Px_16088_CG32815
**Px_09223_Dab**	Px_03489_MESR4	Px_08217_Cralbp	Px_12156_unknow	Px_16118_CG3368
**Px_09535_unknow**	Px_03507_CG9139	Px_08243_Tpi	Px_12182_Smr	Px_16143_CG32685
**Px_09813_CG7422**	Px_03532_CG3271	Px_08271_unknow	Px_12218_CG7879	Px_16153_unknow
**Px_09982_Not1**	Px_03542_CG17514	Px_08493_CG10508	Px_12245_CG13366	Px_16185_CG15896
**Px_11000_Mtor**	Px_03627_Cpr50Ca	Px_08538_CG1309	Px_12246_unknow	Px_16211_CG13398
**Px_11034_CG16940**	Px_03671_unknow	Px_08570_Cyp305a1	Px_12248_Pp2C1	Px_16222_Tbp
**Px_12008_kz**	Px_03708_Nek2	Px_08585_noi	Px_12700_unknow	Px_16854_ird5
**Px_12049_Rnp4F**	Px_03735_unknow	Px_08667_unknow	Px_12738_Gnf1	Px_16968_CG5033
**Px_12555_CG3532**	Px_03821_unknow	Px_08680_ref(2)P	Px_12822_CG7430	Px_17002_CG7137
**Px_12623_CG1815**	Px_03953_CG14303	Px_08820_unknow	Px_12908_CG6664	Px_17085_CG12014
**Px_12709_MBD-R2**	Px_04065_unknow	Px_09120_unknow	Px_13210_CG8959	Px_17109_l(2)09851
**Px_12882_unknow**	Px_04153_CG14120	Px_09129_EndoG	Px_13213_CG3149	Px_17207_unknow
**Px_12987_CG1311**	Px_04195_CG10710	Px_09258_XNP	Px_13215_CG14767	Px_17277_CG6937
**Px_13050_unknow**	Px_04240_CG31457	Px_09296_CG13708	Px_13251_CG3335	Px_17365_CG11790
**Px_13258_xmas-2**	Px_04296_CG6136	Px_09371_CG3520	Px_13356_CG1625	Px_17384_unknow
**Px_13403_Cht6**	Px_04582_CG31274	Px_09547_Egm	Px_13398_unknow	Px_17572_unknow
**Px_13537_CG14882**	Px_04761_CG15744	Px_09587_CG12299	Px_13523_Mms19	Px_17650_CG30085
**Px_13616_CG3803**	Px_04781_Sln	Px_09664_CG10428	Px_13828_CG5867	Px_17677_CLIP-190
**Px_14333_Ret**	Px_05026_CG3773	Px_09784_CG6287	Px_13936_Fs	Px_17712_CG42374
**Px_14756_unknow**	Px_05341_CG34376	Px_09804_CG9098	Px_13959_msl-2	Px_17778_CG4611
**Px_14946_dtr**	Px_05375_su(s)	Px_09877_CG11203	Px_13985_CG33003	Px_17835_CG30394
**Px_14966_l(3)07882**	Px_05378_Rpb4	Px_09883_CG8830	Px_14000_pps	Px_17879_Ir21a

Bold highlighted genes are also positively selected in *P. machaon*.

Supplementary Table 25. List of positively selected genes in *P. machaon* (*Pm*).

Pm_20141_CG8492	***Pm_07599_unknow***	*Pm_13949_CG16791*	*Pm_15548_CG11015*	*Pm_11842_CG17807*
Pm_20310_rno	***Pm_09181_alphaTub84B***	*Pm_13980_unknow*	*Pm_15590_unknow*	*Pm_09034_CG32772*
Pm_16982_CG7922	***Pm_19388_CG34401***	*Pm_14054_CG9522*	*Pm_15617_CG15141*	*Pm_17323_CG7747*
Pm_17018_CG14073	***Pm_19431_unknow***	*Pm_18247_Antp*	*Pm_15628_dl*	*Pm_17372_DNApol-eta*
Pm_06240_CG14882	***Pm_07328_Ret***	*Pm_04202_CG31869*	*Pm_15678_ds*	*Pm_08286_CG7510*
Pm_05768_Pcmt	***Pm_07203_Dab***	*Pm_04073_fab1*	*Pm_15753_Sap-r*	*Pm_08105_ct*
Pm_18337_CG11337	***Pm_07002_unknow***	*Pm_05929_CG10321*	*Pm_15761_Cad88C*	*Pm_08122_mbo*
Pm_18520_Aats-gln	***Pm_10493_CG30115***	*Pm_13120_CG42284*	*Pm_11338_CG7718*	*Pm_08127_Sse*
Pm_08509_Mlh1	***Pm_10525_unknow***	*Pm_13150_CG11436*	*Pm_11154_CG9619*	*Pm_08151_unknow*
Pm_09841_MBD-R2	***Pm_08742_unknow***	*Pm_13173_Rrp1*	*Pm_11189_Ppt1*	*Pm_11272_Past1*
Pm_05241_mus312	*Pm_20223_gammaSnap*	*Pm_03987_CG10252*	*Pm_18801_unknow*	*Pm_11277_unknow*
Pm_14736_CG17086	*Pm_20321_Taf7*	*Pm_04605_CG14331*	*Pm_18822_CG15523*	*Pm_11295_msl-3*
Pm_14763_CG12301	*Pm_20338_CG10804*	*Pm_05971_Meics*	*Pm_18884_Clk*	*Pm_11315_unknow*
Pm_05158_CG15080	*Pm_20350_sowah*	*Pm_12888_CG7632*	*Pm_19081_CSN8*	*Pm_08796_Rox8*
Pm_07188_crol	*Pm_20585_CG9657*	*Pm_12893_CG12484*	*Pm_10939_unknow*	*Pm_07929_Corin*
Pm_04888_CG6133	*Pm_16871_unknow*	*Pm_12938_Pmi*	*Pm_10945_CG6836*	*Pm_07911_Bub1*
Pm_06292_l(3)07882	*Pm_16945_CG8483*	*Pm_12950_unknow*	*Pm_10974_CG9213*	*Pm_08006_CG5168*
Pm_06311_dtr	*Pm_17060_CG5290*	*Pm_12995_CG30010*	*Pm_02495_CG3074*	*Pm_16584_Ugt86Da*
Pm_06814_Cht6	*Pm_09460_CG16908*	*Pm_12999_CG17059*	*Pm_15218_CG32436*	*Pm_16593_Rad17*
Pm_13177_CG13472	*Pm_07439_CG3107*	*Pm_06081_CG10055*	*Pm_15317_unknow*	*Pm_16649_unknow*
Pm_12847_unknow	*Pm_06250_unknow*	*Pm_12778_cyc*	*Pm_10872_Notum*	*Pm_16703_CG9418*
Pm_12985_Cg25C	*Pm_05783_CG4078*	*Pm_12781_unknow*	*Pm_10903_unknow*	*Pm_16705_CG7544*
Pm_12710_Not1	*Pm_18443_CG9951*	*Pm_12796_CG3213*	*Pm_10728_mRpL19*	*Pm_16707_CG6379*
Pm_12795_Smyd4	*Pm_18763_CG8273*	*Pm_03543_CG15019*	*Pm_10729_CG5934*	*Pm_16756_mthl10*
Pm_19701_CG6330	*Pm_08484_Gyc88E*	*Pm_03483_Ir8a*	*Pm_10762_CG7878*	*Pm_16782_mge*
Pm_19874_unknow	*Pm_08494_tin*	*Pm_19730_GlyP*	*Pm_10800_CycJ*	*Pm_16790_Hip1*
Pm_19943_CG1311	*Pm_08515_CG12909*	*Pm_19920_140up*	*Pm_13658_l(1)G0144*	*Pm_07817_CG13692*
Pm_20055_ft	*Pm_09796_Mcm7*	*Pm_20026_CG15269*	*Pm_13345_CG1234*	*Pm_12222_CG10431*
Pm_12391_Sry-alpha	*Pm_09801_CG12807*	*Pm_03282_Dap160*	*Pm_14527_DLP*	*Pm_12254_CG2862*
Pm_15038_CG3532	*Pm_09832_Meics*	*Pm_14893_unknow*	*Pm_14576_RpL34b*	*Pm_12319_CG13759*
Pm_15116_CG1815	*Pm_09871_CG10431*	*Pm_14925_CG3493*	*Pm_14621_unknow*	*Pm_11675_CG3909*
Pm_14264_unknow	*Pm_09890_CG10333*	*Pm_14928_phr6-4*	*Pm_02281_Cad87A*	*Pm_11744_ade3*
Pm_02903_unknow	*Pm_07725_unknow*	*Pm_14947_CG2794*	*Pm_10175_lig*	*Pm_09173_unknow*
Pm_15738_bsf	*Pm_16345_CG12879*	*Pm_15051_CG33331*	*Pm_17709_yip2*	*Pm_09174_unknow*
Pm_11409_CG4554	*Pm_16454_mRpL37*	*Pm_03185_CG4935*	*Pm_17832_CG5482*	*Pm_19281_Vha14*
Pm_11026_CG16940	*Pm_16460_unknow*	*Pm_15882_unknow*	*Pm_17923_CG30069*	*Pm_19330_unknow*
Pm_11059_Mtor	*Pm_16475_unknow*	*Pm_15920_CG7246*	*Pm_10027_HP1b*	*Pm_19345_CG11403*
Pm_02870_unknow	*Pm_16498_CG3259*	*Pm_15933_CG14215*	*Pm_10365_Gmer*	*Pm_19490_unknow*
Pm_19073_unknow	*Pm_16526_CG2698*	*Pm_15955_tatl*	*Pm_11925_unc-119*	*Pm_19496_CG15905*
Pm_19112_unknow	*Pm_16547_unknow*	*Pm_04352_CG34183*	*Pm_09660_CG13957*	*Pm_19573_Mipp2*
Pm_15336_CG10979	*Pm_05492_mu2*	*Pm_03461_Pcf11*	*Pm_09665_Trf*	*Pm_19632_CG8549*
Pm_10259_CG9305	*Pm_08299_barr*	*Pm_13730_ast*	*Pm_09669_rab3-GAP*	*Pm_19643_CG9386*
Pm_17856_CG13185	*Pm_05207_unknow*	*Pm_13787_mys*	*Pm_12076_CG7441*	*Pm_07318_unknow*
Pm_09998_l(2)gd1	*Pm_14709_CG18136*	*Pm_13818_CG5195*	*Pm_09363_CG13350*	*Pm_07320_unknow*
Pm_11911_Rnp4F	*Pm_14827_CG6984*	*Pm_13823_CG6230*	*Pm_09394_CG5190*	*Pm_07220_Hnf4*
Pm_11951_kz	*Pm_05709_CG18631*	*Pm_13893_mei-W68*	*Pm_11464_Ca-beta*	*Pm_07242_unknow*
Pm_09577_unknow	*Pm_05132_unknow*	*Pm_14096_CG15561*	*Pm_17665_CG5288*	*Pm_07102_CG2972*
Pm_12124_CG18809	*Pm_05052_ssp3*	*Pm_14182_CG8814*	*Pm_17675_unknow*	*Pm_07013_CG17264*
Pm_11855_Ten-m	*Pm_05017_Galpha73B*	*Pm_14268_CG13032*	*Pm_09276_unknow*	*Pm_16128_unknow*
Pm_13436_gry	*Pm_14288_CG11342*	*Pm_11649_l(2)37Bb*	*Pm_12450_CG2918*	*Pm_09789_CG6904*
Pm_08010_mus201	*Pm_14295_CG9117*	*Pm_15367_Oaz*	*Pm_12492_l(1)G0255*	*Pm_06854_CG1648*
Pm_16683_CG17266	*Pm_08163_zetaTry*	*Pm_15412_Vps4*	*Pm_10602_unknow*	*Pm_06860_Gug*
Pm_16739_xmas-2	*Pm_04849_CG9319*	*Pm_15443_unknow*	*Pm_11818_CG6359*	*Pm_08708_CG2278*

Bold highlighted genes are also positively selected in *P. xuthus*.

Supplementary Table 26. Gene Ontology (GO) enrichment of the positively selected genes in *P. xuthus* (*Px*) and *P. machaon* (*Pm*).

	GO_ID	GO_Term	GO_Class	P value
Pm	GO:0006139	nucleobase-containing compound metabolic process	BP	1.72E-05
	GO:0090304	nucleic acid metabolic process	BP	6.53E-05
	GO:0006281	DNA repair	BP	0.000141636
	GO:0008168	methyltransferase activity	MF	0.000332975
	GO:0006807	nitrogen compound metabolic process	BP	0.00061991
Px	GO:0003676	nucleic acid binding	MF	3.46E-07
	GO:0005488	binding	MF	3.71E-05

Supplementary Table 27. The statistics of transcriptome sequencing for *P. xuthus* (*Px*) and *P. machaon* (*Pm*).

Library	Total Pair end reads (M)	Total Base (Gb)	Alignment base (Gb)	Alignment ratio
Pm_egg	25.35	4.56	3.53	77.26%
Pm_L1	27.24	4.90	3.73	76.00%
Pm_L2	34.71	6.25	4.86	77.80%
Pm_L3	27.32	4.92	3.72	75.72%
Pm_L4	31.54	5.68	4.29	75.55%
Pm_L5	36.05	6.49	4.87	74.99%
Pm_pupa_female	28.26	5.09	3.97	77.96%
Pm_pupa_male	34.01	6.12	4.49	73.34%
Pm_adult_female	29.00	5.22	4.11	78.66%
Pm_adult_male	33.89	6.10	3.91	64.10%
Px_egg	28.40	5.11	4.13	80.74%
Px_L1	31.27	5.63	4.62	82.11%
Px_L2	36.67	6.60	5.60	84.83%
Px_L3	34.89	6.28	5.15	82.02%
Px_L4	35.18	6.33	5.27	83.16%
Px_L5	30.03	5.41	4.31	79.67%
Px_pupa_female	26.59	4.79	4.02	83.98%
Px_pupa_male	28.37	5.11	4.32	84.65%
Px_adulte_female	25.82	4.65	3.92	84.44%
Px_adulte_male	29.12	5.24	4.33	82.63%

L: larva.

Supplementary Table 28. Developmental stage specific genes based on transcriptome data for *P. xuthus* (*Px*) and *P. machaon* (*Pm*).

Stage	Stage-specific genes number	
	Pm	***Px***
egg	66	78
L1	66	115
L2	262	105
L3	10	26
L4	27	13
L5	155	59
pupa_F	86	51
pupa_M	261	304
adult_F	180	147
adult_M	278	554

L: larva.

Supplementary Table 29. Differential expression gene numbers in different developmental stages for *P. xuthus* (*Px*) and *P. machaon* (*Pm*).

Diff_exp_stages	Diff_exp_gene_number
**10 stages**	**32**
9 stages	51
8 stages	97
7 stages	208
6 stages	455
5 stages	756
4 stages	1,145
3 stages	1,475
2 stages	1,782
1 stages	1,903

10 stages represent egg, 5 larva stages of 1st to 5th intar, male pupa, female pupa, male adult, and femal adult.

Supplementary Table 30. The number of differentially expressed genes at differentially devolomental stages for *P. xuthus* (*Px*) and *P. machaon* (*Pm*).

Fold	egg	L1	L2	L3	L4	L5	Pupa_F	Pupa_M	Adult_F	Adult_M	Total
>2	209	106	210	60	102	90	164	212	411	339	1903
>10	7	4	45	2	2	4	8	6	11	16	105

Supplementary Table 31. Copy numbers of genes encoding enzymes in the pathway of short-chain isoprenyl diphosphate (scIPPS) biosynthesis, juvenile hormone (JH) biosynthesis and degradation.

Fun	*Ag*	*Am*	*Bm*	*Dp*	*Dm*	*Hm*	*PLX*	*Pm*	*Px*	*Tc*	Inquery id	Function note
AB274988	1	1	1	1	1	1	1	**1**	**1**	1	AB274988	Acetoacetyl-CoA thiolase (AACT)
AB274989	1	1	1	1	1	1	1	**1**	**1**	1	AB274989	3-Hydroxy-3-methylglutaryl-CoA synthase (HMGS)
AB274990	1	1	1	1	1	1	1	**1**	**1**	2	AB274990	3-Hydroxy-3-methylglutaryl-CoA reductase(HMGR)
AB274991	1	1	1	1	1	1	1	**2**	**1**	1	AB274991	Mevalonate kinase (MevK)
AB274992	1	1	1	1	1	1	0	**1**	**1**	1	AB274992	Phosphomevalonate kinase (MevPK)
AB274993	1	1	1	1	1	1	1	**1**	**1**	1	AB274993	Diphosphomevalonate decarboxylase (MevPPD)
AB274994	1	0	1	1	1	1	1	**1**	**1**	1	AB274994	Isopentenyl diphosphate isomerase (IPPI)
scIPPS	2	8	3	8	2	9	3	**15**	**15**	3		short-chain isoprenyl diphosphate (scIPPS**)**
FBgn0030347	4	0	2	1	3	1	1	**1**	**1**	3	FBgn0030347	Farnesyl diphosphate pyrophosphatase (FPPP)
GQ344797	8	2	1	1	10	6	2	**1**	**1**	14	GQ344797	Farnesol oxidase (FO)/Farmesol dehydrogenase(FolD)
KC243495	1	1	2	1	1	1	1	**2**	**1**	3	KC243495	Farnesal dehyrogenase (FalD)
NM_001146725	7	4	3	4	2	4	4	**3**	**3**	3	NM_001146725	JH epoxidase(JHO)
AB113578	1	1	7	1	1	15	1	**1**	**1**	2	AB113578	JH methyl transferase (JHMT)
AF287267	25	13	38	35	11	31	27	**40**	**37**	28	AF287267	JH esterase (JHE)
AY377854	3	1	6	7	3	8	6	**13**	**15**	5	AY377854	JH epoxide hydrolase(JHEH)
AY363308	1	1	4	3	1	4	2	**8**	**8**	1	AY363308	JH diol kinase (JHDK)
AB201556	1	1	1	1	1	1	1	**1**	**1**	1	AB201556	farnesyltransferase (FT)β: FNTB
AB201555	1	1	1	1	1	1	1	**1**	**1**	1	AB201555	farnesyltransferase (FT)α: FNTA
AB201555	1	1	1	1	1	1	1	**1**	**1**	1	AB201555	geranylgeranyltransferase 1 (GGT1)α: FNTA
AB568272	0	1	1	1	1	1	1	**1**	**1**	1	AB568272	geranylgeranyltransferase 1 (GGT1)β: PGGT1B
XM_004927714	2	2	1	1	1	1	1	**1**	**2**	1	XM_004927714	geranylgeranyltransferase2 (GGT2)α: RABGGTA
FBgn0028970	1	1	1	1	1	1	1	**1**	**1**	1	FBgn0028970	geranylgeranyltransferase2 (GGT2)β: RABGGTB

Including 4 butterflies: *P. xuthus* (*Px*), *P. machaon* (*Pm*), *H. melpomene* (*Hm*), *D. plexippus* (*Dp*),; 2 moths: *B. mori* (*Bm*), *P. xylostella* (*PLX*); 1 fruitfly: *D. melanogaster* (*Dm*): 1 mosquito: *A. gambiae* (*Ag*); 1 beetle: *T. castaneum* (*Tc*); 1 bee : *A. mellifera*(*Am*)**.**

Supplementary Table 32. Short-chain isoprenyl diphosphate (scIPPS) and their classification in 13 insects.

Species	Total	GPPS	FPPS	GGPPS
Pm	**15**	0	14	1
Px	**15**	0	14	1
Hm	**9**	0	1	8
Dp	**8**	0	1	7
Bm	3	0	2	1
PLX	3	0	3	0
Dm	2	0	1	1
Ag	2	0	1	1
Tc	3	0	2	1
Am	**8**	0	7	1
**Nv**	**4**	2	1	2
Cflo	**5**	2	1	1
Hsal	**5**	2	1	2

The 13 insects including four butterflies: *P. xuthus* (*Px*), *P. machaon* (*Pm*), *H. melpomene* (*Hm*), *D. plexippus* (*Dp*); 2 moths: *B. mori* (*Bm*), *P. xylostella* (*PLX*); 1 fruitfly: *D. melanogaster* (*Dm*): 1 mosquito: *A. gambiae* (*Ag*); 1 beetle: *T. castaneum* (*Tc*); 1 bee: *A. mellifera* (*Am*); 1 wasp: *Nasonia vitripennis* (Nv); 2 ants: *Camponotus floridanus* (Cflo), *Harpegnathos saltato* (Hsal).

Geranyl diphosphate synthase (GPPS); Farnesyl diphosphate synthase (FPPS); Geranylgeranyl diphosphate synthase (GGPPS); Farnesylgeranyl diphosphate synthase (FDPPS)

Supplementary Table 33. Differential expression of farnesyl diphosphate synthase (FPPS) genes between *P. xuthus* (*Px*) and *P. machaon* (*Pm*).

Orthlog of *Pm*		Orthlog of *Px*		Total stages of differential expression	Differential expression stages	Folds of differential expression
Pm_07192_Fpps	up	*Px_02637_Fpps*		1	L2	16.71
Pm_07696_Fpps	up	*Px_02639_Fpps*		1	L2	3.47
Pm_07194_Fpps	up	*Px_02636_Fpps*		2	L3, adult_F	5.33
Pm_07695_Fpps	up	*Px_02638_Fpps*		2	L1, egg	5.83
Pm_07698_Fpps	up	*Px_02641_Fpps*		2	L1, L3	14.31
Pm_07701_Fpps	up	*Px_02642_Fpps*		3	L2, L4, egg	15.31
Pm_07702_Fpps	up	*Px_02643_Fpps*		3	L2, L4, adult_M	8.85
Pm_16572_qm		*Px_03398_qm*	up	3	L1, adult_F, egg	7.571
Pm_07190_Fpps		*Px_00691_Fpps*				
Pm_17866_Fpps		*Px_02640_Fpps*				
Pm_12194_Fpps		*Px_04052_Fpps*				
Pm_10221_Fpps		*Px_09247_Fpps*				
Pm_07694_Fpps		*Px_09248_Fpps*				
NA		*Px-02635-Fpps*				
NA		*Px_00692_Fpps*				
Pm_07193_Fpps		NA				
Pm_07700_Fpps		NA				

NA, not applicable.

Supplementary Table 34. Copy numbers of three detoxification gene families, cytochrome P450, carboxylesterases (COE), and glutathione S-transferase (GST) identified in four butterflies, silkworm, and fruitfly.

Category	*Pm*	*Px*	*Hm*	*Dp*	*Bm*	*Dm*
P450	107	94	119	(88)	(88)	(84)
GST	34	31	29	23	23	33
COE	80	71	79	70	78	31

The number of P450 in the bracket is from reference[55].

P. xuthus (*Px*), *P. machaon* (*Pm*), *H. melpomene* (*Hm*), *D. plexippus* (*Dp*), *B. mori* (*Bm*), *D. melanogaster* (*Dm*).

Supplementary Table 35. Gene numbers of cytochrome P450 superfamily and its some gene familiesin 4 butterflies, silkworm and fruitfly.

**Category**	***Pm***	***Px***	***Hm***	***Dp***	***Bm***	***Dm***
P450	107	94	119	(88)	(88)	(84)
CYP4	14	10	18	17	13	19
CYP9	6	6	7	6	8	6
CYP6	***39***	***30***	***26***	***17***	***15***	***22***
CYP6B	***20***	***16***	***0***	***0***	***1***	***0***
CYP6AB	***14***	***8***	***10***	***7***	***4***	***0***

The number in the bracket is from referene[55]**.**

4 butterflies; *P. xuthus* (*Px*), *P. machaon* (*Pm*), *H. melpomene* (*Hm*), *D. plexippus* (*Dp*); silkworm: *B. mori* (*Bm*); fruitfly: *D. melanogaster* (*Dm*).

Supplementary Table 36. Copy number of pigment-related genes in four butterflies, silkworm and fruitfly.

Query.id	Px	Pm	Hm	Dp	Bm	Dm	Gene_name (symbol)	Pathywas
FBgn0001208	1	1	1	1	1	1	Henna (Hn)	M-Pap-S
FBgn0005626	1	1	1	1	1	1	pale (ple)	M-Pap-S
FBgn0000422	2	1	1	1	1	1	Ddc (Ddc)	M-Pap-S
AB499123	1	2	2	3	2	1	laccase 2 (CG42435)	M, Pap, S
FBgn0004034	11	12	10	16	10	14	Yellow (y)	M, Pap, S
FBgn0086367	1	1	1	1	1	1	tan (t)	M-Pap-S
FBgn0000153	1	1	1	1	1	1	black (b)	Pap
FBgn0000527	1	1	1	1	1	1	ebony (e)	Pap
FBgn0019643	2	2	1	1	2	1	Dopamine N acetyltransferase (Dat)	S
FBgn0003965	1	1	2	1	1	1	vermillion (v)	O
XP_967644	1	1	1	1	3	1	kynurenine formamidase (kf)	O
FBgn0000337	2	1	1	1	1	1	Cinnabar (cn)	O
FBgn0263986	1	1	1	1	2	1	cardinal (cd)	O
FBgn0003210	2	1	2	1	1	1	Ruby (rb)	O
FBgn0037955	1	1	1	1	1	1	kynurenine aminotransferase (CG6950)	O
FBgn0000241	2	1	2	1	3	4	brown (bw)	T
FBgn0002567	1	1	1	1	1	1	lightoid (ltd)	T
FBgn0000247	2	2	1	1	1	1	claret (ca)	T
FBgn0003996	1	1	2	2	1	1	white (w)	T
FBgn0003515; EU233799	3	3	1	3	3	1	scarlet (st)	T
FBgn0001296	2	1	1	1	2	1	karmoisin (kar)	T
EU233800	1	1	1	1	1	1	atet-like (atet-like)	T
FBgn0003162	1	3	1	1	1	1	Punch (Pu)	Pte
AB499124	1	1	1	1	1	1	purple (pr)	Pte
FBgn0014032	1	1	1	1	1	1	lemon (lem)	Pte
FBgn0035964	2	1	1	1	1	1	Dihydropterin reductase (CG4665) (Dhpr)	Pte
FBgn0000318	1	1	1	1	1	2	clot (cl)	Pte
FBgn0261436	1	1	1	0	1	1	Dihydropterin deaminase (DhpD)	Pte
FBgn0086348	1	1	1	1	1	1	sepia (se)	Pte
FBgn0003308	2	3	3	2	1	1	rosy (ry)	Pte
FBgn0024841	1	1	1	1	1	1	CG1963 (Pcd)	Pte
FBgn0038901	1	1	1	1	1	1	bursicon (burs)	
FBgn0262126	1	1	1	2	1	1	ghost (gho)	
FBgn0038947	1	1	1	1	1	1	sar1 (sar1)	
FBgn0262125	1	1	1	2	1	1	sec23 (sec23)	
FBgn0003008	3	1	1	1	1	1	orange (or)	
FBgn0000257	1	1	1	1	1	1	carnation (car)	
FBgn0261241	1	1	1	3	1	1	Vacuolar protein sorting 16A (Vps16A)	
FBgn0000482	1	1	1	1	1	1	deep orange (dor)	
FBgn0030343	1	4	2	2	2	1	ATP7 (ATP7)	
FBgn0001087	1	1	1	1	2	1	garnet (g)	
FBgn0002566	1	1	1	1	1	1	light (lt)	
FBgn0041184	2	1	1	1	1	1	Suppressor of cytokine signaling at 36E (Socs36E)	
FBgn0050077	1	1	1	1	1	1	BLOC-1 subunit 1 (blos1)	
FBgn0003028	1	1	1	1	1	1	ovo (evo)	
FBgn0024326	1	1	1	1	1	1	MAP kinase kinase 4 (Mkk4)	

FBgn0014859	1	1	1	1	1	1	Hormone receptor-like in 38 (Hr38)
FBgn0015765	1	2	1	1	1	2	Mpk2 (Mpk2)
FBgn0024846	2	1	1	1	1	1	p38b (p38b)
FBgn0046322	1	1	1	2	2	1	p38c (p38c)
FBgn0014006	6	1	1	1	1	1	Protein kinase at 92B (Pk92B)
FBgn0000015	1	1	1	0	1	1	Abdominal B (abd-B)
FBgn0004870	2	1	1	1	1	1	bric a brac 1 (bab1)
FBgn0000490	1	1	1	1	1	1	decapentaplegic (dpp)
FBgn0004644	3	3	3	2	2	1	hedgehog (hh)
FBgn0261524	1	1	1	1	1	1	licorne (lic)
FBgn0004009	1	1	1	1	1	1	wingless (wg)
FBgn0025615	1	1	1	1	1	1	Torsin (Torsin)
FBgn0000504	1	1	1	1	1	1	doublesex (dsx)
HQ020406	1	1	1	1	2	1	Antennapedia (Antp)
NM_001044085	5	2	1	2	4	2	Engrailed/invected (En)
FBgn0000119	1	2	1	1	1	1	arrow (arr)
FBgn0011758	2	2	2	1	1	1	BarH1 (B-H1)
FBgn0004854	0	1	0	1	1	1	BarH2 (B-H2)
FBgn0000179	2	2	3	2	2	1	bifid (bi)
FBgn0015622	1	2	1	1	1	3	Calnexin 99A (Cnx99A)
FBgn0000330	1	1	1	1	1	1	carmine (cm)
FBgn0000499	1	1	1	1	1	1	dishevelled (dsh)
FBgn0031464	1	1	1	2	1	1	Dual oxidase (Duox)
FBgn0001981	1	1	1	1	1	3	escargot (esg)
FBgn0038749	1	1	1	1	1	1	exit protein of rhodopsin and TRP (Xport)
FBgn0086130	1	1	1	1	1	1	E2DEAD box protein 21E2 (KH1)
FBgn0002641	1	3	1	1	1	1	maroon-like (mal)
FBgn0261260	1	1	1	1	1	1	Megalin (mgl)
FBgn0002936	1	1	1	1	1	1	ninaA (ninaA)
FBgn0002937	3	2	5	4	1	1	ninaB (ninaB)
FBgn0002939	0	0	1	3	0	3	ninaD (ninaD)
FBgn0037896	2	3	3	3	4	1	ninaG (nmaG)
FBgn0044028	1	1	1	1	1	1	Notum (Notum)
FBgn0038966	2	2	1	2	3	1	pinta (pinta)
FBgn0003116	1	1	1	2	1	1	prune
FBgn0025697	1	1	1	1	1	3	santa-maria (santa-maria)
FBgn0021764	3	2	1	1	1	1	sidekick (sdk)
FBgn0262866	1	1	1	1	1	1	Ribosomal protein S6 kinase II (S6kII)
FBgn0085408	0	0	0	0	0	1	Shroom (Shroom)
X57474	0	0	0	0	0	1	spalt-adjacent (Sal)
FBgn0004050	0	0	0	0	0	1	zeste (z)
FBgn0086679	0	0	0	0	0	1	pink (p)
AB525742	1	1	0	0	0	0	yellow-related gene (YRG)
AB264632	14	12	9	10	6	1	bilin-binding protein gene (BBP)
	1	1	1	1	1	1	CBP
	6	9	8	8	8	0	ombp

Abbreviation**:** **M:** melanin pathway; **Pap**: papiliochorme pathway; **O:** omnochrome pathway; **S:** sclerotization; **Pte:** pterine pathway; **T**: transport; *P. xuthus* (*Px*), *P. machaon* (*Pm*), *H. melpomene* (*Hm*), *D. plexippus* (*Dp*), *B. mori* (*Bm*), *D. melanogaster* (*Dm*).

Supplementary Table 37. Evolutionary analysis of positively selected pigment gene *sepia* in *P. xuthus*.

gene	p-value(chi-square test)	q-value(FDR)	ω0	ω1	ω2
Px_10900_se	1.73E-04	1.73E-04	0.07919	0.0293	0.3296

Supplementary Table 38. Copy number of genes in the planar cell polarity (PCP) pathway in 10 insects.

CG No.	Gene_id	*Ag*	*Am*	*Bm*	*Dp*	*Dm*	*Hm*	*PLX*	*Pm*	*Px*	*Tc*	Gene name (symbol)
**Fat/Dachsous PCP group factors**												
CG3352	FBgn0001075	1	1	1	2	1	2	2	**2**	**3**	2	*fat*(*ft*)
CG17941	FBgn0000497	2	3	2	2	1	2	2	**2**	**2**	2	*dachsous*(*ds*)
CG10917	FBgn0000658	1	0	1	1	1	1	0	**1**	**1**	1	*four-jointed*(*fj*)
CG6964	FBgn0010825	1	1	1	1	1	1	1	**2**	**1**	2	*atrophin*(*atro*)/*Gunge*(*Gug*)
CG10595/ *CG42840*	FBgn0262029	1	1	1	1	1	1	0	**1**	**0**	1	*dachs*(*d*)
**Fz/stan PCP group factors**												
CG17697	FBgn0001085	3	1	1	1	1	1	0	**1**	**1**	1	*frizzled*(*fz*)
CG17697	FBgn0001085	4	2	3	3	2	3	3	**3**	**4**	3	*frizzled*(*fz*) family(*fz2*,*fz3*,*fz4*)
CG18361	FBgn0000499	1	1	1	1	1	1	1	**1**	**1**	1	*disheveled*(*dsh*)
CG11084	FBgn0003090	1	1	1	1	1	1	1	**1**	**2**	1	*prickle*(*pk*)
CG8075	FBgn0015838	1	1	1	1	1	1	0	**1**	**1**	1	*Van Gogh*(*vang*)
CG11895	FBgn0024836	1	2	1	1	1	2	2	**1**	**1**	2	*starry night*(*stan*)
CG12342	FBgn0086898	0	1	1	1	1	1	1	**1**	**1**	1	*diego*(*dgo*)
CG8416	FBgn0014020	1	1	1	1	1	1	1	**1**	**1**	1	*Rho1* (*Rho1*)
**Tissue-specific PCP effectors**												
CG16993	FBgn0001259	1	1	1	1	1	1	1	**1**	**3**	1	*inturned*(*in*)
CG13396	FBgn0001084	1	1	1	1	1	1	1	**1**	**1**	1	*fuzzy*(*fy*)
CG17657	FBgn0086698	1	1	1	1	1	1	1	**1**	**1**	1	*fritz*(*frtz*)
CG43772/ *CG13913*	FBgn0264272	1	1	1	1	1	1	1	**1**	**1**	1	*multiple wing hairs*(*mwh*)

P. xuthus (*Px*), *P. machaon* (*Pm*), *H. melpomene* (*Hm*), *D. plexippus* (*Dp*); *B. mori* (*Bm*), *P. xylostella* (*PLX*); *D. melanogaster* (*Dm*): *A. gambiae* (*Ag*); *T. castaneum* (*Tc*); *A. mellifera* (*Am*).

Supplementary Table 39. List of PCP genes in *P. xuthus* (*Px*) and *P. machaon* (*Pm*).

Gene symbol	*Pm* orthlog	*Px* orthlog	Note
**Fat/Dachsous PCP group factors**			
ds	***Pm_15678_ds***	*Px_16554_ds*	Positively selected in *Pm*
ds	*Pm_15680_ds*	*Px_16552_ds*	
fj	*Pm_12210_fj*	*Px_14508_fj*	
ft	*Pm_19710_ft*	*Px_00718_ft*	
ft	*Pm_19714_ft*	*Px_16421_ft*	
ft	NA	*Px_16425_ft*	
Gug	***Pm_06860_Gug***	*Px_13360_Gug*	Positively selected in *Pm*
Gug	*Pm_06861_Gug*	NA	
d	*Pm_12084_d*	NA	
**Fz/stan PCP group factors**			
dgo	*Pm_14558_dgo*	*Px_11458_dgox*	
dsh	*Pm_04805_dsh*	*Px_01455_dsh*	
fz	*Pm_13043_fz*	***Px_15230_fz***	up-regulatedly expressed in all stages of *Px*
fz2	*Pm_12110_fz2*	*Px_04133_fz2*	
fz4	*Pm_07755_fz4*	*Px_02987_fz4*	
fz3	NA	*Px_01251_fz3*	
pk	*Pm_05215_pk*	*Px_01635_pk*	
pk	NA	*Px_07366_pk*	
Rho1	*Pm_17811_Rho1*	*Px_09252_Rho1*	
stan	*Pm_05830_stan*	*Px_10185_stan*	
vang	*Pm_11813_Vang*	*Px_04780_Vang*	
**Tissue-specific PCP effectors**			
mwh	*Pm_17671_mwh*	*Px_11573_mwh*	
in	*Pm_18111_in*	*Px_13752_unknow*	
in	NA	*Px_11083_inx*	
in	NA	*Px_17722_inx*	
fy	*Pm_19850_fy*	*Px_16290_fy*	
frtz	*Pm_09630_frtz*	*Px_16252_frtz*	

Supplementary Table 40. Evolutionary analysis of two positively selected PCP genes in *P. machaon*.

gene	p-value (chi-squared)	q-value (FDR)	ω0	ω1	ω2
Pm_06860_Gug	2.24E-02	2.24E-02	0.01911	0.017	0.13705
Pm_15678_ds	3.49E-02	3.49E-02	0.01951	0.018	0.0348

Supplementary Table 41. Highly divergent regions in the *P. xuthus* genome resulted from three pairwise comparisons.

Chromosome	Scaffold	Position (bp)	$d_{f(Pp/Pm)}$	$d_{f(Px/Pp)}$	$d_{f(Px/Pm)}$
chr1	scaffold1_2	50000-100000	0.0416	0.03736	0.04664
chr1	scaffold1_2	150000-200000	0.04368	0.04902	0.03996
chr1	scaffold1_2	1650000-1700000	0.03532	0.03634	0.0365
chr1	scaffold1_2	1700000-1750000	0.0358	0.0398	0.03736
chr1	scaffold1_2	1850000-1900000	0.03876	0.03702	0.04166
chr1	scaffold1_2	3200000-3250000	0.03556	0.04112	0.0362
chr1	scaffold21_2	1750000-1800000	0.0399	0.04626	0.04148
chr2	scaffold26	1900000-1950000	0.03726	0.03672	0.03848
chr2	scaffold26	2350000-2400000	0.03528	0.0399	0.03944
chr2	scaffold22_1	150000-200000	0.03956	0.04072	0.0434
chr2	scaffold22_1	500000-550000	0.03664	0.03774	0.03954
chr2	scaffold22_1	600000-650000	0.03578	0.03732	0.03536
chr3	scaffold4_5	1800000-1850000	0.05576	0.05414	0.05206
chr3	scaffold4_5	2150000-2200000	0.03494	0.03942	0.04016
chr3	scaffold37	1550000-1600000	0.04024	0.04018	0.04116
chr5	scaffold3_1	250000-300000	0.0346	0.04406	0.03636
chr5	scaffold3_1	300000-350000	0.03976	0.04066	0.04392
chr6	scaffold4_9	450000-500000	0.0538	0.0666	0.04526
chr6	scaffold4_9	700000-750000	0.03666	0.04288	0.04016
chr6	scaffold4_9	1300000-1350000	0.0351	0.03654	0.0408
chr6	scaffold4_9	1550000-1600000	0.03564	0.03814	0.03692
chr6	scaffold4_9	2300000-2350000	0.03828	0.03924	0.04218
chr6	scaffold4_9	2800000-2850000	0.03684	0.03766	0.03848
chr6	scaffold30_2	1300000-1350000	0.03666	0.03648	0.03756
chr6	scaffold30_2	1400000-1450000	0.04586	0.0436	0.04584
chr6	scaffold30_2	1800000-1850000	0.0399	0.03674	0.04064
chr6	scaffold30_2	2050000-2100000	0.04536	0.04374	0.04684
chr7	scaffold16_1	1900000-1950000	0.03784	0.0397	0.04014
chr9	scaffold46	1350000-1400000	0.04052	0.04074	0.04398
chr9	scaffold43_2	50000-100000	0.04282	0.04166	0.04246
chr10	scaffold18_2	1700000-1750000	0.03492	0.03764	0.036
chr11	scaffold7_1	1600000-1650000	0.03582	0.0403	0.03574
chr12	scaffold5_2	0-50000	0.04134	0.03886	0.04008
chr12	scaffold5_2	850000-900000	0.03318	0.03992	0.03618
chr12	scaffold5_2	2200000-2250000	0.03134	0.04348	0.0365
chr12	scaffold5_2	2300000-2350000	0.04062	0.04636	0.043
chr12	scaffold5_2	2850000-2900000	0.03688	0.04018	0.03642
chr12	scaffold5_2	3300000-3350000	0.0357	0.04088	0.0355
chr13	scaffold13_2	1300000-1350000	0.03724	0.03972	0.03998
chr14	scaffold15_2	1000000-1050000	0.04212	0.04146	0.04298
chr15	scaffold6_1	200000-250000	0.0411	0.03738	0.0501
chr15	scaffold6_1	250000-300000	0.03888	0.03696	0.04888
chr15	scaffold6_3	0-50000	0.03554	0.03672	0.03772

chr15	scaffold6_3	1350000-1400000	0.03574	0.03748	0.03628
chr15	scaffold6_3	1450000-1500000	0.04644	0.04654	0.05148
chr15	scaffold6_3	1500000-1550000	0.03466	0.0385	0.0378
chr16	scaffold29_2	150000-200000	0.03918	0.04048	0.0431
chr16	scaffold29_2	300000-350000	0.03792	0.03756	0.04288
chr16	scaffold29_2	350000-400000	0.0402	0.04168	0.04218
chr16	scaffold29_2	400000-450000	0.04544	0.04526	0.04648
chr16	scaffold6_4	1850000-1900000	0.03854	0.0376	0.04454
chr16	scaffold6_4	3050000-3100000	0.03854	0.03788	0.03768
chr19	scaffold9_1	250000-300000	0.05274	0.0527	0.04994
chr20	scaffold62_1	600000-650000	0.03602	0.03828	0.03922
chr20	scaffold62_1	900000-950000	0.04172	0.04142	0.0404
chr21	scaffold19_2	150000-200000	0.0393	0.04104	0.0414
chr21	scaffold25	1550000-1600000	0.04196	0.03806	0.04466
chr21	scaffold25	2000000-2050000	0.0378	0.03688	0.0457
chr21	scaffold25	2050000-2100000	0.03512	0.037	0.03804
chr21	scaffold25	2250000-2300000	0.04118	0.04348	0.04136
chr21	scaffold25	2600000-2650000	0.04632	0.04468	0.04636
chr21	scaffold25	3450000-3500000	0.04168	0.04114	0.0452
chr21	scaffold25	3500000-3550000	0.04012	0.0413	0.0437
chr23	scaffold2_1	1350000-1400000	0.0316	0.03726	0.03544
chr23	scaffold2_1	1450000-1500000	0.03786	0.03866	0.04084
chr23	scaffold2_1	1650000-1700000	0.04108	0.04122	0.04446
chr23	scaffold2_1	1700000-1750000	0.03492	0.0391	0.039
chr25	scaffold12_2	50000-100000	0.04084	0.04014	0.04538
chr30	scaffold14_2	450000-500000	0.04076	0.04034	0.04766
chr30	scaffold14_2	2600000-2650000	0.04944	0.05396	0.04906

Supplementary Table 42. Functional enrichment of genes located in highly divergent regions.

GO_ID	GO_Term	GO_Class[a]	P value	Over/Under
GO:0055085	transmembrane transport	BP	0.000879	Under
GO:0038023	signaling receptor activity	MF	0.004159	Under
GO:0016740	transferase activity	MF	0.014129	Over
GO:0048519	negative regulation of biological process	BP	0.037833	Over
GO:0051234	establishment of localization	BP	0.040106	Under
GO:0016787	hydrolase activity	MF	0.040262	Under
GO:0009892	negative regulation of metabolic process	BP	0.045958	Over

[a]BP and MF mean biological process and molecular function, respectively.

Supplementary Table 43. The genes located in outlier regions with differentially expression at all development stages or positively selected in both *P. xuthus* (*Px*) and *P. machaon* (*Pm*).

	Gene ID
Differentially expressed genes	*Px_12650_Cct1*
	Px_15206_Kua
	Px_15230_fz
Positively selected genes in both *Px* and *Pm*	*Px_03366_CG14073*
	Px_05826_CG17086
	Px_05855_CG12301
	Px_07729_CG13185
	Px_12555_CG3532
	Px_12987_CG1311
	Px_13050_unknown
	Px_15070_unknown

Supplementary Table 44. Target sites and primers for knocking out genes *Abd-B*, *e*, and *fz* in *P. xuthus* (*Px*).

Gene	Target No.	Target sites (N20)	Strand	PAM	Primer Name	Primer
Px_03961_ Abd-B	**Px_03961_T42**	**GGTCCAGTCCAGGGGGTTAG**	minus	agg	Px_03961_F3	TTGTTTAGCTATTCGTCAGCC
	**Px_03961_T95**	**AGAGTCTGGAATTTGGAGTA**	minus	ggg	Px_03961_F9	TTAGCTATTCGTCAGCCGAT
	Px_03961_T100	GAGTCTGGAATTTGGAGTA	minus	ggg	Px_03961_F10	CGTCAGCCGATAATGGACAGGTT
	Px_03961_T248	GAAGAATTCACAGCGACAAG	plus	cgg	Px_03961_R6	CACGTACTAAGTCTCGTTACAGT
					Px_03961_R11	CTCGGAACCTTAAGTACATGC
Px_01073_e	**Px_01073_T2**	**GGCGGCGATGGCCCGGCCCA**	minus	ggg	Px_01073_F2	CTGCGTCTCTAGTCTATGTG
	**Px_01073_T303**	**GGGTGCAGCGTATGTTCCGA**	plus	tgg	Px_01073_R2	CTCCGCATCCTTTAGTATGTG
	**Px_01073_T454**	**GAACTATCTTTAGAAGCCAG**	plus	tgg	Px_01073_F7	TCACACATACTAAAGGATGCG
	**Px_01073_T6**	**GGAAGCACGGGGATACCGAA**	plus	agg	Px_01073_R4	TAACAATCACATCAATGCACT
Px_15230_fz	**Px_15230_T268**	**GATAAAGTGGAGAAAGACTG**	plus	tgg	Px_15230_F2	GCCAGAAAATTTGAATTGCT
	**Px_15230_T283**	**GACTGTGGTGCACCTTGCAA**	plus	tgg	Px_15230_R2	AAATACTTGAGGCCATGCTG
	Px_15230_T432	AGAAATATAATAGGTCTTTC	minus	agg		
	Px_15230_T474	GGTTGCAGCATCATATGTAA	plus	tgg		
	Px_15230_T508	GGTGACAGTGTAAGTTGTCA	plus	agg		

Bold represents the targets with morphologically phenotypic mutation.

Supplementary Table 45. Summary ofinjection combinations of different target sites and concentration of *Abd-B* sgRNA and Cas9 mRNA and information of mutations.

Treat No.	Combination	Injected final con. (ng/µl)	Injected eggs	Larva (h;uh)	Phenotype (+) (h;uh)	Phenotype (+) (%)	T7EI(+) (h; uh)	Sequenced(+) (h; uh)*	Mutation rate (h; uh)**	Mutation types (h; uh)
Px_03961_I	T42/T95/T248; cas9	100/25/25; 300	231	7; 8	0	0	NA	1(5); 2(7)	6%(4/60); 18%(22/120)	D: 4-68; D: 5-136, I: 2-25.
Px_03961_II	T42/T100; cas9	150/50; 50	110	5; 10	0		NA	NA	NA	NA
Px_03961_III	T42; cas9	300; 600	50	10; 10	0		NA	NA	NA	NA
WT(I-III)			59	13						
Px_03961_IV	T95/T100/248; cas9	60/50/100; 300	251	91; 43	0		NA	NA	NA	NA
Px_03961_V	T42/T95; cas9	50/50; 100	150	79; 21	3; 3	6	3(3), 1(33); 3(3), 13(21)	3(3), 1(1); 3(3), 4(13)	27%(7/26), 0; 12%(4/34), 21%(10/48)	D: 5-59, I: 3-4; D: 4-103, I: 4-19.
Px_03961_VI	T42; cas9	200; 600	199	90; 5	0		NA	NA	NA	NA
Px_03961_VII	T42; cas9	200; 300	146	69; 19	5; 2	7.95	5(5), 0(12); 2(2), 5(17)	5(5), 0(0); 2(2), 1(5)	43%(20/46), NA; 16%(3/19), 33%(2/6)	D: 3-51, I: 4-18; D: 4-8, I: 3.
Px_03961_VIII	T42; cas9	600; 600	160	75; 46	3; 7	8.26	3(3), 1(33); 7(7), 235)	3(3), 1(1); 7(7), 2(2)	23%(10/44), 0%(1/5); 28%(11/40), 9%(1/11)	D: 3-66, I: 4-5; D: 4-29, I: 4, M: 4-107.
Px_03961_IX	T42; cas9	1000; 600	110	27; 49	0		NA	NA	NA	NA
WT (IV-IX, XII)			251	245						
Px_03961_XIII	T42/T95; cas9	566/416; 1200	153	35; 99	27; 96	91.79	5(5);10(10)	5(5); 10(10)	95%(38/40); 87%(111/124)	D: 3-59, I: 3-62, M: 3-29; D: 3-73, I: 3-34, M: 3-13.
WT (XIII)			50	46						

The number before and after semicolon (;) represent the information of hatched (h) and unhatched (uh) larva; the number before stomma denote individuals with mutated morphologicall phenotype, and the number after stomma denote individuals without mutated morphologicall phenotype. *The number in bracket denotes totally checked indvidual number; ** Thefraction number in bracket denotesgenetically mutated individual/total checked individuals. D: delete fragment (bp); I: insert fragment (bp); M: mutated fragment (bp). NA: not applicable.

Supplementary Table 46. Summary of *Abd-B* gene mutants.

Types	Phenotype description	Hatched	Unhatched	Total	Mutation rate	Mutation types	Treat No.
Type1	abnormal terga of A3 and thereafter resulting abdomen curling up, ventral side of from A7 to A10 each with a pair of legs	**19**	**88**	**107**	90%	D: 3-73; I: 3-62; M: 3-29.	XIII
Type2	abnormal terga of A3 and thereafter resulting abdomen curling up, ventral side of from A7 to A9 each with a pair of legs	10	14	24	63%	D: 8-59; I: 4-18; M: 3-10.	V,VII,VIII,XIII
Type3	abnormal terga of A3 and thereafter resulting abdomen curling up, right or left ventral side of from A7 to A10 each with a leg	8	6	9	15%	D: 54-66; I: 4-19.	V,VIII,XIII
Type4	abnormal terga of A3 and thereafter resulting abdomen curling up, right or left ventral side of from A7 with a leg	2	6	8	25%	D: 5-123; I:3-19; M: 3-5.	V,VII,VIII
Type5	abnormal terga of A3 and thereafter resulting abdomen curling up without redudant legs on the ventral sides of from A7 to A10	7	2	9	25%	D:5-41; I: 4-5.	V,VII,VIII,XIII
Type6	noobvious morphologically mutation	212	69	281	0~24%	D: 4-103; I: 3-19.	V,VII,VIII,XIII
**Total**		**258**	**185**	**443**			
**Total(+)**		46	116	157			

D: delete fragment (bp); I: insert fragment (bp); M: mutated fragment (bp). NA: not applicable.

Supplementary Table 47. Summary of injection combinations of different target sites and concentration of *ebony* sgRNA and Cas9 mRNA and information of mutations.

Treat No.	Combination	Injected final con. (ng/μl)	Injected eggs	Hatched/ L5	Phenotype (+)	Phenotype (+) (%)	T7EI (+)	Sequenced (+)	Mutation rate (%)	Mutation types
Px_01073_I	T2<br>Cas9mRNA	40<br>300	1857	547/351	0 (351)	0	NA	0(160)	NA	NA
WT (I)			168	89						
Px_01073_II	T2/T303<br>Cas9mRNA	158/189<br>1200	136	60/15	5(16)	31.25	NA	5(5)	66.67	D: 5-33; I: 3-10; M: 3.
Px_01073_III	T454/T6<br>Cas9mRNA	200/150<br>1200	144	59/25	22(25)	88	NA	13(13)	29.92	D: 6-62; I: 3-15; M: 3-8.
WT (II-III)			50	46						

D: delete fragment (bp); I: insert fragment (bp); M: mutated fragment (bp). NA: not applicable.

Supplementary Table 48. Sumamry of *ebony* mutants.

**Types**	**Phenotype description of L5 larvae**	**II**	**III**	**Total**	**Mutation rate**	**Mutation types**	**Treatment No.**
Type1	orange area of both eye spot completely dissapearing or vestige; whole dorsal side darken more or less	4	12	16	40.74%	D: 5-40; I: 3-13.	II, III
Type2	orange area of left eye spot dissapearing; left dorsal side darken more or less	1	6	7	41.46%	D: 7-62; I: 15; M: 3.	II, III
Type3	orange area of right eye spot dissapearing; right dorsal side darken more or less	0	4	4	42.10%	D: 4-53; I: 3-11; M: 3-8.	II, III
Type4	orange area of both eye spot normal; whole dorsal side normal	11	3	14	NA	NA	II, III
Total		16	25	41			
Mutated Rate (%)		31 %	88%	61%			

D: delete fragment (bp); I: insert fragment (bp); M: mutated fragment (bp). NA: not applicable.

Supplementary Table 49. Summary of injection combinations of different target sites and concentration of *fz* sgRNA and Cas9 mRNA and information of mutations.

Treat No.	Combination	Injected final con. (ng/µl)	Injected eggs	Hatched	Phenotype (+)	Phenotype (+) (%)	T7EI(+)	Sequenced (+)	Mutation rate(%)	Mutation types
Px_15230_I	T432/474/508 Cas9mRNA	350/350/300 1000	190	121/	0	**0**	NA	NA	NA	NA
Px_15230_II	T268/283 Cas9mRNA	500/500 1000	188	96	4	4%	4 (4)	4 (4)	45.16	D: 3-64; I: 3-15; M: 4-5.
WT (I-II)			50	46						

D: delete fragment (bp); I: insert fragment (bp); M: mutated fragment (bp). NA: not applicable.

Supplementary Table 50. Summary of *frizzled* mutants.

Types	Phenotype description	I	II	Total	Mutation rate	Mutation types
Type1	mutated 2nd larva with prolegs of left side smaller than those of right side	0	1	1	66.66%	D: 4-64; I: 3; M: 4.
Type2	mutated 3rd larva with smoothy and colorless dorsal cuticle in right side	0	1	1	33.33%	D: 3; I: 4-5.
Type3	mutated 4th larva with vestigial tubercle of right prothorax	0	1	1	83.33%	D: 4; I: 15.
Type4	mutated 4th larva with vestigial tubercle of right metathorax	0	1	1	23.07%	D: 5-18; M: 5.
Type5	no obviously morpholgocial mutation	121	92	213	NA	NA

D: delete fragment (bp); I: insert fragment (bp); M: mutated fragment (bp). NA: not applicable.

Supplementary Table 51. Potential off-target numbers identified by different methods for eight target sites of *Abd-B*, *ebony*, and *frizzled* genes in *P. xuthus* (*Px*).

Target Gene	Target Name	Method for off-target identification	Number of off-targets
Px_03961_Abd-B	T42	CasOT	19
		Cas_OFFinder	27
		COSMID	0
		**Total**	**30**
	T95	CasOT	98
		Cas_OFFinder	125
		COSMID	2
		**Total**	**135**
Px_01073_e	T2	CasOT	80
		Cas_OFFinder	158
		COSMID	3
		**Total**	**201**
	T303	CasOT	36
		Cas_OFFinder	77
		COSMID	0
		**Total**	**72**
	**T454**	CasOT	104
		Cas_OFFinder	140
		COSMID	4
		**Total**	**143**
	**T6**	CasOT	42
		Cas_OFFinder	54
		COSMID	1
		**Total**	**54**
Px_15230_fz	T268	CasOT	136
		Cas_OFFinder	218
		COSMID	12
		**Total**	**225**
	T283	CasOT	56
		Cas_OFFinder	85
		COSMID	1
		**Total**	**90**

Parameters for different methods: (1) CasOT: mismatch: ≤5 bp (≤3 bpin 12 bp of seed region); insertion: NA; deletion: NA; PAM: NGG or NAG. (2) Cas_OFFinder: mismatch: ≤5 bp (in 20bp of non-seed and seed region); insertion: NA; deletion: NA; PAM: NGG or NAG. (3) COSMID-like: 1) mismatch: ≤3 bp; insertion: 0; deletion: 0; PAM: NGG or NAG. 2) mismatch: ≤2 bp; insertion: 1 bp; deletion: 0; PAM: NGG or NAG. 3) mismatch: ≤2 bp, insertion: 0; deletion:1 bp; PAM: NGG or NAG.

Supplementary Table 52. Summary on SNV or indel frequencies detected at theon- or off-target sites using whole genome sequencing.

Target	Number of mismatch (Indel) in potential off-target*	Number of potential off-target identified**	Number of potential off-target sites with SNV or indel detected by whole genome sequencing***	Number of off-targets locating in coding region****
Px_01073_T454	0	1 (on-target)	1	1 (*Px_01073_e*)
	1	0	0	0
	2(1)	3(1)	0	0
	3	2	0	0
	4	6	1(0, 1)	0
	5	132	25(22, 3)	2
	**Subtotal**	**143**	**26 (22, 4)**	**2**
Px_01073_T6	0	1 (on-target)	1	1(*Px_01073_e*)
	1	0	0	0
	2(1)	1(1)	0	0
	3	0	0	0
	4	2	0	0
	5	51	7 (7,0)	1
	**Subtotal**	**54**	**7 (7, 0)**	**1**
**Total**		**197**	**33 (29, 4)**	**3**

*The number in bracket shows indel base number;

** The number in bracket denotes identified off-target number with one indel base;

***The number in bracket denote (the site number only with SNV detected, the sites number with INDEL detected);

*****The three genes including potential off-targets are due to one synonymous SNV.

Supplementary Note 1. Genome sequencing and *de novo* assembly

Library construction and genome sequencing strategy.
For each of the two species, we first constructed and sequenced short-insert libraries. The pilot assemblies using short reads of 250 base pairs (bp) and 500 bp libraries suggested that their high heterozygous genomes (**Supplementary Figure 2)** hindered the assemblies **(Supplementary Table 1).** Considering the possibilities of long inbred time to get homogenous iso-female line, labor- and time-expensive nature of BAC-to-BAC or fosmid-pooling sequencing methods, which are usually alternative strategies for sequencing and assembling complex genomes[28, 37, 46, 58, 59], we adopted Roche 454FLXPlus technology to add longer reads for helping assembly[60, 61].

For short-insert libraries of Illumina sequencing, 5 µg of DNA was sheared to fragments of 180-800 bp, end-repaired, A-tailed and ligated to Illumina paired-end adapters (Illumina, San Diego, USA). The ligated fragments were size selected at 180 bp, 350 bp and 800 bp on agarose gel and amplified by LM-PCR to yield the corresponding short-insert libraries. For long-insert library construction of Illumina sequencing, 20-40 µg of genomic DNA was sheared to the desired insert size using nebulization for 2 Kilobase (Kb) or HydroShear (Covaris, Woburn, MA) for 5 Kb, 10 Kb and 20 Kb. The DNA fragments were end-repaired using biotinylated nucleotide analogues, size-selected at 2 Kb, 5 Kb, 10 Kb and 20 Kb, and circularized by intramolecular ligation. The circular DNA molecules were sheared with Adaptive Focused Acoustic (Covaris) to an average size of 500 bp. The biotinylated fragments were purified with magnetic beads (Invitrogen, CA, USA), end-repaired, A-tailed and ligated to Illumina paired-end adapters, size-selected again and purified by LM-PCR.

Sequencing data and quality control (QC).
The raw single end reads from 454 platform were converted to fastq format by sff_extract_0_2_8.py (http://bioinf.comav.upv.es/sff_extract/) and reads less than 500 bp were removed. We evaluated per base sequence quality (**Supplementary Figure 3**) and sequence length distribution (**Supplementary Figure 4**) with FastQC (http://www.bioinformatics.babraham.ac.uk/projects/fastqc/). More than 3 million reads were obtained from 454 sequencing platform with mean length longer than 700 bp for *Px* (2.4 Gigabase (Gb), 9.6 ×) and *Pm* (2.6 Gb, 8.4 ×), respectively (**Supplementary Table 2**). For the Illumina sequencing data from libraries with insert size of about 150 bp, 250 bp, 500 bp, 2 Kb, 5 Kb, 10 Kb and 20 Kb, the low quality bases with the average Phred score at the position less than 7 for each library were clipped at the end of reads. Adapters, low quality and duplicated reads were filtered out as follows: (1) the reads with a quality score less than 7 of 40% bases or with N of 10% bases; (2) the reads with the adapter sequence of >10 bp (allowing ≤ 2 bp mismatches); (3) the small insert size paired-end reads that overlapped ≥ 10 bp between the two ends; (4) the read 1 and read 2 of two paired-end reads that were completely identical (considered to be the products of PCR duplication). Finally, 46.35 Gb (194 ×) and 42.08 Gb (140.51 ×) of clean data areyielded from Illumina sequencing platform for *Px* and *Pm*, respectively (**Supplementary Table 2**).

Estimation of genome features from *K-mer* analysis.

The Illumina short reads from 250 bp and 500 bp libraries are used for *K-mer* frequency analyses of two *Papilio* genomes (**Supplementary Table 3**). For a diploid genome without repetitive elements and heterozygosis, the relationship between *K-mer* frequencies and sequence depth follows Poisson distribution[62]. The frequency of each *K-mer* can be calculated from genome sequence reads. A *K-mer* is an artificial sequence division iteratively from sequencing reads into pieces of K bases. As the length of each *K-mer* is K bp, a read with L bases contains (L-k+1) *K-mers*. A bimodal distribution is observed for both *Pm* and *Px* in which the depth of the first peak is about half of that of the second peak (**Supplementary Figure 2**). In bimodal model, the first peak repersents the depth of heterozygosity, and the second peak is the average depth of *K-mer*. Thus, the *K-mer* data suggest that the two genomes are highly heterozygous. We further used the Illumina short reads from 250 bp libraries to calculate the level of heterozygosity of these two butterfly genomes, which (*Px*: 1.008%; *Pm*: 1.229%) are twice as that of monarch butterfly (0.55%) [33]. *K-mer* analysis can also be used to calculate a genome size (Gb) by the formula of G=K_num/K_depth, where the K_num is the total number of *K-mer* and K_depth is the average depth of *K-mer*. The genome sizes of *Px* and *Pm* are estimated as about 226 Megabase (Mb) and 243 Mb, respectively (**Supplementary Table 3**).

Short reads assembly and hybrid assembly.

We carried out several rounds of assemblies using the data from different sequencing platforms, i.e, Illumina short reads, 454 long reads, or combined both. Firstly, we used Illumina short reads to do *de novo* genome assemblyusing SOAPdenovo[63] (http://soap.genomics.org.cn). SOAPdenovo is based on the *de Bruijn* graph algorithm in order to both simplify the assembly and reduce computational complexity. The assembly from illumina reads produced a mass of contig sequences longer than 100 bp (*Px*: 616,620; *Pm*: 500,090) with very short contig N50 (*Px*: 501 bp; *Pm*: 777 bp) for both *Px* and *Pm* (**Supplementary Table 1**), which suggest that short reads only are powerless when used for assembling such complex genomes as *Papilio* butterflies, as found in the oyster genome of the high heterozygosity[46]. Next, we carried out a *de novo* assembly based on only 454 long reads using Newbler 2.6[64] in DataAnalysis_2.6 toolkit with options "-mi 90 -ml 40 -nrm -het -m -cpu 20 -l 500" to build contigs. The assembly of 454 long reads provided an improved result with longer contig N50 for both *Px* (5.6 Kb) and *Pm* (1.9 Kb) than those of Illumina data assembly (**Supplementary Table 1**), which, however, is still far shorter than that of a reliable assembly. In order to evaluate the effects of read length on assembly, we also made a simulating assembly of *Px* by splitting 454 long reads into such short reads as 225 bp, 377 bp and 754 bp. The contig N50 of *Px* simulating assemblies from different reads of 225 bp,377 bp and 754 bp are 1.1 Kb, 4.7 Kb and 5.6 Kb (**Supplementary Table 4**), which suggest the longer reads indeed contribute to an assembly of a higher quality. The final assembly was a hybrid assembly combining Roche 454FLXPlus long reads with Illumina short reads (**Supplementary Figure 5)**.. Hybrid assembly method of combining Illumina short reads with 454 long reads produced high quality assemblies of both *Px* and *Pm* (**SupplementaryTables 6-7**), but also resulted in larger genome sizes (*Px*: 236 Mb; *Pm*: 294 Mb) than those (*Px*: 226 Mb; *Pm*: 243 Mb) estimated by *K-mer* analyses **(Supplementary Table 3)**.

Genome size estimation by flow-cytometry.
In order to evaluate our current assemblies, we estimated genome size using flow-cytometry after Bennett et al.[65] and Jiggins et al.[66]. The brain tissue of single adults of *Px* and *Pm,* the heads of 20 adults of *Drosophila melanogaster* (*Dm*) standard were added into 200 µl of cold Galbraith buffer[67] in a 1.5 ml Pellet pestles (Sigma) issue grinder, stroked 40 times with a pestle, and then added into cold Galbraith buffer to get a final volume of 500 µl for two butterflies and 1500 µl for fruitfly. In total, we prepared cell suspension from 10 individuals (5 males and 5 females) of *Px* and *Pm* as biological replicates, respectively. Finally, the above three kinds of cell suspension were filtered through a 20 mm nylon filter. We also prepared the blood of the domestic chicken (*Gallus domesticus*: *Gd*) as an alternative standard. In the case of *Dm* as standard, 35 µl *Dm* cell suspension were added into 1.5 ml eppendorf (EP) tubes containing 200 µl cell suspension of *Px*, *Pm* or *Gd*. In the case of *Gd* as standard, 15 µl chicken blood was added into 1.5 ml EP tubes containing 200 µl cell suspension of *Px*, *Pm* or *Dm*. Propidium iodide was added into a final concentration of 50 part permillion (ppm), and the mixture was co-stained in the dark at 4°C for 30-40 min. The fluorescence of co-stained nuclei for each sample was quantified three times using an Accuri C6 flow cytometer (BD, USA) with a laser tuned at 488 nanometers (nm). Fluorescence was detected by a photomultiplier screened by a long-pass filter. DNA content (pictogram: pg) was determined by comparing the ratio of the 2C mean of the sample with the 2C mean for *Dm* (1 C = 0.18 pg) or for chicken (1 C = 1.25 pg)[65, 67]. Genome size (bp) were calculated from DNA content (pg) following the formula[68]: genome size (bp) = (0.978 × 109) × DNA content (pg). According to this formula, *Dm* and *Gd* genome size are 176 Mb and 1222.5 Mb.

Both male and female of *Px* and *Pm* have almost equal genome size but the estimated genome size is variable with different size standard (**Supplementary Table 5)**. In the case of *Dm* as standard, genome size were underestimated because the estimated size of well-known chicken (1119 Mb) is less than reported (1222.5 Mb); in contrast, *Gd* standard resulted in overestimation for the estimated size of *Dm* (194 Mb) are larger than the reported (176 Mb). Underestimation of genome size in case of *Dm* standard (176 Mb) and overestimation in case of *Gd* standard (1222.5 Mb) are also found in *Arabidopsis* genome size measurement by flow cytomery[69]. Thus, we conclude that the genome size of *Px* and *Pm* should be 218-238 Mb and 234-256 Mb , respectively, which are near those estimation by *K-mer* analyses (*Px*: 226 Mb; *Pm*: 243 Mb) (**Supplementary Table 3**). The data suggest that the *Px* assembly (236 Mb) accords with the experimental value, but the *Pm* assembly (294 Mb) is far larger than the measurement. The genome sizes of these two *Papilio* butterflies are more similar to those of *Heliconius melpomene* (*Hm*) (292 Mb) [66] and *Danaus plexippus* (*Dp*) (0.29 pg, 284 Mb)[70], but are half than those of two other *Papilio* butterflies (*P. canadensis* and *P. glaucus*: 0.44 pg)[70].

Haplotype separation and the exclusion of microsporidium genome.
Because the assembled size (294 Mb) of *Pm* is far larger than its C-value (234-256 Mb), we firstly supposed that some divergent haplotype copies of homologous chromosomal regions remains. To deal with it, we aligned the *Pm* assembly to itself by LASTZ[71], and we found that 18.5 Mb scaffolds could be aligned to another longer scaffold with identity greater than 90 and coverage greater than 90%, which might be the candidate of

redundant regions. Then we checked the coverage depth by mapping Illumina reads to those of scaffolds and discarded those scaffolds with coverage less than 23 × (the main depth of Illumina is 46 ×) and no genes located on. For the scaffolds with genes, we BLASTPed[72] those genes against the left genes in *Pm* and we discarded those scaffolds with all the located genes mapped to the other scaffolds with gene sequence identity greater than 98%. In total, 35,985 scaffolds summing up to 15.6 Mb with an average length of 432 bp were separated from original assembly. Then we found that some proteins in *Pm* have best hits to microsporidia *Nosema ceranae* and *N. bombycis* when we aligned the *Pm* proteins to GenBank, which indicates some contamination in *Pm* assembly. *N. ceranae* and *N. bombycis* are two species of microsporidia which most infect insects such as honeybee, silkworm, etc. In order to remove mirosporidium contamination from original assembly, we aligned *Pm* proteins by BLASTP[72] to the proteins of *Px*, *Bm*, *Hm*, *Dp*, *Microsporidia* (from GenBank) and *N. bombycis* (http://microbe.swu.edu.cn/silkpathdb/node/5). If the best hit of a *Pm* protein was from *Microsporidia,* we defined the protein as a contaminated one. Furthermore, we validated those proteins on scaffolds: if 90% of the genes on one scaffold were contaminated, the scaffold was then deleted from *Pm* original assembly. In total 965 proteins were defined as contaminated proteins and7.7 Mb sequence were deleted. Besides, we also made an alignment of *Pm* original assembly to *N. bombycis* genome sequence using BLASTN[72]. We discarded the scaffold if greater than 70% of it (experience value from contaminated scaffold by protein alignment) was mapped to *N. bombycis* sequences and no gene existed on it. After haplotype separation and the exclusion of mirosporidium genomes, we got a final assembly of 265 M contigs and 281 M scaffolds, which are consistent with the genome size estimated by flow cytometer (234-256 Mb); the N50 of *Pm* final assembly is 81 Kb for contig and 1.15 Mb for scaffold (**SupplementaryTables 6-7**).

From *Px* original assembly, the same method of haplotype separation and the exclusion of microsporidium genomes as that for *Pm* were used. 4.9 Mb haplotype sequence was separated, and no contamination scaffold was found in *Px* original assembly. Thus, the *Px* final assembly is 231 Mb contigs and 244 Mb scaffolds, which are compatible with the genome size estimated by flow cytometer (218-238 Mb); the N50 of *Px* final assembly is 492 Kb for contig and 3.4 Mb for scaffold (**Supplementary Tables 6-7**).

Assembly quality evaluation.

Align short reads to assemblie. To assess the assembly quality, the Illumina reads from short insert size libraries with high quality were aligned to the assemblies using BWA[73] with default parameters. For *Px*, 95.82% of the reads could be aligned to the assembled genome and 98.04% of the assembly could be covered by reads. For *Pm*, the alignment ratio and coverage are 88.39% and 95.12%, respectively. Considering the high heterozygosity of *Pm* genome, its low mapping rate may be owing to the genetic diversity among sequenced individuals, since data generated by 454 and Illumina platforms are from different individuals, and the assembled sequences are majorly contributed by 454 data while Illumina data manly provides paired-end information for scaffolding. For further validation, we split the 454 reads into non-overlapping pieces with a size of 50 bp and then mapped onto the assembly using BWA with same parameters. And the alignment ratio and coverage rose to 93.69% and 96.94%, respectively. This analysis suggests both assemblies cover over 90% of the genome (**Supplementary Table 8)**. The depth curves

plotted based on the reads alignment show unimodal distribution (**Supplementary Figure 6**) with a peak consistent with theoretical Possion distribution suggesting good randomness of our data and no obvious genome-wide error in our assemblies.

Align transcripts to evaluate completeness of assemblies. To evaluate the completeness of the coding regions in the assemblies, RNA-seq libraries generated from male pupae were sequenced and the reads were assembled *de novo* (see **Supplementary Note 6**). We aligned transcripts longer than 200 bp to *Papilio* genomes using BLAT. 96.67 % (*Pm*) and 99.40% (*Px*) of the transcripts longer than 1000 bp can be aligned to the assembled genomes with at least 90% of aligned length (**Supplementary Table 9**). The results demonstrated that our assemblies are of high coding region coverage.

Evaluate assemblies' completeness by CEGM. To address whether or not one can identify conserved genes in assemblies, we used another method for gene space assessing that utilizes the CEGMA (Core Eukaryotic Genes Mapping Approach)[74] (http://korflab.ucdavis.edu/Datasets/cegma/) mapping protocol to map a set of highly conserved eukaryotic genes. Here, 248 CEGs (Core Eukaryotic Genes) that are generally present in low copy numbers are mapped against the assembled genomes to provide a rough approximation for the proportion of all known genes that may be present. 227 and 236 CEGs are completely identified by CEGMA for *Pm* and *Px*, respectively (**Supplementary Table 10)**; the numbers including partially identified CEGs are 240 and 245. For CEGs unfound by CEGMA, we used TBLASTN and GeneWise[75] for prediction and then manually checked the results. Finally, 247 out of 248 CEGs are identified in both *Pm* and *Px* assemblies (**Supplementary Table 11)**. For only one CEG (KOG2311) unfound in *Papilio* assemblies, we furthermore predicted it using GeneWise in the genomes of *Dm*, *Bm*, *Hm*, and *Dp*, and only found a validated copy in *Dm*. Thus, we concluded that KOG2311 had lost in five Lepidoptera insect species. Our data demonstrate that the assemblies of *Px* and *Pm* are complete.

Supplementary Note 2. Chromosomal scaffolding using a RAD linkage map in *Px*

Sample and RAD library preparation, sequencing and marker extraction.
All 94 larvae were carefully dissected to remove internal plant fragments. A standard phenol-chloroform protocol was used to extract genomic DNA from 96 samples (two adults and 94 larvae) and one more whole genome amplification step was performed on those genomic DNA extracted from 23 2nd instar larvae using REPLI-g Mini Kit (Qiagen). RAD library preparation was performed mainly according to Etter et al.[76] with some modifications: 4 different P2 adapters and 12 different P1 adapters with an AATT (*EcoRI*) overhang were designed to multiplex up to 48 samples in a lane. Prepared RAD libraries were validated by cloning and Sanger sequencing for barcodes and DNA source check. 96 indexed RAD samples were pooled in 2 lanes and sequenced with an Illumina Hiseq 2000. 154,821,324 100 bp read pairs were sequenced, 1,367,693 of which were discarded due to low quality (**Supplementary Table 13**). The genome scaffolds contained 55851 *EcoRI* sites so the expected RAD tags should be around 110,000. RAD-tag sequencing

data from 20 progeny were discarded due to very low coverage and the rest were aligned to *Px* genome scaffolds using Bowtie2 v2.1.0[77] and then re-ordered and sorted by Picard v1.84 (http://picard.sourceforge.net). RealignerTargetCreator and IndelRealigner[78] in GATK v2.1 were used to realign indels and UnifiedGenotyper[79] was used to call genotypes using following parameters: heterzygosity 0.01, stand_call_conf 50, stand_emit_conf 10, dcov 250, which yielded total 1,652,231 bases across 5,350 scaffolds, covering 235.57 Mb (96.52%) of the genome. Low quality bases where any individual had Genotype Quality less than 8 or read depth less than 3 were masked. Then bases heterozygous in at least one parent and supported by more than 65 progeny were extracted, which yielded 19,291 filtered bases. Positions with identical segregation patterns across all progeny were collapsed and finally 14,740 valid markers were identified, across 162 scaffolds and covering 215.31 Mb (88.22%) of the genome.

Linkage mapping.
Because JoinMap 4.1 could only accommodate a maximum of 5,000 markers, firstly we selected 4,588 markers supported by more than 70 individuals and all the markers were formatted into CP format as input to JoinMap 4.1[80]. Markers were grouped into 30 chromosomes at LOD>4.0 and for each group, linkage maps were constructed using both regression (Kosambi mapping function) and maximum likelihood algorithms. After this step, 4,255 markers across 103 scaffolds were mapped. In order to take advantage of all 14,740 markers, redundant markers were removed from major scaffolds and then the rest were added, which were grouped into 29 chromosomes (LOD>4.0) using maximum likelihood algorithm for linkage mapping. Finally 4,426 markers across 126 scaffolds were mapped to 30 chromosomes, which covered 214.82 Mb (88.03%) of the genome. Then all 3 linkage maps were taken into consideration to merge scaffolds and remove distorted markers while cM distances were mainly calculated based on the regression map.

Synteny analysis and assembly error correction.
To further guide scaffolding and rearrange distorted markers, we BLATed 126 *Px* scaffolds against *Bm* CDS regions and then only used conserved fragments (aligned length>150 bp) to target *Px* scaffolds to *Bm* chromosomes. Scaffolds from 26 *Px* chromosomes were uniquely mapped to 26 *Bm* chromosomes with some expected rearrangements except chromosome (chr) 19, 28, 26 and 29 (chr19 and chr28 were both derived from *Bm* chr11 while chr26 and chr29 were from *Bm* chr24), which provided a strong independent evidence of our linkage mapping result. Indeed, we identified some long scaffolds mapped to multiple chromosomes during linkage mapping and synteny analysis, which implies potential mis-scaffolding in longer scaffolds. We thus integrated *Px*-*Bm* synteny analysis with 3 linkage maps to correct, order and orientate mapped scaffolds and finally were able to map 1,158 markers on 30 chromosomes, covering 202 corrected scaffolds and 207.09 Mb (87.22%) of the re-scaffolded genome. The linkage map (**Supplementary Figure 7**) was presented using MapChart[81].

Syntenic relationship of chromosomal linkage between *Px* and *Pm*.
Px has 30 chromosomes, but *Pm* has 31[82], which infers the ancestral chromosome number[48]. In order to check the syntenic relationship between chromosomal linkage of *Px* and *Pm*, we first assigned *Pm* chromosome based on the synteny of 11,116 orthologs

between *Pm* and *Px* and then decided the synteny relationships according to their reciprocal best hits. We then decided the synteny of both *Pm* and *Px* against Glanville fritillary *Melitaea cinxia* (*Mc*) according to their reciprocal best hits (6,072 in *Mc* and *Px*; 5,450 in *Mc* and *Pm*). Thus, based on the synteny among *Pm*, *Px* and *Mc,* we identified chr8 in *Px* are a fusion of ancestral lepidopteran chr8 and chr31. Finally, based on 10,045 and 10,229 orthologs yielded from *Px* to *Pm* and to *Bm* respectively, we decided the syntenies between *Px* and *Pm*, *Bm* and *Pm*, and represented them by CIRCOS[83].

Supplementary Note 3. Annotation of repetitive elements and transposable elements (TEs)

We searched across the genomes using TRF (Tandem Repeats Finder)[84] to identify non-interspersed repetitive elements with default parameters. Transposable elements (TEs) were firstly predicted in the genomes by homology searches to known RepBase TE libraries (version 2011-4-19) using RepeatProteinMask and RepeatMasker[85] with default parameters. Then we constructed a *de novo* repeat library using RepeatScout[86] with default parameters and obtained consensus sequences and classification information for each repeat family. Using these RepeatScout consensus sequences as input library we again searched repetitive elements in the assemblies using RepeatMasker with default parameters. All TE were classified according to Wicker et al[87]. We employed the same pipeline to annotate repetitive elements in *Px, Pm, Dp* and *Hm* genomes and the divergence distribution of classified TE families in the genomes of four butterflies and silkworm was estimated using the methodology described in Kim et al.[88].

Using a combination of homology to Repbase sequences, *de novo* prediction approaches and TRF, we annotated repetitive sequences and TEs in *Px*, *Pm*, and two nymphids (*Dp* and *Hm*) based on their published genomes (**Supplementary Tables 14-16)**. Our two butterflies *Px* and *Pm* show very similar TE compositions in the genomes (*Px*: 21.09%; *Pm*: 22.31%), which are more similar to that of fruitfly *Dm* (~22%)[89] than to those of other Lepidopteran insects (*Hm*: 28.47% (this study) and 25%[32, 90]; *Dp*: 12% (this study) and 11%[33]; *Bm*: 35%[91]). Among them, the richest TE in *Pm* is DNA transposon with a high percentage of up to 4.71%, which is higher than in the closed related species *Px* (1.47%) and other two butterflies *Hm* (1.60%) and *Dp* (1.88%); and another TE with very high coverage (3.48%) in *Pm*, next to DNA transposon, is SINE, which is also the richest TE in *Hm* and *Dp*. On the other hand, the richest order of TE in *Px* is LTR, which is up to 3.25% and far higher than in other three butterflies.

Based on the data identified using repeatmask, we analyzed the copy numbers of different TE families. Very interestingly, the most prevalent copy of classified TEs in both *Pm* and *Px* is *Helitron*, a unique kind of DNA transposon firstly discovered in the genomes of the thale crest (*Arabidopsis thaliana*), rice (*Oryza sativa*), and worm (*Caenorhabditis elegans*)[89]. Especially in*Pm*, more than thirty thousand copies are identified and they

make up 1.21% of the genomic DNA, a ratio between 1% of *Dm*[92] and 2% of the thale crest, rice, and worm[89]. In contrast, fewer copies and very low percentage of genomic DNA are observed in other three butterflies (**Supplementary Table 16**). Our data confirm the view that genomic copy numbers of *Helitron* are highly variable even among closely related species regardless of its wide distribution in all eukaryotic kingdoms[93].

By comparing the detected TEs with consensus sequences derived from Repbase, we estimated the divergence distribution of classified TE families in all the genomes of five Lepidopteran species, including four butterflies (*Pm*, *Px*, *Hm*, *Dp*) and one silkworm (*Bm*). Our data show that the four butterfly genomes follow a roughly similar tendency and a relative diversity compared to the genome of silkworm in all four classified TEs (**Supplementary Figure 8)**.

Supplementary Note 4. Gene annotation

To identify protein coding genes in *Papilio* genomes, we integrated the annotationsyielded from homology-based methods, *ab initio* gene prediction and RNA-seq data. In homology-based prediction, proteins from bee (*Apis mellifera*: *Am)*, beetle (*Tribolium castaneum*: *Tc*), *Bm*, *Dp* and *Dm* were mapped to the *Papilio* genomes using TblastN[94]; then, homologous regions defined by TblastN were fed into GeneWise[75] to obtain gene models. For *ab initio* prediction, Augustus[95], SNAP[96] and GlimmHMM[97] were employed to predict coding genes. In addition, transcripts identified from differential developmental stages (from egg to adult) of *Pm* and *Px* were incorporated to improve the gene models.Finally, we integrated the three kinds of gene predictions to produce a comprehensive and non-redundant reference gene set usingGLEAN[98] (http://sourceforge.net/projects/glean-gene/) and further removed those Glean integrated genes with overlaps with homolog and transcript regions less than 50 % and/or with their highest expression values of RPKM in all of RNA-seq libraries less than 1. Gene features of *Pm* and *Px*, together with nymphids (*Hm*, *Dp*) and silkworm (*Bm*), were compared. In addition, we also compared paralog identity distribution of these five lepidopteran insects. Gene function information of *Pm*, *Px*, *Dp* and *Hm* were assigned based on the best hits derived from the alignments to proteins annotated in the SwissProt and TrEMBL[99] and KEGG[100] database using BLASTP[72]. Motifs and domains of proteins were annotated using InterPro [101] by searching the public databases, including Pfam, PRINTS, PROSITE, ProDom, and SMART. We aslo described gene functions via retrieving Gene Ontology (GO)[102]. In order to avoid biased comparisons of gene sets among butterflies species in this study (*Pm* and *Px*) and two former studies (*Dp* and *Hm*), we repeated all these analyses of the four butterfly genomes.

In summary, 15,499 and 15,322 genesare finally annotated in *Pm* and *Px*, respectively (**Supplementary Table 17**). In both species, more than 80% of the Glean gene sets are supported by evidences from at least two prediction methods (*ab initio*, homolog-

mapping, and RNA-seq) (**Supplementary Figure 9**), among which the genes identified by all three methods are over 50%.

We compare gene features among four butterflies and one silkworm including *Px* and *Pm* (**Supplementary Table 17** and **Supplementary Figure 10**). All the butterflies and silkworm showed a similarity in proportion of single exon genes, gene average length, the distribution of exon numbers and lengths, except that the silkworm genome seems to include higher proportion of longer introns. In addition, we found that the identity distributions of genomic paralogues in a species are very similar not only among butterflies and moths, but also among lepidopteran and dipteran insects (**Supplementary Figure 11**). The results of function annotation using different methods are shown as **Supplementary Table 18.** In total more than 80% predicted genes have been found to be functional among all the species.

Supplementary Note 5. Gene evolution

Gene family clusters.

In order to explore gene evolution among butterflies, our gene cluster analysis involves the genomes of five butterflies (*Px, Pm*, *Dp*, *Hm, Melitaea cinxia* (*Mc*)), moths (*Bm*, *Plutella xylostella* (*PLX*)), mosquito (*Anopheles gambiae* (*Ag*)), fruitfly (*Dm*), beetle (*Tc*), bee (*Am*), which cover all orders of holometabolan insects. Proteins of all genes (>50 Amino acid (AA)) for 11 species are chosen for gene family clustering using Treefam methodology[103]. We aligned all protein sequences against themselves using BLASTP[72] with the e-value cutoff of 1e-7. We assigned a connection (edge) between two nodes (genes) if more than 1/3 of the region aligned to both genes. An Hscore of similarity ranging from 0 to 100 was used to weigh the edges. For two genes G1 and G2, the Hscore was defined as a Score(G1,G2)/max(Score(G1,G1), Score(G2,G2)) (function score is raw Blast score). Gene families were clustered by Hcluster_sg. We used average distance for the hierarchical clustering algorithm, requiring a minimum edge weight (Hscore) larger than 5 and a minimum edge density (total number of edges/theoretical number of edges) larger than 1/3.

The gene family clusters by Treefam are summarized in **Supplementary Table 19**. Totally, we obtained 17,329 gene clusters. 2,963 clusters are shared among 11 species and 1,071 of them are single copy orthologs. There are 107 families only found in the five butterflies (butterfly-specific families), which include 118 (*Px*), 112 (*Pm*), 119 (*Dp*) and 117 (*Hm*), 117 (*Mc*) genes; GO enrichment analyses of the 107 butterfly-specific gene families are shown in **Supplementary Table 20**. There are 430 families only found in both *Pm* and *Px* (*Papilio*-specific gene families), which include 454 (*Pm*) and 486 (*Px*) genes (*Papilio* orphan genes); GO enrichments of these *Papilio* orphan genes are shown in **Supplementary Table 21**. In addition, we also identified 81 (*Pm*) and 101 (*Px*) singleton genes in *Pm* and *Px*, which cannot be clustered with any other copy of all 11

species. Thus, combining the genes (copy≥2) in *Pm*-specific or *Px*-specific gene families and singletons in these two species, we finally got 1510 and 1548 orphan gene in *Pm* and *Px*, respectively. Among others, 614 (*Pm*) and 950 (*Px*) have no any hit with all 11 species with BLASTP[72] cutoff 1e-7. In addition, in 17,329 of butterfly-related families, 8,532 families are shared by five butterflies (*Pm, Px, Hm, Mc* and *Dp*) (**Supplementary Figure 12**). Compared with other butterflies, there are 171 and 260 species-specific families in *Pm* and *Px*, respectively; there are 546 families specific to *Papilio* (**Supplementary Figure 12**).

Divergence time of Lepidoptera.

1071 single-copy gene clusters identified using the methodology afore-mentioned were used for constructing phylogenetic tree of 11 species. These single-copy genes for 11 species are firstly aligned by MUSCLE[69] and then linked to super-genes, and the poor alignment regions and gap are removed by the Gblocks[104] with codon model in the super-gene. The remained 825,564 positions were used to construct the phylogeny of 11 insects (bee, beetle, fruitfly, mosquito, silkworm, moth and five butterflies) using the maximum-likelihood principle[105]. The species divergence time was estimated based on 32,642 four-fold degenerate sites via Bayesian estimation approach using the program PAML:mcmctree[106]. Here we used two fossil calibrations, one for the most recent common ancestor of mosquito (*Ag*) and fruit fly (*Dm*) (238.5 to 295.4 Mya)[107] and another for the the split of Diptera and Lepidoptera with (290 to 417 Mya)[108, 109]. In addition, the evolutionary changes in the protein family size (expansion or contraction) were analyzed using the CAFÉ program[110], which assesses the protein family expansion or contraction based on the topology of the phylogenetic tree.

A phylogenetic relationship of 11 insects was constructedand their divergence time with the 95% highest posterior density (HPD) credibility intervals in brackets and the number of gene family expansion/contraction was shown (**Supplementary Figure 13)**. Based on our analyses, *Pm* and *Px* diversified 38 million years ago (mya), which is consistent with those divergent time estimation (33-36 mya) based on a combination of biogeographic time constraints[111]. Our result shows older divergent time not only between Papiliodidae and Nymphalidae (131 mya) but also between Danaine and Nymphilinae (112 mya) than the former studies[112, 113] , in which Wahlber et al proposed Nymphalidae began diversifying in the late Cretaceous around 90mya and Daninae diverged at about 89 mya. Among the families with changed size for *Pm*, *Px*, and *Papilio* clades, the significant expansion/contraction families (P<0.01) are +23/-7, +20/-7, +19/-6, respectively, with lineage-specific expansion of 16 (8-3 genes), 16 (3-22genes), 3 (9-14genes) for *Pm*, *Px*, and *Pm-Px*. The GO enrichment results of these significant expansion and constriction familiesare shown as **Supplementary Table 22.**

Orthologous relationship and DNA divergence.

To determine the orthologous relationship among Lepidopteran insects (*Pm*, *Px*, *Hm*, *Dp*, *Bm*), we concatenated all proteins for each of the four butterflies plus silkworm, and then performed all-against-all alignment using BLASTP[72] with an e-value cutoff of 1e-5. Reciprocal best hit protein pairs among these taxa were defined as orthologs with both align Rate (aligned length/query length) and align identify of greater than 10%.

The ortholog numbers among lepidoptera insects are shown in **Supplementary Table 23.** As expected, the highest number of orthologs (11,784) was found between the two most closely-related taxa *Pm* and *Px*; moreover, the orthologous gene numbers between *Pm* and the other three Lepidopteran *Dp*, *Hm*, *Bm* are almost equal to the counterparts between *Px* and *Dp, Hm, Bm,* which suggests that more closely-related taxa have more similar orthologs. In our case, orthologs identity between *Pm* and *Px* is significantly higher than those between the other species pairs; and the identity between *Papilionidae* (*Pm* and *Px*) and *Nymphalidae* (*Dp* and *Hm*) is also slightly higher than that between butterfly (Papilionidae) and silkworm (*Bm*) (**Supplementary Figure 14)**.

We further analyzed DNA divergence in coding regions and in the whole genome between *Pm* and *Px*. We firstly aligned coding regions of orthologous genes using BLAT, and then linked all alignments to one sequence for each species. For the whole genome DNA divergence, we used LASTZ to align scaffolds between *Pm* and *Px*, and then alignments were linked to one sequence for *Pm* and *Px*. According to the alignments, DNA divergence was calculated using distmat in EMBOSS-6.5.7 with do Jin-Nei correction method[114]. DNA divergences between *Pm* and *Px* are 8.79 % and 21.57 % in coding sequence and in the whole genome, respectively.

Detection of positively selected genes.
All orthologs in *Pm* and at last 5,330 orthologs in five insects were aligned using Prank of in Guidence pipeline[115], which can improve the performance of positive selection inference by filtering unreliable alignment region[116]. Codeml in the package of PAML was used to detect positive selections with branch model. Finally, 255 genes in *Px* (**Supplementary Table 24)** and 265 genes in *Pm* (**Supplementary Table 25)** were identified under positiveselection, and 63 orthologous genes (**Supplementary Tables 24-25)** are positively selected inboth *Pm* and *Px*. The GO enrichment of these positively selected genes is shown as **Supplementary Table 26.**

Supplementary Note 6. Transcriptome sequencing, assembly and analysis

RNA isolation, library construction and transcriptome sequencing.
In order to assist assembled genome evaluation, gene prediction, annotation and developmental studies, transcriptomes of different development stages of *Px* and *Pm* were sequenced. Total RNA was extracted using the guanidinium thiocyanate-phenol-chloroform extraction method (Trizol, Invitrogen) according to manufacturer’s protocol. RNA sequencing libraries were generated using Illumina mRNA-Seq Prep Kit. Briefly, oligo (dT) magnetic beads were used to purify polyA containing mRNA molecules which were further fragmented and randomly primed during first strand synthesis by reverse transcription. This procedure was followed by second-strand synthesis with DNA polymerase I to create double-stranded cDNA fragments. The double stranded cDNA was subjected to end repair by Klenow and T4 DNA polymerases and A-tailed by Klenow

lacking exonuclease activity. Ligation to Illumina Paired-End Sequencing adapters, size selection by gel electrophoresis and then PCR amplification completed library preparation. The 200 bp insert size paired-end libraries were sequenced using Illumina HiSeq 2000 sequencers with read length of 90 bp[117].

Transciptome assembly and abundance of annotated genes.
We produced more than 4.5 Gb reads for each sample (**Supplementary Table 27**). For RNA-seq libraries with the insert size of 200 bp, low quality and duplicated reads were removed with the methods mentioned above (see **Suppementary Note 1**). We assembled RNA-seq reads using two methods. First, we *de novo* assembled clean RNA-seq reads from one male pupa by using SOAPtrans (http://soap.genomics.org.cn/SOAPdenovo-Trans.html), which aims to evaluate the completeness of coding regions of the assemblies (see **Suppementary Note 1**). In total, the transcripts longer than 500 bp and 1000 bp are 21,941 and 12,388 in *Px*, as well as 21,787 and 12,505 in *Pm* (**Supplementary Table 9**). Second, RNA-seq reads from a series of developmental stages were assembled by mapping against the assembled genome using TopHat[118], which can align reads across splice junctions. Then, the TopHat mapping results were combined together and Cufflinks[119] was used to predict transcript structures. In total, 11,068 and 10,033 transcripts were identified to support gene model in *Pm* and *Px* (**Supplementary Figure 9).**

In RNA-Seq experiments, RNA-Seq fragment counts can be also used as a measure of relative abundance of genes. To estimate gene abundance, the reads from differential developmental stages were aligned against the genomes using TopHat (version 1.3.1). For most of the samples the ratio of read alignment is greater than 75% (**Supplementary Table 27**). We measured gene expression abundance of all annotated genes (*Pm*: 15,499; *Px*: 15,322) in different developmental stagesin RPKM (Reads Per Kb per Million reads)[120] and normalized the effects of different gene length and different total mapped reads among samples. These RPKM values were used in the analyses of expression pattern.

Analysis of expression dynamics during development.
Gene expressions in all developmental stages were clustered by Cluster[121] (http://bonsai.hgc.jp/~mdehoon/software/cluster/software.htm#ctv), and stage clustering was done using heat map[122] function with Pearson correlation in R package. For homologous genes in *Pm* and *Px*, expression were conjoined and clustered.

Expression clustering of all genes indifferent developmental stages of *Pm* and *Px* and their stage clustering based on Pearson correlation in any two stages are show in the **Supplementary Figures 15.** The numbers of stage-specific genes are shown in the **Supplementary Table 28.** Expression clustering of homologous gene between *Pm* and *Px* is shown as **Supplementary Figure 15**, and stage clustering of *Pm* and *Px* based on Pearson correlation of homologous gene expression in any two stages is shown as **Supplementary Figure 16**.

Gene differential expression analysis.
Detection of differentially expressed genes was based on Poisson distribution[123] and

normalization[124] for differences in RNA output sizes and sequencing depth between samples. When comparing differential expressions among multiple samples, we used P-value to test statistical significance and FDR (false discovery rate) to determine the threshold of P-value in multiple tests. Criteria “FDR ≤0.001” and “absolute value of log2Ratio≥1” were used to determine if the differences are significant.

Differential expression genes in the correspondent developmental stage of *Pm* and *Px* and the stage number of their differential expression are shown as **Supplementary Table 29.** Totally, 7,905 orthologous pairs of *Pm* and *Px* show differential expressions of less than two-fold in at least one stages. Among others, there are 32 orthologous pairs with differential expressions in all developmental stages of *Pm* and *Px*. It is very interesting that 15 orthologous pairs are up-regulated in *Pm*, and the other 18 pairs are up-regulated in *Px* (**Supplementary Figure 17**), which may play a general role in species divergence. In addition, 1,903 pairs are differentially expressed by greater than two-fold in only one stage (**Supplementary Tables 29-30**), among which, 105 gene pairs show differential expressions by larger than 10-folds, which may contribute to the traits of that stage.

Expression level of positively selected genes.

Heat map of expression of 265 positively selected genes in *Pm* and 255 positively selected genes in *Px*, together those of their othologs in *Px* and *Pm*, respectively, were analyzed **(Supplementary Figures 18a-b)**. In addition, we also presented a separated heatmap of 63 pair orthologous genes selected in both *Pm* and *Px* (**Supplementary Figure 18c)**.

Developmental expression pattern of lineage-specific genes.

Among *Papilio* orphan genes (*Pm*: 454; *Px*: 486) of *Papilio*-specific gene families, 449 orthologous pairs were identified. In addition, 1,548 and 1,510 orphan genes are identified in *Px*and *Pm*, respectively.The expression heat maps of these orthologs and orphan genes are shown in **Supplementary Figure 19.**

Supplementary Note 7. Farnesyl pyrophosphate synthase (FPPS) and other enzymes in the pathway of juvenile hormone (JH) metabolism

We identified an intriguing expanded family with functional annotations related to farnesyl pyrophosphate synthase (FPPS) (= Farnesyl diphosphate synthase) in isoprenoid biosynthetic pathway. FPPS, together with geranyl diphosphate synthase (GPPS) (= dimethylallyltransferase) and geranylgeranyl diphosphate synthase (=geranylgeranyl pyrophosphate synthase) (GGPPS), belong to the short-chain isoprenyl diphosphate synthases (scIPPS) or prenyltransferases in the isoprenoid biosynthesis pathway (**Supplementary Figure 20)**, which play a key role in insect development, reproduction, and behavior[125]. These scIPPS catalyze condensation of isoprenyl diphosphate (IPP, C5) with an allylic cosubstrate (i.e. dimethylallyl diphosphate: DMAPP, C5), geranyl

diphosphate (GPP, C10), or farnesyl diphosphate (FPP, C15), to produce GPP, FPP, or GGPP (geranylgeranyl diphosphate, C20). GPP, FPP and GGPP are the precursors of semiochemical compounds mono-, sesqui- and diterpenes, respectively[126, 127]. FPP is an indispensable precursor for the biosynthesis of juvenile hormone (JH) in insects[1, 128]. FPP and GGPP are also the precursors for several classes of essential isoprenoid-derived metabolites such as steroids, cholesterol, farneylated/geranylgeranyllated proteins, Heme A, Vatamin K2, carotenoids, chlorophylls, dolichols, ubiquinones and long chain isoprenoids[127, 129]. In order to explore the possible significance of unexpected expansion of FPPS in *Papilio* butterflies, we investigated all genes encoding scIPPS and other enzymes in the pathways of JH biosynthesisand degradation as well as protein prenyltransferases in ten insect species.

Identification of genes in the pathway of JH metabolism.
We searched and downloaded all the sequences of GPPS (EC 2.5.1.1), FPPS (EC 2.5.1.10), GGPPS (EC 2.5.1.29), and FGPPS (EC 2.5.1.81) from GenBank, and then aligned all the protein sequences inthe ten insects against downloaded sequences using BLASTP[72] with an e-value cutoff of 1e-5. We also further investigated the scIPPS of another three hymenopteran insects (wasp: *Nasonia vitripennis*; ants: *Camponotus floridanus*, *Harpegnathos saltator*). The primary searched gene-sets were further aligned against nr database of GenBank to confirm functional annotation of the scIPPS and were classified into correspondent category of scIPPS. For the identification of genes encoding other enzymes in the pathways of JH biosynthesis and degradation as well as protein prenyltransferases, we searched and downloaded all the sequences of the enzymes reported in *Bm* and *Dm* from GenBank, and then aligned all the protein sequences in the ten insects against downloaded sequences using BLASTP[72] with an e-value cutoff of 1e-5.

The copy numbers of all the genes encoding enzymes in the pathway of JH metabolism and the classifications of all the identified scIPPS are shown in **Supplementary Tables 31-32**.

Phylogenetic analysis of scIPPS genes, JHEH and JHDK genes.
All phylogenetic trees of scIPPS, JHEH and JHDK genes were constructed using Maximum likelihood method in PAML package[106]. In order to compare the currently identified scIPPS of 10 insects with those cloned and characterized, 24 published cloned scIPPS sequences in GenBank from 1 fruitfly *Drosophila melanogaster* (*Dm.*)[130, 131, 132], 4 moths (*Agrotis ipsilon* (Ai.), *Mythimna unipuncta* (*Pseudaletia unipuncta*) (Mu.), *Choristoneura fumiferana* (Cf.), *Bombyx mori* (*Bm.*)[131, 132, 133, 134], 3 beetles (*Anthonomus grandis* (ANg.), *Ips pini* (Ip.), *Phaedon cochleariae* (Pc.))[135, 136, 137], 3 aphids (*Myzus persicae* (Mp.), *Rhopalosiphum padi* (Rp.), *Acyrthosiphon pisum* (Ap.))[126, 138, 139], 2 termites (*Nasutitermes takasagoensis* (Nt.), *Reticulitermes speratus* (Rs.)))[140, 141] were also included in the phylogenetic analysis. All the sequence name of scIPPS genes identified in the genomes of 10 holometabolous insects are initially labled as in bracket: 4 butterflies: *P. xuthus* (Px), *P. machaon* (Pm), *Heliconius melpomene* (Hm), *Danaus plexippus* (Dp); 2 moths: *Bombyx mori* (Bm), *Plutella xylostella* (CCG); 1 fruitfly: *Drosophila melanogaster* (CG);1 mosquito: *Anopheles gambiae* (AgAP); 1 bee: *Apis mellifera* (GB); 1 beetle: *Tribolium castaneum* (TC)).

Diversity of FPPS/GGPPS genes in butterflies may be related to JH diversity in Lepidoptera, and further to butterfly unique traits, given that FPP is the precursor of JH, a key regulator of insect development and reproduction in conjunction with ecdysone. Most insects produce only one (JHIII) of six different chemical forms of JH (JHIII: C16-JH; JHII: C17-JH; JHI: C18-JH; JH 0: C19-JH; 4-CH3-JH I: Me-C19-JH; JHB3: bisepoxide form of JHIII) so far identified in Insecta, but the Lepidoptera produce another four derivatives featuring ethyl branches (JH II, JH I , JH 0 , 4-CH3-JH I) and the biogenesis of these ethyl-branched JHs requires the synthesis of ethyl-substituted farnesyl diphosphate (FPP) by FPPS[134].The relative level of each JH is modulated during development of tobacco hornworm *Manduca sexta* with its embryos containing JH 0,4-methyl JH I and JH I, larvae containing JH I and I1, and adults containing JHII plus III[142, 143, 144]. The occurrence of ethy-branched JHs and their modulation in lepidopterans are related to the availability of propionyl-CoA, which can be efficiently produced in Lepidopterans by catabolism of the branched-chain amino acids (BCAA) isoleucine and valine[143, 144]. Thus, the propionyl-CoA and acetyl-CoA in mevalonate pathway of Lepidoptera produce both mevalonate and homomevalonate, which further form FPP and ethyl-substituted FPP, finally resulting in the formation of JHIII and the ethyl-branched juvenile hormones, respectively. There is a single gene encoding each enzyme involved in the mevalonate pathway portion of the juvenile hormone (JH) biosynthetic pathway in *Bm*, with the exception of farnesyl diphosphate synthase (FPPS), for which three homologs were identified[132]. Although some independent studies also indicated that FPPS purified from animals unable to inherently produce ethyl-branched JHs, can use ethyl-substituted isoprene substrates (C6) to produce the farnesyl-related precursors of the lepidopteran JHs, it is usually low efficient and needs the extended incubation, which suggests there may exist different kinds of FPPs for using ethyl-substituted FPPP to synthesize ethyl-branched JHs. Correspondent with FPPS expansion, the genes encoding JH epoxide hydrolase (JHEH) and JH diol kinase (JHDK) in the pathway of JH degradation also expanded in *Papilio* (**Supplementary Table 31** and **Supplementary Figures 21a-b)**.

scIPPS gene expression.

We checked the RPKM expression levels of all identified scIPPS (FPPS and GGPPS) in different developmental stages. Eight of 14 orthologous FPPS show over two-fold differential expression patterns in at least one stage (**Supplementary Table 33**). The difference among these FPPS genes may play an important role in diversification of the two butterflies, possibly by modulating JH form and/or the relative level of each JH, as in be related tobacco hornworm *Manduca sexta*[142, 143, 144].

Supplementary Note 8. Cytochrome P450 monooxygenases (P450s)

We identified one large family (family No.1535: 162 members) that underwent multiple expansions in *Pm*, *Pm-Px*, and lepidopteran-specific (*Pm*, *Px*, *Dp*, *Hm*, *Bm* and *PLX*) clades, and also separately expanded in the clade of bee. This family is annotated as CYP6 family of cytochrome P450 monooxygenases (P450s), one of the most abundant gene superfamilies found in eukaryotic genomes. The P450 enzymes, a diverse class of enzymes found in virtually all insect tissues, fulfill many important tasks, from the synthesis and degradation of ecdysteroids and juvenile hormones to the metabolism of foreign chemicals of natural or synthetic origin[145]. P450s occupy a position of unique importance in the evolution of interspecies adaptive strategies due to their extraordinary versatility[146]. The phenomenon of P450 induction by chemicals (xenobiotics, plant chemicals, fungal metabolites, hormones) is well documented and a general observation is that herbivorous insects fed different host plants show markedly different P450 level[145]. In order to explore the evolution of P450 superfamily in *Papilio* butterfly and adaptive significance of CYP6 subfamily, we identified all P450 members in *Pm*, *Px* and *Hm* (as an outgroup). Furthermore, we also identified all the members of CYP6 family in *Pm*, *Px*, *Hm*, *Dp*, *Bm* and *Dm*.

Identification and classification.

By searching all P450 IPR (IPR001128, IPR002397, IPR002401, IPR002402, IPR002403, IPR002974, IPR008066, IPR008067, IPR008067, IPR008068, IPR008069, IPR008070, IPR008071, IPR008072, IPR020469), we identified all the P450 members in *Pm*, *Px* and *Hm*. Then we downloaded the amino acid sequences of all P450s in silkworm (*Bm*), fruitfly (*Dm*), and *Dp* from P450 homepage (http://drnelson.uthsc.edu/CytochromeP450.html)[55] and in other arthropods from GenBank. Then we aligned all these P450 protein sequences in*Pm*, *Px*, *Hm* to those downloaded ones using BLASTP[72], and then according to the rule of the best hit, classified the P450 sequences into the correspondent groups.

The total numbers of P450 in *Pm*, *Px* and *Hm* are 107, 94, and 119 **(Supplementary Table 34),** respectively, each of which is more than those reported in *Dp*, *Bm* and *Dm*[55]; the members of CYP6 family take up a higher coverage in *Pm* and *Px* than in *Hm*, *Dp*, *Bm*, and *Dm*; 50% of CYP6 belong to CYP6B subfamily in *Pm* and *Px*, while there is no CYP6B found in nymphid butterflies *Hm* and *Dp* (**Supplementary Table 35**); CYP6AB subfamily, a subfamily only reported in Lepidoptera so far, is a little expanded in *Pm*. CYP6, together with CYP9 and alpha esterase families, are thought to contribute xenobiotic detoxification in insects[147]. CYP6B isolated from many larval Lepidoptera such as the *Helicoverpa*/*Heliothis* complex and *Papilio* species[148] are among the best characterized allelochemical-metabolizing P450s[149]. Significant expansion of CYP6B subfamily in *Papilio* butterfly and a further expansion of CYP6B and CYP6AB in *Pm* suggest that CYP6B and CYP6AB play key roles in adaptive strategies of host plant specialization for *Pm* and *Px*.

Phylogeny of P450.

For all the P450 genes, coding region sequences were multiply aligned using Prank with codon model in Guidance pipeline[115]. The phylogenetic trees were constructed using

phyml in PAML package[106].

The phylogenetic trees of all the P450 members in *Pm*, *Px* and *Hm* (**Supplementary Figure 22a**), of the CYP6 members in 4 butterflies, silkworm and fruitfly (**Supplementary Figure 22b**) show that all CYP6B strongly expanded in *Papilio* clade anda further expansion of *Pm*. The phylogenetic tree of CYP6B subfamily based on all the available known 6B members is almost consistent with species phylogeny. We speculate that, within Lepidoptera lineage, a common ancestral P450 evolved to CYP46-like protein, which further diversified through time into more efficient and specialized CYP6B1- and CYP6B21-like proteins in *Pm* and *Px* with continual exposure to furanocoumarins; moreover, the different diversification of CYP6B1- and CYP6B21-like proteins in *Pm* and *Px* may specify the diversification of the host plants for these two closely related species with *Pm* using *Apiaceae* and *Px* using *Rutaceae* and other families**.** Multiple duplication and divergence events are thought to have allowed xenobiotic-metabolizing P450s, such as CYP2 and CYP3 in mammals and CYP6 in insects, to diversify and acquire new functions[149]. Specificity is maintained for the induction process and for metabolism[145]. The birth-and-death model may be particularly applicable to the diversification of P450s in herbivorous insects, which, during host shifts, encounter different selective forces associated with the biochemical defense profiles of their host plants[150]. CYP6B3, a paralog of CYP6B1, only found in *Pm*, not in *Px*, may suggest its key role for *Pm* using Apiaceae host plant. More copies of CYP6B genes may be useful to metabolize linear and angular furanocoumarins albeit at different efficiencies and provides this species with the potential to acquire new P450s with novel functions[151]. In addition to CYP6B1, we speculate that CYP6B21 also plays an important role for *Pm* and *Px* host plant use and diversification. CYP6B21 was initially cloned from *P. glaucus* (eastern tiger swallowtail) which encounters toxic furanocoumarins occasionally in its diet in its rutaceous host plants and shows some special character with its high overall identity and exon1 identity to CYP6B4 group (96.0-98.0%; 98.2-99.5%) but a higher level of identity to CYP6B17 in the highly variable C-terminal domain[152]. In *Px,* CYP6B21 may have diversified into CYP6B16, and CYP6B20, both of which exist in *P. glaucus*; in *Pm*, CYP6B21 may have diversified into CYP6B17, a P450 existing in *P. glaucus*, and CYP6B47, a membrane-p450 existing in one moth species *Spodoptera litura* and possibly metabolizing plant allelochemicals, insecticides and fatty acids in particular[153]. Although both vegetables (*Apiaceae*) as the *Pm* host plants and*Citrus* fruits (*Rutaceae*) as the *Px* host plants contain toxic furanocoumarins, it is reported that the content of furanocoumarins in *Apiaceae* is far higher than in *Rutaceae*[154]. Thus, a further expansion of CYP6B and CYP6AB and their divergence in *Pm* may greatly contribute feeding adaptation to such high toxic furanocoumarin-containing plant such as *Apiaceae*, as the theory proposed by Berenbaum et al that high inducible activity toward a specific chemistry can serve as an adaptation of herbivores to a host containing this type of chemistry[155].

In summary, strong expansions of CYP6B in *Pm* and *Px* demonstrate their adaptive strategies to furanocoumarin-containing host plants; and different diversification and duplication events of CYP6B after the split of *Pm* and *Px* provide the genetic basis of their host plant diversification, that is, *Pm* is specified to use high toxic *Apiaceae*, while *Px* can use *Rutaceae* and other families. This study, for the first time, shows a genome-

wide outline of CYP6B in *Papilio*, one of the main CYP6B-distributing taxa so far and thus provides a new model system to explore host diversification between closely related species.

Expression of CYP6 family.
We analyzed the expression patterns of CYP6 subfamily in *Pm* and *Px*. Both *Pm* and *Px* show higher expression levels in larval stage than in other stages for most of the CYP6 members, which suggests that CYP 6 family may play an important role in larval feeding.

Supplementary Note 9. Glutathione S-transferase (GST) and carboxylesterases (COE)

Except P450, Glutathione S-transferase (GST) and carboxylesterases (COE) are involved in detoxification of various xenobiotics[156]. Here, we searched GST genes in the genomes of our two butterflies (*Pm* and *Px*), *Hm*, *Dp*, *Bm* and *Dm*. Considering that COE genes include the genes encoding juvenile hormone esterase (JHE), we searched COE genes in 10 species.

The gene numbers of GST are similar among lepidoptera and fruitfly (**Supplementary Table 34**). As in *Bm*[157], *Papilio* GSTs can be classified as subgroups of *Theta*, *Omega*, *Theta*, *Zeta*, *Delta* and *Epsilon* (**Supplementary Figure 23**). A strong expansion of COE, like in *Bm*[158], is found in all butterflies (**Supplementary Table 34** and **Supplementary Figure 24**), which suggests COE may play a key role in Lepidoptera evolution.

Supplementary Note 10. The genes related to body color

Butterfly body color is composed of structural color and pigment color, or combination of both. Compared with other butterflies, swallowtails have a complex coloration pattern, which, besides pigments such as melanin, pteridine and ommochrome, also includes one lineage-specific pigment, papiliochrome **(Supplementary Figure 25)**. Melanin synthesis pathway is a fundamental pathway inboth invertebrate and vertebrate; ommochrome synthesis pathway is also a common pathway in eye development in many animals, which is also related to body color in many invertebrates. Papiliochrome, a special trait in swallowtail butterflies, is synthesized from metabolites from both melanin and ommochrome pathways. It is noted that the early steps of melanin synthesis are in fact shared with the biosynthesis of papiliochrome and cuticle sclerotization. Although

pteridine, the product of pteridine pathway, is famous in pterid butterfly, the metabolites of its biosynthesis take part in many biological processes, including the above mentioned pigmentation and cuticle sclerotization. In order to explore the molecular and genomic mechanism of butterfly body color, we investigated the pigmentation genes and other related genes at a genome-wide level.

Pigmentation genes.

By checking the gene list with GO terms including pigmentation (GO:0007593: chitin-based cuticle sclerotization; GO:0048067: cuticle pigmentation; GO:0043473: pigmentation) in Amigo (http://amigo.geneontology.org/cgi-bin/amigo/go.cgi) with *Dm* as species filter, combining redundant genes from different GOs, and then deleting genes with only one phenotype, we got a non-redundant pigmentation gene list of 107 genes. Then we downloaded gene sequences in FlyBase and geneswithout sequences were further discarded. In addition, we added those reported genes related to the color of butterfly wing or larva cases but not to be dealt with in Amigo. Thus, we got a final list of 95 genes. We first aligned the proteins of all the above genes against all the proteins in*Pm*, *Px*, *Dp*, *Hm*, *Bm* and *Dm* genomes using BLASTP[72]. The hits with alignment identity and coverage of no less than 30% were defined as candidate copies. Then we aligned the proteins of all the candidate copies in six insects using Prank[159, 160] and constructed the gene trees. Combining gene trees with species tree, we redefined copies for each pigmentation gene. The copy numbers of these pigmentation genes in six insects are shown in **Supplementary Table 36**.

Genes in the biosynthesis pathways of melanin, ommochrome, papiliochrome, pterine and cuticle sclerotization.

We further scrutinized the genes in biosynthesis pathways of melanin, ommochrome, papiliochrome, pterine and cuticle sclerotization, most of which are 1-3 copies with the exception of yellow gene family (**Supplementary Table 36**). The expression patterns of all these genes in differential stages are shown as **Supplementary Figure 26**. The most intriguing gene family is yellow, which is only identified in insects and a number of bacteria so far and has been associated with behavior, pigmentation, and sex-specific reproductive maturation and may also play a role in caste specification and neuronal signaling in honeybees[161]. We further downloaded all know yellow proteins in insects from uniprot and constructed a tree (**Supplementary Figure 27**).

Pleiotropic gene *ebony* (*e*).

Ebony (*e*) is a pleiotropic gene related to body color and behavior[162]. Its genetic variation and reciprocal function with other genes in pigmentation pathways play key roles in adaptive mechanism within and among population and species of many insects[163, 164, 165; 166, 167, 168, 169]. Also very interesting, *e* is also responsible for the synthesis of papiliochrome[170], a specific pigment in swallowtails.

We compared the amino acid sequencesof *e* between *Pm* and *Px* (**Supplementary Figure 28a**). *Ebony* of *Papilo* (*Pm* and *Px*) form a clade, which is sister to *e* of nymphid (**Supplementary Figure 28b**). The expression of *e* is up-regulated in the 2nd intar larva and adult of *Pm*, while is up-regulated in the 5th instar larva of *Px* (**Supplementary Figure 28c**). Both *e* genes contain 16 exons and 15 introns in *Pm* and *Px*

(**Supplementary Figure 29a**)

In order to check the possible contribution of *e* to different body colors in the 5th instar larvae, we checked the expression patterns of three regions (**Supplementary Figure 30a**) of *Px* with the primer pairs (Px_01073_F6: CTCTGTTAGCGACATGGAA; Px_01073_R5: AGAATATGTTAGACGCACACC) (**Supplementary Figure 29a**), and of two regions (**Supplementary Figure 30b**) in *Pm* with the primer pairs (Pm_05964_F6: CCATATTCAGCGATCTGCAAC; Pm_05964_R1: ATGTCACGTCTCCCATAACCT) (**Supplementary Figure 29a**) by reverse transcription PCR (RT-PCR). RT-PCR of conserved gene RpL3 is as an internal control (for *Px*: Px_15129_RpL3_mF3: ACGTCGCTTCTATAAGAACTGG; Px_15129_RpL3_mR3: AAGCTACTTTACGCAGACCCT; for *Pm*: Px_15129_RpL3_mF1: CTGACCGTCCTGGATCAAAG; Px_15129_RpL3_mR2: TGGGCAATAACTCTGATCACA). Our data show *e* is expressed in all three regions of *Px* (**Supplementary Figure 30c**) and two regions of *Pm* (**Supplementary Figure 30d**), which suggest the possible contribution of *e* to green color.

Positively selected gene *sepia* in *Px*.
The product of *sepia* gene, a member of GST gene family (**Supplementary Figure 23**) encoding PDA synthase, plays a key role in the production of red-orange pigment drosopterin of pteridine pathway (**Supplementary Figure 25**)[12]. Its positive selection in *Px* (**Supplementary Table 37**) suggests that red-orange pigment drosopterin may contribute to red-orange color in *Px*. Interestingly, besides the red-orange color at the hind wing eyespot of adults of both *Px* and *Pm* (**Supplementary Figure 1**), the red-orange color is also found at the faked eyes located in the metathorax of the fifth instar *Px*larva, suggesting that the red-orange pigment drosopterin may contribute to the red-orange color in *Px*. Thus, we analyzed this gene.

Sepia gene contains five exons and four introns in both *Px* and *Pm* **(Supplementary Figure 29b**). Its evolution analysis is as shown in **Supplementary Table 39.**

We also checked the expression pattern of *sepia* gene from RNA extracted from three regions (**Supplementary Figure 30a**) of *Px* using the primer pair (Px_10090_se_mF3: ACCTGAATGGCTTACTCGT; Px_10090_se_mR3: CACCGAAGAATTGAGT ACCAC) (**Supplementary Figure 29b**) and two regions (**Supplementary Figure 30b**) of *Pm* using primer pairs (Px_10090_se_mF2: GCACAACGAACTATTCTAGC; Px_10090_se_mR1: TCATCTAAATATTCCACCGT) (**Supplementary Figure 29b**) by RT-PCR. RT-PCR of conserved gene RpL3 is as an internal control (for *Px*: Px_15129_RpL3_mF3: ACGTCGCTTC TATAAGAACTGG; Px_15129_RpL3_mR3: AAGCTACTTTACGCAGACCCT; for *Pm*: Px_15129_RpL3_mF1: CTGACCGTCCTGGATCAAAG; Px_15129_RpL3_mR2: TGGGCAATAACTCTGATCACA). Our data show *sepia* is expressed in all three regions of *Px* (**Supplementary Figure 30b)** and two regions of *Pm* (**Supplementary Figure 30d**) as gene *e*.

Ommochrome-binding protein (Ombp) genes.

We found two Lepidoptera-specific families based on the Swissprot annotation of "OMBP_MANSE Ommochrome-binding protein OS=*Manduca sexta* PE=1 SV=1". Ommochrome binding proteins (Ombps) transport ommochromes, which are derivatives of the amino acid tryptophan via kynurenine and 3-hydroxykynurenine and are responsible for a wide variety of colors (yellows, reds, browns, and black) owing to different combinations of pigment metabolites[171], and also represent important routes for detoxification and excretion of tryptophan in arthropod[172, 173]. Ommb genes were identified and cloned from many Lepidoptera insects such as tobacco hormworm *Manduca sexta*, *Hyalophora cecropia* and *Bm*[172, 173, 174], but not found in fruit fly[175]. Thus, we searched and downloaded all the sequences of Ombps from GenBank, and then aligned all the protein sequences in ten insects against downloaded sequences using BLASTP[72] with an e-value cutoff of 1e-5.

Our searching in ten species confirms that Ombp genes only exist in butterflies and moths (**Supplementary Table 36**). The phylogenetic tree of all the Ombps genes (**Supplementary Figure 31a**) showed that duplication events happened to the ancestor of butterflies and moths. Most of the Ombp genes show high expression levels in adult stages but different expression patterns in larval stages (**Supplementary Figure 31b**).

Bilin-binding protein (BBP) genes.
Bilin-binding protein (BBP) is a member of the lipocalin superfamily and a pigment binding protein in Lepidoptera. In order to explore its role in butterfly color, we identified all the invertebrate coloration proteins (IPR003057), which include BBP in the cabbage white butterfly (*Pieris brassicae*)[176], the closely related protein insecticyanin in tobacco hawkmoth *Manduca sexta*[177] and the lobster protein crustacyanin[178]. We first searched all the items with the annotation of IRP003057 in 10 insects (*Px*, *Pm*, *Hm*, *Dp*, *PLX*, *Bm*, *Dm*, *Ag*, *Am*, *Tc*), which resulted in *Pm* (12), *Px* (14), *Hm* (9), *Dp* (10), *Bm* (6), *PLX* (5), *Ag* (5), *Dm* (1), *Am* (3) and *TC* (1) (**Supplementary Table 36**). In total, butterflies have more copies than other insects. Then we searched all the proteins in Uniprot with the query entry of "Bilin-binding protein". Combining all BBP and related proteins, we constructed a tree (**Supplementary Figure 32a**). The tree shows a strong expansion of BBP in Lepidoptera, which also includes all the reported BBP protein in *Pieris brassicae*, *Papilio*, etc. It is noted that some of the mosquito's BBP clustered with Lepidoptera BBP. Among others, five duplications are observed in *Papilio* clade and all of them are the highest expressed in larval stage, which may be the genomic basis for *Papilio* larval color.

We further analyzed the expression pattern of BBP genes in *Pm* and *Px*. Our data showed that different BBP genes were expressed in different stages, and the highest expressed BBP, especially in *Papili*o, is in the 5th instar larvae (**Supplementary Figures 32b-c**).

Yellow related gene (YRG).
We identified one very interesting *Papilio*-specific gene family (15968) including only one pair of orthologous genes (*Px_16957_unknow*, *Pm_08750_unknow*)in *Pm* and *Px*, and the Genbank BLASTP[72] and TBLASTN[72] results show that the pair orthologs hit the yellow related genes in three *Papilio* species (*Pm*, *Px* and *P. Polytes* (*Pp*)), which is reported to be related to yellow or green body (in the case of combination with BBP) color in *Papilio* larvae and whose expression is trans-regulated possibly by 20E-inducible

transcription factors[179, 180]. In order to explore the possible contribution of these yellow-related proteins to yellow color marking, we analyzed their gene structures and co-expression patterns in different developments.

YRG gene in*Pm* and *Px* include three exons and two introns, with those in*Pm* longer than in *Px* (**Supplementary Figure 33a)**. Together with the only three hits (*Px*, *Pm* and *Pp*) based on GenBank BLASTP[72] results, the five *YRG* proteins were all aligned using Bioedit. The alignment of all these five proteins shows that except for lack of the first 12 amino acids, reported *YRG* proteins in*Pm* and *Px* are almost identical to the sequences in this study (**Supplementary Figure 33b)**, which suggests the reliable annotation of *YRG* and their possibly limited distribution in swallowtails.

These two *YRG* proteins are highly expressed in the 5th instar larva with a much higher expression in *Pm* than in *Px* in all developmental stages (**Supplementary Figure 33c**), which strongly corresponds to their phenotypes of yellow color in different developmental stages (**Supplementary Figure 1**). We analyzed those genes and their co-expression patterns with BGI wego (http://wego.genomics.org.cn/cgi-bin/wego/index.pl). There are 98 and 101 genes strongly co-expressed with *YRG* protein (R>0.9) in *Pm* and *Px,* and all these coexpressed genes have at least one kind of annotation. WEGO comparisons of 77 and 75 genes with GO annotation (**Supplementary Figure 34**) show differences of the co-expression genes between the two species. In cellular component ontology, *Px* has more genes annotated to extracellular region (GO:0044421) (1:0) while *Pm* has more annotated to intracellular one (GO:0005622) (2:9), in which *Pm_13266_br* (orthologous to *BGIBMGA009908-PA*, *Hm_12561_br*, *Px_13432_br*) annotated as broad-complex core-protein, a transcription factor inducible by 20-E. In molecular function, although there are almost equal numbers of genes annotated to binding (GO:0005488) (27: 31), *Px* has more protein binding terms (GO:0005515) (11:4) whereas *Pm* has more nucleic acid binding ones (GO:0003676) (0:4) including one translation initiation factor (*Pm_10129_eIF4E-5*) and 3 transcription factors (*Pm_08801_ERR*; *Pm_20019_lat*; *Pm_20353_fd59A*); in addition, *Px* has more genes associated with structural constituent of cuticle (GO: 0042302) (13: 3, p0.007). In biological process, although *Px* and *Pm* have similar numbers of genes (4:3) (*Pm_08801_ERR*, *Pm_20353_fd59A*, *Pm_01074_pdfr; Px_05108_Pde6*, *Px_07389_Rgk1, Px_13744_NepYr* (Px-0019: PCB1), *Px_06657_Camta*) related to pigmentation process (GO:0043473), these genes show different functional annotations. The two G-protein coupled receptors*Pm_01074_pdfr* and *Px_13744_NepYr* suggest that their body color may be regulated by G-protein coupled receptor protein signaling pathway. *Pm_08801_ERR*, *Pm_20353_fd59A* and *Px_06657_Camta* belong to regulation of transcription (GO:0045449) in pigmentation process, which suggests that these two species may have different transcription regulation mechanisms in pigmentation.

Supplementary Note 11. Planar cell polarity (PCP) genes

Planar cell polarity (PCP), which coordinates the polarization of cells in the tissue plane, has been shown to play a fundamental role in morphogenesis of vertebrates and invertebrates[181]. There are two independently acting PCP pathways, i.e, the core pathway (*frizzled/stan*: *fz/stan*) and *Fat/Dachsous* (*ft/ds*) pathway[182].

Identification of PCP genes in butterflies.
According tothe published *Dm* PCP genes[183, 184, 185], we generated a PCP gene list, downloaded all the sequences of *Dm* PCP genes from GenBank, and then aligned all the protein sequences in ten insects against the downloaded sequences using BLASTP[72] with the e-value cutoff of 1e-5. The hits with alignment identity and coverage of no less than 30% were defined as candidate copies. Then we aligned the proteins of all the candidate copies in six insects using Prank[159] and constructed the gene trees using Maximum likelihood methods[106]. Combining gene trees with species tree, we redefined the copies for each PCP gene. The copy numbers in 10 species and the lists of *Pm* and *Px* are shown in **Supplementary Tables 38-39**.

Positively selected PCP genes in *Pm*.
By comparing the lists of PCP genes (**Supplementary Table 38**) and positively selected genes (**SupplementaryTables 26-27**), we found two PCP genes in *Ft/Ds* pathway were positively selected in *Pm* (**SupplementaryTables 39-40**).

Expression of PCP genes in *Pm* and *Px*.
Most of the PCP genes are expressed in different stages in both *Px* and *Pm* (**Supplementary Figure 35**). Among them, the expression of *fz* gene in the core *fz/stan* pathway is up-regulated in all stages in *Px*compared with in *Pm*. On the other hand, two positively selected PCP genes in *Pm* show higher expression levels than in *Px*.

Comparison of *frizzled* in *Pm* and *Px*.
We compared the 5' UTR of 500 bp (**Supplementary Figure 36a**), CDS (**Supplementary Figure 36b**) and gene structure (**Supplementary Figure 36c**) of *frizzled* genes between *Pm* and *Px*.

Supplementary Note 12. Genomic landscape of divergence

Divergence counting.

The divergence counting was performed based on the assembled genomes of *P. xuthus*, *P. machaon* and illumina genome resequencing data of one individual of a third *Papilio* species (*P. polytes*) which we generated and deposited in public database (SRA accession number: SRR1107999)[186], by randomly choosing one allele from heterozygous genotypes. The third species *P. polytes* was used to narrow down the candidate list for functional test, which also helped identify the most divergent regions across papilio genomes. The *P. xuthus* genome was used as a reference and the *P. machaon* genome was simulated to 40 × illumina paired-end reads using ART toolkit[187] and processed with the downloaded genome resequencing data from one *P. polytes* individual (SRA accession number: SRR1107999). The *P. machaon* and *P. polytes* data were aligned to the *P. xuthus* reference using Bowtie2 v2.0.0-beta7[77] with parameter –very-sensitive-local and then were re-ordered and sorted by Picard v1.84 (http://picard.sourceforge.net). RealignerTargetCreator[78] and IndelRealigner in GATK v2.1 were used to realign indels and UnifiedGenotyper[79] was used to call genotypes using the following parameters: heterozygosity 0.01, stand_call-conf 50, stand_emit_conf 10, dcov 250. Then the genome was divided into non-overlapping windows of 50 Kb in size and the density of different SNPs per bp of each window was estimated for three comparisons: *P. xuthus*/*P. machaon*, *P. xuthus*/*P. polytes* and *P. machaon*/*P.polytes*.

Identification of highly divergent regions and the relevant genes.

The data distributions of all three comparisons (*P. xuthus*/*P. machaon*, *P. xuthus*/*P. polytes* and *P. machaon*/*P.polytes*) were estimated, and we did merge three divergence maps and aimed to show all the informative results in one turn. 70 highly divergent outlier regions were selected using 95% smoothed empirical likelihood quantiles among all three comparisons as a cutoff (**Supplementary Table 41** and **Supplementary Figure 37**). It makes sense that some highly divergent regions can be promoted by adaptation via positive selection and differential expression. To functionally test candidate genes fulfilling all the criteria will help better understand the relevance and significance of these biological mechanisms. The genes under recent positive selection were also possibly involved in historical adaptations via recurrent/episodic positive selection. Taking the butterfly linkage disequilibrium into consideration, 915 genes were extracted from both original and extended outlier regions (±50 kb), among which there were four genes differentially expressed between *P. xuthus* and *P. machaon* in ten development stages and eight genes under positive selection in both *P. xuthus* and *P. machaon* (**Supplementary Table 43**). Notably the three differentially expressed genes were all up-regulated in *P. xuthus* and one of them was *frizzled* (*fz*). Functional enrichment of the 915 genes is shown in **Supplementary Table 42**.

Supplementary Note 13. Genome editing in butterfly using CRISPR/Cas9 system

Functional elucidation of causal genetic variants and elements requires precise genome editing technologies. Besides zinc-finger nucleases (ZFNs) and transcription activator-like effector nucleases (TALENs), clustered regulatory interspaced short palindromic repeat (CRISPR) associated (Cas) based RNA-guided DNA endonucleases recently emerged as a potentially facile and efficient alternative to ZFNs and TALENs for inducing targeted genetic alterations[188]. The *Streptococcus pyogenes* Cas9 (SpCas9) nuclease can be efficiently targeted to genomic sites by means of single guide RNAs (sgRNAs) to enable genome editing from bacteria, model plants, fruitfly, zebrafish, mouse, and human cells[189, 190, 191]. Although butterflies are emerging as exceptional model systems with which to link the developmental and genetic processes that generate morphological variation with the ecological and evolutionary processes that mould variation in natural populations[192], relatively immature genome editing technologies in butterflies, to a great degree, restrict their application as models. In order to develop butterflies as new model organisms, we developed CRISPR/Cas9 technology platform in butterfly (**Supplementary Figure 38**), through the cases of homoebox (hox) gene *Abdominal-B* (*Abd-B*), a pleiotropic gene *ebony* (*e*) and plunar cell polarity (PCP) gene *frizzled* (*fz*).

Genome editing of homeobox gene *Abd-B* in *Px*.
Because the mutation and decreased expression of *Abd-B* were demonstrated to cause the development of extra prolegs on all segments posterior to abdomen segments 6 (A6) in silkworm[193], a visible morphologica phenotype, we selected *Abd-B* as our model to test Cas9 system in such non-model organisms as butterflies. We designed four sgRNAs (T42, T95, T100, T248) (**Supplementary Table 44)** to target double-strand break in the second exon of *Abd-B*. Although our preliminary injection with the different combination (I, II, III) of the four sgRNAs and Cas9 mRNA showed no mutated individual with extra prolegs, the sequencing of target sites for one (I: T42, T95, T248) of combinations demonstrated that 3 out of 15 individuals investigated were genome-edited in two target sites (T42, T95) of *Abd-B* with deletion of 4-136 bp between T42 and T95 or insertion of 7-25 bp around T42 (**Supplementary Table 45** and **Supplementary Figure 40**), suggesting that T42 and T95 may be two ideal targets.

Then we tried seven different combinations (IV, V, VI, VII, VIII, IX, XI) of different concentrations of T42-T95 and T42 (**Supplementary Table 45**). Excitingly, three (V, VII, VIII) of these treatments gave rise to 23 morphologically mutated individuals (including hatched larvae and unhatched larvae dissected from eggs) with extra prolegs (16 mutants) or without extra prolegs but with abnormal abdomen (7 mutants), which could be grouped into four types (type2, 3, 4, 5). These mutant types (type2-5) are described as: type2, abnormal terga of A3 and thereafter resulting in abdomen curling up, ventral side of from A7 to A9 each with a pair of legs; type3, abnormal terga of A3 and thereafter resulting abdomen curling up, right or left ventral side of from A7 to A10 each with a proleg; type4, abnormal terga of A3 and thereafter resulting abdomen curling up, right or left ventral side of A7 with a proleg; type5, abnormal terga of A3 and thereafter resulting abdomen curling up without redundant legs on the ventral sides of from A7 to A10) (**Supplementary Table 46** and **Supplementary Figure 43)**. All 11 hatched larvae were

carefully raised but could not finish their larval developments because their abnormal abdomens seriously affected their behaviors such as feeding and defecation. It is noted that all type2 mutated individual died very soon after hatching because of feeding difficulties; type3-5 mutated individuals could develop to 2nd or 3th instar. We amplified target sites from all morpholically mutated individualsand unmutated indviduals.T7EI check confirms the genome-editing of all morphologically mutated individuals (**Supplementary Figure 41a**) and also identifiedsome morphologically unmutated but possibly genome-edited individuals**.** Sequencing further confirms genome-editing of morphologicallymutated individuals and T7EI-positive morphogically unmutated indviduals **(Supplementary Figure 42).**

Even so, the rate of mutated individual is very low (7.4%: 23/309) for the three combinations (V, VII, VIII). We speculate that low concentration of sgRNAs and Cas9mRNA may be the reasons for low mutation rate. So, we carried out another injection (XIII) with higher concentration of sgRNA (T42, 566.1 ng/μl; T95, 415.8ng/μl) and Cas9mRNA (1200 ng/μl) (**Supplementary Table 45)** and with shortened period from egg laying to injection within 2 hr. Surprisingly, this injection results in not only high rate of mutated individuals (92.5%: 123/133) but also more abnormal phenotypes with four pair extra legs from A7 to A10 in most of the mutated individuals (type1: 90% (107/123)) (**Supplementary Tables 45-46** and **Supplementary Figure 43a)**. Most of the mutated individuals could not hatch from egg; among hatched individuals, type 1, like type 2, died very soon after hatching owing to feeding difficulties, and compared with the individuals of wide type and no-mutated phenotype hatched at the same day, type 3 and 5 show delayed development (**Supplementary Figure 44**) and died in their 2nd instar. We selected 10 unhatched larvae and five hatched larva for DNA extraction, target sites amplification. All these individuals show positive results by T7EI analyses (**Supplementary Figure 41b**) and are confirmed by sequencing (**Supplementary Figure 42**).

In order to check effect of disruption of target gene *Abd-B* on its expression at the RNA level, we performed quantitative reverse transcription-PCR (qRT-PCR) for type1 mutants of injection XIII and wild type. Total RNA was extracted using the guanidinium thiocyanate-phenol-chloroform extraction method (Trizol, Invitrogen) according to manufacturer's protocol, and was reverse transcribed into cDNA using the RevertAid HMinus First Strand cDNA Synthesis kit. Our primary experiment suggested that total RNA of one developed unhatched larvae dissected from egg is not enough for qRT-PCR. Thus, five individual of developed but unhatched larvae dissected from eggs were mixed as one biological sample for extraction of total RNA, for both mutant and wild type respectively.Three biological replicates were carried out for both mutant and wild type. Quantitative RT-PCR was performed using QUANTSTUDIOTM 12K FLEX (Applied Biosystems) with the SYBR Premix Ex Taq (Takara), with four experiment duplicates for each sample. RT-PCR primers for *Abd-B* gene are Px_03961-qRT_F1 (TCCCACGACGCATACGGTCT) and Px_03961-qRT_R1 9 (CACCTGCCCGGTCCAGTCC) with forward primer in the first exon and reverse primer in the second exon. The gene for cytoplasmis actin gene A3 that is expressed constitutively in the cell was used as an internal standard to estimate the relative

expression of mRNA. *Px* actin A3 gene was identified as *Px_11652_Act5C* by BLAST the sequence of *B. mori* actin A3 gene (GenBank accession number X04507.1) against *Px* genes. The primers for Acting A3 were shown as: Px_11652_Act5C_F1 (GCACCACACCTTCTACAATGAGC) and Px_11652_Act5C_R1 (ATCCCTCATAGATGGGCACCGT). The relative expression of *Abd-B* in mutants and in wild type was analyzed by $2^{-\Delta\Delta CT}$ method. The differences in expression of *Abd-B* gene between mutants and wild type were compared by SPSS16.0 statistics sortware (SPSS Inc., Chicago, USA) using Independent_Sample *t*Test. A p value <0.05 was considered significant. Our qRT-PCR data demonstrated that comparing with wild type, mutants hasa very low expression level of *Abd-B* gene with significance level (*t*-test, P=0.026) (**Supplementary Figure 45a).**

In order to confirm effect of disruption of target gene *Abd-B* on its expression at the protein level, we also performed western blotting analysis for type1 mutants of injection XIII and wild types. Protein samples were prepared in RIPA lysis buffer (Sigma). All protein samples from 10 unhatched mutated larvae and 10 unhatched wild type larvae dissected from developed eggs were run on 10% SDS-polyacylamide gels and then transferred to the polyvinylidene difluoride (PVDF) membranes (Millipore, Bedford, MA, USA). Blotting was performed using antibodies detecting *Abd-B* gene product (1A2E9) (DSHB) (diluted in bovine albumin (0218054991) (MPbio) with the ration of 1: 50) developedfrom fruitfly[194], andβ-actin (66009-1-Ig) (Proteintech) (dilution rate of 1: 500) used as the control. Horseradishperoxidase-coupled anti-mouse IgG (SA00001-1) (Proteintech) was used as the secondary antibody (dilution rate of 1: 1000). Supersignal™ West Pico Chemiluminescent Substrate (Thermo scientific) was used to detect chemiluminescence according to the manufacturer's instructions.Thewestern blot result confirmed extremely low or even undetectable level of Abd-B protein in mutants compared to the wild types (**Supplementary Figure 45b).**

Genome editing of pleiotropic gene *ebony* in in *Px*.
In order to confirm the reliability of Cas9 technology in butterflies, we selected pleiotropic gene *ebony*related to body color and behavior[162] as another case. We first carried out injection (*Px_01073_e*-I) with a single target site (*Px_01073_e*-T2) (**Supplementary Table 44)**, but neither morphologically mutated individual nor disruption of target site from injected individuals was identified (**Supplementary Table 47**).

Later, we carried another two experiments (*Px_01073_e*-II, III) with co-injection of two sgRNAs (II: T2, T303; III: T454, T6) and with improved experimental details. As expected, both experiments show morphologically mutated larvae of 5th instar, which lack orange regions of false eye with black color in half or whole dorsal side (**Supplementary Figure 46**). Experiment III (88%: 22/25) produced more mutated individuals than II (25%: 5/16) (**Supplementary Table 48**). Among mutated larvae, the one developed into a female adult, which, as expected, also shows body color change, from yellow to brown (**Supplementary Figure 48**). The mutated female was successfully hand-paired with wild-type male, but laid no eggs and died two days after mating. From unmutated larvae,

one developed into a male, which shows no color change. In addition, we also observed two mutated individuals showing abnormal ostemerium morphology and behavior (**Supplementary Figure 47**). We amplified target sites from most of the morphologically mutated individuals (**Supplementary Figure 49a)**.T7EI mutation analysis (**Supplementary Figure 49b)** and TA clone sequencing **(Supplementary Figure 50)** confirm the genome editing of all investigated morphologically mutated individuals.

Aiming to verify theexpression change of *ebony* in mutants, we also performed qRT-PCR as described in that of *Abd-B* gene except that total RNA was extracted from a single individual for both mutants and wild types. RT-PCR primers for *ebony* gene are Px_10703-qF1 (CTCACACATACTAAAGGATGCG) and Px_10703-qR7 (CACTCCCGGAAAACATAGAGG) with forward primer in the third exon and reverse primer in the fourth exon. As in qRT-PCR of *Abd-B* gene, cytoplasmis actin gene A3 was used as an internal standard to estimate the relative expression of *ebony* mRNA. Our qRT-PCR data demonstrated that comparing with wild type, mutants hasa very low expression level of *ebony* gene with significance level (*t*-test, *P*=0.001) (**Supplementary Figure 51).**

Genome editing of planar cell polarity (PCP) gene *frizzled* (*fz*) in *Px*.
In order to explore the function of the PCP gene *fz* with higher expression in *Px* than in *Pm*, we designed five target sites (**Supplementary Table 44**) and carried out two kinds of injections with two or three target sites (*Px_15230_fz*-I: T432, T474, T508; *Px_15230*_II: T268, T283) (**Supplementary Table 49**). Initial check of 10 mixed unhatched larvae of both I and II shows the disruption of some target sites with II better than with I (**Supplementary Figure 52**). We observed no morphologically mutated individual in I, but found 4 larva in II showed obviously morphological mutation (**Supplementary Table 49** and **Supplementary Figure 53**).We amplified target sites from four morphologically mutated individuals (**Supplementary Figure 54a)**.T7EI mutation analysis (**Supplementary Figure 54b)** and TA clone sequencing **(Supplementary Table 50** and **Supplementary Figure 55)** confirm the disruption of*fz* in all investigated morphologically mutated individuals.

Off-target analyses for *Abd-B, ebony* and *frizzled* and validation by resequencing mutant in *Px*.
There are 8 target sites that resulted in observable phenotye changes in our gene editing experiments for the three genes (**Supplementary Table 51**). All 8 target sites were selected according to the identification criteria of 12 nt seed region unique in the genome and PAM pattern NGG because previousevidence suggest 12 nt seed region is necessary for Cas9 nuclease[189]. However, some evidence especially from human cell also demonstrate tolerance of 7-12 nt seed region,alternative PAM pattern (NAG) and especially up to 5 mismatches for this nuclease[195, 196, 197, 198, 199]. Thus, in order to exclude whether the phenotypic changes were also contributed in some cases by the off-target cleavage events during the genome editing process,we further identified the potential off-targets of these 8 target sites by thorough methods including CasOT[200], Cas-OFFinder[201], and COSMID[202], which include mismatch of up to 5 bp and/or 1 insertion/deletion of 1 bp. In searching for potential off-targets,CasOT[200] was used with the parameters of

mismatch ≤ 5 bp (≤ 3 bp in 12nt of seed region) and PAM of NGG or NAG, and Cas-OFFinder was used with the parameters of mismatch ≤ 5 bp in 20 nt of seed- and non-seed region and PAM of NGG or NAG. Nevertheless, these two searching tools (CasOT and Cas-OFFinger) can only identify off-targets with mismatch, but not those with insertion or deletion, which could be also tolerated by Cas9 nuclease with varied activity[199]. So, COSMID[202], which can identify off-targets with insertion, deletion, and/or mismatch, was used to search for potential off-targets with three kinds of parameters ((1) mismatch: ≤3 bp; insert: 0; deletion: 0; PAM: NGG or NAG. (2) mismatch: ≤2 bp; insert: 1 bp; deletion: 0; PAM: NGG or NAG. (3) mismatch: ≤2 bp; insert: 0; deletion: 1 bp; PAM: NGG or NAG.). The results are summarized in **Supplementary Table 51.** For the two target site (Px_10703_T454 and T6) of *ebony* gene, the three methods identified 197 possible off-target sites, but none of them is completely identical to the target sites **(Supplementary Table 51).**

The use of high-throughput sequencing technology are thought to enable the interrogation of large numbers of candidate off-target sites and provide a more sensitive method for detecting bona fide off-target mutation[195, 196, 197, 203]. Aiming to examine large numbers of potential off-targets, we carried out whole genome next generation sequencing for one *ebony* mutant (Px_10703_e-III-4) produced in the injection treatment Px_10703_e-III (co-injection of sgRNA of targets T454 and T6). The same DNA extracted for amplification of target sites in T7EI assay and TA clone sequencing was used for resquencing with Illumina Hiseq 2500 platform. By pair-ended 125 bp (PE125) sequencing, we got 6.7 Gb high quanlity reads, and the coverage depth is 27 folds of *Px* reference genome. We firstly successfully aligned 94.19% reads to the *Px* reference genome by BWA-MEM (version 0.7.12-r1039)[73] with the parameters (-k17 -B 3 -O 5,5 -t 5 -r 3). Then, in order to assess the mutant for small indels and single-nucleotide variants (SNVs), we carried out calling variations by samtools-1.2 mpipeup (http://github.com/samtools/samtools) with default and BCFtools (BCFtools call) (http://github.com/samtools/bcftools). Finally we filter false positive SNP and INDEL by BCFtools (BCFtools view) with parameter (-i '(TYPE="indel" | TYPE="snp") & MIN (DP)>5 & MIN (MQ>20) & MAX (DP) <54 & QUAL>50').

Reads alignment clearly showed the disruption of the two target sites of the *ebony* gene, identical to that observed by Sanger sequencing (**Supplementary Figure 56),** but didn’t disrupted any other functional gene in the mutant **(SupplementaryTable 52)**. Among the 197 possible off-target sites of *ebony* T454 (143 sites) and T6 (54 sites), we observed that 33 sites have variation different from the reference genome. Among these 33 sites, 29 are due to SNVs, and 4 are due to indels. Three SNVs are found at the possible off-target sites of three genes, respectively, but they are all synonymous. Other 30 sites are all in non-coding regions. Therefore, the observed phenotypes are most possibly caused by the designed target genes’ disruption rather than off-targeting. The 33 variation sites that didn’t disrupt genes could result from polymorphisms given the high heterozygosity of *P.*

xuthus, although the possibility of off-targeting could not completely excluded at this point. High specificity of Cas9 editing and low incidence of off-target mutations have recently been reported by whole-genome sequencing of human stem cell[203, 204], but in butterflies future detailed analysis on offsprings from the same parents will allow to test whether some of the varaitions are from off-targeting or not. Nevertheless, our results show that Cas9 is generally an efficient and realiable tool in butterfly gene editing. The resequencing reads have been deposited into the NCBI Sequence Read Archive (SRA) under the accession number SRA272356.

Supplementary References

1. Belles X, Martin D, Piulachs MD. The mevalonate pathway and the synthesis of juvenile hormone in insects. *Annual review of entomology* **50**, 181-199 (2005).

2. Kinjoh T, Kaneko Y, Itoyama K, Mita K, Hiruma K, Shinoda T. Control of juvenile hormone biosynthesis in Bombyx mori: Cloning of the enzymes in the mevalonate pathway and assessment of their developmental expression in the corpora allata. *Insect biochemistry and molecular biology* **37**, 808-818 (2007).

3. Vandermoten S*, et al.* Structural features conferring dual Geranyl/Farnesyl diphosphate synthase activity to an aphid prenyltransferase. *Insect biochemistry and molecular biology* **39**, 707-716 (2009).

4. Ferre J, Silva FJ, Real MD, Mensua JL. Pigment patterns in mutants affecting the biosynthesis of pteridines and xanthommatin in Drosophila melanogaster. *Biochemical genetics* **24**, 545-569 (1986).

5. Ficner R, Sauer UH, Stier G, Suck D. Three-dimensional structure of the bifunctional protein PCD/DCoH, a cytoplasmic enzyme interacting with transcription factor HNF1. *The EMBO journal* **14**, 2034-2042 (1995).

6. Borycz J, Borycz JA, Loubani M, Meinertzhagen IA. tan and ebony genes regulate a novel pathway for transmitter metabolism at fly photoreceptor terminals. *Journal of Neuroscience* **22**, 10549-10557 (2002).

7. Ziegler I. The pteridine pathway in zebrafish: Regulation and specification during the determination of neural crest cell-fate. *Pigm Cell Res* **16**, 172-182 (2003).

8. Wittkopp PJ, Carroll SB, Kopp A. Evolution in black and white: genetic control of pigment patterns in Drosophila. *Trends in Genetics* **19**, 495-504 (2003).

9. Kato T, Sawada H, Yamamoto T, Mase K, Nakagoshi M. Pigment pattern formation in the quail mutant of the silkworm, Bombyx mori: parallel increase of pteridine biosynthesis and

pigmentation of melanin and ommochromes. *Pigm Cell Res* **19**, 337-345 (2006).

10. Meng Y*, et al.* The Silkworm Mutant lemon (lemon lethal) Is a Potential Insect Model for Human Sepiapterin Reductase Deficiency. *Journal of Biological Chemistry* **284**, 11698-11705 (2009).

11. Ferguson LC, Jiggins CD. Shared and divergent expression domains on mimetic Heliconius wings. *Evolution & development* **11**, 498-512 (2009).

12. Kim J, Park SI, Ahn C, Kim H, Yim J. Guanine Deaminase Functions as Dihydropterin Deaminase in the Biosynthesis of Aurodrosopterin, a Minor Red Eye Pigment of Drosophila. *Journal of Biological Chemistry* **284**, 23426-23435 (2009).

13. Nijhout HF. Molecular and Physiological Basis of Colour Pattern Formation. *Adv Insect Physiol* **38**, 219-265 (2010).

14. Andersen SO. Insect cuticular sclerotization: A review. *Insect biochemistry and molecular biology* **40**, 166-178 (2010).

15. Clark AG*, et al.* Evolution of genes and genomes on the Drosophila phylogeny. *Nature* **450**, 203-218 (2007).

16. Adams MD*, et al.* The genome sequence of Drosophila melanogaster. *Science* **287**, 2185-2195 (2000).

17. Flot JF*, et al.* Genomic evidence for ameiotic evolution in the bdelloid rotifer Adineta vaga. *Nature* **500**, 453-457 (2013).

18. Grbic M*, et al.* The genome of Tetranychus urticae reveals herbivorous pest adaptations. *Nature* **479**, 487-492 (2011).

19. Srivastava M*, et al.* The Trichoplax genome and the nature of placozoans. *Nature* **454**, 955-960 (2008).

20. Lindblad-Toh K*, et al.* Genome sequence, comparative analysis and haplotype structure of the domestic dog. *Nature* **438**, 803-819 (2005).

21. Smith JJ*, et al.* Sequencing of the sea lamprey (Petromyzon marinus) genome provides insights into vertebrate evolution. *Nature genetics* **45**, 415-421, 421e411-412 (2013).

22. Venter JC*, et al.* The sequence of the human genome. *Science* **291**, 1304-1351 (2001).

23. Nishikawa H*, et al.* A genetic mechanism for female-limited Batesian mimicry in Papilio butterfly. *Nature genetics*, (2015).

24. Mikkelsen TS*, et al.* Genome of the marsupial Monodelphis domestica reveals innovation in non-coding sequences. *Nature* **447**, 167-177 (2007).

25. Kelley JL*, et al.* Compact genome of the Antarctic midge is likely an adaptation to an extreme environment. *Nature communications* **5**, 4611 (2014).

26. Qu Y*, et al.* Ground tit genome reveals avian adaptation to living at high altitudes in the Tibetan plateau. *Nature communications* **4**, 2071 (2013).

27. Jones FC, *et al.* The genomic basis of adaptive evolution in threespine sticklebacks. *Nature* **484**, 55-61 (2012).

28. Nene V, *et al.* Genome sequence of Aedes aegypti, a major arbovirus vector. *Science* **316**, 1718-1723 (2007).

29. Groenen MA, *et al.* Analyses of pig genomes provide insight into porcine demography and evolution. *Nature* **491**, 393-398 (2012).

30. Alfoldi J, *et al.* The genome of the green anole lizard and a comparative analysis with birds and mammals. *Nature* **477**, 587-591 (2011).

31. Mitreva M, *et al.* The draft genome of the parasitic nematode Trichinella spiralis. *Nature genetics* **43**, 228-235 (2011).

32. Dasmahapatra KK, *et al.* Butterfly genome reveals promiscuous exchange of mimicry adaptations among species. *Nature* **487**, 94-98 (2012).

33. Zhan S, Merlin C, Boore JL, Reppert SM. The Monarch Butterfly Genome Yields Insights into Long-Distance Migration. *Cell* **147**, 1171-1185 (2011).

34. Cong Q, Borek D, Otwinowski Z, Grishin NV. Tiger Swallowtail Genome Reveals Mechanisms for Speciation and Caterpillar Chemical Defense. *Cell reports* **10**, 910-919 (2015).

35. You MS, *et al.* A heterozygous moth genome provides insights into herbivory and detoxification. *Nature genetics* **45**, 220-225 (2013).

36. Insights into social insects from the genome of the honeybee Apis mellifera. *Nature* **443**, 931-949 (2006).

37. Richards S, *et al.* The genome of the model beetle and pest Tribolium castaneum. *Nature* **452**, 949-955 (2008).

38. Stein LD, *et al.* The genome sequence of Caenorhabditis briggsae: a platform for comparative genomics. *PLoS biology* **1**, E45 (2003).

39. Gibbs RA, *et al.* Genome sequence of the Brown Norway rat yields insights into mammalian evolution. *Nature* **428**, 493-521 (2004).

40. El-Sayed NM, *et al.* The genome sequence of Trypanosoma cruzi, etiologic agent of Chagas disease. *Science* **309**, 409-415 (2005).

41. Putnam NH, *et al.* The amphioxus genome and the evolution of the chordate karyotype. *Nature* **453**, 1064-1071 (2008).

42. Waterston RH, *et al.* Initial sequencing and comparative analysis of the mouse genome. *Nature* **420**, 520-562 (2002).

43. Dehal P, *et al.* The draft genome of Ciona intestinalis: insights into chordate and vertebrate origins. *Science* **298**, 2157-2167 (2002).

44. Terrapon N, *et al.* Molecular traces of alternative social organization in a termite genome. *Nature communications* **5**, 3636 (2014).

45. Putnam NH*, et al.* Sea anemone genome reveals ancestral eumetazoan gene repertoire and genomic organization. *Science* **317**, 86-94 (2007).

46. Zhang G*, et al.* The oyster genome reveals stress adaptation and complexity of shell formation. *Nature* **490**, 49-54 (2012).

47. Hellsten U*, et al.* The genome of the Western clawed frog Xenopus tropicalis. *Science* **328**, 633-636 (2010).

48. Ahola V*, et al.* The Glanville fritillary genome retains an ancient karyotype and reveals selective chromosomal fusions in Lepidoptera. *Nature communications* **5**, 4737 (2014).

49. Xia Q*, et al.* A draft sequence for the genome of the domesticated silkworm (Bombyx mori). *Science* **306**, 1937-1940 (2004).

50. Srivastava M*, et al.* The Amphimedon queenslandica genome and the evolution of animal complexity. *Nature* **466**, 720-726 (2010).

51. Genome sequence of the pea aphid Acyrthosiphon pisum. *PLoS biology* **8**, e1000313 (2010).

52. Wang X*, et al.* The locust genome provides insight into swarm formation and long-distance flight. *Nature communications* **5**, 2957 (2014).

53. Sodergren E*, et al.* The genome of the sea urchin Strongylocentrotus purpuratus. *Science* **314**, 941-952 (2006).

54. Colbourne JK*, et al.* The ecoresponsive genome of Daphnia pulex. *Science* **331**, 555-561 (2011).

55. Nelson DR. The cytochrome p450 homepage. *Human genomics* **4**, 59-65 (2009).

56. Arikawa KS, D.; Fujii, T. Hindsight by genitalia: photo-guided copulation in butterflies. *J comp Physiol A* **180**, 5 (1997).

57. Lederhouse RCA, M. P.; Scriber J. M. Evaluation of spermatorphore counts in studying mating systems of Lepidoptera. *Journal of the Lepidopterists' Society* **43**, 9 (1989).

58. Langley CH, Crepeau M, Cardeno C, Corbett-Detig R, Stevens K. Circumventing heterozygosity: sequencing the amplified genome of a single haploid Drosophila melanogaster embryo. *Genetics* **188**, 239-246 (2011).

59. Holt RA*, et al.* The genome sequence of the malaria mosquito Anopheles gambiae. *Science* **298**, 129-149 (2002).

60. Star B*, et al.* The genome sequence of Atlantic cod reveals a unique immune system. *Nature* **477**, 207-210 (2011).

61. Dalloul RA*, et al.* Multi-platform next-generation sequencing of the domestic turkey (Meleagris gallopavo): genome assembly and analysis. *PLoS biology* **8**, (2010).

62. Li R*, et al.* The sequence and de novo assembly of the giant panda genome. *Nature* **463**, 311-317 (2010).

63. Li Y, Hu Y, Bolund L, Wang J. State of the art de novo assembly of human genomes from massively parallel sequencing data. *Human genomics* **4**, 271-277 (2010).

64. Margulies M*, et al.* Genome sequencing in microfabricated high-density picolitre reactors. *Nature* **437**, 376-380 (2005).

65. Bennett MD, Leitch IJ, Price HJ, Johnston JS. Comparisons with Caenorhabditis (approximately 100 Mb) and Drosophila (approximately 175 Mb) using flow cytometry show genome size in Arabidopsis to be approximately 157 Mb and thus approximately 25% larger than the Arabidopsis genome initiative estimate of approximately 125 Mb. *Annals of botany* **91**, 547-557 (2003).

66. Jiggins CD, Mavarez J, Beltran M, McMillan WO, Johnston JS, Bermingham E. A genetic linkage map of the mimetic butterfly Heliconius melpomene. *Genetics* **171**, 557-570 (2005).

67. Galbraith DW, Harkins KR, Maddox JM, Ayres NM, Sharma DP, Firoozabady E. Rapid flow cytometric analysis of the cell cycle in intact plant tissues. *Science* **220**, 1049-1051 (1983).

68. Dolezel J, Bartos J, Voglmayr H, Greilhuber J. Nuclear DNA content and genome size of trout and human. *Cytometry Part A : the journal of the International Society for Analytical Cytology* **51**, 127-128; author reply 129 (2003).

69. Edgar RC. MUSCLE: multiple sequence alignment with high accuracy and high throughput. *Nucleic acids research* **32**, 1792-1797 (2004).

70. Gregory TRHPD. Genome size variation in lepidopteran insects. *Can J Zool* **81**, 7 (2003).

71. Harris RS. Improved pairwise alignment of genomic DNA. *PhD Thesis The Pennsylvania State University*, (2007).

72. Altschul SF, Gish W, Miller W, Myers EW, Lipman DJ. Basic local alignment search tool. *Journal of molecular biology* **215**, 403-410 (1990).

73. Li H, Durbin R. Fast and accurate short read alignment with Burrows-Wheeler transform. *Bioinformatics* **25**, 1754-1760 (2009).

74. Parra G, Bradnam K, Korf I. CEGMA: a pipeline to accurately annotate core genes in eukaryotic genomes. *Bioinformatics* **23**, 1061-1067 (2007).

75. Birney E, Clamp M, Durbin R. GeneWise and Genomewise. *Genome research* **14**, 988-995 (2004).

76. Etter PD, Bassham S, Hohenlohe PA, Johnson EA, Cresko WA. SNP discovery and genotyping for evolutionary genetics using RAD sequencing. *Methods Mol Biol* **772**, 157-178 (2011).

77. Langmead B, Salzberg SL. Fast gapped-read alignment with Bowtie 2. *Nat Methods* **9**, 357-359 (2012).

78. McKenna A*, et al.* The Genome Analysis Toolkit: a MapReduce framework for analyzing next-generation DNA sequencing data. *Genome Res* **20**, 1297-1303 (2010).

79. DePristo MA*, et al.* A framework for variation discovery and genotyping using next-generation DNA sequencing data. *Nat Genet* **43**, 491-498 (2011).

80. Van Ooijen J. JoinMap 4. *Software for the calculation of genetic linkage maps in experimental*

populations Kyazma BV, Wageningen, Netherlands, (2006).

81. Voorrips RE. MapChart: Software for the graphical presentation of linkage maps and QTLs. *Journal of Heredity* **93**, 77-78 (2002).

82. Maeki K. A Use of Chromosome Numbers in the Study of Taxonomy of the Lepidoptera and Notes on the Internal Reproductive Anatomy. *OURNAL OF THE FACULTY OF SCIENCE HOKKAIDO UNIVERSITY Series VI Zoology* **13**, 4 (1957).

83. Krzywinski M*, et al.* Circos: An information aesthetic for comparative genomics. *Genome research* **19**, 1639-1645 (2009).

84. Benson G. Tandem repeats finder: a program to analyze DNA sequences. *Nucleic acids research* **27**, 573-580 (1999).

85. Tarailo-Graovac M, Chen N. Using RepeatMasker to identify repetitive elements in genomic sequences. *Current protocols in bioinformatics / editoral board, Andreas D Baxevanis [et al]* **Chapter 4**, Unit 4 10 (2009).

86. Price AL, Jones NC, Pevzner PA. De novo identification of repeat families in large genomes. *Bioinformatics* **21 Suppl 1**, i351-358 (2005).

87. Wicker T*, et al.* A unified classification system for eukaryotic transposable elements. *Nature reviews Genetics* **8**, 973-982 (2007).

88. Kim EB*, et al.* Genome sequencing reveals insights into physiology and longevity of the naked mole rat. *Nature* **479**, 223-227 (2011).

89. Kapitonov VV, Jurka J. Molecular paleontology of transposable elements in the Drosophila melanogaster genome. *Proceedings of the National Academy of Sciences of the United States of America* **100**, 6569-6574 (2003).

90. Wong DC, Pearson RD, Elvin CM, Merritt DJ. Expression of the rubber-like protein, resilin, in developing and functional insect cuticle determined using a Drosophila anti-Rec 1 resilin antibody. *Developmental dynamics : an official publication of the American Association of Anatomists* **241**, 333-339 (2012).

91. Osanai-Futahashi M, Suetsugu Y, Mita K, Fujiwara H. Genome-wide screening and characterization of transposable elements and their distribution analysis in the silkworm, Bombyx mori. *Insect biochemistry and molecular biology* **38**, 1046-1057 (2008).

92. Kapitonov VV, Jurka J. Helitrons in fruit flies. *Repbase Reports* **7**, 6 (2007).

93. Kapitonov VV, Jurka J. Helitrons on a roll: eukaryotic rolling-circle transposons. *Trends in genetics : TIG* **23**, 521-529 (2007).

94. Mount DW. Using the Basic Local Alignment Search Tool (BLAST). *CSH protocols* **2007**, pdb top17 (2007).

95. Stanke M, Waack S. Gene prediction with a hidden Markov model and a new intron submodel. *Bioinformatics* **19 Suppl 2**, ii215-225 (2003).

96. Korf I. Gene finding in novel genomes. *BMC bioinformatics* **5**, 59 (2004).

97. Majoros WH, Pertea M, Salzberg SL. TigrScan and GlimmerHMM: two open source ab initio eukaryotic gene-finders. *Bioinformatics* **20**, 2878-2879 (2004).

98. Elsik CG, Mackey AJ, Reese JT, Milshina NV, Roos DS, Weinstock GM. Creating a honey bee consensus gene set. *Genome biology* **8**, R13 (2007).

99. Bairoch A, Apweiler R. The SWISS-PROT protein sequence database and its supplement TrEMBL in 2000. *Nucleic acids research* **28**, 45-48 (2000).

100. Kanehisa M, Goto S. KEGG: kyoto encyclopedia of genes and genomes. *Nucleic acids research* **28**, 27-30 (2000).

101. Mulder N, Apweiler R. InterPro and InterProScan: tools for protein sequence classification and comparison. *Methods Mol Biol* **396**, 59-70 (2007).

102. Ashburner M*, et al.* Gene ontology: tool for the unification of biology. The Gene Ontology Consortium. *Nature genetics* **25**, 25-29 (2000).

103. Li H*, et al.* TreeFam: a curated database of phylogenetic trees of animal gene families. *Nucleic acids research* **34**, D572-580 (2006).

104. Talavera G, Castresana J. Improvement of phylogenies after removing divergent and ambiguously aligned blocks from protein sequence alignments. *Systematic biology* **56**, 564-577 (2007).

105. Guindon S, Dufayard JF, Lefort V, Anisimova M, Hordijk W, Gascuel O. New algorithms and methods to estimate maximum-likelihood phylogenies: assessing the performance of PhyML 3.0. *Systematic biology* **59**, 307-321 (2010).

106. Yang Z. PAML 4: phylogenetic analysis by maximum likelihood. *Molecular biology and evolution* **24**, 1586-1591 (2007).

107. Benton MJ, Donoghue PC. Paleontological evidence to date the tree of life. *Molecular biology and evolution* **24**, 26-53 (2007).

108. Douzery EJ, Snell EA, Bapteste E, Delsuc F, Philippe H. The timing of eukaryotic evolution: does a relaxed molecular clock reconcile proteins and fossils? *Proceedings of the National Academy of Sciences of the United States of America* **101**, 15386-15391 (2004).

109. Benton MJ. Fossil Record 2. (1993).

110. De Bie T, Cristianini N, Demuth JP, Hahn MW. CAFE: a computational tool for the study of gene family evolution. *Bioinformatics* **22**, 1269-1271 (2006).

111. Zakharov EV, Caterino MS, Sperling FA. Molecular phylogeny, historical biogeography, and divergence time estimates for swallowtail butterflies of the genus Papilio (Lepidoptera: Papilionidae). *Systematic biology* **53**, 193-215 (2004).

112. Wahlberg N*, et al.* Nymphalid butterflies diversify following near demise at the Cretaceous/Tertiary boundary. *Proceedings Biological sciences / The Royal Society* **276**, 4295-4302 (2009).

113. Heikkila M, Kaila L, Mutanen M, Pena C, Wahlberg N. Cretaceous origin and repeated tertiary

diversification of the redefined butterflies. *Proceedings Biological sciences / The Royal Society* **279**, 1093-1099 (2012).

114. Jin L, Nei M. Limitations of the Evolutionary Parsimony Method of Phylogenetic Analysis. *Molecular biology and evolution* **7**, 82-102 (1990).

115. Penn O, Privman E, Ashkenazy H, Landan G, Graur D, Pupko T. GUIDANCE: a web server for assessing alignment confidence scores. *Nucleic acids research* **38**, W23-28 (2010).

116. Privman E, Penn O, Pupko T. Improving the performance of positive selection inference by filtering unreliable alignment regions. *Molecular biology and evolution* **29**, 1-5 (2012).

117. Peng Z*, et al.* Comprehensive analysis of RNA-Seq data reveals extensive RNA editing in a human transcriptome. *Nature biotechnology* **30**, 253-260 (2012).

118. Trapnell C, Pachter L, Salzberg SL. TopHat: discovering splice junctions with RNA-Seq. *Bioinformatics* **25**, 1105-1111 (2009).

119. Trapnell C*, et al.* Transcript assembly and quantification by RNA-Seq reveals unannotated transcripts and isoform switching during cell differentiation. *Nature biotechnology* **28**, 511-515 (2010).

120. Mortazavi A, Williams BA, McCue K, Schaeffer L, Wold B. Mapping and quantifying mammalian transcriptomes by RNA-Seq. *Nature methods* **5**, 621-628 (2008).

121. Li RQ*, et al.* De novo assembly of human genomes with massively parallel short read sequencing. *Genome research* **20**, 265-272 (2010).

122. Boetzer M, Henkel CV, Jansen HJ, Butler D, Pirovano W. Scaffolding pre-assembled contigs using SSPACE. *Bioinformatics* **27**, 578-579 (2011).

123. Audic S, Claverie JM. The significance of digital gene expression profiles. *Genome research* **7**, 986-995 (1997).

124. Robinson MD, Oshlack A. A scaling normalization method for differential expression analysis of RNA-seq data. *Genome biology* **11**, R25 (2010).

125. Zhang YL, Li ZX. Functional analysis and molecular docking identify two active short-chain prenyltransferases in the green peach aphid, Myzus persicae. *Archives of insect biochemistry and physiology* **81**, 63-76 (2012).

126. Vandermoten S*, et al.* Characterization of a novel aphid prenyltransferase displaying dual geranyl/farnesyl diphosphate synthase activity. *FEBS letters* **582**, 1928-1934 (2008).

127. Vandermoten S, Haubruge E, Cusson M. New insights into short-chain prenyltransferases: structural features, evolutionary history and potential for selective inhibition. *Cellular and molecular life sciences : CMLS* **66**, 3685-3695 (2009).

128. Szkopinska A, Plochocka D. Farnesyl diphosphate synthase; regulation of product specificity. *Acta biochimica Polonica* **52**, 45-55 (2005).

129. Wang KC, Ohnuma S. Isoprenyl diphosphate synthases. *Biochimica et biophysica acta* **1529**, 33-48 (2000).

130. Lai C, McMahon R, Young C, Mackay TF, Langley CH. quemao, a Drosophila bristle locus, encodes geranylgeranyl pyrophosphate synthase. *Genetics* **149**, 1051-1061 (1998).

131. Sen SE, Trobaugh C, Beliveau C, Richard T, Cusson M. Cloning, expression and characterization of a dipteran farnesyl diphosphate synthase. *Insect biochemistry and molecular biology* **37**, 1198-1206 (2007).

132. Kinjoh T, Kaneko Y, Itoyama K, Mita K, Hiruma K, Shinoda T. Control of juvenile hormone biosynthesis in Bombyx mori: cloning of the enzymes in the mevalonate pathway and assessment of their developmental expression in the corpora allata. *Insect biochemistry and molecular biology* **37**, 808-818 (2007).

133. Castillo-Gracia M, Couillaud F. Molecular cloning and tissue expression of an insect farnesyl diphosphate synthase. *European journal of biochemistry / FEBS* **262**, 365-370 (1999).

134. Cusson M*, et al.* Characterization and tissue-specific expression of two lepidopteran farnesyl diphosphate synthase homologs: implications for the biosynthesis of ethyl-substituted juvenile hormones. *Proteins* **65**, 742-758 (2006).

135. Gilg AB, Bearfield JC, Tittiger C, Welch WH, Blomquist GJ. Isolation and functional expression of an animal geranyl diphosphate synthase and its role in bark beetle pheromone biosynthesis. *Proceedings of the National Academy of Sciences of the United States of America* **102**, 9760-9765 (2005).

136. Taban AH, Tittiger C, Blomquist GJ, Welch WH. Isolation and characterization of farnesyl diphosphate synthase from the cotton boll weevil, Anthonomus grandis. *Archives of insect biochemistry and physiology* **71**, 88-104 (2009).

137. Frick S*, et al.* Metal ions control product specificity of isoprenyl diphosphate synthases in the insect terpenoid pathway. *Proceedings of the National Academy of Sciences of the United States of America* **110**, 4194-4199 (2013).

138. Vandermoten S*, et al.* Structural features conferring dual geranyl/farnesyl diphosphate synthase activity to an aphid prenyltransferase. *Insect biochemistry and molecular biology* **39**, 707-716 (2009).

139. Zhang YL, Li ZX. Two different farnesyl diphosphate synthase genes exist in the genome of the green peach aphid, Myzus persicae. *Genome / National Research Council Canada = Genome / Conseil national de recherches Canada* **51**, 501-510 (2008).

140. Hojo M, Matsumoto T, Miura T. Cloning and expression of a geranylgeranyl diphosphate synthase gene: insights into the synthesis of termite defence secretion. *Insect molecular biology* **16**, 121-131 (2007).

141. Hojo M, Toga K, Watanabe D, Yamamoto T, Maekawa K. High-level expression of the Geranylgeranyl diphosphate synthase gene in the frontal gland of soldiers in Reticulitermes speratus (Isoptera: Rhinotermitidae). *Archives of insect biochemistry and physiology* **77**, 17-31 (2011).

142. Bergot BJ, Jamieson GC, Ratcliff MA, Schooley DA. JH Zero: New Naturally Occurring Insect Juvenile Hormone from Developing Embryos of the Tobacco Hornworm. *Science* **210**, 336-338 (1980).

143. Brindle PA, Baker FC, Tsai LW, Reuter CC, Schooley DA. Sources of propionate for the biogenesis of ethyl-branched insect juvenile hormones: Role of isoleucine and valine. *Proceedings of the National Academy of Sciences of the United States of America* **84**, 7906-7910 (1987).

144. Brindle PA, Schooley DA, Tsai LW, Baker FC. Comparative metabolism of branched-chain amino acids to precursors of juvenile hormone biogenesis in corpora allata of lepidopterous versus nonlepidopterous insects. *The Journal of biological chemistry* **263**, 10653-10657 (1988).

145. Feyereisen R. Insect P450 enzymes. *Annual review of entomology* **44**, 507-533 (1999).

146. Schuler MA. The role of cytochrome P450 monooxygenases in plant-insect interactions. *Plant physiology* **112**, 1411-1419 (1996).

147. Danielson PB, MacIntyre RJ, Fogleman JC. Molecular cloning of a family of xenobiotic-inducible drosophilid cytochrome p450s: evidence for involvement in host-plant allelochemical resistance. *Proceedings of the National Academy of Sciences of the United States of America* **94**, 10797-10802 (1997).

148. Berenbaum MR. Postgenomic chemical ecology: from genetic code to ecological interactions. *Journal of chemical ecology* **28**, 873-896 (2002).

149. Li X, Baudry J, Berenbaum MR, Schuler MA. Structural and functional divergence of insect CYP6B proteins: From specialist to generalist cytochrome P450. *Proceedings of the National Academy of Sciences of the United States of America* **101**, 2939-2944 (2004).

150. Li W, Schuler MA, Berenbaum MR. Diversification of furanocoumarin-metabolizing cytochrome P450 monooxygenases in two papilionids: Specificity and substrate encounter rate. *Proceedings of the National Academy of Sciences of the United States of America* **100 Suppl 2**, 14593-14598 (2003).

151. Wen Z, Rupasinghe S, Niu G, Berenbaum MR, Schuler MA. CYP6B1 and CYP6B3 of the black swallowtail (Papilio polyxenes): adaptive evolution through subfunctionalization. *Molecular biology and evolution* **23**, 2434-2443 (2006).

152. Li W, Berenbaum MR, Schuler MA. Molecular analysis of multiple CYP6B genes from polyphagous Papilio species. *Insect biochemistry and molecular biology* **31**, 999-1011 (2001).

153. Zhou J, Zhang G, Zhou Q. Molecular characterization of cytochrome P450 CYP6B47 cDNAs and 5'-flanking sequence from Spodoptera litura (Lepidoptera: Noctuidae): its response to lead stress. *Journal of insect physiology* **58**, 726-736 (2012).

154. Peroutka R, Schulzova V, Botek P, Hajslova J. Analysis of furanocoumarins in vegetables (Apiaceae) anal citrus fruits (Rutaceae). *Journal of the science of food and agriculture* **87**, 2152-2163 (2007).

155. Berenbaum MR. The chemistry of defense: theory and practice. *Proceedings of the National Academy of Sciences of the United States of America* **92**, 2-8 (1995).

156. Li X, Schuler MA, Berenbaum MR. Molecular mechanisms of metabolic resistance to synthetic and natural xenobiotics. *Annual review of entomology* **52**, 231-253 (2007).

157. Yu Q, *et al.* Identification, genomic organization and expression pattern of glutathione S-transferase in the silkworm, Bombyx mori. *Insect biochemistry and molecular biology* **38**, 1158-

1164 (2008).

158. Yu QY, Lu C, Li WL, Xiang ZH, Zhang Z. Annotation and expression of carboxylesterases in the silkworm, Bombyx mori. *BMC genomics* **10**, 553 (2009).

159. Loytynoja A, Goldman N. webPRANK: a phylogeny-aware multiple sequence aligner with interactive alignment browser. *BMC bioinformatics* **11**, 579 (2010).

160. Loytynoja A, Goldman N. webPRANK: a phylogeny-aware multiple sequence aligner with interactive alignment browser. *Bmc Bioinformatics* **11**, (2010).

161. Ferguson LC, Green J, Surridge A, Jiggins CD. Evolution of the insect yellow gene family. *Molecular biology and evolution* **28**, 257-272 (2011).

162. Takahashi A. Pigmentation and behavior: potential association through pleiotropic genes in Drosophila. *Genes Genet Syst* **88**, 165-174 (2013).

163. Sakai RK, Baker RH, Iqbal MP. Genetics of Ebony, a Nonlethal Recessive Melanotic Mutant in a Mosquito. *Journal of Heredity* **63**, 275-& (1972).

164. Brehme KS. The effect of adult body color mutations upon the larva of Drosophila melanogaster. *P Natl Acad Sci USA* **27**, 254-261 (1941).

165. Futahashi R*, et al.* yellow and ebony Are the Responsible Genes for the Larval Color Mutants of the Silkworm Bombyx Mori. *Genetics* **180**, 1995-2005 (2008).

166. Pool JE, Aquadro CF. The genetic basis of adaptive pigmentation variation in Drosophila melanogaster. *Mol Ecol* **16**, 2844-2851 (2007).

167. Wittkopp PJ, True JR, Carroll SB. Reciprocal functions of the Drosophila Yellow and Ebony proteins in the development and evolution of pigment patterns. *Development* **129**, 1849-1858 (2002).

168. Rebeiz M, Pool JE, Kassner VA, Aquadro CF, Carroll SB. Stepwise Modification of a Modular Enhancer Underlies Adaptation in a Drosophila Population. *Science* **326**, 1663-1667 (2009).

169. Wittkopp PJ*, et al.* Intraspecific Polymorphism to Interspecific Divergence: Genetics of Pigmentation in Drosophila. *Science* **326**, 540-544 (2009).

170. Koch PB, Behnecke B, Weigmann-Lenz M, Ffrench-Constant RH. Insect pigmentation: Activities of beta-alanyldopamine synthase in wing color patterns of wild-type and melanic mutant swallowtail butterfly Papilio glaucus. *Pigm Cell Res* **13**, 54-58 (2000).

171. Oxford GS, Gillespie RG. Evolution and ecology of spider coloration. *Annual review of entomology* **43**, 619-643 (1998).

172. Martel RR, Law JH. Purification and Properties of an Ommochrome-Binding Protein from the Hemolymph of the Tobacco Hornworm, Manduca-Sexta. *Journal of Biological Chemistry* **266**, 21392-21398 (1991).

173. Yepizplascencia GM, Ho CM, Martel RR, Law JH. Molecular-Cloning and Sequence of a Novel Ommochrome-Binding Protein Cdna from an Insect, Manduca-Sexta. *Journal of Biological Chemistry* **268**, 2337-2340 (1993).

174. Coates BS, Hellmich RL, Lewis LC. Two differentially expressed ommochrome-binding protein-like genes (obp1 and obp2) in larval fat body of the European corn borer, Ostrinia nubilalis. *Journal of Insect Science* **5**, (2005).

175. Wiley K, Forrest HS. Drosophila-Melanogaster Lacks Eye-Pigment Binding-Proteins. *Biochemistry* **18**, 473-476 (1979).

176. Schmidt FS, Skerra A. The bilin-binding protein of Pieris brassicae. cDNA sequence and regulation of expression reveal distinct features of this insect pigment protein. *European journal of biochemistry / FEBS* **219**, 855-863 (1994).

177. Li W, Riddiford LM. Two distinct genes encode two major isoelectric forms of insecticyanin in the tobacco hornworm, Manduca sexta. *European journal of biochemistry / FEBS* **205**, 491-499 (1992).

178. Zagalsky PF, Cheesman DF. Purification and Properties of Crustacyanin. *Biochemical Journal* **89**, P21-& (1963).

179. Shirataki H, Futahashi R, Fujiwara H. Species-specific coordinated gene expression and trans-regulation of larval color pattern in three swallowtail butterflies. *Evolution & development* **12**, 305-314 (2010).

180. Futahashi R, Shirataki H, Narita T, Mita K, Fujiwara H. Comprehensive microarray-based analysis for stage-specific larval camouflage pattern-associated genes in the swallowtail butterfly, Papilio xuthus. *BMC biology* **10**, 46 (2012).

181. Goodrich LV, Strutt D. Principles of planar polarity in animal development. *Development* **138**, 1877-1892 (2011).

182. Lawrence PA, Struhl G, Casal J. Planar cell polarity: one or two pathways? *Nature Reviews Genetics* **8**, 555-563 (2007).

183. Thomas C, Strutt D. The roles of the cadherins Fat and Dachsous in planar polarity specification in Drosophila. *Developmental dynamics : an official publication of the American Association of Anatomists* **241**, 27-39 (2012).

184. Adler PN. The frizzled/stan pathway and planar cell polarity in the Drosophila wing. *Current topics in developmental biology* **101**, 1-31 (2012).

185. Simons M, Mlodzik M. Planar cell polarity signaling: from fly development to human disease. *Annual review of genetics* **42**, 517-540 (2008).

186. Kunte K*, et al.* doublesex is a mimicry supergene. *Nature* **507**, 229-+ (2014).

187. Huang W, Li L, Myers JR, Marth GT. ART: a next-generation sequencing read simulator. *Bioinformatics* **28**, 593-594 (2012).

188. Gaj T, Gersbach CA, Barbas CF, 3rd. ZFN, TALEN, and CRISPR/Cas-based methods for genome engineering. *Trends in biotechnology* **31**, 397-405 (2013).

189. Cong L*, et al.* Multiplex Genome Engineering Using CRISPR/Cas Systems. *Science* **339**, 819-823 (2013).

190. Jiang WY, Bikard D, Cox D, Zhang F, Marraffini LA. RNA-guided editing of bacterial genomes using

CRISPR-Cas systems. *Nature biotechnology* **31**, 233-239 (2013).

191. Wang HY*, et al.* One-Step Generation of Mice Carrying Mutations in Multiple Genes by CRISPR/Cas-Mediated Genome Engineering. *Cell* **153**, 910-918 (2013).

192. McMillan WOM, A.; Kapan, D. D. Development and evolution on the wing. *Trends in ecology & evolution* **17**, 6 (2002).

193. Tomita S, Kikuchi A. Abd-B suppresses lepidopteran proleg development in posterior abdomen. *Developmental biology* **328**, 403-409 (2009).

194. Celniker SE, Keelan DJ, Lewis EB. The Molecular-Genetics of the Bithorax Complex of Drosophila - Characterization of the Products of the Abdominal-B Domain. *Genes & development* **3**, 1424-1436 (1989).

195. Fu YF*, et al.* High-frequency off-target mutagenesis induced by CRISPR-Cas nucleases in human cells. *Nature biotechnology* **31**, 822-+ (2013).

196. Hsu PD*, et al.* DNA targeting specificity of RNA-guided Cas9 nucleases. *Nature biotechnology* **31**, 827-832 (2013).

197. Pattanayak V, Lin S, Guilinger JP, Ma E, Doudna JA, Liu DR. High-throughput profiling of off-target DNA cleavage reveals RNA-programmed Cas9 nuclease specificity. *Nature biotechnology* **31**, 839-843 (2013).

198. Mali P*, et al.* CAS9 transcriptional activators for target specificity screening and paired nickases for cooperative genome engineering. *Nature biotechnology* **31**, 833-+ (2013).

199. Lin YN*, et al.* CRISPR/Cas9 systems have off-target activity with insertions or deletions between target DNA and guide RNA sequences. *Nucleic acids research* **42**, 7473-7485 (2014).

200. Xiao A*, et al.* CasOT: a genome-wide Cas9/gRNA off-target searching tool. *Bioinformatics* **30**, 1180-1182 (2014).

201. Bae S, Park J, Kim JS. Cas-OFFinder: a fast and versatile algorithm that searches for potential off-target sites of Cas9 RNA-guided endonucleases. *Bioinformatics* **30**, 1473-1475 (2014).

202. Cradick TJ, Qiu P, Lee CM, Fine EJ, Bao G. COSMID: A Web-based Tool for Identifying and Validating CRISPR/Cas Off-target Sites. *Mol Ther-Nucl Acids* **3**, (2014).

203. Yang LH*, et al.* Targeted and genome-wide sequencing reveal single nucleotide variations impacting specificity of Cas9 in human stem cells. *Nature communications* **5**, (2014).

204. Veres A*, et al.* Low Incidence of Off-Target Mutations in Individual CRISPR-Cas9 and TALEN Targeted Human Stem Cell Clones Detected by Whole-Genome Sequencing. *Cell Stem Cell* **15**, 27-30 (2014).
